# Electrophilic Fluorination of Alkenes via Bora‐Wagner–Meerwein Rearrangement. Access to β‐Difluoroalkyl Boronates

**DOI:** 10.1002/anie.202109461

**Published:** 2021-11-10

**Authors:** Qiang Wang, Maria Biosca, Fahmi Himo, Kálmán J. Szabó

**Affiliations:** ^1^ Department of Organic Chemistry Stockholm University Sweden

**Keywords:** boron, DFT modeling, fluorination, organic synthesis, rearrangement

## Abstract

The electrophilic fluorination of geminal alkyl substituted vinyl‐Bmida derivatives proceeds via bora‐Wagner–Meerwein rearrangement. According to DFT modelling studies this rearrangement occurs with a low activation barrier via a bora‐cyclopropane shaped TS. The Bmida group has a larger migration aptitude than the alkyl moiety in the Wagner–Meerwein rearrangement of the presented electrophilic fluorination reactions.

## Graphical abstract

Electrophilic fluorination of alkyl substituted vinyl‐Bmida species proceeds through bora‐Wagner–Meerwein type rearrangement.

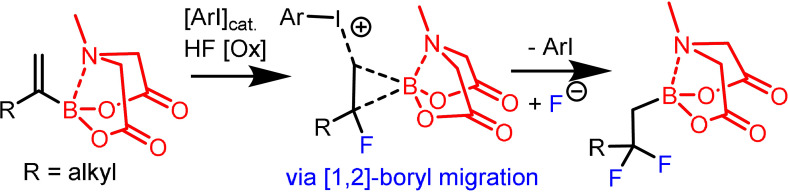

Synthetic boron and fluorine chemistries have received a lot of attention recently.^[1]^ Organoboron reagents are very attractive in synthesis of organofluorine compounds, which are employed in many fields of life‐sciences, such as in pharmaceutical, agrochemical and medical diagnostic areas.^[2]^ In a particularly interesting class of reagents the boron containing groups control the outcome of the fluorination reaction.^[3]^ Here, we present an electrophilic fluorination reaction of vinyl boronate reagents proceeding via MIDA boronate^[4]^ rearrangement.

The Wagner–Meerwein rearrangement is obviously one of the most important and most studied processes in organic chemistry. The most common is migration of alkyl/aryl groups and hydrogen between two vicinal carbon atoms (Figure 1 a).^[5]^ The groups of Yudin^[6]^ and Burke^[7]^ reported interesting Meinwald‐type rearrangements^[8]^ of oxiranyl MIDA boronates (Figure 1 b). In this reaction the MIDA boronate group undergoes a [1,2] migration process, which is very similar to the H/alkyl migration to electron deficient carbon centers. Mechanistic studies by Yudin and co‐workers^[9]^ revealed that the high migration aptitude of MIDA boronate in this [1,2] process is due to the hemilabile bonding of nitrogen to boron in the Bmida group. Interestingly, this B‐N hemilability lends a migration aptitude to Bmida group, which is in the same magnitude as the ability of H/alkyl/aryl groups to undergo [1,2] migration to electron deficient carbon centers.^[10]^ Electrophilic fluorination reactions are also known to proceed via carbocations or electron‐deficient carbon centers. In particular, fluorination of alkenes with hypervalent iodines proceed via these intermediates. Previous studies have shown^[11]^ that fluorination of styrene derivatives with hypervalent iodines usually proceeds via rearrangement involving cationic phenonium ion intermediates (Figure 1 c). Recently, the Jacobsen group^[11f]^ reported an asymmetric 1,3‐difluorinative Wagner–Meerwein rearrangement of β‐substituted styrene derivatives (Figure 1 d). In this process the electrophilic fluorination by hypervalent iodines proceeds through a carbocation intermediate, which undergoes [1,2] migration of an alkyl or aryl group.^[11f]^ Wang and co‐workers^[11i]^ presented a study on electrophilic fluorination of styryl boronate derivatives (Figure 1 e) affording geminal difluorinated products.

**Figure 1:**
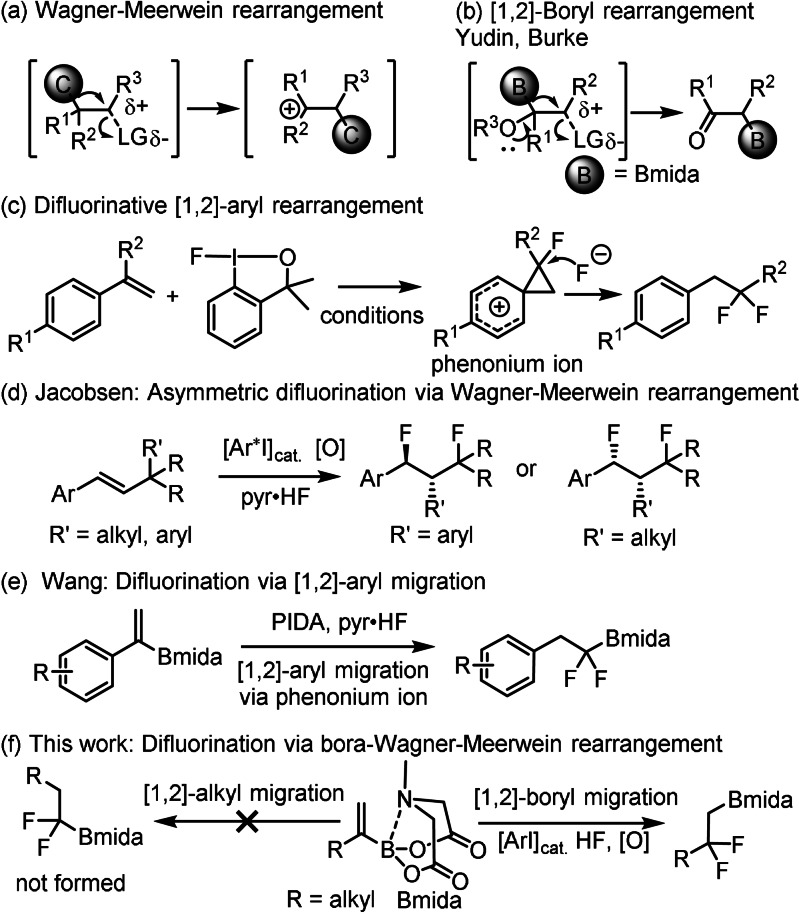
Reactions occurring with [1.2]‐aryl/alkyl or boron migrations.

These substrates reacted by [1,2]‐aryl migration most probably via phenonium ion intermediates. Surprisingly, when a similar electrophilic fluorination reaction was performed with alkyl vinyl boronates instead of [1,2]‐alkyl migration a bora‐Wagner–Meerwein type [1,2]‐boryl migration occurred (Figure 1 f). This reaction is suitable for synthesis of geminal difluoroalkyl boronates. Both the difluoroalkyl group and alkyl/aryl boronates occur in important drug substances (Figure 2).^[12]^ Catalytic fluorination of alkyl vinyl boronates were developed (Table 1) using MIDA boronate **1 a** and Selectfluor in the presence of various HF sources and aryl iodide catalysts (**3 a–d**).[^11d^, ^11e^]

**Figure 2:**
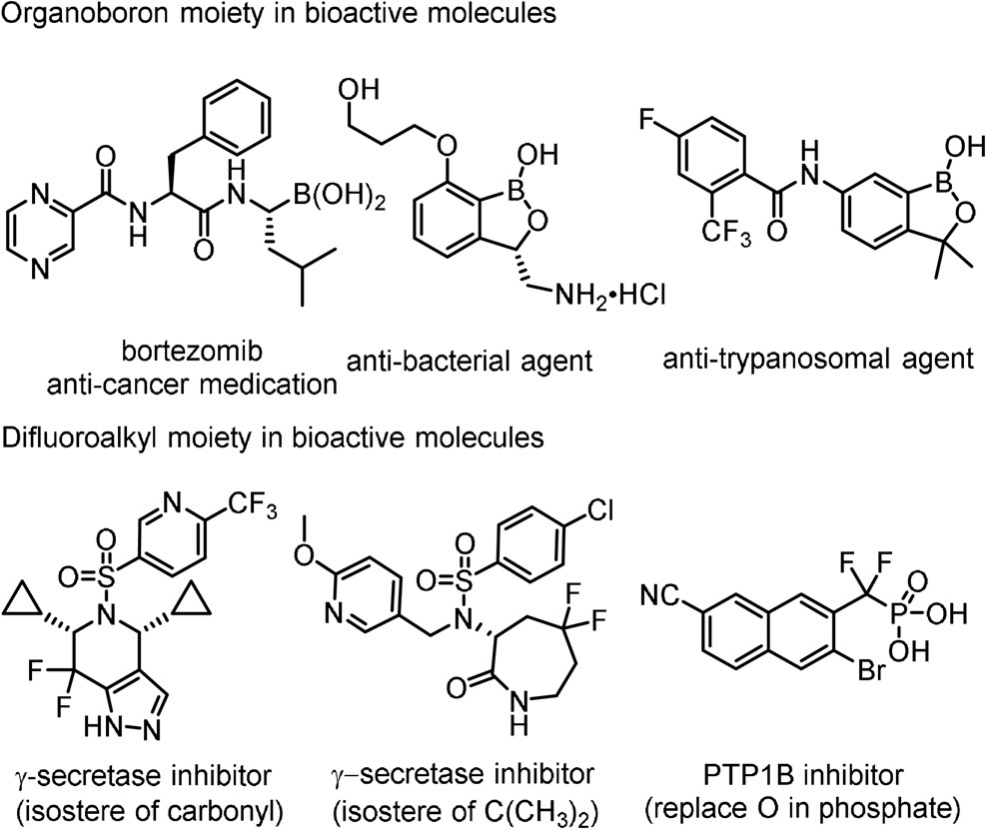
Examples for organoboron and difluoromethyl containing bioactive compounds.

**Table 1:** Optimization of the reaction conditions.^[a]^ 
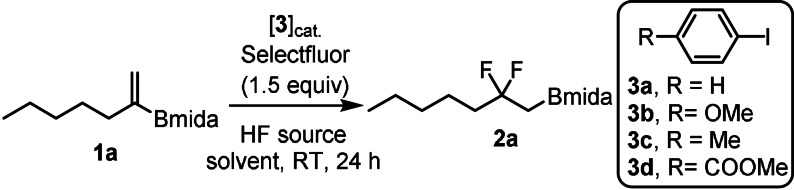

Entry	Catalyst	HF source^[b]^ (*x* equiv)	Solvent	Yield (%)^[c]^
1	**3 a**	pyr⋅9 HF (65)	CH_2_Cl_2_	12
2	**3 a**	TEA⋅3 HF (65)	CH_2_Cl_2_	0
3	**3 a**	**A** (65)	CH_2_Cl_2_	83
4	**3 a**	**B** (75)	CH_2_Cl_2_	53
5	**3 a**	**C** (55)	CH_2_Cl_2_	68
6	**3 b**	**A** (65)	CH_2_Cl_2_	81
7	**3 c**	**A** (65)	CH_2_Cl_2_	91 (69)^[d]^
8	**3 d**	**A** (65)	CH_2_Cl_2_	22
9	**3 c**	**A** (65)	CHCl_3_	72
10	**3 c**	**A** (65)	PhMe	44
11^[e]^	**3 c**	**A** (65)	CH_2_Cl_2_	25
12^[f]^	–	**A** (65)	CH_2_Cl_2_	0

[a] Unless otherwise stated: **1 a** (0.1 mmol), catalyst (0.02 mmol), Selectfluor (0.15 mmol) and HF source in 0.5 mL of solvent stirred at room temperature for 24 h. [b] Composition of the HF source: **A**=0.1 mL pyr⋅9 HF + 0.15 mL TEA⋅3 HF, **B**=0.1 mL pyr⋅9 HF + 0.2 mL TEA⋅3 HF, **C**=0.1 mL pyr⋅9 HF + 0.1 mL TEA⋅3 HF. [c] ^19^F NMR yields with fluorobenzene as an internal standard. [d] Isolated yield. [e] *m*CPBA was used instead of Selectfluor. [f] Without catalyst.

Using phenyl iodide as catalyst in the presence of pyr⋅9 HF as fluorine source, product **2 a** was formed in 12 % yield (Table 1, entry 1) with full consumption of starting material **1 a**. These results suggested that **1 a** or **2 a** were unstable under the applied reaction conditions. This prompted us to investigate the stability of starting material **1 a** and product **2 a** in the presence of HF‐pyridine. Monitoring the reaction with ^1^H NMR spectroscopy showed that starting material **1 a** was completely decomposed at RT in 24 h, while product **2 a** was reasonable stable [Eqs. 1–(2)]. We concluded that MIDA boronate in **1 a** probably underwent solvolysis^[13]^ and the products, such as the unprotected alkylboronic acid, rapidly decomposed. Protonation of the nitrogen in the Bmida group may trigger the solvolysis.^[13]^ Therefore, we attempted the fluorination reaction using TEA⋅3 HF (entry 2), which is less acidic than pyr⋅9 HF. Under these conditions **1 a** was stable but product **2 a** did not form. Gilmour and co‐workers have shown that the reactivity in the oxidative fluorination reactions is largely dependent on the source of hydrogen fluoride, especially when Selectfluor is used as oxidant.[^11d^, ^11e^, ^14^] Apparently, there is an optimal acidity, that is, HF vs. base concentration, for the presented reactions. Therefore, we carried out fluorination of **1 a** with different fluorine sources **A–C**, in which the composition of pyr⋅9 HF and TEA⋅3 HF was varied. When a mixture of 0.1 mL pyr⋅9 HF and 0.15 mL TEA⋅3 HF was used as fluoride source (HF source A), product **2 a** was obtained in 83 % yield with almost complete conversion of **1 a** (entry 3). Further variation of the HF/base by increase (HF source B) or decrease (HF source C) of the amount of TEA⋅3 HF vs. pyr⋅9 HF led to a decrease of the yields (entries 4 and 5). This indicates that HF source A is optimal for the reaction. Subsequently, we varied the aryl iodide catalysts.

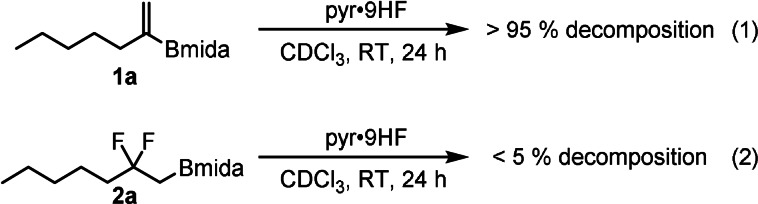




Methoxy iodobenzene (**3 b**) gave about the same yield as iodobenzene (**3 a**) (c.f. entries 6 and 3). However, application of iodotoluene **3 c** led to increase of the yield to 91 % (entry 7). Product **2 a** could be isolated by silica gel chromatography with some purification loss (69 % isolated yield). Catalyst **3 d** with electron‐withdrawing COOMe substituent was less efficient than **3 c**, as the yield dropped to 22 % (entry 8). When CH_2_Cl_2_ was replaced by toluene or CHCl_3_, the yield also decreased (c.f. entries 9/10 and 7). Application of *m*CPBA as oxidant was less efficient than Selectfluor, since the yield of the reaction decreased from 81 % to 25 %, when the oxidant was changed (c.f. entries 11 and 7). When the reaction was performed without iodoarene catalyst formation of **2 a** was not observed (entry 12).

With the optimal conditions (Table 1, entry 7) in hand, the synthetic scope of the reaction was studied using alkenyl‐Bmida derivatives **1 b**–**m**. Alkenyl‐Bmida derivatives with linear alkyl chain (**1 a**–**d**) reacted smoothly to give the rearranged products **2 a**–**d** in 50–69 % yields (entries 1–4). Notably, in all cases clean [1,2]‐boryl migration occurred, as formation of the isomeric product (see Figure 1 f) arising from the [1,2]‐alkyl migration was not observed. The presence of the bulky groups in the substrate, such as cyclohexyl (**1 e**) leads to lower yield of 45 % (entry 5). Phenyl alkyl substituted (**1 f**) and chloro (**1 g**) substrates gave the corresponding rearrangement products **2 f**,**g** with acceptable yields (45–57 %). Benzyl substituted product **2 h** formed in a poor yield of 19 % when HF source **A** [pyr⋅9 HF (0.1 mL) and TEA⋅3 HF (0.15 mL)] was employed (entry 8). In the crude reaction mixture large amounts of starting material **1 h** was detected. This suggested that the reaction proceeded slower with this substrate than with **1 a**–**g**. Therefore, a more acidic HF source, pyr⋅9 HF (without TEA⋅3 HF) was employed in the reaction (entry 9). In this case the yield increased substantially from 19 % to 86 % (c.f. entries 8 and 9). The relatively high yield indicates that **1 h** is more stable than **1 a** in the presence of pyr⋅9 HF and the fluorination reaction proceeds faster than in HF source **A**. In fact, the rate of decomposition of **1 h** [Eq. 3] found to be much slower than **1 a** [Eq. (1)]. We have found that other benzyl substituted substrates **1 i**,**j** had similar stability features in pyr⋅9 HF. The reaction of these substrates resulted in difluoro Bmida products **2 i**,**j** in 75 % and 40 % yields, respectively (entries 10–11). In addition, using pyr⋅9 HF as sole HF source phthalimide derivative **2 k** (63 %) and relatively bulky cyclohexyl derivative **2 l** (56 %) could be obtained in good yields (entries 12–13). When trisubstituted alkenyl‐Bmida derivative **1 m** was used as a substrate, the reaction gave a complex mixture, from which we could not isolate the expected rearrangement product **2 m** (entry 14). The reactions can easily be scaled up. For example, **2 d**, **2 k** and **2 l** were obtained at 1 mmol scale without significant change of the yields. The Bmida group in the product can be easily converted to Bpin group with excellent yield [Eq. (4)].

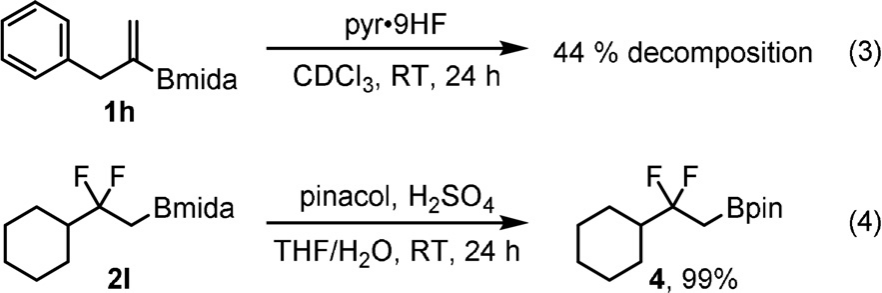




To gain insight into the mechanism of the above aryl iodide‐catalyzed electrophilic fluorination via the anticipated bora‐Wagner–Meerwein rearrangement, we performed density functional theory (DFT) calculations. In these modeling studies, benzyl substituted olefin (**1 h**) was used as a model substrate with iodoarene **3 c** as catalyst (see Table 2, entries 9–10). The calculations were carried out using the B3LYP‐D3(BJ) functional.^[15]^ Implicit solvation using the SMD^[16]^ model with the parameters for dichloromethane was included in the geometry optimizations (see SI for computational details, S103).

**Table 2:**
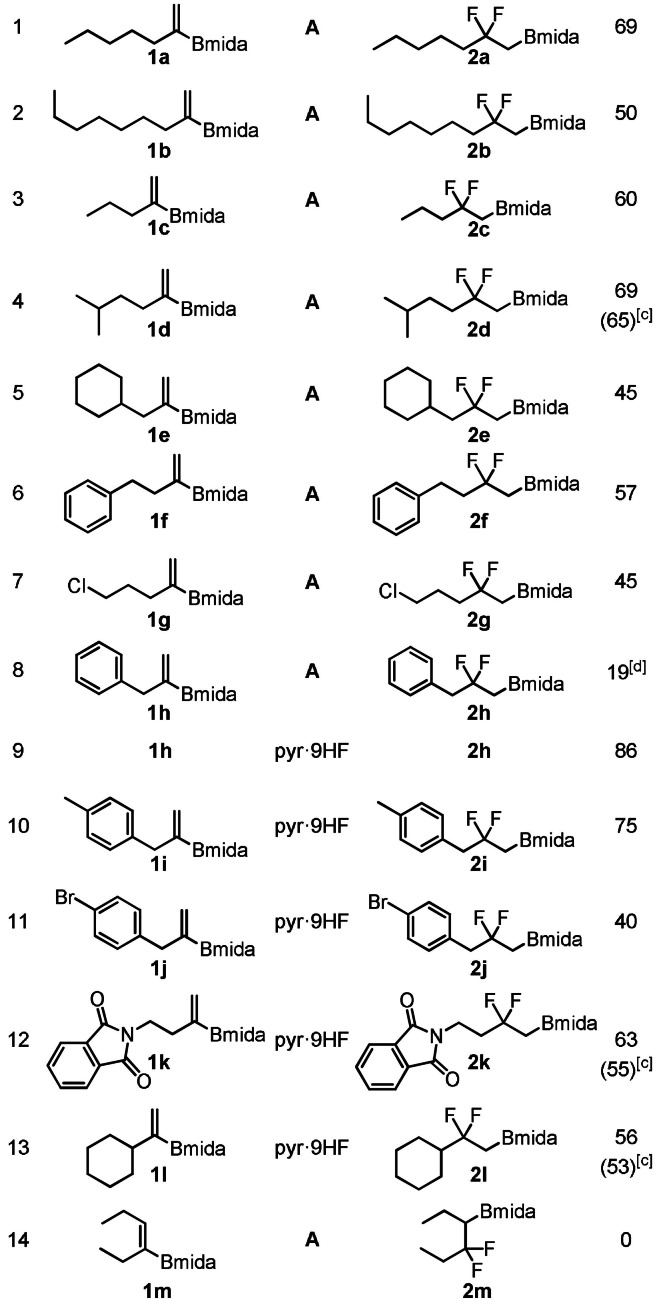
Scope of the *gem*‐difluorinative [1,2]‐boryl migration.^[a]^ 
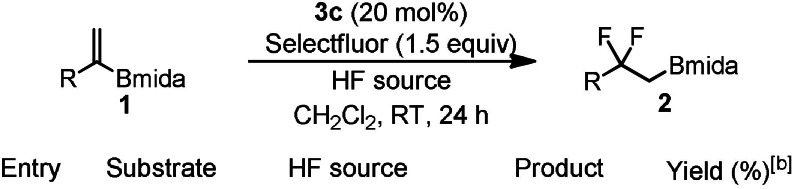

[a] Unless otherwise stated: **1** (0.1 mmol), **3 c** (0.02 mmol), Selectfluor (0.15 mmol), HF source **A**: pyr⋅9 HF (0.1 mL) and TEA⋅3 HF (0.15 mL) in CH_2_Cl_2_ (0.5 mL) stirred at room temperature for 24 h. [b] Isolated yield. [c] 1 mmol scale. [d] ^19^F NMR yield.

The associated free energy profile that emerges from the calculations is displayed in Figure 3. The optimized geometries of the intermediates and transition states and the catalytic cycle are given in the SI. Similarly to our previous calculations on the oxyfluorocyclization of styrene derivatives,^[17]^ some of the species (**Int1**, **Int2**, **Int4**, **TS2** and **Int5**) are modeled as ion‐pairs, consisting of a cationic catalyst species and an (HF)_2_F^−^ counterion.

**Figure 3:**
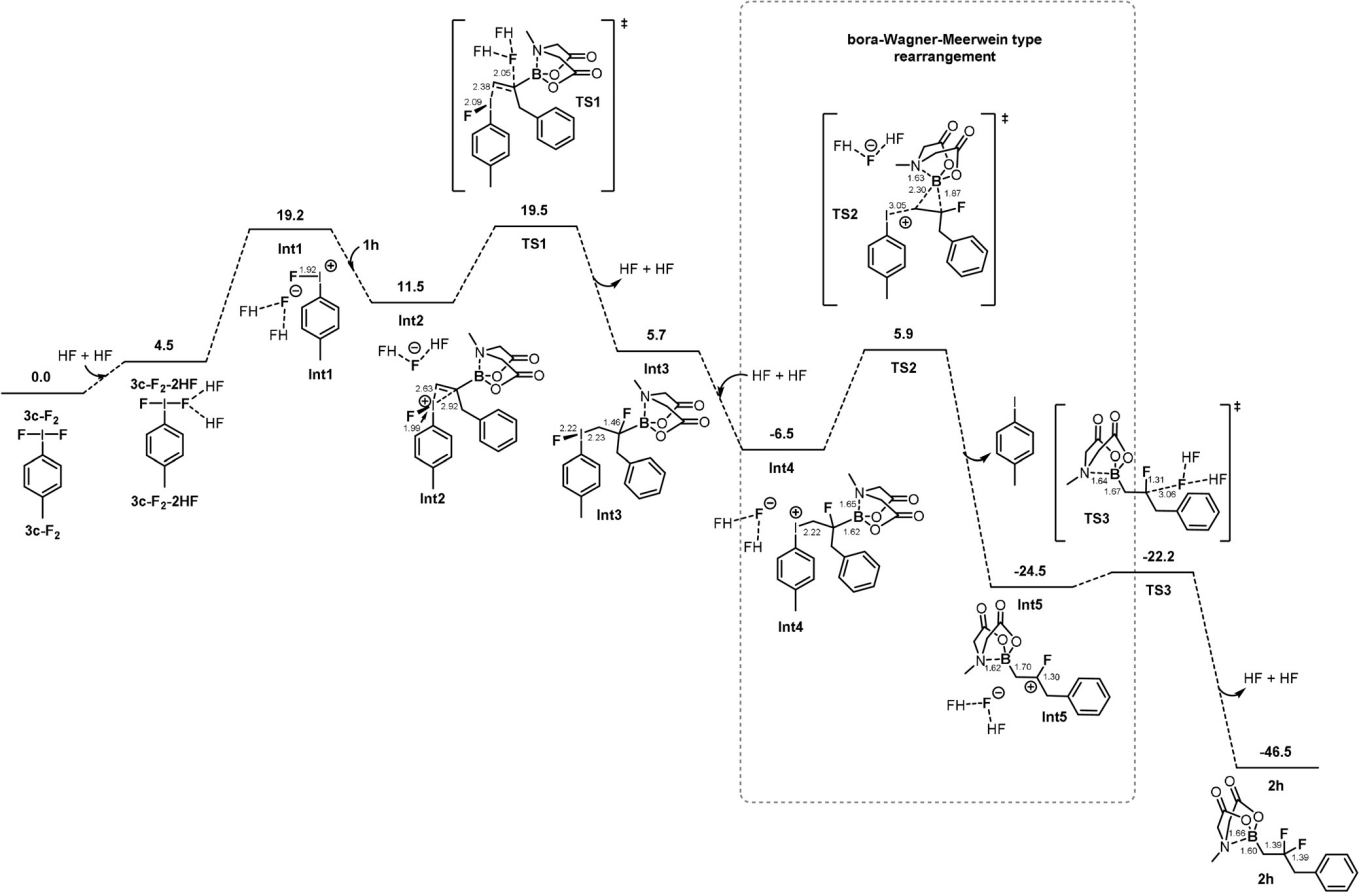
Calculated free energy profile (kcal mol^−1^) for the aryl iodide‐catalyzed fluorination of **1 h** with **3 c** occurring via bora‐Wagner–Meerwein rearrangement.

The first step of the cycle is the formation of **3 c‐F_2_** by oxidation and fluorination of iodoarene **3 c** using Selectfluor and HF‐amine source. Modeling of the energetics of this step is associated with large uncertainties and was not considered explicitly by the calculations. However, the formation of **3 c‐F_2_** with the Selectfluor protocol is supported by experimental evidences and can be assumed to take place readily.^[18]^

Activation of **3 c‐F_2_** takes place to generate the cationic fluoroiodonium active catalytic species **Int1**. Similarly to previous computational studies involving hypervalent iodines,[^11h^, ^17^] we employed two HF molecules for modelling the activation of iodoarene difluoride. First, the two HF molecules coordinate to the iodoarene difluoride (**3 c‐F_2_**), giving the hydrogen‐bonded difluoride intermediate **3 c‐F_2_‐2HF**. This complex is 4.5 kcal mol^−1^ higher than **3 c‐F_2_**. Abstraction of the fluoride then takes place to give **Int1**. A transition state for this step could be located but after addition of the energy corrections the resulting Gibbs free energy of this TS was slightly lower than the following intermediate. Therefore, the calculated endergonicity of the step, amounting to 19.2 kcal mol^−1^, can be considered as the barrier for this transformation. Next, the coordination of Bmida substrate **1 h** to **Int1** takes place to provide iodonium ion intermediate **Int2**, which results in a lowering of the energy by 7.7 kcal mol^−1^. Then, (HF)_2_F^−^ attacks the olefin on the most substituted carbon through **TS1**. This nucleophilic attack has a barrier of 8.0 kcal mol^−1^ relative to **Int2** and results in the formation of **Int3**, in which two new σ‐bonds are formed (C−I at 2.23 Å and C−F at 1.46 Å) and the double bond of the alkene is converted to a single bond. The reverse regiochemistry, including the nucleophilic attack at the less substituted carbon of the olefin, was also considered but the activation energy was higher by 3 kcal mol^−1^ than for **TS1** (see SI, S106).

The formation of the C−I bond in **Int3**, weakens the I‐F bond, which is elongated from 1.99 Å to 2.22 Å. The fluoride is then readily abstracted by two HF molecules to yield **Int4**, which is 12.2 kcal mol^−1^ lower in energy than **Int3**. For this step a TS could not be located, but considering the elongation of the I−F bond in **Int3** and the exergonicity of the step, the barrier is expected to be very low. In **Int4** the carbon atom attached to the positively charged iodine is electron deficient and iodotoluene (**3 c**) is obviously an excellent leaving group. These factors pave the road for a bora‐Wagner–Meerwein type [1,2]‐boryl migration of Bmida group.

From **Int4**, the bora‐Wagner–Meerwein rearrangement occurs via **TS2**, with a barrier of 12.4 kcal mol^−1^. Similarly to the studies of the groups of Yudin^[9]^ and Pellegrinet^[10]^ the migration occurs via a bora‐cyclopropane type structure (**TS2**). Formation of a three‐membered ring‐shaped TS including electron deficient carbon centers represents a clear analogy to the Wagner–Meerwein rearrangement involving aryl/alkyl/H groups.^[19]^ The migration step leads to formation of carbocation **Int5** and the release of the iodoarene catalyst **3 c**. Notably, the C−F bond (1.30 Å) is relatively short indicating a C(pπ*)‐F(n_π_) type of stabilization of the carbocation center. This stabilization can be regarded as an additional driving force for the [1,2]‐boryl migration. We have also considered the possibility of the competing [1,2]‐alkyl (benzyl) migration in **Int4** but the activation barrier was higher by 6.9 kcal mol^−1^ than for **TS2**. Another alternative pathway (leading to vicinal difluorination without Bmida rearrangement) is an initial attack of (HF)_2_F^−^ of **Int4**. In this case the activation energy is 4.0 kcal mol^−1^ above **TS2** (see SI, S107).

These results are in line with the above experimental findings, as we did not observe formation of the isomeric [1,2]‐alkyl migration products (see Figure 1 f) or vicinal difluorinated species. In conclusion, the migration aptitude of the MIDA boronate group is apparently larger than the alkyl group in the above electrophilic fluorination reactions of α‐substituted alkenyl‐Bmida substrates, such as **1 h**. Taking into consideration the results by Wang and co‐workers^[11i]^ on [1,2]‐aryl migration of aryl vinyl‐Bmida derivatives (Figure 1 e), the expected order of the migration aptitude in electrophilic fluorination of geminally substituted vinyl‐Bmida substrates is aryl > Bmida > alkyl. We have also performed calculations on representative cases of aryl vs. Bmida group migration. These studies show that the energy differences between the aryl and Bmida migrations are relatively small and also depend on the substituents of the aryl group (see SI, S108). Finally, carbocation **Int5** undergoes a nucleophilic attack by the (HF)_2_F^−^, resulting in the final product **2 h**, which is 24.3 kcal mol^−1^ lower in energy than **Int5**. This transformation occurs via **TS3**, with a very low barrier of 2.3 kcal mol^−1^.

In summary, we presented a catalytic electrophilic fluorination reaction of geminal alkyl substituted vinyl‐Bmida derivatives. The reaction proceeds via bora‐Wagner–Meerwein type [1,2]‐boryl migration. As far as we know, this is the first example for electrophilic fluorination reactions occurring via [1,2]‐boryl migration. The products of the reactions are difluoroalkyl boronates. Both motifs are important pharmacophores in bioactive substances (Figure 2). DFT calculations revealed that the migration proceeds through a low activation barrier via a bora‐cyclopropane shaped TS. The migration aptitude of the Bmida group is higher than the alkyl group in electrophilic fluorination of alkenyl‐Bmida species. Our assumption is that other vinyl boron derivatives may also undergo similar rearrangement reactions. However, a hemilabile bonding between the boron and a Lewis base, such as the hemilabile B−N bonding^[9]^ in the Bmida group, is probably important for a high migration aptitude.

## Conflict of interest

The authors declare no conflict of interest.

## Supporting information

As a service to our authors and readers, this journal provides supporting information supplied by the authors. Such materials are peer reviewed and may be re‐organized for online delivery, but are not copy‐edited or typeset. Technical support issues arising from supporting information (other than missing files) should be addressed to the authors.

Supporting Information
