# Supplementary material for: Electrophilic Fluorination of Alkenes via Bora‐Wagner–Meerwein Rearrangement. Access to β‐Difluoroalkyl Boronates
Source: Angew Chem Int Ed Engl. 2021 Nov 10;60(50):26327–31. doi: 10.1002/anie.202109461 (PMC9299629; doi:10.1002/anie.202109461)
Supplement: Supplementary file 1 — Supporting Information [file ANIE-60-26327-s001.pdf]

Angewandte Chemie
Eine Zeitschrift der Gesellschaft Deutscher Chemiker

Supporting Information

Electrophilic Fluorination of Alkenes via Bora-Wagner–Meerwein Rearrangement. Access to β-Difluoroalkyl Boronates

Qiang Wang[+], Maria Biosca[+], Fahmi Himo, and Kálmán J. Szabó**

anie_202109461_sm_miscellaneous_information.pdf

Supporting Information

<u>Contents:</u>

Supporting Information for the Experimental Studies

**Page S3**	General information
**Page S4**	Experimental procedures and spectroscopic data
**Page S4**	Procedure for the synthesis of alkenyl MIDA boronates **1a**, **1d**, **1e**, **1h** and **1j**
**Page S7**	Procedure for the synthesis of alkenyl MIDA boronate **1b**
**Page S9**	Procedure for the synthesis of alkenyl MIDA boronate **1c**
**Page S10**	Procedure for the synthesis of alkenyl MIDA boronates **1f** and **1l**
**Page S13**	Procedure for the synthesis of alkenyl MIDA boronate **1g**
**Page S15**	Procedure for the synthesis of alkenyl MIDA boronate **1i**
**Page S17**	Procedure for the synthesis of alkenyl MIDA boronate **1k**
**Page S19**	General procedure for fluorination of alkyl vinyl-Bmida derivatives
**Page S24**	Control experiments for stability of **1a**, **2a** and **1h** in pyr·9HF
**Page S25**	Transformation of Bmida group to Bpin group
**Page S26**	^{1}H NMR, ^{13}C NMR, ^{19}F NMR and ^{11}B NMR spectra of substrates and products

Supporting Information for the Computational Studies

Page S103 Computational details

Page S104 Catalytic cycle

Page S105 Optimized structures of intermediates and transition states

Page S106 TS for the nucleophilic attack at the less substituted carbon of **1h**

Page S107 Alternative mechanism Wagner-Meerwein rearrangement of the alkyl group and nucleophilic attack of the $(HF)_2F^-$ in **Int4**

Page S108 Energetics of the TSs for aryl/alkyl migration vs Bmida migration

Page S109 Absolute energies and energy corrections

Page S110 Cartesian coordinates

Page S117 References

Supporting Information for the Experimental Studies

General Information

Reagents were used as obtained from commercial suppliers without further purification. Selectfluor®, 4-iodotoluene, pyr·9HF, TEA·3HF, *N*-methyliminodiacetic acid were obtained from Sigma-Aldrich. MeCN, CH_2Cl_2 and toluene were dried by a solvent purification system (VAC Solvent Purifier from Vacuum Atmospheres Company). Dry DMSO and THF were purchased from Sigma-Aldrich. Flash chromatography was carried out using 60 Å (35-70 μm mesh) silica gel (VWR) together with eluent systems based on hexanes / EtOAc mixtures or pure EtOAc. Analytical TLC was carried out on aluminum-backed plates (1.5 Å, ~ 5 cm) pre-coated (0.25 mm) with silica gel (Merck, Silica Gel 60 F254). Compounds were visualized by exposure to UV light (254 nm) or by dipping the plates into a solution of 0.75% $KMnO_4$ (w/w) in an aqueous solution of K_2CO_3 0.36 M. Melting points were recorded in a metal block instrument and are uncorrected. ^{1}H NMR spectra were recorded at 400 MHz; ^{13}C NMR spectra were recorded at 100 MHz; ^{19}F NMR spectra were recorded at 377 MHz and ^{11}B NMR spectra were recorded at 128 MHz with a Bruker Advance spectrometer. ^{1}H and ^{13}C NMR chemical shifts (δ) are reported in ppm from tetramethylsilane, using the residual solvent resonance (^{1}H-NMR: δ_H = 7.26 ppm ($CDCl_3$) and in ^{13}C-NMR: δ_C = 77.16 ppm ($CDCl_3$) or ^{1}H-NMR: δ_H = 2.05 ppm ($(CD_3)_2CO$) and in ^{13}C-NMR: δ_C = 29.84 ppm ($(CD_3)_2CO$) as internal references). The multiplicity is abbreviated as follows: s (singlet), d (doublet), t (triplet), q (quartet), p (pentuplet), m (multiplet) and br (broad). The ^{13}C carbon shifts for the boronated carbon (C-Bmida) are obscured because of the nuclear quadrupole coupling with the boron atom. Coupling constants (*J*) are given in Hz. High-resolution mass spectra (HRMS) were recorded with a Bruker microTOF ESI-TOF mass spectrometer in positive ion mode unless otherwise specified.

Experimental procedures and spectroscopic data

Procedure for the synthesis of alkenyl MIDA boronates 1a, 1d, 1e, 1h and 1j

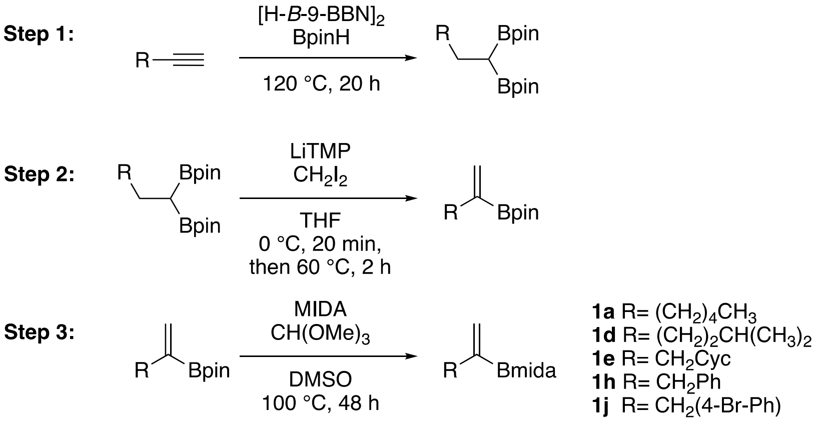

Scheme S1. Synthesis of alkenyl MIDA boronates 1a, 1d, 1e, 1h and 1j.

According to the procedure reported by Thomas and co-workers,[1] alkyne (1.0 equiv., typically 5-10.0 mmol) and $[H\text{-}B\text{-}9\text{-}BBN]_2$ (0.2 equiv.) were reacted in excess pinacolborane (3.0 equiv.) at 120 °C for 20 h (Scheme S1, Step 1). The crude reaction mixture was purified by flash column chromatography (SiO_2, mixtures of hexanes and EtOAc) to obtain pure product.

Step 2 (Scheme S1) was furnished following the reported methodology by Morken and co-workers.[2] To an oven-dried round-bottomed flask equipped with a stir bar was added lithium tetramethylpiperidide (LiTMP, 1.1 equiv.) in glove box. Then, the flask was sealed and brought out of the glovebox. THF (0.7 M) was added to the reaction mixture, and the solution was cooled in an ice bath to 0 °C. Once the solution was cooled, a solution of 1,1-diboronate (1.0 equiv.) in THF (0.5 M) was added dropwise, and the reaction mixture was stirred at 0 °C for 5 min. Subsequently, diiodomethane (2.0 equiv.) in THF (1.0 M) was added dropwise at 0 °C and the mixture was stirred at this temperature for an additional 15 min. Then, the reaction mixture was warmed to 60 °C and stirred for 2 h. After completion, the reaction mixture was cooled to room temperature, diluted with Et_2O and filtered through a pad of silica gel (100% Et_2O). The resulting mixture was concentrated under reduced pressure and purified using column chromatography (SiO_2, mixtures of hexanes and EtOAc).

Step 3 (Scheme S1) was carried out by a modified procedure of Grygorenko and co-workers.[3] In this step the pinacol group of the alkenyl-Bpin compound was exchanged with *N*-methyliminodiacetic acid (MIDA) group. Accordingly, to a Schlenk flask (equipped with a magnetic stirring bar) the alkenyl-Bpin (1.0 equiv.) in anhydrous DMSO (0.25 M) was added. Then, *N*-methyliminodiacetic acid (2.0 equiv.) and $CH(OMe)_3$ (3.0 equiv.) were added to the solution. The resulting mixture was stirred at 100 °C for 48 h. After cooling to room

temperature, it was diluted with EtOAc and H_2O. The organic phase was seperated and the aqueous layer was extracted with EtOAc for three times. The combined organic layers were dried over anhydrous $MgSO_4$ and concentrated under reduced pressure. The resulting crude product was purified by flash chromatography (SiO_2, 100% EtOAc) to afford the pure alkenyl MIDA boronate.

2-(Hept-1-en-2-yl)-6-methyl-1,3,6,2-dioxazaborocane-4,8-dione (1a)

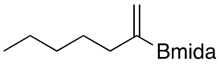

1a

Title compound **1a** was obtained as a white solid (0.95 g, 75% yield). ^{1}**H NMR** (400 MHz, $(CD_3)_2CO$): δ = 5.46 (s, 1H), 5.37 (d, J = 3.3 Hz, 1H), 4.22 (d, J = 17.0 Hz, 2H), 4.04 (d, J = 17.0 Hz, 2H), 2.98 (s, 3H), 2.11 – 2.05 (m, 2H), 1.49 (p, J = 7.3 Hz, 2H), 1.39 – 1.25 (m, 4H), 0.88 (t, J = 6.7 Hz, 3H) ppm. ^{13}**C NMR** (100 MHz, $(CD_3)_2CO$): δ = 169.2, 122.9, 62.6, 47.3, 35.4, 32.6, 29.3, 23.3, 14.4 ppm.

Melting point: 119-120 °C

Spectroscopic data is in agreement with the literature values.[4]

6-Methyl-2-(5-methylhex-1-en-2-yl)-1,3,6,2-dioxazaborocane-4,8-dione (1d)

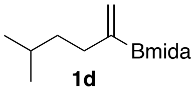

1d

Title compound **1d** was obtained as a white solid (1.3 g, 77% yield). ^{1}**H NMR** (400 MHz, $(CD_3)_2CO$): δ = 5.51 – 5.41 (m, 1H), 5.37 (d, J = 3.2 Hz, 1H), 4.22 (d, J = 16.9 Hz, 2H), 4.04 (d, J = 17.0 Hz, 2H), 2.98 (s, 3H), 2.11 – 2.07 (m, 2H), 1.56 (dp, J = 13.2, 6.6 Hz, 1H), 1.42 – 1.33 (m, 2H), 0.89 (d, J = 6.6 Hz, 6H) ppm; ^{13}**C NMR** (100 MHz, $(CD_3)_2CO$): δ = 169.2, 122.9, 62.6, 47.3, 39.1, 33.2, 28.7, 23.0 ppm.

HRMS (ESI): m/z calcd. for $C_{12}H_{20}BNO_4+Na^+$: 276.1378 $[M+Na]^+$; found: 276.1380.

Melting point: 135-136 °C

2-(3-Cyclohexylprop-1-en-2-yl)-6-methyl-1,3,6,2-dioxazaborocane-4,8-dione (1e)

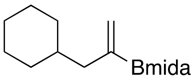

1e

Title compound **1e** was obtained as a white solid (0.85 g, 76% yield). ^{1}**H NMR** (400 MHz, $(CD_3)_2CO$): δ = 5.42 (s, 2H), 4.21 (d, J = 16.9 Hz, 2H), 4.03 (d, J = 16.9 Hz, 2H), 2.98 (s, 3H), 1.96 (dt, J = 6.9, 1.1 Hz, 2H), 1.80 – 1.72 (m, 2H), 1.72 – 1.63 (m, 3H), 1.62 – 1.49 (m, 1H), 1.31 – 1.12 (m, 3H), 0.92 – 0.78 (m, 2H) ppm; ^{13}**C NMR** (100 MHz, $(CD_3)_2CO$): δ = 169.1, 124.2, 62.6, 47.3, 44.0, 37.3, 34.2, 27.4, 27.1 ppm.

HRMS (ESI): m/z calcd. for $C_{14}H_{22}BNO_4+Na^+$: 302.1534 $[M+Na]^+$; found: 302.1537.

Melting point: 178-179 °C

6-Methyl-2-(3-phenylprop-1-en-2-yl)-1,3,6,2-dioxazaborocane-4,8-dione (1h)

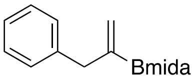

1h

Title compound **1h** was obtained as a white solid (0.56 g, 76% yield). ^{1}**H NMR** (400 MHz, $(CD_3)_2CO$): δ = 7.40 – 7.07 (m, 5H), 5.45 (d, J = 3.1 Hz, 1H), 5.29 (s, 1H), 4.18 (d, J = 16.9 Hz, 2H), 3.85 (d, J = 16.9 Hz, 2H), 3.43

(s, 2H), 2.91 (s, 3H) ppm; ^{13}C **NMR** (100 MHz, $(CD_3)_2CO$): δ = 169.1, 141.2, 130.5, 129.1, 126.8, 125.3, 62.7, 47.4, 42.2 ppm.

HRMS (ESI): m/z calcd. for $C_{14}H_{16}BNO_4+Na^+$: 296.1065 [M+Na]$^+$; found: 296.1067.

Melting point: 132-133 °C

2-(3-(4-Bromophenyl)prop-1-en-2-yl)-6-methyl-1,3,6,2-dioxazaborocane-4,8-dione (1j)

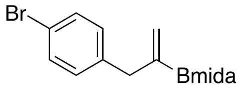

1j

Title compound **1j** was obtained as a white solid (80 mg, 43% yield). ^{1}**H NMR** (400 MHz, $(CD_3)_2CO$): δ = 7.45 (d, J = 8.4 Hz, 2H), 7.16 (d, J = 8.4 Hz, 2H), 5.47 (dd, J = 2.8, 1.4 Hz, 1H), 5.25 (s, 1H), 4.22 (d, J = 17.0 Hz, 2H), 3.97 (d, J = 17.0 Hz, 2H), 3.41 (s, 2H), 2.99 (s, 3H) ppm; ^{13}**C NMR** (100 MHz, $(CD_3)_2CO$): δ = 169.0, 140.8, 132.5, 132.0, 125.6, 120.0, 62.7, 47.4, 41.2 ppm.

HRMS (ESI): m/z calcd. for $C_{14}H_{15}BBrNO_4+Na^+$: 374.0170 [M+Na]$^+$; found: 374.0172.

Melting point: 158-159 °C

Procedure for the synthesis of 6-methyl-2-(non-1-en-2-yl)-1,3,6,2-dioxaza-borocane-4,8-dione (1b)

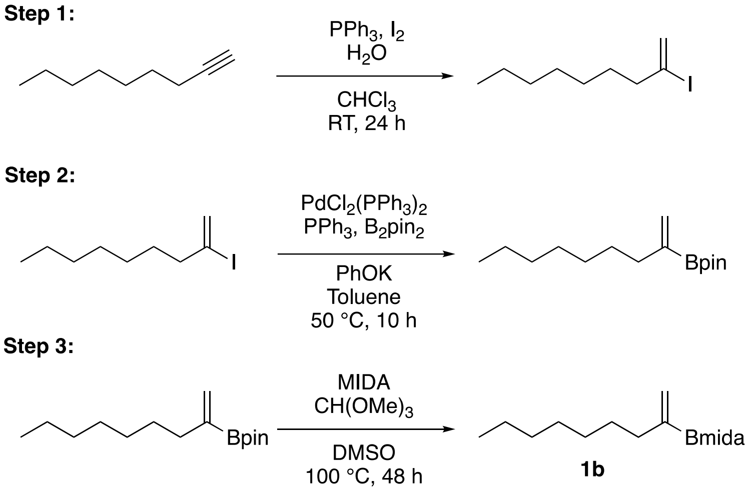

Scheme S2. Synthesis of 6-methyl-2-(non-1-en-2-yl)-1,3,6,2-dioxazaborocane-4,8-dione (1b).

The 2-iodonon-1-ene was synthetized following a reported procedure by Ogawa and co-workers (Scheme S2, Step1).[5] Under argon atmosphere, at room temperature PPh_3 (2.09 g, 8.0 mmol, 1 equiv.), I_2 (2.03 g, 8.0 mmol, 1 equiv.), and H_2O (0.14 mL, 1.0 mmol, 1 equiv.) were placed in a Schlenk flask, followed by $CHCl_3$ (16 mL). Then, 1-Nonyne (0.99 g, 8.0 mmol, 1.0 equiv.) was added to the reaction mixture and the mixture was slowly stirred for 24 h at room temperature, followed by quenching with MeOH (8 mL). The solvent and MeI were then removed under reduced pressure. The crude product was used in the next reaction without further purification.

Next (Scheme S2, Step 2), alkenyl boronic acid pinacol ester was synthetized following a methodology reported by Aggarwal and co-workers.[6] To a Schlenk flask (equipped with a magnetic stir bar, a septum inlet, and a condenser) was added $PdCl_2(PPh_3)_2$ (126 mg, 0.18 mmol, 0.03 equiv.), Ph_3P (96 mg, 0.36 mmol, 0.06 equiv.), bis(pinacolato)diboron (1.67 g, 6.6 mmol, 1.1 equiv.), and PhOK (fine powder, 1.19 g, 9.0 mmol, 1.5 equiv.). The flask was flushed with argon and then charged with toluene (36 mL) and 2-iodonon-1-ene (1.51 g, 6.0 mmol, 1.0 equiv.). The reaction mixture was then stirred at 50 °C for 10 h before cooling to room temperature and treating with H_2O (20 mL). Then the reaction mixture was extracted with Et_2O (3 × 20 mL), washed with brine (20 mL), dried ($MgSO_4$), filtered, and concentrated under reduced pressure. The residue was purified by flash column chromatography (SiO_2, 5% Et_2O/pentane) to give the alkenyl boronic acid pinacol as a colourless liquid.

The final alkenyl MIDA boronate was synthetized following a modified procedure reported by Grygorenko and co-workers (Scheme S2, Step 3).[3] To a Schlenk flask (equipped with a magnetic stirring bar) the alkenyl boronic acid pinacol ester (1.00 g, 4.0 mmol, 1.0

equiv.) in anhydrous DMSO (16 mL), *N*-methyliminodiacetic acid (1.18 g, 8.0 mmol, 2.0 equiv.) and $CH(OMe)_3$ (1.27 g, 12.0 mmol, 3.0 equiv.) were added. The resulting mixture was stirred at 100 °C for 48 h. After cooling to room temperature, it was diluted with EtOAc (30 mL) and H_2O (20 mL). The organic phase was seperated and the aqueous layer was extracted with EtOAc (30 mL) for three times. The combined organic layers were dried over anhydrous $MgSO_4$ and concentrated under reduced pressure. The crude product was purified via flash chromatography (SiO_2, 100% EtOAc) to obtain the desired alkenyl MIDA boronate **1c** as a white solid (0.79 g, 70% yield). **^{1}H NMR** (400 MHz, $CDCl_3$): δ = 5.55 (s, 1H), 5.45 (s, 1H), 3.84 (d, J = 16.4 Hz, 2H), 3.71 (d, J = 16.4 Hz, 2H), 2.82 (s, 3H), 2.00 (m, 2H), 1.48 (m, 2H), 1.28 (m, 8H), 0.88 (m, 3H) ppm; **^{13}C NMR** (100 MHz, $CDCl_3$): δ = 167.3, 124.4, 61.9, 46.7, 34.9, 32.0, 29.7, 29.4, 28.9, 22.8, 14.3 ppm.

HRMS (ESI): m/z calcd. for $C_{14}H_{24}BNO_4+Na^+$: 304.1691 $[M+Na]^+$; found: 304.1693.

Melting point: 120-121 °C

Procedure for the synthesis of 6-methyl-2-(pent-1-en-2-yl)-1,3,6,2-dioxazaborocane-4,8-dione (1c)

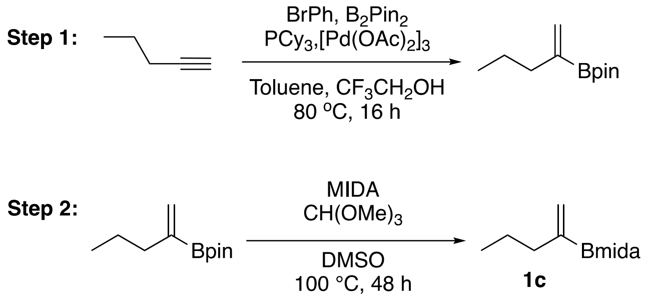

Scheme S3. Synthesis of 6-methyl-2-(pent-1-en-2-yl)-1,3,6,2-dioxazaborocane-4,8-dione (1c).

Step 1 (Scheme S3) is based on the procedure reported by Prabhu and co-workers.[7] To a Schlenk flask (equipped with a magnetic stirring bar) were added sequentially bromobenzene (0.78 g, 5.0 mmol, 1.0 equiv.), bis(pinacolato)diboron (1.40 g, 5.5 mmol, 1.1 equiv.), PCy_3 (0.14 g, 0.5 mmol, 0.1 equiv.), $[Pd(OAc)_2]_3$ (0.17 g, 0.25 mmol, 0.05 equiv.), 1-pentyne (0.34 g, 5.0 mmol, 1.0 equiv.), toluene (10 mL) and trifluoroethanol (1.00 g, 10.0 mmol, 2.0 equiv.) under argon and this mixture was stirred at 80 °C for 16 h. Upon completion of the reaction, the resulting mixture was filtered through a short column of silica gel (eluted with Et_2O) and concentrated. This crude mixture was further purified on a silica gel column chromatography (SiO_2, 4:1 pentane/EtOAc) to obtain the desired alkenyl boronic acid pinacol as a colorless oil.

Step 2 in Scheme S3 was achieved following the modified Grygorenko's procedure.[3] In a Schlenk flask (equipped with a magnetic stirring bar) the alkenyl boronic acid pinacol ester (0.88 g, 4.5 mmol, 1.0 equiv.) was dissolved in anhydrous DMSO (18 mL). Then, *N*-methyliminodiacetic acid (1.32 g, 9.0 mmol, 2.0 equiv.) and $CH(OMe)_3$ (1.43 g, 13.5 mmol, 3.0 equiv.) were added. The resulting mixture was stirred at 100 °C for 48 h. After cooling to room temperature, the reaction mixture was diluted with EtOAc (30 mL) and H_2O (20 mL). The organic phase was seperated and the aqueous layer was extracted with EtOAc (30 mL) for three times. The combined organic layers were dried over anhydrous $MgSO_4$ and concentrated under reduced pressure. The crude product was purified by flash chromatography (SiO_2, 100% EtOAc) to obtain the desired alkenyl MIDA **1c** as a white solid (0.83 g, 82% yield). **^{1}H NMR** (400 MHz, $CDCl_3$): δ = 5.54 (s, 1H), 5.44 (s, 1H), 3.93 (d, J = 16.5 Hz, 2H), 3.73 (d, J = 16. 5 Hz, 2H), 2.82 (s, 3H), 1.98 (m, 2H), 1.50 (m, 2H), 0.92 (t, J = 7.3 Hz, 3H) ppm; **^{13}C NMR** (100 MHz, $CDCl_3$): δ = 168.8, 124.4, 61.9, 46.8, 37.0, 22.0, 14.2 ppm.

HRMS (ESI): m/z calcd. for $C_{10}H_{16}BNO_4+Na^+$: 248.1065 $[M+Na]^+$; found: 248.1066.

Melting point: 108-109 °C

Procedure for the synthesis of alkenyl MIDA boronates 1f and 1l

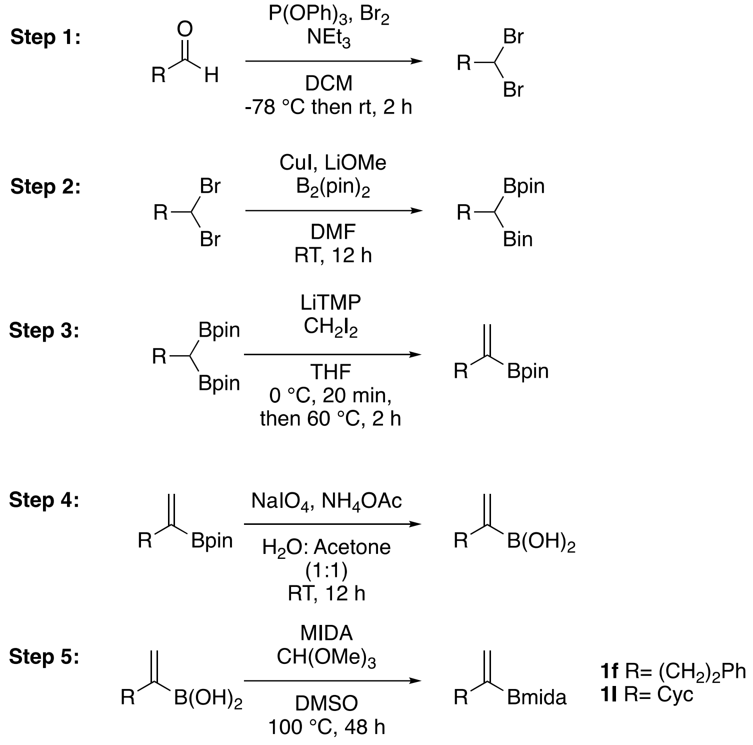

Scheme S4. Synthesis of alkenyl MIDA boronates 1f and 1l.

Alkenyl boronic acid pinacol esters were synthetized following the methodology reported by Morken and co-workers.[2,8] Step 1 (Scheme S4), to a stirred solution of triphenyl phosphite (1.5 equiv) in anhydrous DCM (1.0 M) at -78 °C under argon atmosphere was added bromine (1.3 equiv) dropwise. Then, triethylamine (3.0 equiv) and the corresponding aldehyde (1.0 equiv, 20 mmol) were added at -78 °C. The reaction mixture was warmed to room temperature and stirred for 2 h. Upon completion, the solvent was evaporated in vacuo and the crude reaction mixture was purified by silica chromatography using pentane as eluent to afford the 1,1-dibromide.

Step 2 (Scheme S4), in the glovebox, an oven-dried round-bottom flask with a magnetic stirbar was charged with CuI (0.1 equiv), LiOMe (2.5 equiv) and $B_2(pin)_2$ (1.9 equiv). The flask was sealed with a rubber septum, removed from the glovebox, and DMF (0.5 M) was added under argon atmosphere. After stirring at room temperature for 10 min, a solution of 1,1-dibromide (1.00 equiv) in DMF was added via syringe at room temperature. The reaction stirred at room temperature for 12 h. Upon completion, H_2O was added, and extracted with hexane for three times. The combined organic layers were dried over $MgSO_4$, filtered, and concentrated under reduced pressure. The 1,1-diboronate products were isolated after column chromatography (SiO_2, mixtures of hexanes and EtOAc).

Step 3 (Scheme S4), in the glovebox, to an oven-dried round-bottomed flask equipped with a stir bar was added lithium tetramethylpiperidide (LiTMP, 1.1 equiv.), the flask was sealed and brought out of the glovebox. To the reaction flask was added THF (0.7 M), and the solution was cooled in an ice bath to 0 °C. Once the solution was cooled, a solution of 1,1-diboronate (1.0 equiv.) in THF (0.5 M) was added dropwise, and the reaction mixture was allowed to stir at 0°C for 5 minutes. Next, diiodomethane (2.0 equiv.) in THF (1.0 M) was added dropwise at 0 °C and the mixture was allowed to stir at this temperature for an additional 15 min. Then, the reaction mixture warmed to 60 °C and stirred for 2 h. After completion, the reaction mixture was cooled to room temperature, diluted with Et_2O and filtered through a pad of silica gel (100% Et_2O). The resulting mixture was concentrated under reduced pressure and purified using column chromatography (SiO_2, mixtures of hexanes and EtOAc).

Step 4 (Scheme S4), the deprotection of the alkenyl boronic acid pinacol was carried out following a procedure reported by Anderson and co-workers.[9] A flask was charged with alkenyl boronic acid pinacol ester (1.0 equiv.), $NaIO_4$ (3.6 equiv.), and NH_4OAc (3.6 equiv.). This mixture of reagents was then diluted with a mixture of acetone and water in a 1:1 ratio to form a 0.63 M solution of the alkenyl boronic acid pinacol ester and the resulting slurry stirred vigorously for 12 h. Then the slurry was filtered and the acetone was removed from the filtrate under reduced pressure. The aqueous solution was extracted with Et_2O for three times. The combined organic layers were washed with H_2O and brine, dried with $MgSO_4$, and concentrated under reduced pressure. The crude product was used in the next reaction without further purification.

Finally (Scheme S4, Step 5), the boronic acid was protected with *N*-methyliminodiacetic acid (MIDA), following the modified procedure reported by Grygorenko and co-workers.[3] To a Schlenk flask equipped with a magnetic stirring bar the alkenyl boronic acid pinacol ester (1.0 equiv.) was dissolved in anhydrous DMSO (0.25 M). Then, *N*-methyliminodiacetic acid (2.0 equiv.) and $CH(OMe)_3$ (3.0 equiv.) were added. The resulting mixture was stirred at 100 °C for 48 h. After cooling to room temperature, it was diluted with EtOAc and H_2O. The organic phase was seperated and the aqueous layer was extracted with EtOAc for three times. The combined organic layers were dried over anhydrous $MgSO_4$ and concentrated under reduced pressure. The resulting crude product was purified by flash chromatography (SiO_2, 100% EtOAc) to afford the pure alkenyl MIDA boronate.

6-Methyl-2-(4-phenylbut-1-en-2-yl)-1,3,6,2-dioxazaborocane-4,8-dione (1f)

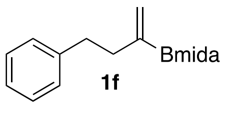

Title compound **1f** was obtained as a white solid (0.7 g, 54% yield). **^{1}H NMR** (400 MHz, $(CD_3)_2CO$): δ = 7.37 – 6.99 (m, 5H), 5.55 (s, 1H), 5.44 (d, J = 3.0 Hz, 1H), 4.24 (d, J = 17.0 Hz, 2H), 4.06 (d, J = 17.0 Hz, 2H),

2.99 (s, 3H), 2.85 – 2.69 (m, 2H), 2.49 – 2.21 (m, 2H) ppm; **^{13}C NMR** (100 MHz, $(CD_3)_2CO$): δ = 169.2, 143.6, 129.3, 129.1, 126.4, 123.6, 62.7, 47.3, 37.9, 36.2 ppm.

HRMS (ESI): m/z calcd. for $C_{15}H_{18}BNO_4$+Na^+: 310.1221 [*M*+Na]$^+$; found: 310.1224.

Melting point: 148-149 °C

2-(1-Cyclohexylvinyl)-6-methyl-1,3,6,2-dioxazaborocane-4,8-dione (1l)

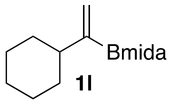

Title compound **1l** was obtained as a white solid (0.68 g, 64% yield). **^{1}H NMR** (400 MHz, $(CD_3)_2CO$): δ = 5.49 (s, 1H), 5.35 (d, *J* = 2.7 Hz, 1H), 4.21 (d, *J* = 17.0 Hz, 2H), 4.03 (d, *J* = 17.0 Hz, 2H), 2.97 (s, 3H), 2.00 – 1.92 (m, 1H), 1.85 – 1.58 (m, 5H), 1.36 – 1.09 (m, 5H) ppm; **^{13}C NMR** (100 MHz, $(CD_3)_2CO$): δ = 169.2, 121.3, 62.7, 47.5, 42.7, 34.4, 27.8, 27.1 ppm.

HRMS (ESI): m/z calcd. for $C_{13}H_{20}BNO_4$+Na^+: 288.1378 [*M*+Na]$^+$; found: 288.1380.

Melting point: 189-190°C

Procedure for the synthesis of 2-(5-chloropent-1-en-2-yl)-6-methyl-1,3,6,2-dioxazaborocane-4,8-dione (1g)

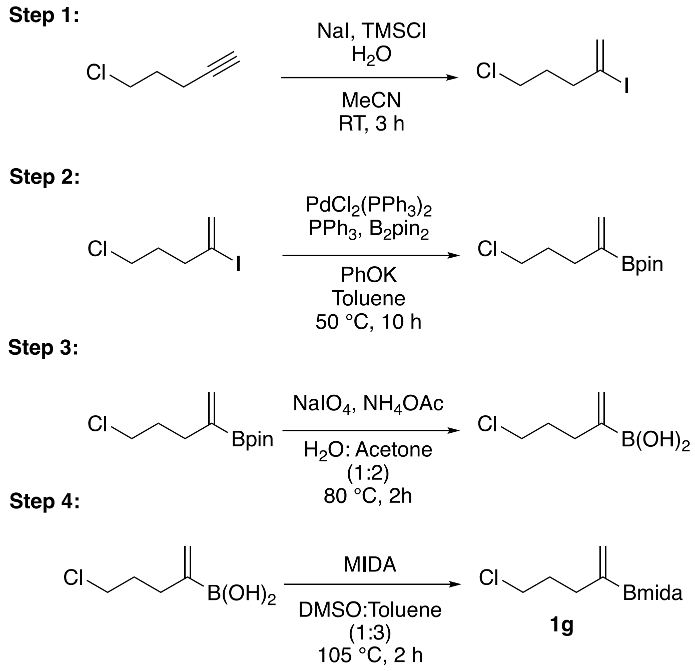

Scheme S5. Synthesis of 2-(5-chloropent-1-en-2-yl)-6-methyl-1,3,6,2-dioxazaborocane-4,8-dione (1g).

The alkenyl boronic acid was synthetized following a reported procedure by Aggarwal and co-workers.[6] Step 1 (Scheme S5), to a solution of NaI (3.00 g, 20.0 mmol, 2.0 equiv.) in MeCN (16 mL) at room temperature was added TMSCl (2.20 g, 20.0 mmol, 2.0 equiv.) followed by H_2O (0.18 mL, 10.0 mmol, 1.0 equiv.), and the cloudy solution was stirred for 10 min. 5-Chloro-1-pentyne (1.30 g, 10.0 mmol, 1.0 equiv.) in MeCN (4 mL) was then added dropwise and the solution stirred for an additional 3 h. The reaction was quenched with H_2O (15 mL) and extracted into Et_2O (3 × 20 mL). The organic extracts were combined and washed with 5% aqueous NaOH (20 mL), brine (25 mL), dried ($MgSO_4$), filtered, and concentrated under reduced pressure. The residue was then purified by flash column chromatography (5% EtOAc/hexane) to give 5-chloro-2-iodopent-1-ene as a yellow oil.

Step 2 (Scheme S5), to a Schlenk flask (equipped with a magnetic stir bar, a septum inlet, and a condenser) was subsequently added $PdCl_2(PPh_3)_2$ (126 mg, 0.18 mmol, 0.03 equiv.), Ph_3P (96 mg, 0.36 mmol, 0.06 equiv.), bis(pinacolato)diboron (1.67 g, 6.6 mmol, 1.1 equiv.), and PhOK (fine powder, 1.19 g, 9.0 mmol, 1.5 equiv.). The flask was flushed with argon and then charged with toluene (36 mL) and 5-chloro-2-iodopent-1-ene (1.38 g, 6.0 mmol, 1.0 equiv.). The reaction was then stirred at 50 °C for 10 h before cooling to room temperature and treating with H_2O (20 mL). The mixture was extracted into Et_2O (3 × 20 mL), washed with brine (20 mL), dried ($MgSO_4$), filtered, and concentrated under reduced pressure. The residue

was purified by flash column chromatography (5% Et_2O/pentane) to give the alkenyl boronic acid pinacol ester as a colourless liquid.

Step 3 (Scheme S5), deprotection of the pinacol group and subsequent protection with MIDA was carried out following the reported procedure by Perrin and co-workers.[10] To a solution of pinacol ester (0.92 g, 4.0 mmol, 1.0 equiv.) in a 2:1 mixture of acetone and water (0.1 M) were added ammonium acetate (0.96 g, 12.4 mmol, 3.1 equiv.) and sodium periodate (2.65 g, 12.4 mmol, 3.1 equiv.). This suspension was stirred at 80 °C for 2 h. The mixture was then cooled down to room temperature and treated with a 0.5 M aqueous thiosulfate solution. Subsequently, the reaction mixture was extracted with EtOAc until complete extraction confirmed by TLC. The combined organic extracts were washed with brine, dried with $MgSO_4$, filtered and concentrated to afford the corresponding boronic acid.

Step 4 (Scheme S5), the crude residue was resuspended in a 3:1 mixture of toluene and DMSO (0.1 M) and *N*- methyliminiodiacetic acid (1.77 g, 12.0 mmol, 3.0 equiv.) was added. The suspension was stirred at 105 °C for 2 h. The mixture was then cooled down to room temperature and treated with diluted brine (30% brine in H_2O) and the mixture was extracted with EtOAc until complete extraction confirmed by TLC. The combined organic extracts were dried with $MgSO_4$, filtered and concentrated. The crude residue was purified by column chromatography (SiO_2, 100% EtOAc) to afford pure **1g** as a white solid (0.53 g, 51% yield). **^{1}H NMR** (400 MHz, $CDCl_3$): δ = 5.56 (s, 1H), 5.49 (s, 1H), 3.96 (d, *J* = 16.6 Hz, 2H; H-2), 3.75 (d, *J* = 16.6, 2H), 3.58 (t, *J* = 6.3 Hz, 2H), 2.84 (s, 3H), 2.18 (m, 2H), 1.97 (m, 2H) ppm.

Spectroscopic data is in agreement with the literature values.[10]

Procedure for the synthesis of 6-methyl-2-(3-(p-tolyl)prop-1-en-2-yl)-1,3,6,2-dioxazaborocane-4,8-dione (1i)

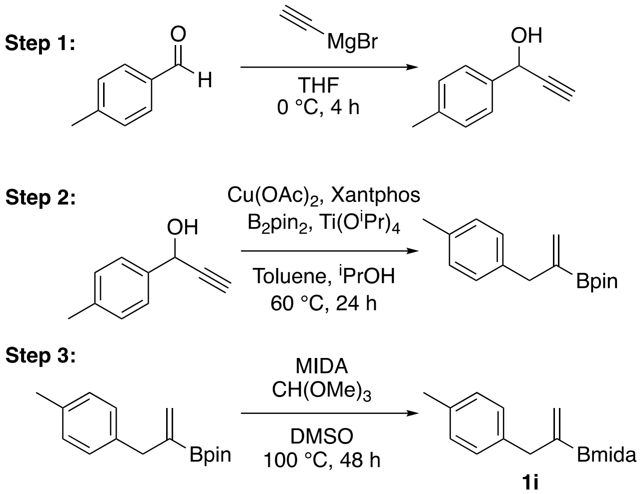

Scheme S6. Synthesis of 6-methyl-2-(3-(p-tolyl)prop-1-en-2-yl)-1,3,6,2-dioxazaborocane-4,8-dione (1i).

Step 1 (Scheme S6) was carried out according to the procedure reported by Gotor-Fernández and co-workers.[11] To a solution of 4-methylbenzaldehyde (0.60 g, 5.0 mmol, 1.0 equiv.) in dry THF (2.5 mL) at 0 °C and under argon atmosphere, a solution (0.5 M) of ethynylmagnesium bromide in dry THF (11 mL, 5.5 mmol, 1.1 equiv.) was added. The reaction mixture was stirred at 0 °C for 4 h and then, the reaction was quenched by the addition of a saturated NH_4Cl aqueous solution (10 mL). The solvent was removed under reduced pressure, and the mixture extracted with CH_2Cl_2 (3 x 10 mL). The combined organic phases were washed with a saturated NaCl aqueous solution (2 x 10 mL), dried over $MgSO_4$, filtered and concentrated under reduced pressure. The reaction crude was purified by column chromatography (SiO_2, hexane/EtOAc = 8/1) to afford 0.46 g (63 %) alcohol product.

Step 2 (Scheme S6) was carried out following the procedure reported by Marder and co-workers.[12] $Cu(OAc)_2$ (0.06 g, 0.32 mmol, 0.1 equiv.) and Xantphos (0.24 g, 0.41 mmol, 0.13 equiv.) were dissolved in 0.5 mL of toluene in a dried vial in the glove-box under argon and the reaction was stirred for 5 min. Then, B_2pin_2 (0.84 g, 3.3 mmol, 1.05 equiv.), 1-(*p*-tolyl)prop-2-yn-1-ol (0.46 g, 3.15 mmol, 1.0 equiv.), $Ti(O^iPr)_4$ (0.89 g, 3.15 mmol, 1.0 equiv.) and ^{i}PrOH (0.28 g, 4.72 mmol, 1.5 equiv.) were added in this order. Finally, another 0.5 mL of toluene was added to the mixture. The reaction was heated at 60 ºC under argon for 24 hours. The crude mixture was filtered through a pad of Celite. Then, the solvent was removed under reduced pressure. The crude product was purified by column chromatography (SiO_2, pentane/Et_2O = 50/1) to afford 0.4 g (49 %) alkenyl boronic acid pinacol product.

Step 3 (Scheme S6) was carried out following the modified Grygorenko's procedure.[3] In a Schlenk flask equipped with a magnetic stirring bar, the alkenyl boronic acid pinacol ester (0.4 g, 1.55 mmol, 1.0 equiv.) was dissolved in anhydrous DMSO (5 mL). Then, *N*-methyliminodiacetic acid (0.46 g, 3.1 mmol, 2.0 equiv.) and $CH(OMe)_3$ (0.49 g, 4.65 mmol, 3.0 equiv.) were added. The resulting mixture was stirred at 100 °C for 48 h and after cooling to room temperature, it was diluted with EtOAc (30 mL) and H_2O (20 mL). The organic phase was seperated and the aqueous layer was extracted with EtOAc (30 mL) for three times. The combined organic layers were dried over anhydrous $MgSO_4$ and concentrated under reduced pressure. The crude product was purified via flash chromatography (SiO_2, 100% EtOAc) to obtain pure **1i** as a white solid (0.21 g, 47% yield). **^{1}H NMR** (400 MHz, $CDCl_3$): δ = 7.09 (s, 4H), 5.73 – 5.63 (m, 1H), 5.50 (d, J = 2.7 Hz, 1H), 3.56 (d, J = 16.2 Hz, 2H), 3.45 (s, 2H), 3.08 (d, J = 16.1 Hz, 2H), 2.48 (s, 3H), 2.31 (s, 3H) ppm; **^{13}C NMR** (100 MHz, $CDCl_3$): δ = 167.3, 136.8, 136.3, 129.6, 129.5, 127.1, 62.2, 46.8, 42.7, 21.2. ppm.

HRMS (ESI): m/z calcd. for $C_{15}H_{18}BNO_4+Na^+$: 310.1221 $[M+Na]^+$; found: 310.1224.
Melting point: 142-143 °C

Procedure for the synthesis of 2-(4-(1,3-dioxoisoindolin-2-yl)but-1-en-2-yl)-6-methyl-1,3,6,2-dioxazaborocane-4,8-dione (1k)

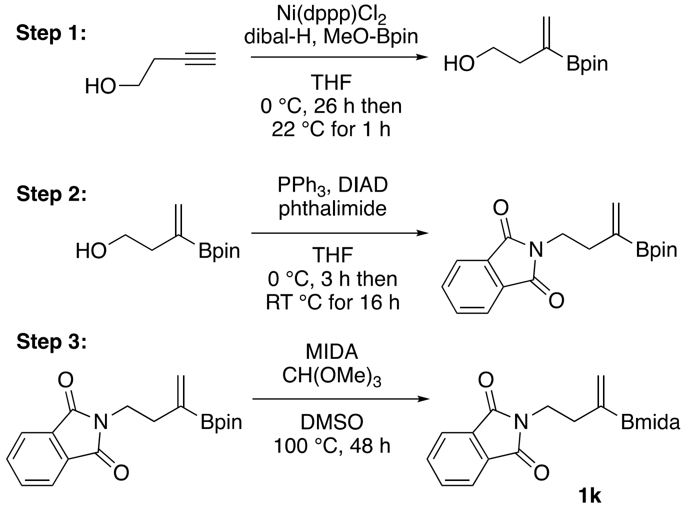

Scheme S7. Synthesis of 2-(4-(1,3-Dioxoisoindolin-2-yl)but-1-en-2-yl)-6-methyl-1,3,6,2-dioxaza-borocane-4,8-dione (1k).

Step 1 (Scheme S7) is based on the reported procedure by Hoveya and co-workers.[13] 1,3-Bis(diphenylphosphino)propane nickel(II) chloride (Ni(dppp)Cl_2, 163 mg, 0.3 mmol, 0.03 equiv.) was placed in an flame-dried round bottom flask equipped with a stir bar. The apparatus was sealed and purged with argon for approximately 10 min. Then, THF (25 mL) was added through a syringe, followed by dropwise addition of dibal-H (3.27 g, 23.0 mmol, 2.3 equiv.) at 22 °C (gas evolution occurs as dibal-H is added). The resulting black solution was cooled to 0 °C before but-3-yn-1-ol (0.70 g, 10.0 mmol, 1.0 equiv.) was added slowly over 5 min (reaction is exothermic). The resulting black solution was allowed to warm to 22 °C and stired for an additional 2 h. After 2 hours, 2-methoxy-4,4,5,5-tetramethyl-1,3,2-dioxaborolane (MeO-Bpin, 4.74 g, 30.0 mmol, 3.0 equiv.) was added dropwise through a syringe into the reaction solution at 0 °C (ice bath). The resulting solution was stirred for 24 h before the reaction was quenched by dropwise addition of H_2O (3.0 mL) at 0 °C (ice bath). The mixture warmed to 22 °C and stirred for one additional hour before it was washed with Et_2O (5.0 mL x 3). The combined organic layers were passed through a plug of anhydrous $MgSO_4$ and concentrated under reduced pressure to afford yellow oil, which was purified by column chromatography (SiO_2, pentane/EtOAc = 5/1) to afford 1.16 g (59%) alkenyl boronic acid pinacol ester product.

Step 2 (Scheme S7), to a Schlenk flask was sequentially added the alkenyl boronic acid pinacol (1.16 g, 5.86 mmol, 1.0 equiv.), PPh_3 (1.84 g, 7.03 mmol, 1.2 equiv.) and phthalimide (0.94 g, 6.44 mmol, 1.1 equiv.). These reagents were dissolved in THF (15 mL). To the reaction mixture at 0 °C, DIAD (7.61 mmol) was added dropwise. The mixture was stirred for 3 h at 0 °C before warming to room temperature overnight. After this time, THF was removed under

reduced pressure, the residue was purified by column chromatography (SiO_2, pentane/EtOAc = 4/1) to afford the phthalimide substituted alkenyl boronic acid pinacol ester product (1 g, 48 % yield).

Step 3 (Scheme S7) was carried out following a modified procedure by Grygorenko and co-workers.[3] In a Schlenk flask (equipped with a magnetic stirring bar) the alkenyl boronic acid pinacol ester (1.00 g, 3.05 mmol, 1.0 equiv.) was dissolved in anhydrous DMSO (12 mL). Then, *N*-methyliminodiacetic acid (0.90 g, 6.1 mmol, 2.0 equiv.) and $CH(OMe)_3$ (0.97 g, 9.15 mmol, 3.0 equiv.) were added. The resulting mixture was stirred at 100 °C for 48 h. After cooling to room temperature, it was diluted with EtOAc (30 mL) and H_2O (20 mL). The organic phase was seperated and the aqueous layer was extracted with EtOAc (30 mL) for three times. The combined organic layers were dried over anhydrous $MgSO_4$ and concentrated under reduced pressure. The crude product was purified via flash chromatography (SiO_2, 100% EtOAc) to obtain the desired alkenyl MIDA boronate **1k** as a white solid (0.8 g, 73% yield). **^{1}H NMR** (400 MHz, $(CD_3)_2CO$): δ = 7.86 – 7.81 (m, 4H), 5.56 (s, 1H), 5.47 (d, *J* = 2.7 Hz, 1H), 4.24 (d, *J* = 16.9 Hz, 2H), 4.07 (d, *J* = 17.0 Hz, 2H), 3.90 – 3.77 (m, 2H), 3.03 (s, 3H), 2.47 (ddd, *J* = 7.7, 6.3, 1.2 Hz, 2H) ppm; **^{13}C NMR** (100 MHz, $(CD_3)_2CO$): δ = 169.0, 168.7, 134.9, 133.2, 125.1, 123.7, 62.6, 47.3, 38.2, 33.7 ppm.

HRMS (ESI): m/z calcd. for $C_{17}H_{17}BN_2O_6+Na^+$: 379.1072 $[M+Na]^+$; found: 379.1075.
Melting point: 80-81 °C

General procedure for fluorination of alkyl vinyl-Bmida derivatives

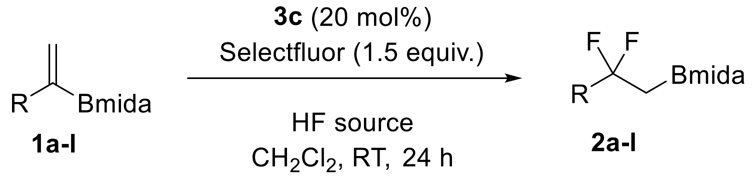

Scheme S8. Aryl iodide-catalyzed bora-Wagner-Meerwein rearrangement.

A 15 mL polypropylene (PP) tube fitted with a PP-screw cap, containing a Teflon magnetic stir bar was charged with the substrate **1** (0.1 mmol, 1.0 equiv.) and 1-iodo-4-methylbenzene **3c** (0.02 mmol, 0.2 equiv.) before the CH_2Cl_2 was added (0.5 mL) as solvent (Scheme S8). Then, the HF source (0.17 mL of pyr·9HF, 6.5 mmol HF, 65 equiv. HF or **A** = 0.10 mL pyr·9HF + 0.15 mL TEA·3HF, 6.5 mmol HF, 65 equiv.) was added by syringe. Finally, Selectfluor (0.15 mmol, 1.5 equiv.) was added in one portion and the cap was screwed on tightly. The reaction mixture was stirred at room temperature. After 24 h, it was quenched by addition of aq. sat. $NaHCO_3$ solution (8 mL) and stirred for 5 min. The biphasic mixture was poured into a phase separator (25 mL, Biotage) and the organic phase was collected in a flask. The PP tube was rinsed twice with CH_2Cl_2 and after collection of the entire organic phase. The solvent was removed under reduced pressure and the crude material was purified on silica gel affording the desired β-difluroalkyl boron compound **2**.

2-(2,2-Difluoroheptyl)-6-methyl-1,3,6,2-dioxazaborocane-4,8-dione (2a)

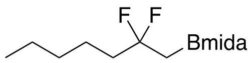

2a

Title compound **2a** was prepared according to the general procedure using HF source **A** and purified by column chromatography (SiO_2; pentane/EtOAc = 1/3 to 100% EtOAc) affording **2a** as a white solid (20 mg, 69% yield). **^{1}H NMR** (400 MHz, $CDCl_3$): δ = 3.93 (d, J = 16.5 Hz, 2H), 3.79 (d, J = 16.5 Hz, 2H), 3.01 (s, 3H), 1.89 (dddd, J = 22.1, 17.0, 9.2, 6.9 Hz, 2H), 1.64 – 1.40 (m, 4H), 1.37 – 1.26 (m, J = 4.8 Hz, 4H), 1.02 – 0.79 (m, 3H) ppm; **^{13}C NMR** (100 MHz, $CDCl_3$): δ = 168.0, 127.4 (t, J = 238.1 Hz), 62.4, 46.4, 38.9 (t, J = 25.9 Hz), 31.6, 24.6 – 23.6 (br), 22.6, 22.5 (t, J = 4.6 Hz), 14.0 ppm; **^{19}F NMR** (377 MHz, $CDCl_3$): δ = -85.30 (p, J = 18.5 Hz, 2F) ppm; **^{11}B NMR** (128 MHz, $CDCl_3$) δ = 11.90 ppm.

HRMS (ESI): m/z calcd. for $C_{12}H_{20}BF_2NO_4$+Na^+: 314.1346 [M+Na]$^+$; found: 314.1348.

Melting point: 106-107 °C

2-(2,2-Difluorononyl)-6-methyl-1,3,6,2-dioxazaborocane-4,8-dione (2b)

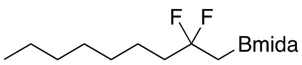

2b

Title compound **2b** was prepared according to the general procedure using HF source **A** and purified by column chromatography (SiO_2; EtOAc (100%)) affording **2b** as a white

solid (15.9 mg, 50% yield). **^{1}H NMR** (400 MHz, $CDCl_3$): δ = 3.80 (m, 4H), 3.02 (s, 3H), 1.90 (m, 2H), 1.57 (t, J = 19.8 Hz, 2H), 1.45 (m, 2H), 1.29 (m, 8H), 0.88 (m, 3H) ppm; **^{13}C NMR** (100 MHz, $CDCl_3$): δ = 166.7, 127.5 (t, J = 237.8 Hz), 62.6, 46.3, 38.7 (t, J = 25.9 Hz), 31.8, 29.4, 29.2, 22.8, 14.2 ppm; **^{19}F NMR** (377 MHz, $CDCl_3$): δ = -84.90 (p, J = 18.6 Hz) ppm; **^{11}B NMR** (128 MHz, $CDCl_3$) δ = 12.25 ppm.

HRMS (ESI): m/z calcd. for $C_{14}H_{24}BF_2NO_4$+Na^+: 342.1659 [M+Na]$^+$; found: 342.1661.

Melting point: 110-111 °C

2-(2,2-Difluoropentyl)-6-methyl-1,3,6,2-dioxazaborocane-4,8-dione (2c)

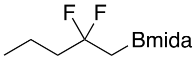

2c

Title compound **2c** was prepared according to the general procedure using HF source **A** and purified by column chromatography (SiO_2; EtOAc (100%)) affording **2c** as a white solid (15.7 mg, 60% yield). **^{1}H NMR** (400 MHz, $CDCl_3$): δ = 3.99 (d, J = 16.5 Hz, 2H), 3.80 (d, J = 16.5 Hz, 2H), 2.99 (s, 3H), 1.87 (m, 2H), 1.49 (m, 4H), 0.95 (t, J = 7.4 Hz, 3H) ppm; **^{13}C NMR** (100 MHz, $CDCl_3$): δ = 167.9, 127.4 (t, J = 238.1 Hz), 62.4, 46.4, 40.9 (t, J = 25.8 Hz), 16.2 (t, J = 5.0 Hz), 14.0 ppm; **^{19}F NMR** (377 MHz, $CDCl_3$): δ = -84.94 (p, J = 18.5 Hz) ppm; **^{11}B NMR** (128 MHz, $CDCl_3$) δ = 11.87 ppm.

HRMS (ESI): m/z calcd. for $C_{10}H_{16}BF_2NO_4$+Na^+: 286.1033 [M+Na]$^+$; found: 286.1034.

Melting point: 81-82 °C

2-(2,2-Difluoro-5-methylhexyl)-6-methyl-1,3,6,2-dioxazaborocane-4,8-dione (2d)

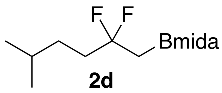

2d

Title compound **2d** was prepared according to the general procedure using HF source **A** and purified by column chromatography (SiO_2; pentane/EtOAc = 1/3 to 100% EtOAc) affording **2d** as a white solid (20 mg, 69% yield). **^{1}H NMR** (400 MHz, $(CD_3)_2CO$): δ = 4.24 (d, J = 16.9 Hz, 2H), 4.03 (d, J = 16.9 Hz, 2H), 3.16 (s, 3H), 2.02 – 1.86 (m, 2H), 1.66 – 1.45 (m, 3H), 1.41 – 1.30 (m, 4H), 0.91 (d, J = 6.6 Hz, 6H) ppm; **^{13}C NMR** (100 MHz, $(CD_3)_2CO$): δ = 168.5, 128.1 (t, J = 238.3 Hz), 62.8, 46.7, 36.8 (t, J = 25.9 Hz), 32.3 (t, J = 4.4 Hz), 28.6, 22.7 ppm; **^{19}F NMR** (377 MHz, $(CD_3)_2CO$): δ = -79.63 (p, J = 17.9 Hz, 2F) ppm; **^{11}B NMR** (128 MHz, $(CD_3)_2CO$) δ = 11.36 ppm.

HRMS (ESI): m/z calcd. for $C_{12}H_{20}BF_2NO_4$+Na^+: 314.1346 [M+Na]$^+$; found: 314.1348.

Melting point: 137-138 °C

2-(3-Cyclohexyl-2,2-difluoropropyl)-6-methyl-1,3,6,2-dioxazaborocane-4,8-dione (2e)

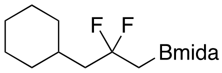

2e

Title compound **2e** was prepared according to the general procedure using HF source **A** and purified by column chromatography (SiO_2; pentane/EtOAc = 1/3 to 100% EtOAc) affording **2e** as a white solid (14.2

mg, 45% yield). **^{1}H NMR** (400 MHz, $(CD_3)_2CO$): δ = 4.24 (d, J = 16.9 Hz, 2H), 4.02 (d, J = 16.9 Hz, 2H), 3.15 (s, 3H), 1.91 – 1.77 (m, 4H), 1.72 – 1.45 (m, 6H), 1.37 – 1.13 (m, 3H), 1.06 – 0.91 (m, 2H) ppm; **^{13}C NMR** (100 MHz, $(CD_3)_2CO$): δ = 168.4, 128.3 (t, J = 239.0 Hz), 62.8, 46.8 (t, J = 1.7 Hz), 46.0 (t, J = 24.7 Hz), 34.7, 33.5 (t, J = 3.3 Hz), 26.9 ppm; **^{19}F NMR** (377 MHz, $(CD_3)_2CO$): δ = -82.00 (p, J = 18.6 Hz, 2F) ppm; **^{11}B NMR** (128 MHz, $(CD_3)_2CO$) δ = 11.37 ppm.

HRMS (ESI): m/z calcd. for $C_{14}H_{22}BF_2NO_4+Na^+$: 340.1502 $[M+Na]^+$; found: 340.1505.

Melting point: 165-166 °C

2-(2,2-Difluoro-4-phenylbutyl)-6-methyl-1,3,6,2-dioxazaborocane-4,8-dione (2f)

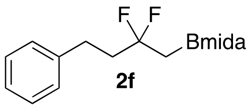

2f

Title compound **2f** was prepared according to the general procedure using HF source **A** and purified by column chromatography (SiO_2; pentane/EtOAc = 1/3 to 100% EtOAc) affording **2f** as a white solid (18.5 mg, 57% yield). **^{1}H NMR** (400 MHz, $(CD_3)_2CO$): δ = 7.37 – 7.13 (m, 5H), 4.26 (d, J = 16.9 Hz, 2H), 4.06 (d, J = 16.9 Hz, 2H), 3.18 (s, 3H), 2.83 – 2.73 (m, 2H), 2.34 – 2.18 (m, 2H), 1.60 (t, J = 18.9 Hz, 2H) ppm; **^{13}C NMR** (100 MHz, $(CD_3)_2CO$): δ = 168.5, 142.1, 129.3, 129.2, 127.4 (t, J = 237.6 Hz), 126.8, 62.8, 46.7, 41.0 (t, J = 26.0 Hz) ppm; **^{19}F NMR** (377 MHz, $(CD_3)_2CO$): δ = -86.07 (p, J = 18.5 Hz, 2F) ppm; **^{11}B NMR** (128 MHz, $(CD_3)_2CO$) δ = 11.4 ppm.

HRMS (ESI): m/z calcd. for $C_{15}H_{18}BF_2NO_4+Na^+$: 348.1189 $[M+Na]^+$; found: 348.1192.

Melting point: 84-85 °C

2-(5-Chloro-2,2-difluoropentyl)-6-methyl-1,3,6,2-dioxazaborocane-4,8-dione (2g)

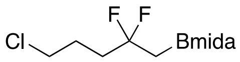

2g

Title compound **2g** was prepared according to the general procedure using HF source **A** and purified by column chromatography (SiO_2; EtOAc (100%)) affording **2g** as a white solid (12.7 mg, 45% yield). **^{1}H NMR** (400 MHz, $CDCl_3$): δ = 3.89 (d, J = 16.4 Hz, 2H), 3.81 (d, J = 16.4 Hz, 2H), 3.60 (t, J = 6.4 Hz, 2H), 3.05 (s, 3H), 2.09 (m, 4H), 1.62 (m, 2H) ppm; **^{13}C NMR** (100 MHz, $CDCl_3$): δ = 166.5, 126.8 (t, J = 238.1 Hz), 62.6, 46.4, 44.4, 36.0 (t, J = 26.3 Hz), 25.9 (t, J = 4.6 Hz) ppm; **^{19}F NMR** (377 MHz, $CDCl_3$): δ = 85.80 (p, J = 18.5 Hz) ppm; **^{11}B NMR** (128 MHz, $CDCl_3$) δ = 11.55 ppm.

HRMS (ESI): m/z calcd. for $C_{10}H_{15}BClF_2NO_4+Na^+$: 234.0537 $[M+Na]^+$; found: 234.0539.

Melting point: 162-163 °C

2-(2,2-Difluoro-3-phenylpropyl)-6-methyl-1,3,6,2-dioxazaborocane-4,8-dione (2h)

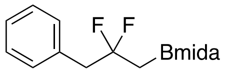

2h

Title compound **2h** was prepared according to the general procedure using pyr·9HF and purified by column chromatography (SiO_2; EtOAc (100%)) affording **2h** as a white solid (25.1 mg, 86% yield). **^{1}H NMR** (400 MHz, $CDCl_3$): δ = 7.28 (m, 5H), 3.89 (d, J = 16.5 Hz, 2H), 3.74 (d, J = 16.5 Hz, 2H), 3.21 (t, J = 16.7 Hz, 2H), 2.85 (s, 3H), 1.49 (t, J = 19.9 Hz, 2H) ppm; **^{13}C NMR** (100 MHz, $CDCl_3$): δ = 166.9, 133.6 (t, J = 4.6 Hz), 130.6, 128.5, 127.4, 126.3, 123.9, 62.4, 46.2, 44.6 (t, J = 26.7 Hz) ppm; **^{19}F NMR** (377 MHz, $CDCl_3$): δ = -83.92 (p, J = 18.2 Hz, 2F) ppm; **^{11}B NMR** (128 MHz, $CDCl_3$) δ = 12.06 ppm.

HRMS (ESI): m/z calcd. for $C_{14}H_{16}BF_2NO_4$+Na^+: 334.1033 [M+Na]$^+$; found: 334.1035.

Melting point: 126-127 °C

2-(2,2-Difluoro-3-(p-tolyl)propyl)-6-methyl-1,3,6,2-dioxazaborocane-4,8-dione (2i)

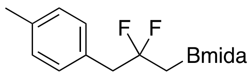

2i

Title compound **2i** was prepared according to the general procedure using pyr·9HF and purified by column chromatography (SiO_2; pentane/EtOAc = 1/3 to 100% EtOAc) affording **2i** as a white solid (24.4 mg, 75% yield). **^{1}H NMR** (400 MHz, $(CD_3)_2CO$): δ = 7.19 (d, J = 7.8 Hz, 2H), 7.15 – 7.10 (m, 2H), 4.27 (d, J = 17.0 Hz, 2H), 4.02 (d, J = 16.9 Hz, 2H), 3.22 (t, J = 16.7 Hz, 2H), 3.11 (s, 3H), 2.30 (s, 3H), 1.48 (t, J = 19.1 Hz, 2H) ppm; **^{13}C NMR** (100 MHz, $(CD_3)_2CO$): δ = 168.5, 137.2, 132.3 (t, J = 4.4 Hz), 127.0 (t, J = 238.3 Hz), 131.4, 129.6, 62.9, 46.9 (t, J = 1.7 Hz), 44.4 (t, J = 26.6 Hz), 21.06 ppm; **^{19}F NMR** (377 MHz, $(CD_3)_2CO$): δ = -84.38 (p, J = 18.0 Hz, 2F) ppm; **^{11}B NMR** (128 MHz, $(CD_3)_2CO$) δ = 11.36 ppm.

HRMS (ESI): m/z calcd. for $C_{15}H_{18}BF_2NO_4$+Na^+: 348.1189 [M+Na]$^+$; found: 348.1192.

Melting point: 160-161 °C

2-(3-(4-Bromophenyl)-2,2-difluoropropyl)-6-methyl-1,3,6,2-dioxazaborocane-4,8-dione (2j)

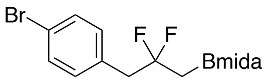

2j

Title compound **2j** was prepared according to the general procedure using pyr·9HF and purified by column chromatography (SiO_2; pentane/EtOAc = 1/3 to 100% EtOAc) affording **2j** as a white solid (15.6 mg, 40% yield). **^{1}H NMR** (400 MHz, $(CD_3)_2CO$): δ = 7.58 – 7.43 (m, 2H), 7.27 (d, J = 8.1 Hz, 2H), 4.28 (d, J = 17.0 Hz, 2H), 4.04 (d, J = 16.9 Hz, 2H), 3.27 (t, J = 16.7 Hz, 2H), 3.13 (s, 3H), 1.50 (t, J = 18.9 Hz, 2H) ppm; **^{13}C NMR** (100 MHz, $(CD_3)_2CO$): δ = 168.5, 134.7 (t, J = 4.2 Hz), 133.6, 132.0, 126.6 (t, J = 240.1 Hz), 121.5, 62.9, 46.9, 44.1 (t, J = 26.7 Hz) ppm; **^{19}F NMR** (377 MHz, $(CD_3)_2CO$): δ = -84.57 (p, J = 18.0 Hz, 2F) ppm; **^{11}B NMR** (128 MHz, $(CD_3)_2CO$) δ = 11.26 ppm.

HRMS (ESI): m/z calcd. for $C_{14}H_{15}BBrF_2NO_4$+Na^+: 412.0138 [M+Na]$^+$; found: 412.0140.

Melting point: 146-147 °C

2-(4-(1,3-Dioxoisoindolin-2-yl)-2,2-difluorobutyl)-6-methyl-1,3,6,2-dioxazaborocane-4,8-dione (2k)

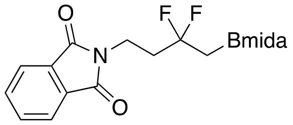

Title compound **2k** was prepared according to the general procedure using pyr·9HF and purified by column chromatography (SiO_2; EtOAc (100%)) affording **2k** as a white solid (25 mg, 63% yield). ^{1}**H NMR** (400 MHz, $(CD_3)_2CO$): δ = 8.10 – 7.72 (m, 4H), 4.25 (d, J = 16.9 Hz, 2H), 4.05 (d, J = 16.9 Hz, 2H), 3.96 – 3.84 (m, 2H), 3.17 (s, 3H), 2.40 (tt, J = 16.7, 7.3 Hz, 2H), 1.63 (t, J = 19.4 Hz, 2H) ppm; ^{13}**C NMR** (100 MHz, $(CD_3)_2CO$): δ = 168.5, 168.4, 135.0, 133.2, 127 (t, J = 239.2 Hz), 123.7, 62.8, 46.8, 37.0 (t, J = 25.7 Hz), 32.78 (t, J = 6.1 Hz) ppm; ^{19}**F NMR** (377 MHz, $(CD_3)_2CO$): δ = -86.01 (p, J = 18.0 Hz, 2F) ppm; ^{11}**B NMR** (128 MHz, $(CD_3)_2CO$) δ = 11.28 ppm.

HRMS (ESI): m/z calcd. for $C_{17}H_{17}BF_2N_2O_6$+Na^+: 417.1040 [M+Na]$^+$; found: 417.1043.

Melting point: 175-176 °C

2-(2-Cyclohexyl-2,2-difluoroethyl)-6-methyl-1,3,6,2-dioxazaborocane-4,8-dione (2l)

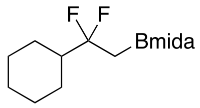

Title compound **2l** was prepared according to the general procedure using pyr·9HF and purified by column chromatography (SiO_2; pentane/EtOAc = 1/3 to 100% EtOAc) affording **2l** as a white solid (17 mg, 56% yield). ^{1}**H NMR** (400 MHz, $(CD_3)_2CO$): δ = 4.23 (d, J = 16.9 Hz, 2H), 4.02 (d, J = 16.9 Hz, 2H), 3.15 (s, 3H), 1.91 – 1.74 (m, 5H), 1.71 – 1.61 (m, 1H), 1.47 (t, J = 20.8 Hz, 2H), 1.33 – 1.08 (m, 5H) ppm; ^{13}**C NMR** (100 MHz, $(CD_3)_2CO$): δ = 168.5, 128.7 (t, J = 240.7 Hz), 62.8, 46.7, 46.2 (t, J = 24.6 Hz), 26.8 (t, J = 4.5 Hz), 26.78, 26.4 ppm; ^{19}**F NMR** (377 MHz, $(CD_3)_2CO$): δ = -93.69 (td, J = 20.9, 13.5 Hz, 2F) ppm; ^{11}**B NMR** (128 MHz, $(CD_3)_2CO$) δ = 16.82 ppm.

HRMS (ESI): m/z calcd. for $C_{13}H_{20}BF_2NO_4$+Na^+: 326.1346 [M+Na]$^+$; found: 326.1348.

Melting point: 188-189 °C

Control experiments for stability of 1a, 2a and 1h in pyr·9HF

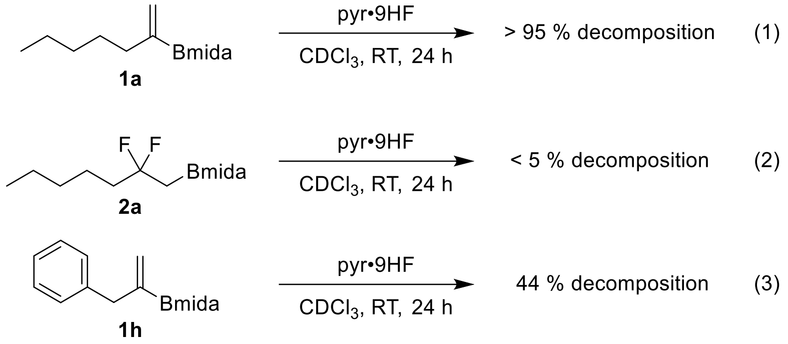

Scheme S9. Stability of compounds 1a, 2a and 1h in pyr·9HF **(see Eqs 1-3 in the paper).**

A 15 mL polypropylene (PP) tube fitted with a PP-screw cap, containing a Teflon magnetic stir bar was charged with MIDA boronates (0.02 mmol) before $CDCl_3$ was added (0.5 mL) (Scheme S9). Then, the pyr·9HF (0.17 mL, 6.5 mmol HF) was added by syringe. The reaction mixture was stirred at room temperature. After 24 h, it was quenched by addition of aq. sat. $NaHCO_3$ solution (8 mL) and stirred for 5 min. The biphasic mixture was poured into a phase separator (25 mL, Biotage) and the organic phase was collected in a flask. The PP tube was rinsed twice with $CDCl_3$ and after collection of the entire organic phase. Then appropriate amount of CH_2Br_2 (5 or 6 equiv.) was added as internal standard. The decomposition rate was then confirmed by ^1H NMR.

Transformation of Bmida group to Bpin group

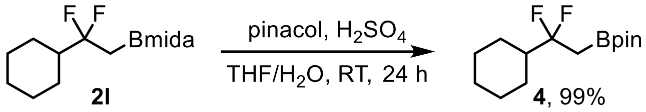

Scheme S10. Conversion of 2l to 4. (see Eq 4 in the paper).

A 5 mL glass vial containing a Teflon magnetic stir bar were charged with MIDA boronate **2l** (0.1 mmol) and pinacol (0.4 mmol) before THF (1 mL) was added (Scheme S10). Then, 0.1 mmol H_2SO_4 (95-97%) in 0.1 mL H_2O was added and the vial was sealed. The mixture was stirred for 24 h at rt before being quenched by the addition of water (10 mL). The mixture was then extracted with pentane (10 mL). The organic layer was washed twice with water (10 mL for each time), and dried over with anhydrous $MgSO_4$ for 30 min. Then, the mixture was filtered and concentrated to give product **4** as a colorless oil (27 mg, 99% yield). Note: chromatography was not needed, as the product is sucfficiently pure. Compound **4** decomposed, when silica gel chromatography was attempted.

^{1}H NMR (400 MHz, $CDCl_3$): δ = 1.88 – 1.75 (m, 4H), 1.71 – 1.61 (m, 1H), 1.51 (t, J = 20.5 Hz, 2H), 1.36 – 1.08 (m, 18H) ppm; **^{13}C NMR** (100 MHz, $CDCl_3$): δ = 126.7 (t, J = 241.6 Hz), 83.9, 45.5 (t, J = 24.2 Hz), 26.2 (t, J = 4.5 Hz), 26.1, 25.9, 24.8 ppm; **^{19}F NMR** (377 MHz, $CDCl_3$): δ = -93.67 (td, J = 20.4, 13.1 Hz) ppm; **^{11}B NMR** (128 MHz, $CDCl_3$) δ = 31.98 ppm. **HRMS** (ESI): m/z calcd. for $C_{14}H_{25}BF_2O_2+Na^+$: 297.1808 [M+Na]$^+$; found: 297.1810.

^{1}H NMR ((CD$_3$)$_2$CO, 400 MHz). 2-(Hept-1-en-2-yl)-6-methyl-1,3,6,2-dioxazaborocane-4,8-dione (**1a**)

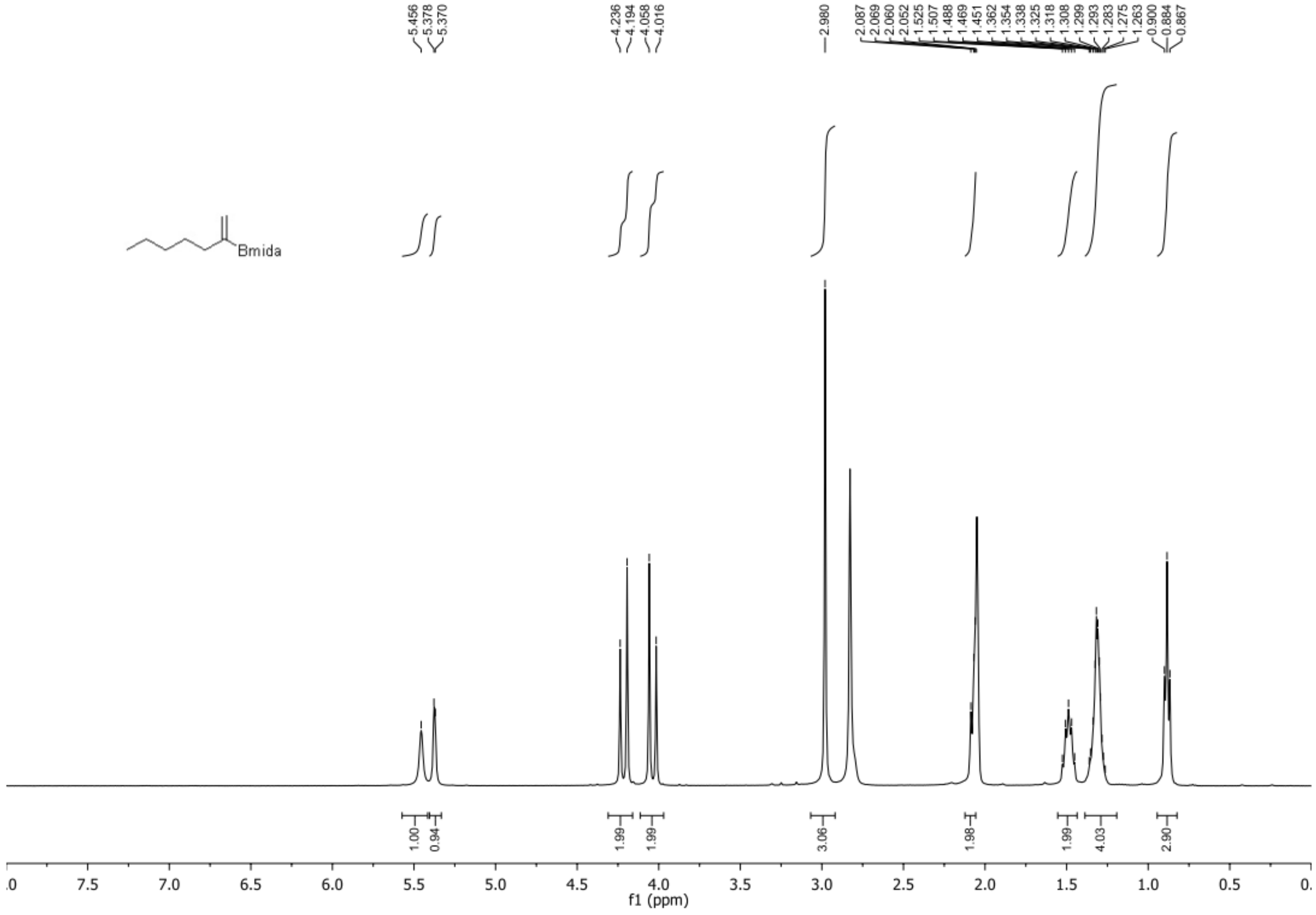

^{13}C NMR ($(CD_3)_2CO$, 100 MHz). 2-(Hept-1-en-2-yl)-6-methyl-1,3,6,2-dioxazaborocane-4,8-dione (**1a**)

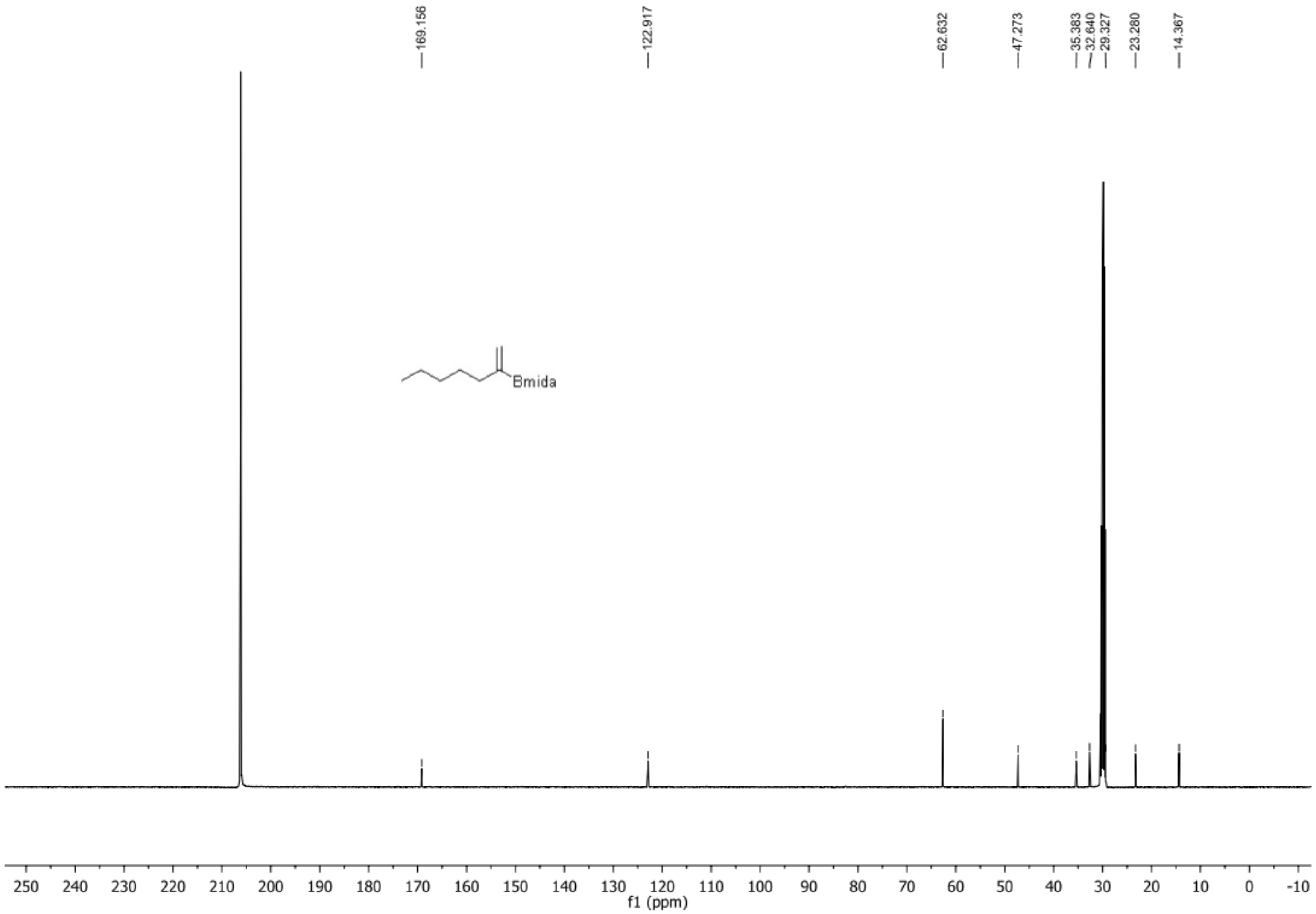

^{1}H NMR ($CDCl_3$, 400 MHz). 6-Methyl-2-(non-1-en-2-yl)-1,3,6,2-dioxaza-borocane-4,8-dione (**1b**)

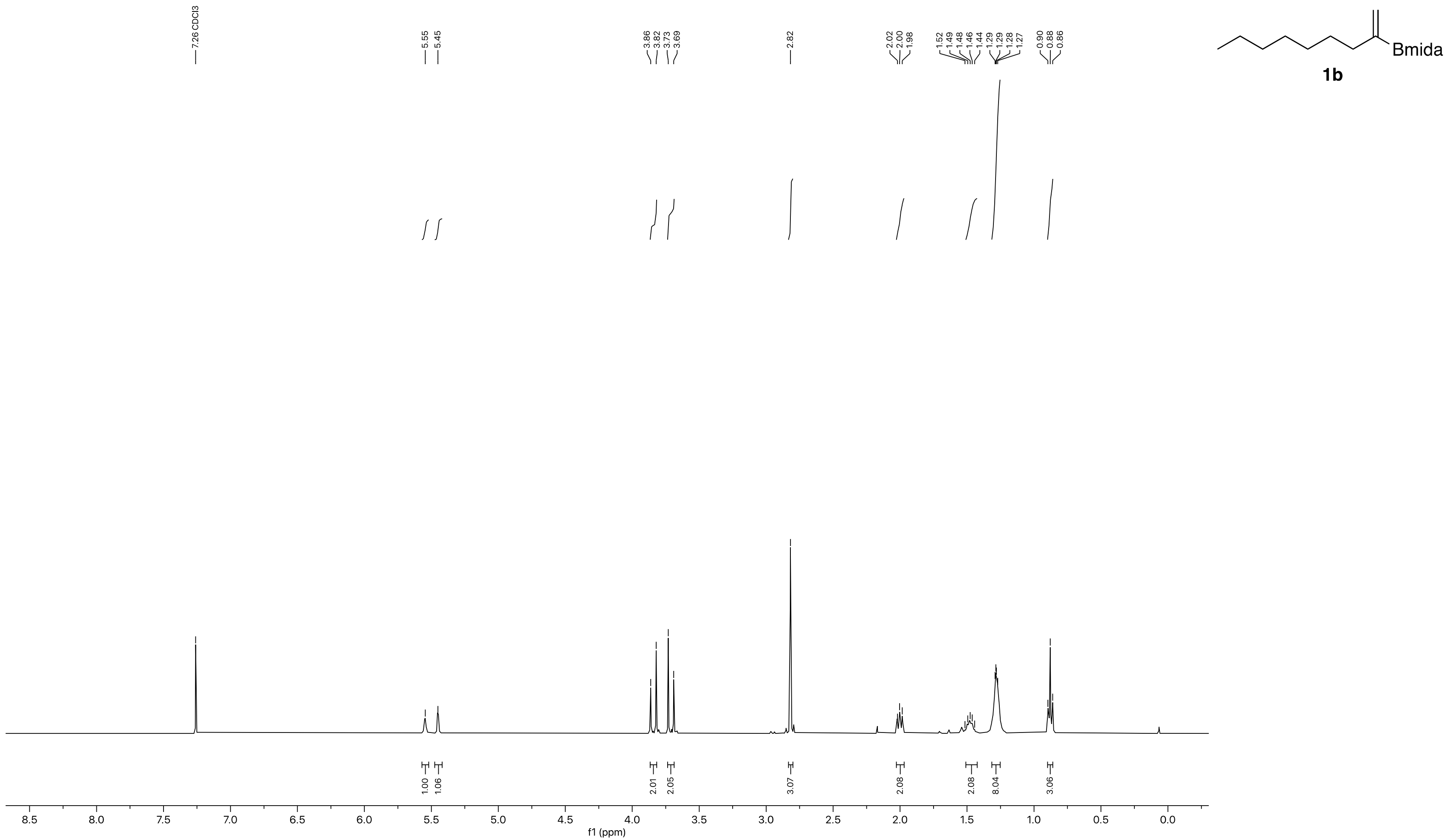

1b

^{13}C NMR ($CDCl_3$, 100 MHz). 6-Methyl-2-(non-1-en-2-yl)-1,3,6,2-dioxaza-borocane-4,8-dione (**1b**)

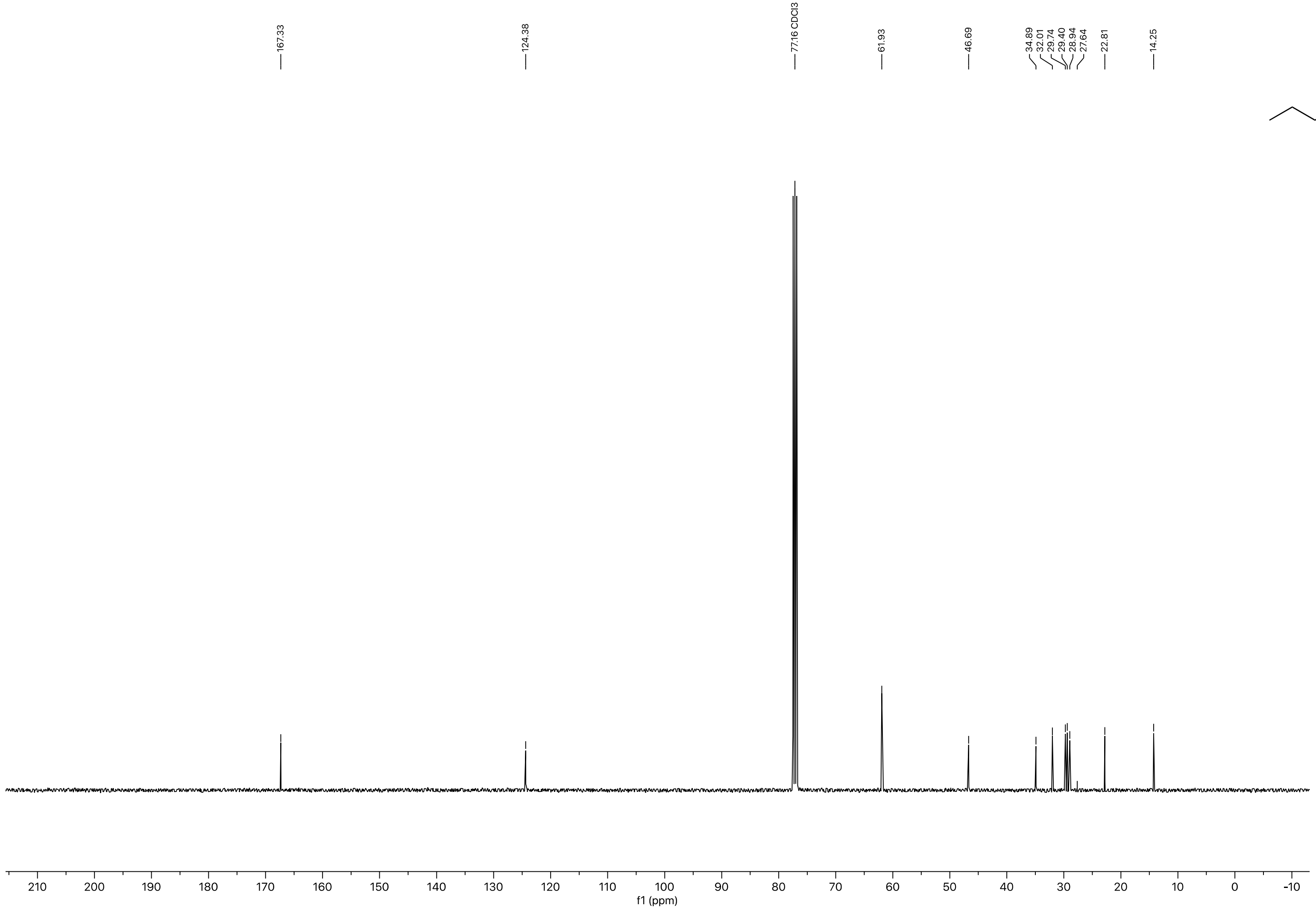

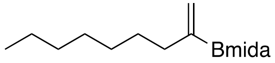

1b

^{1}H NMR ($CDCl_3$, 400 MHz). 6-Methyl-2-(pent-1-en-2-yl)-1,3,6,2-dioxazaborocane-4,8-dione (**1c**)

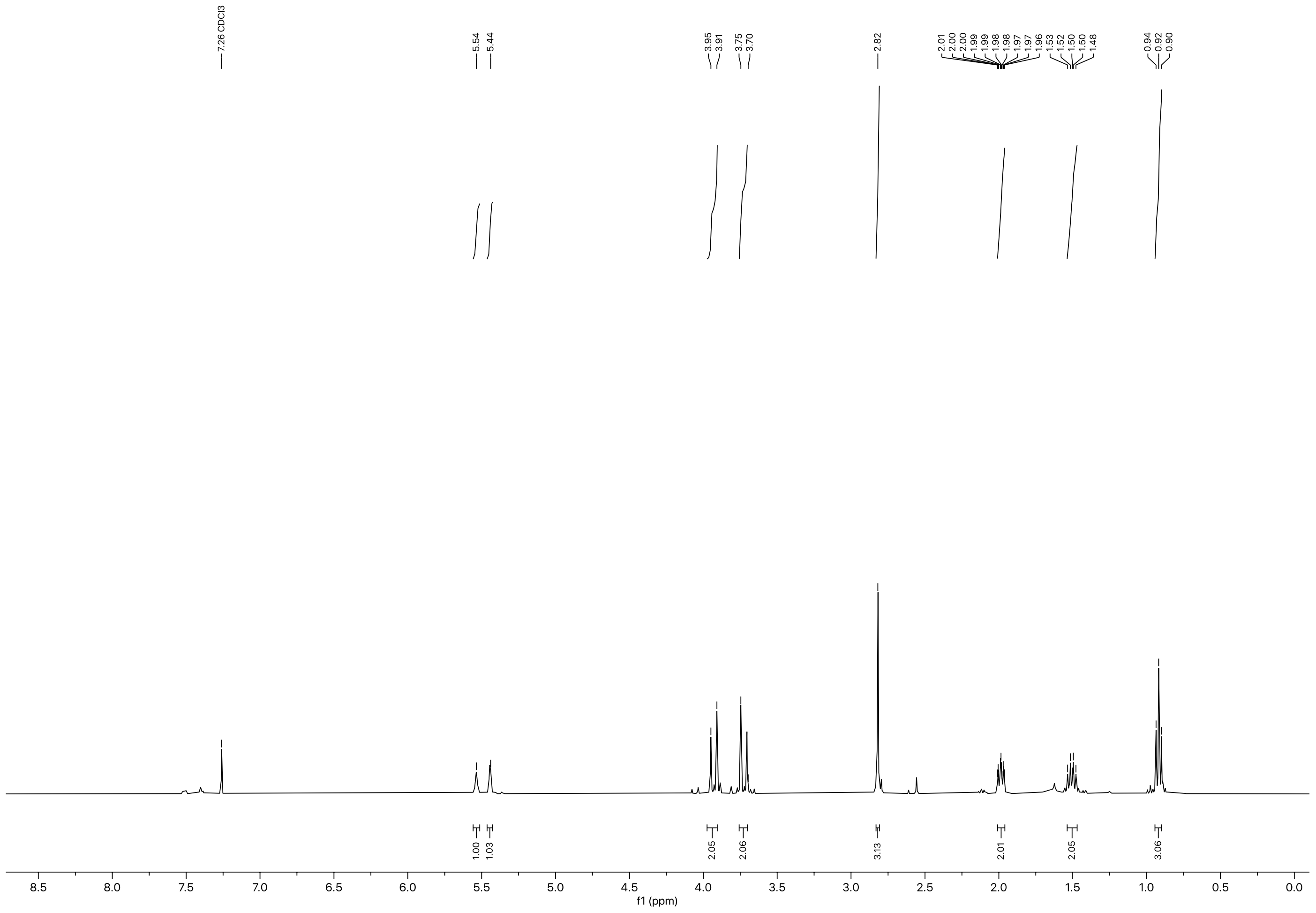

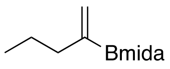

1c

^{13}C NMR ($CDCl_3$, 100 MHz). 6-Methyl-2-(pent-1-en-2-yl)-1,3,6,2-dioxazaborocane-4,8-dione (**1c**)

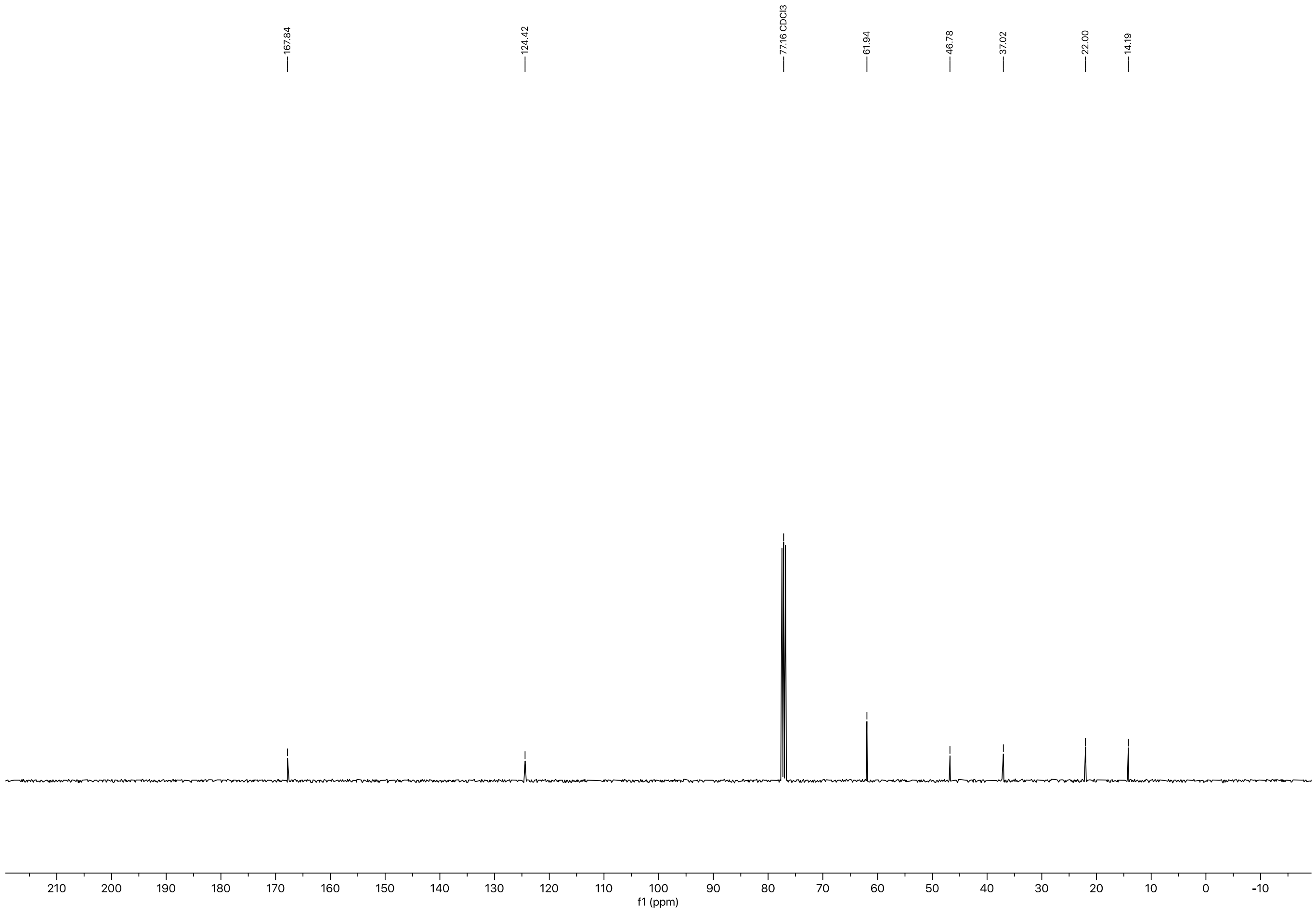

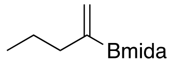

1c

^{1}H NMR (($(CD_3)_2CO$, 400 MHz). 6-Methyl-2-(5-methylhex-1-en-2-yl)-1,3,6,2-dioxazaborocane-4,8-dione (**1d**)

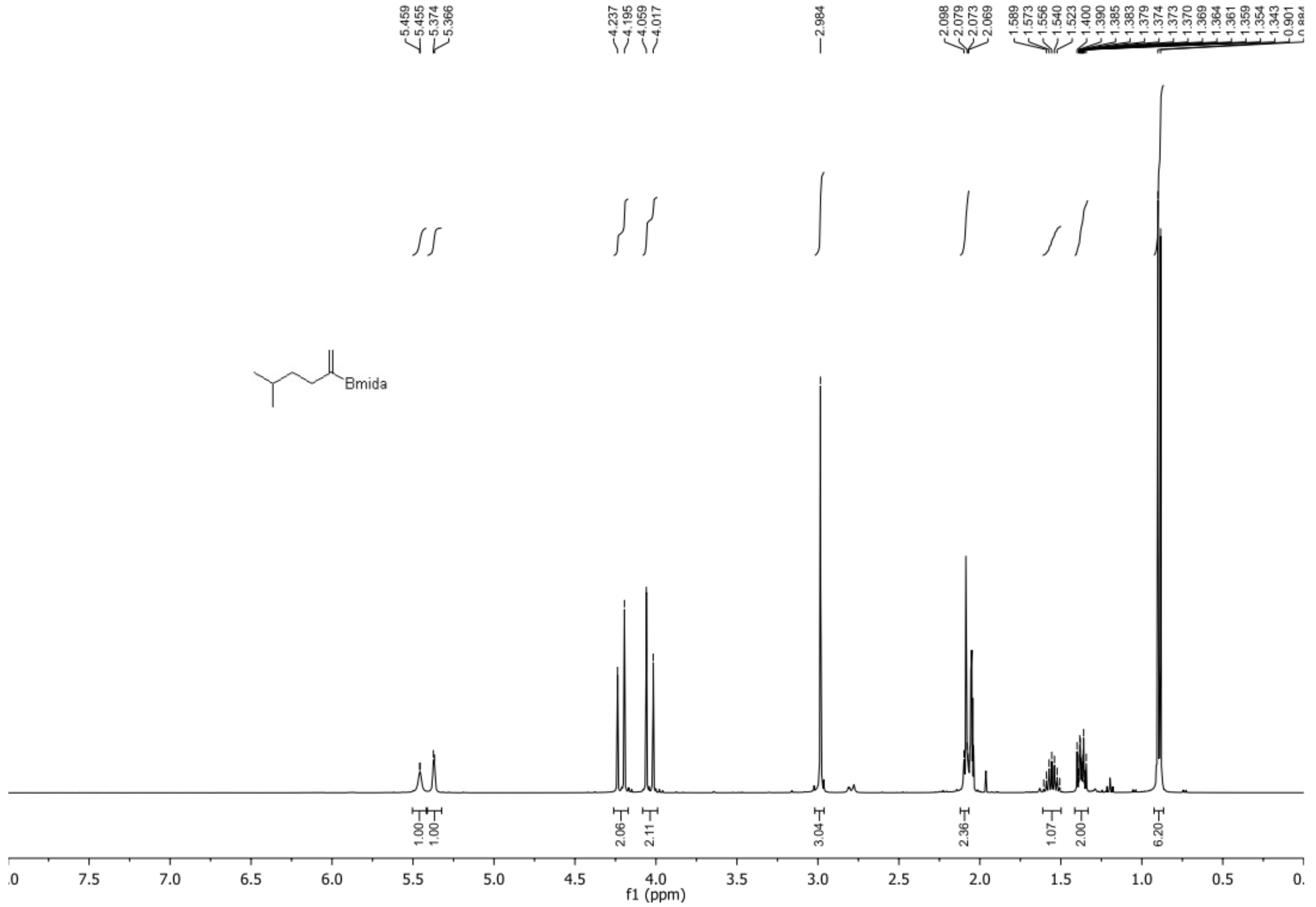

^{13}C NMR ($(CD_3)_2CO$, 100 MHz). 6-Methyl-2-(5-methylhex-1-en-2-yl)-1,3,6,2-dioxazaborocane-4,8-dione (**1d**)

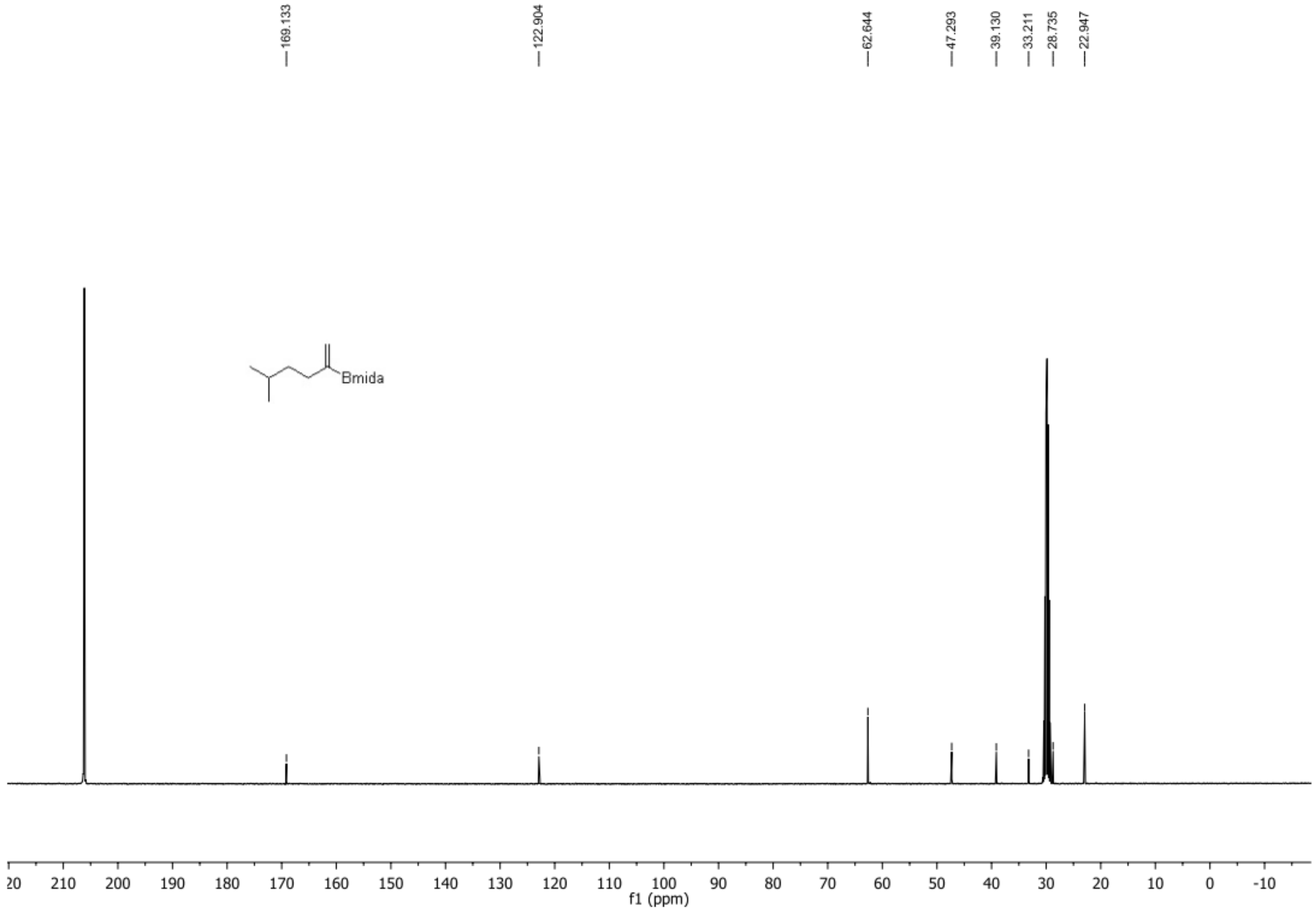

^{1}H NMR ($(CD_3)_2CO$, 400 MHz). 2-(3-Cyclohexylprop-1-en-2-yl)-6-methyl-1,3,6,2-dioxazaborocane-4,8-dione (**1e**)

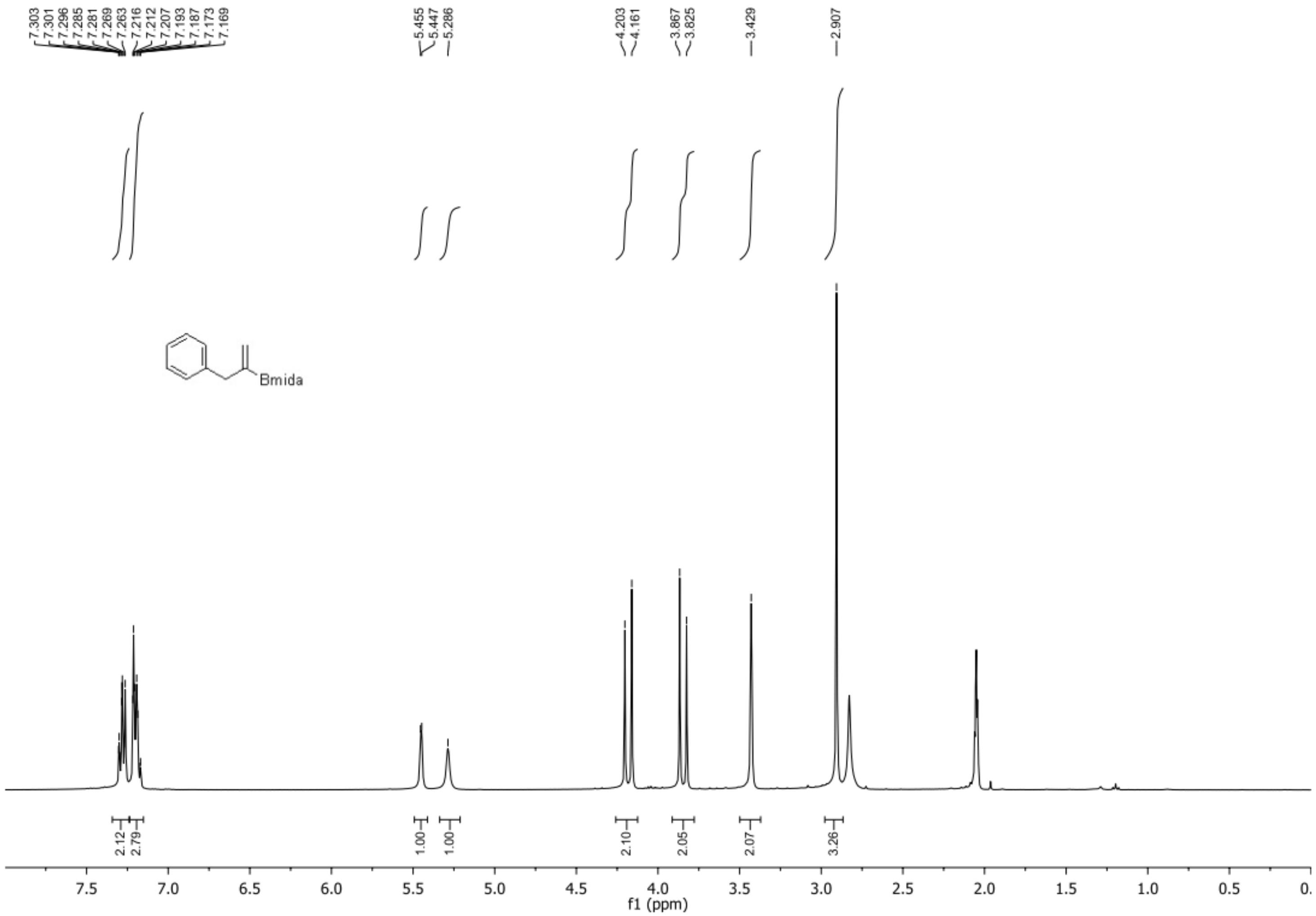

^{13}C NMR ($(CD_3)_2CO$, 100 MHz). 2-(3-Cyclohexylprop-1-en-2-yl)-6-methyl-1,3,6,2-dioxazaborocane-4,8-dione (**1e**)

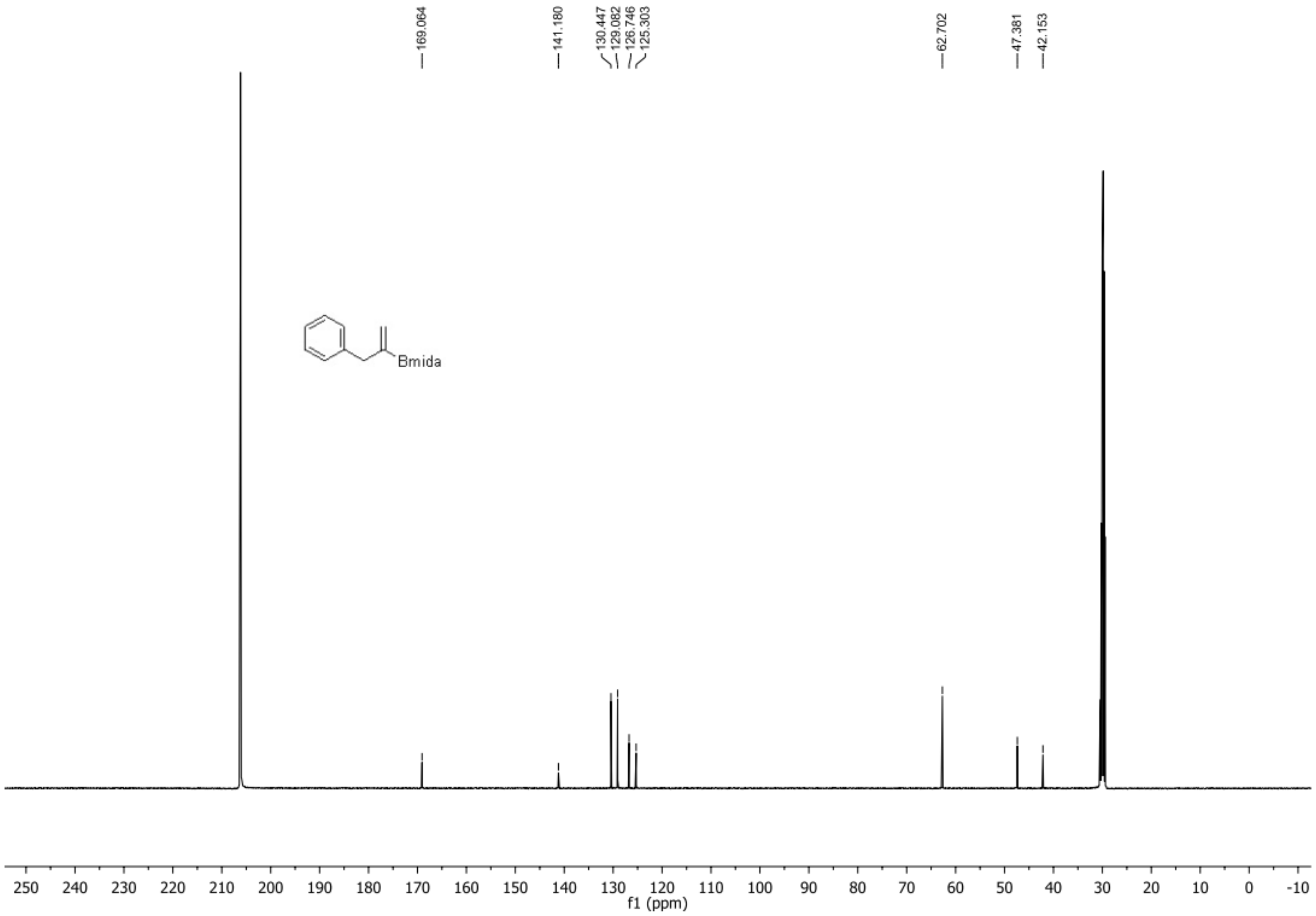

^{1}H NMR ($(CD_3)_2CO$, 400 MHz). 6-Methyl-2-(4-phenylbut-1-en-2-yl)-1,3,6,2-dioxazaborocane-4,8-dione (**1f**)

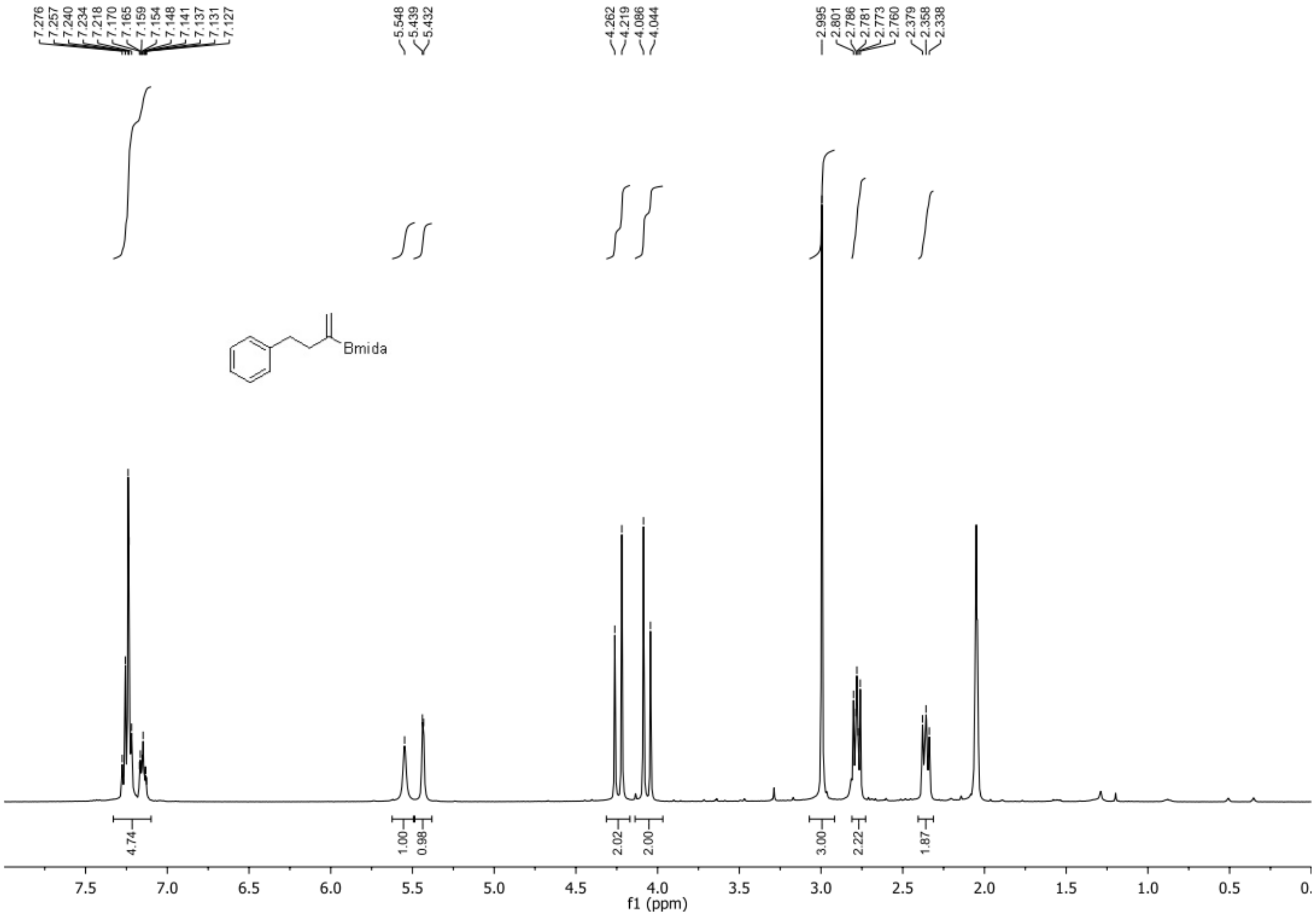

^{13}C NMR ($(CD_3)_2CO$, 100 MHz). 6-Methyl-2-(4-phenylbut-1-en-2-yl)-1,3,6,2-dioxazaborocane-4,8-dione (**1f**)

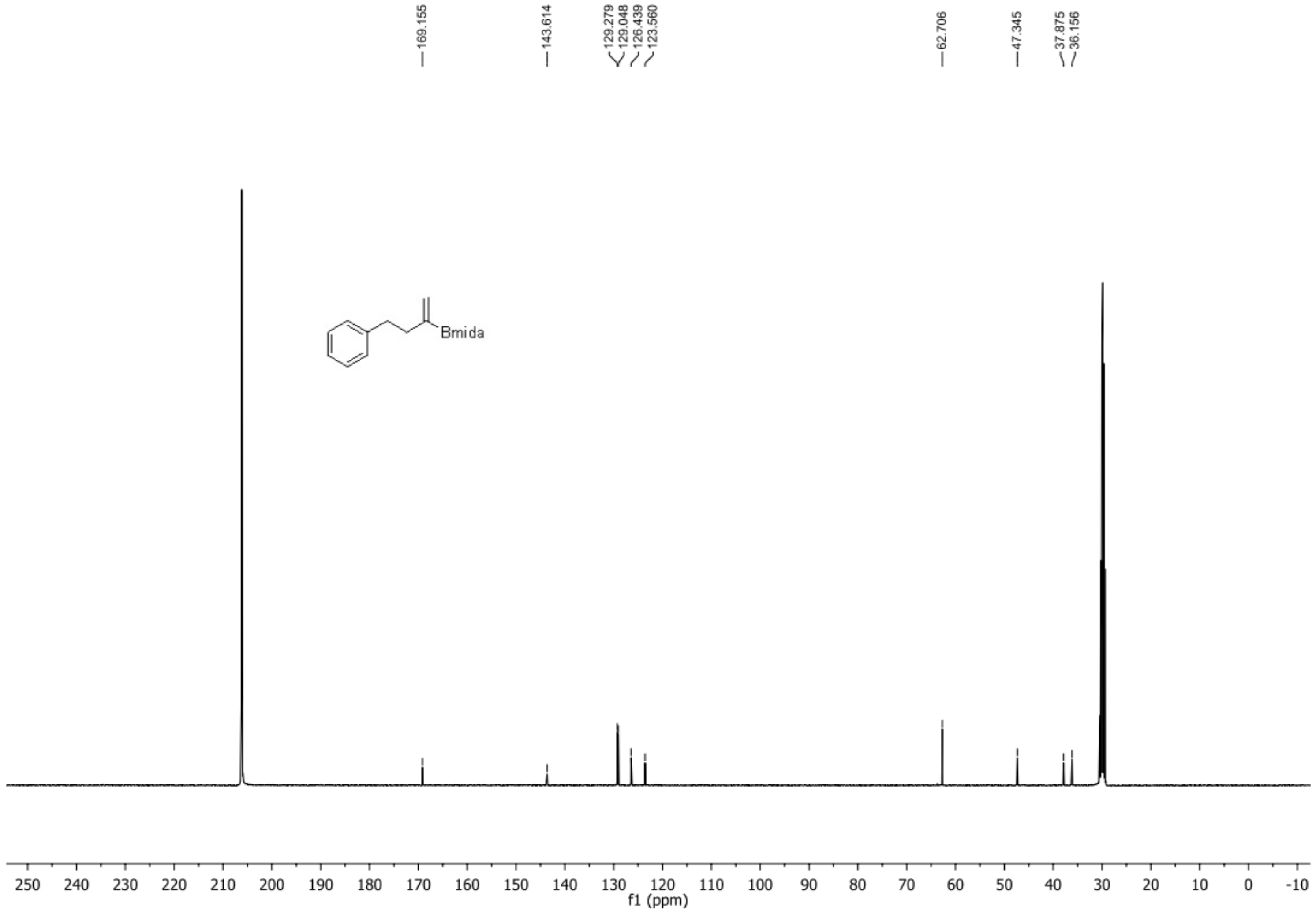

^{1}H NMR ($(CD_3)_2CO$, 400 MHz). 6-Methyl-2-(3-phenylprop-1-en-2-yl)-1,3,6,2-dioxazaborocane-4,8-dione (**1h**)

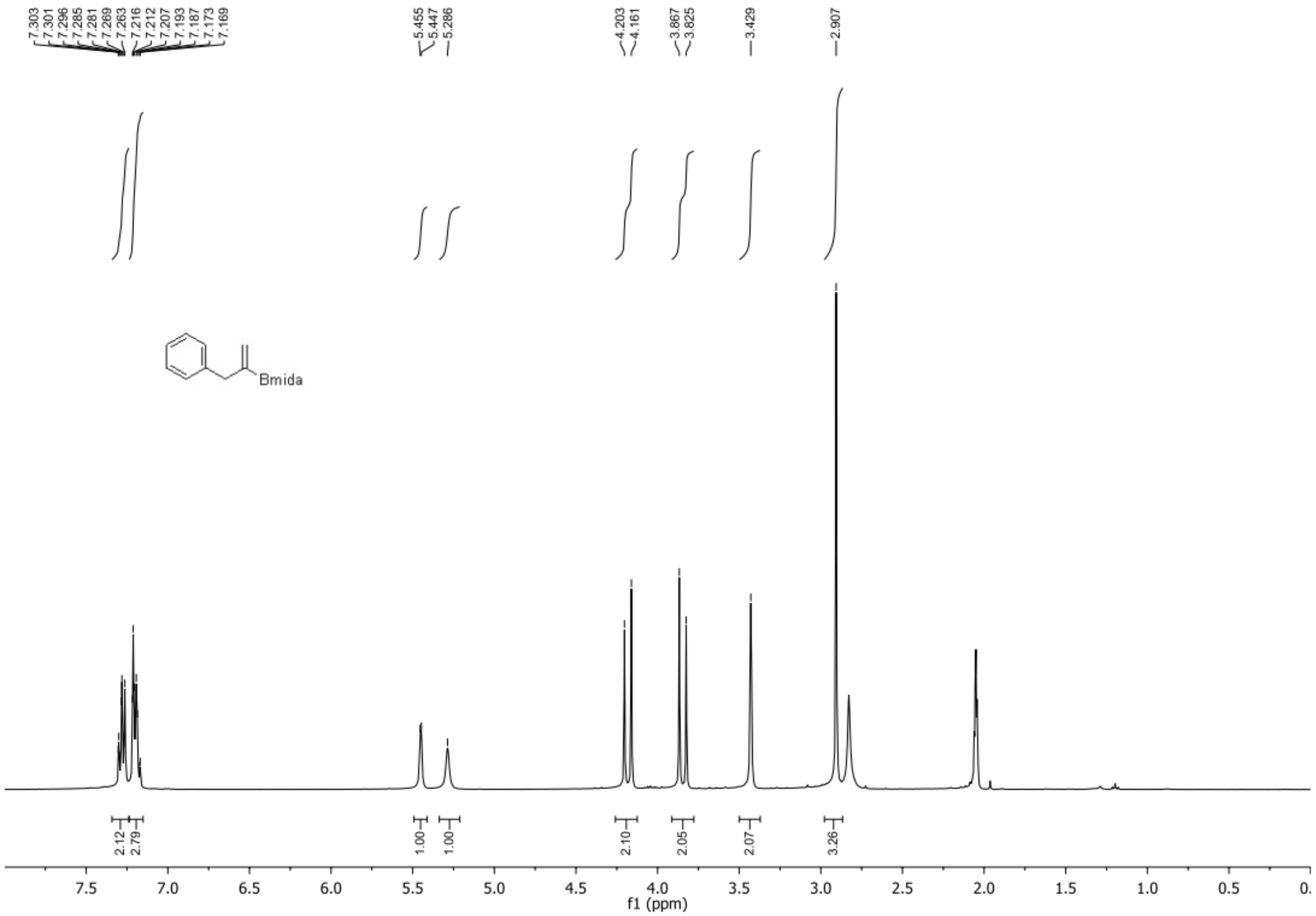

^{13}C NMR ($(CD_3)_2CO$, 100 MHz). 6-Methyl-2-(3-phenylprop-1-en-2-yl)-1,3,6,2-dioxazaborocane-4,8-dione (**1h**)

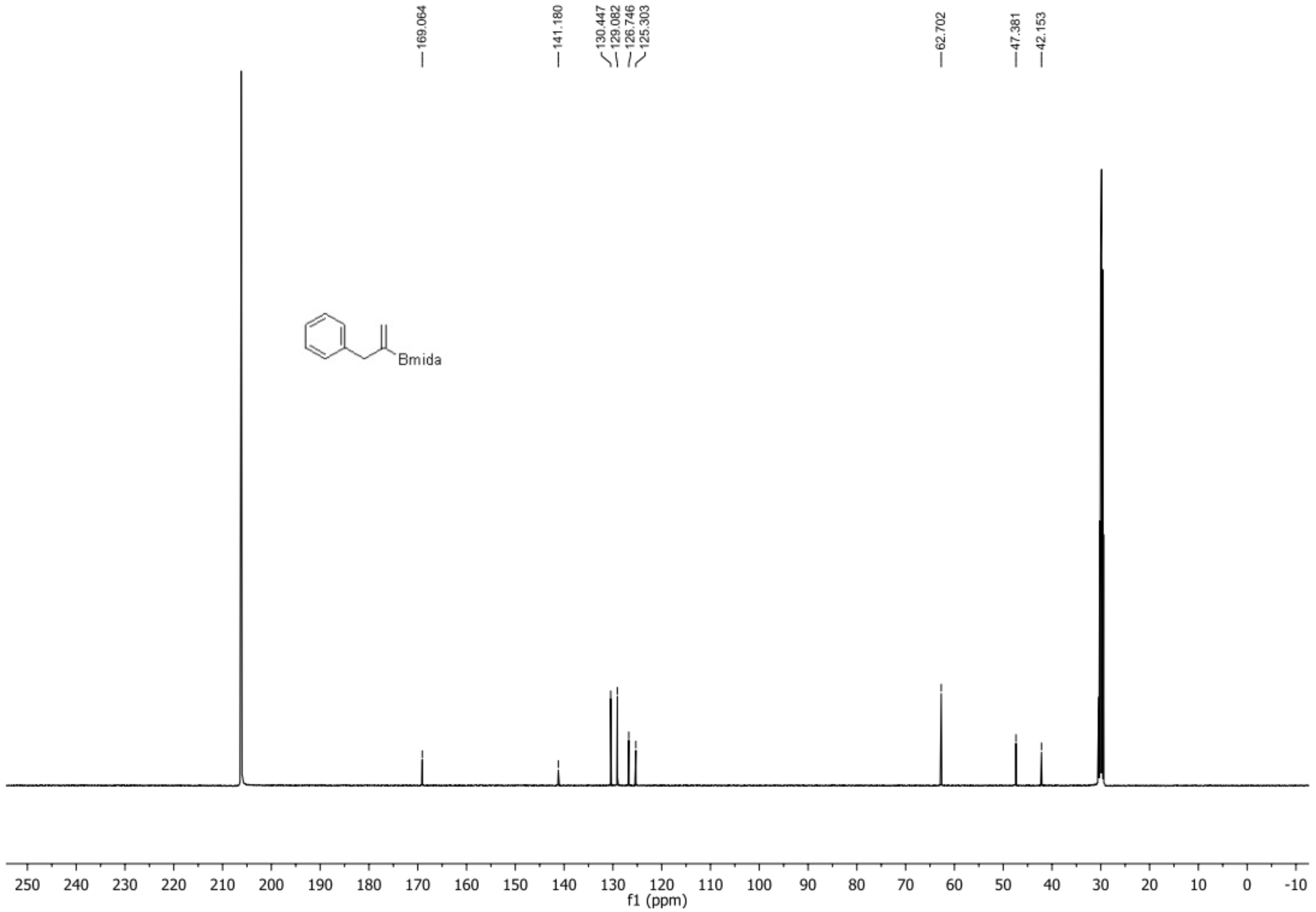

^{1}H NMR ($CDCl_3$, 400 MHz). 6-Methyl-2-(3-(p-tolyl)prop-1-en-2-yl)-1,3,6,2-dioxazaborocane-4,8-dione (**1i**)

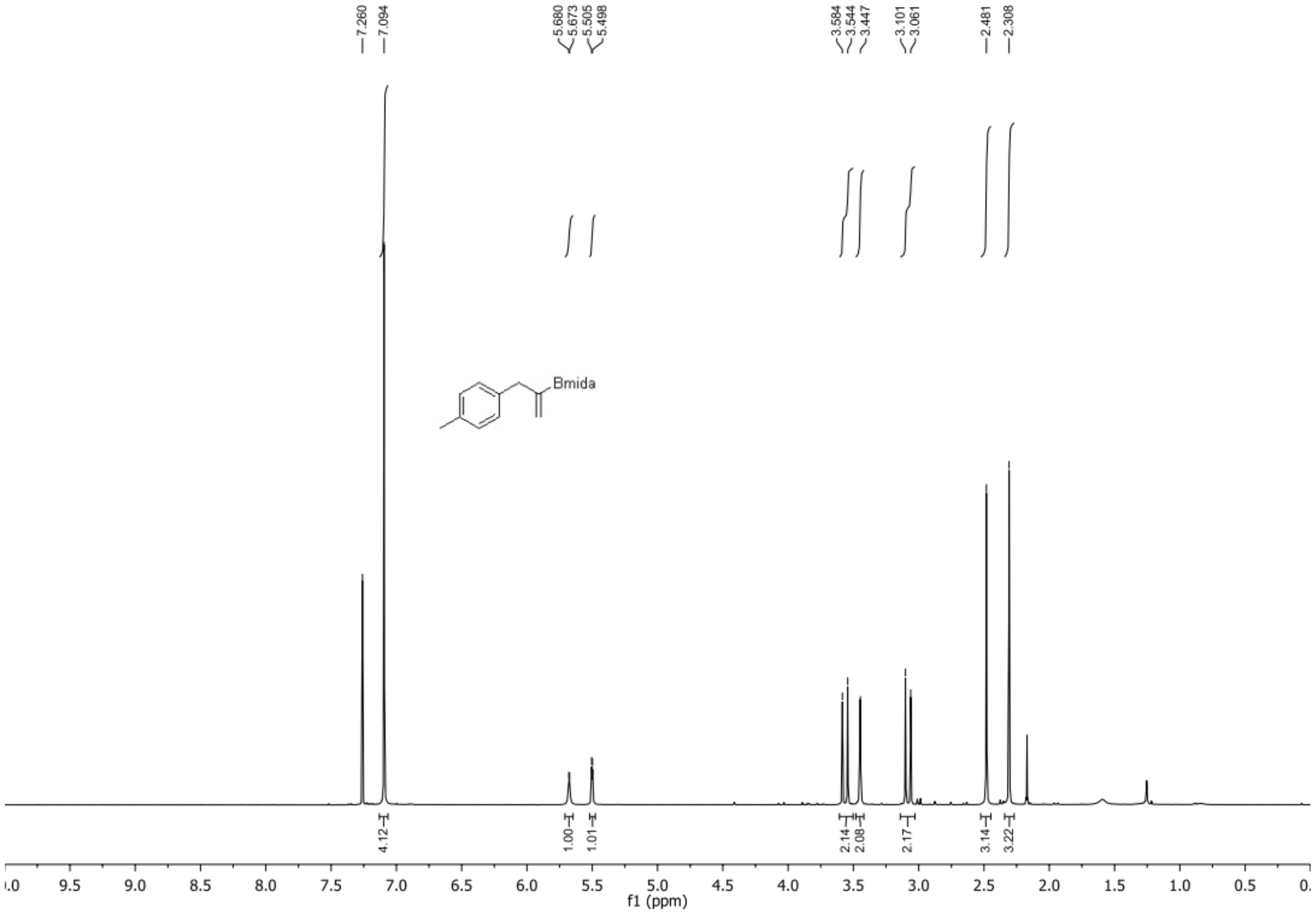

^{13}C NMR ($CDCl_3$, 100 MHz). 6-Methyl-2-(3-(p-tolyl)prop-1-en-2-yl)-1,3,6,2-dioxazaborocane-4,8-dione (**1i**)

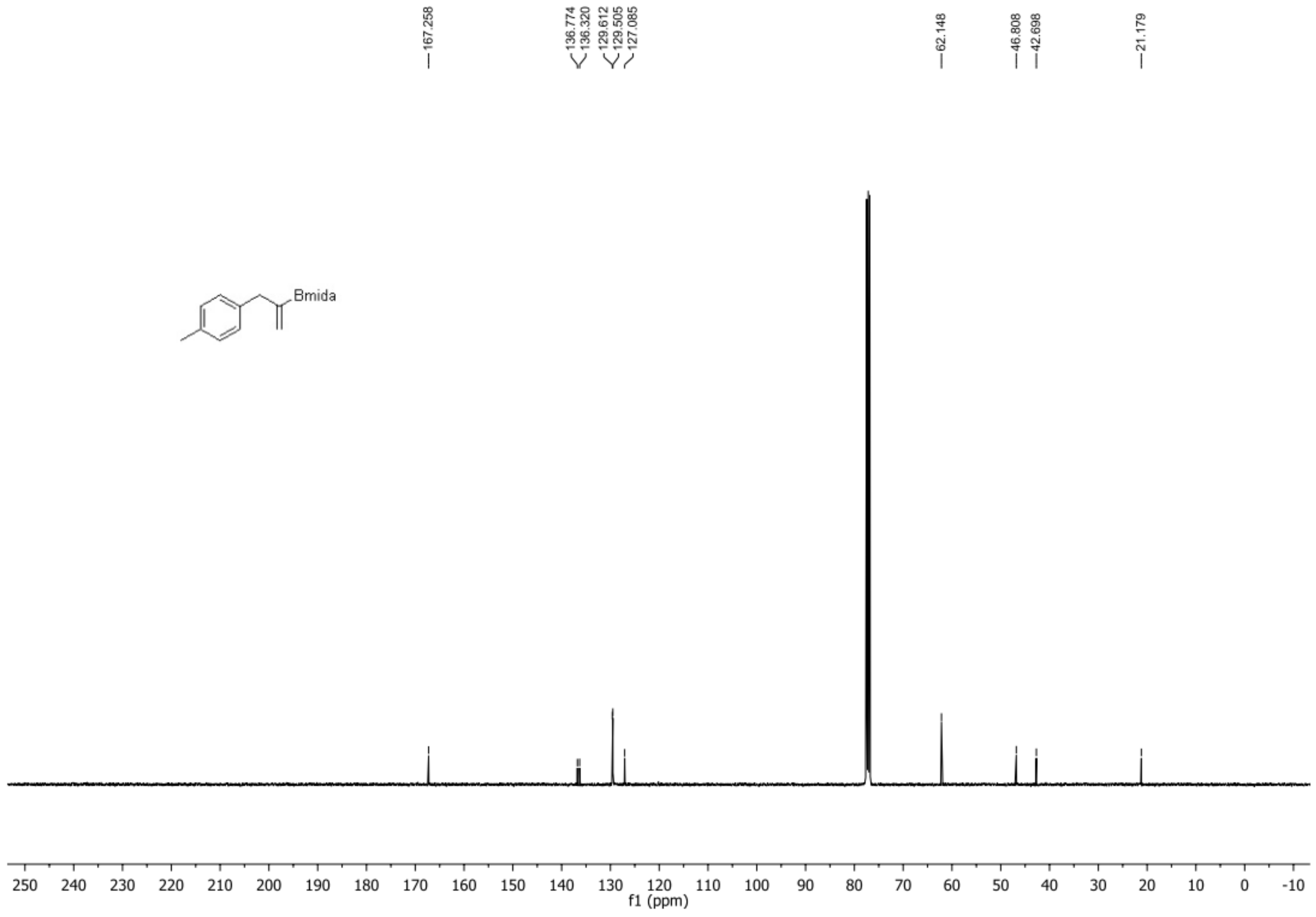

^{1}H NMR ($(CD_3)_2CO$, 400 MHz). 2-(3-(4-Bromophenyl)prop-1-en-2-yl)-6-methyl-1,3,6,2-dioxazaborocane-4,8-dione (**1j**)

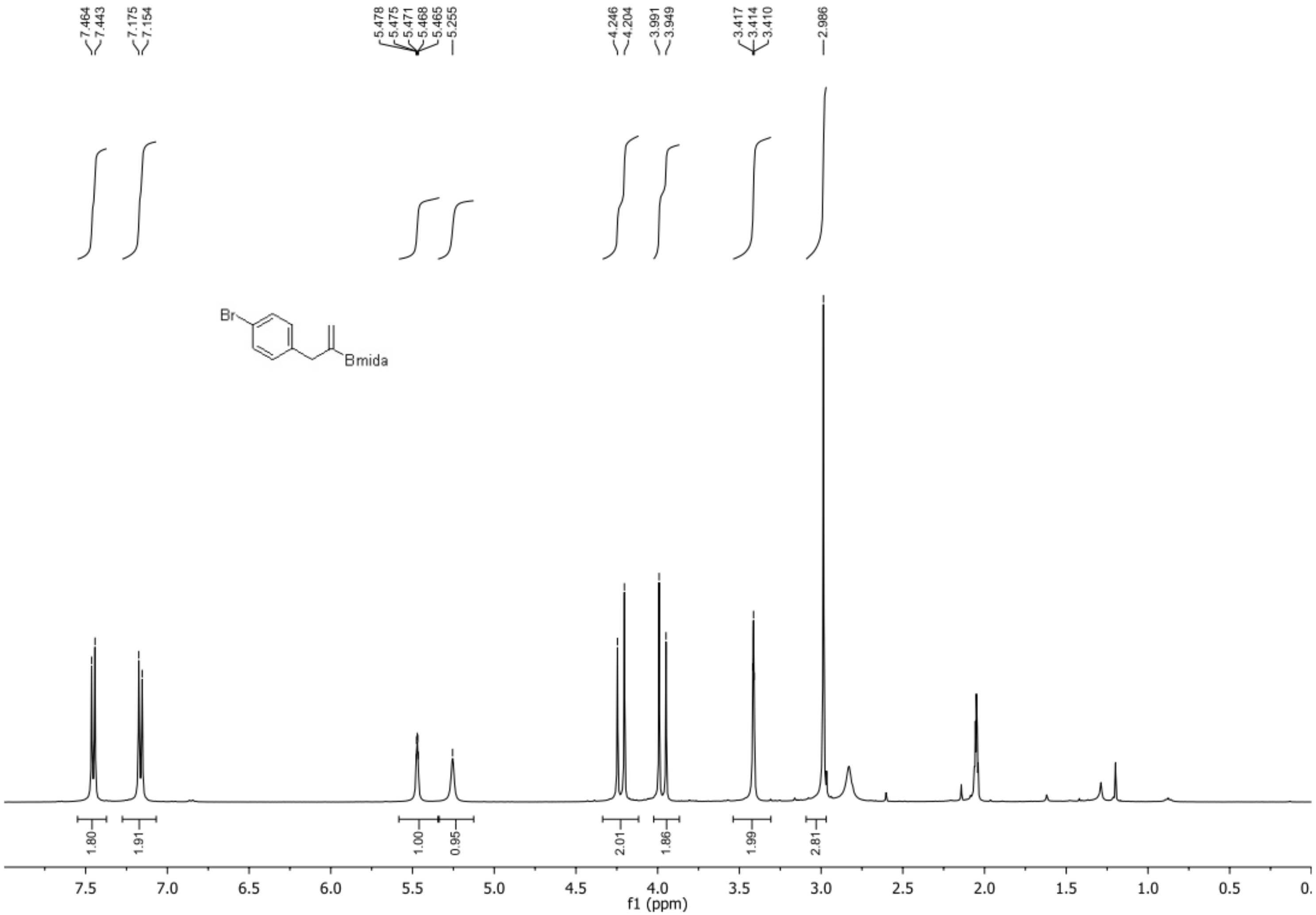

^{13}C NMR ($(CD_3)_2CO$, 100 MHz). 2-(3-(4-Bromophenyl)prop-1-en-2-yl)-6-methyl-1,3,6,2-dioxazaborocane-4,8-dione (**1j**)

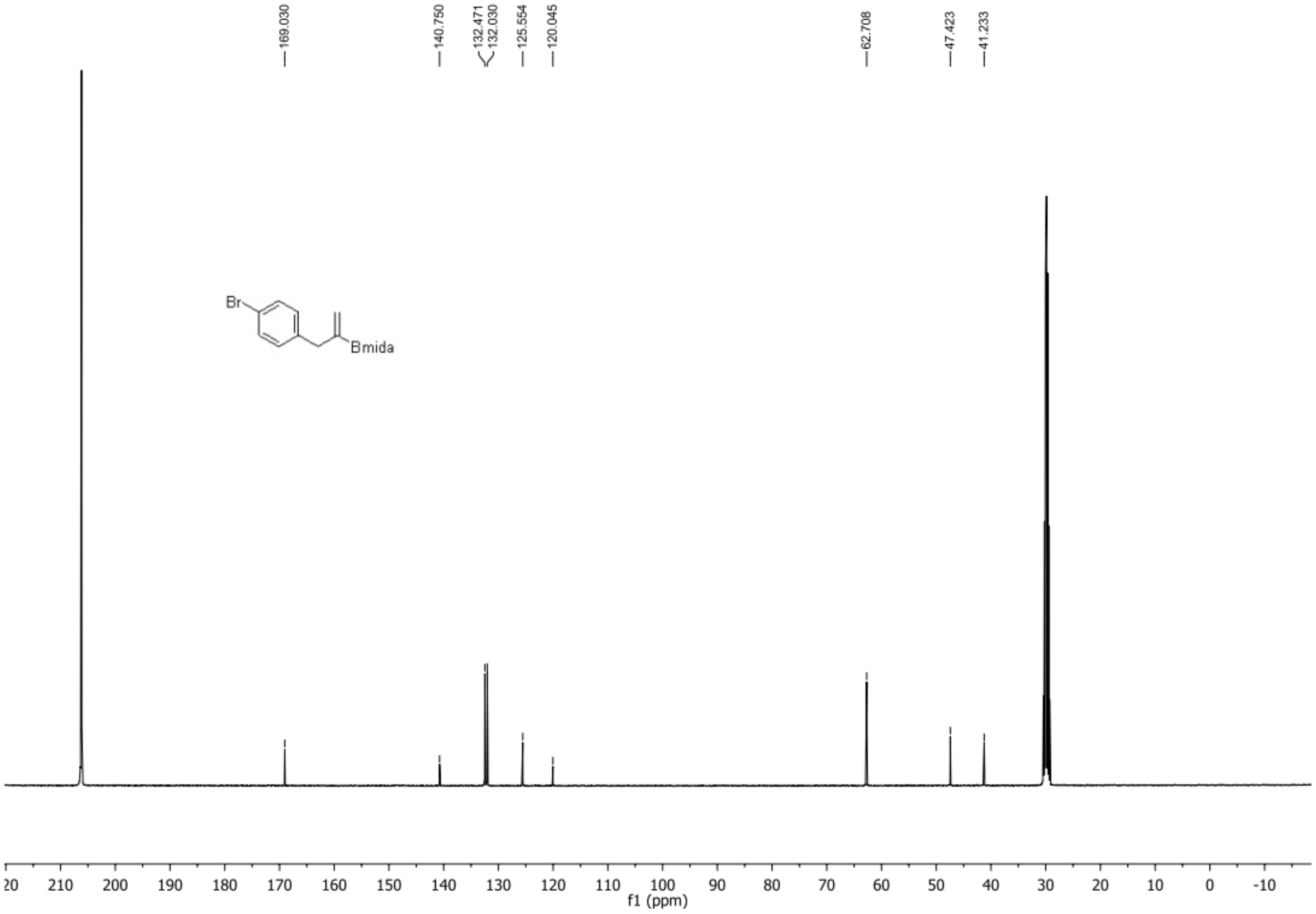

^{1}H NMR ($(CD_3)_2CO$, 400 MHz). 2-(4-(1,3-Dioxoisoindolin-2-yl)but-1-en-2-yl)-6-methyl-1,3,6,2-dioxazaborocane-4,8-dione (**1k**)

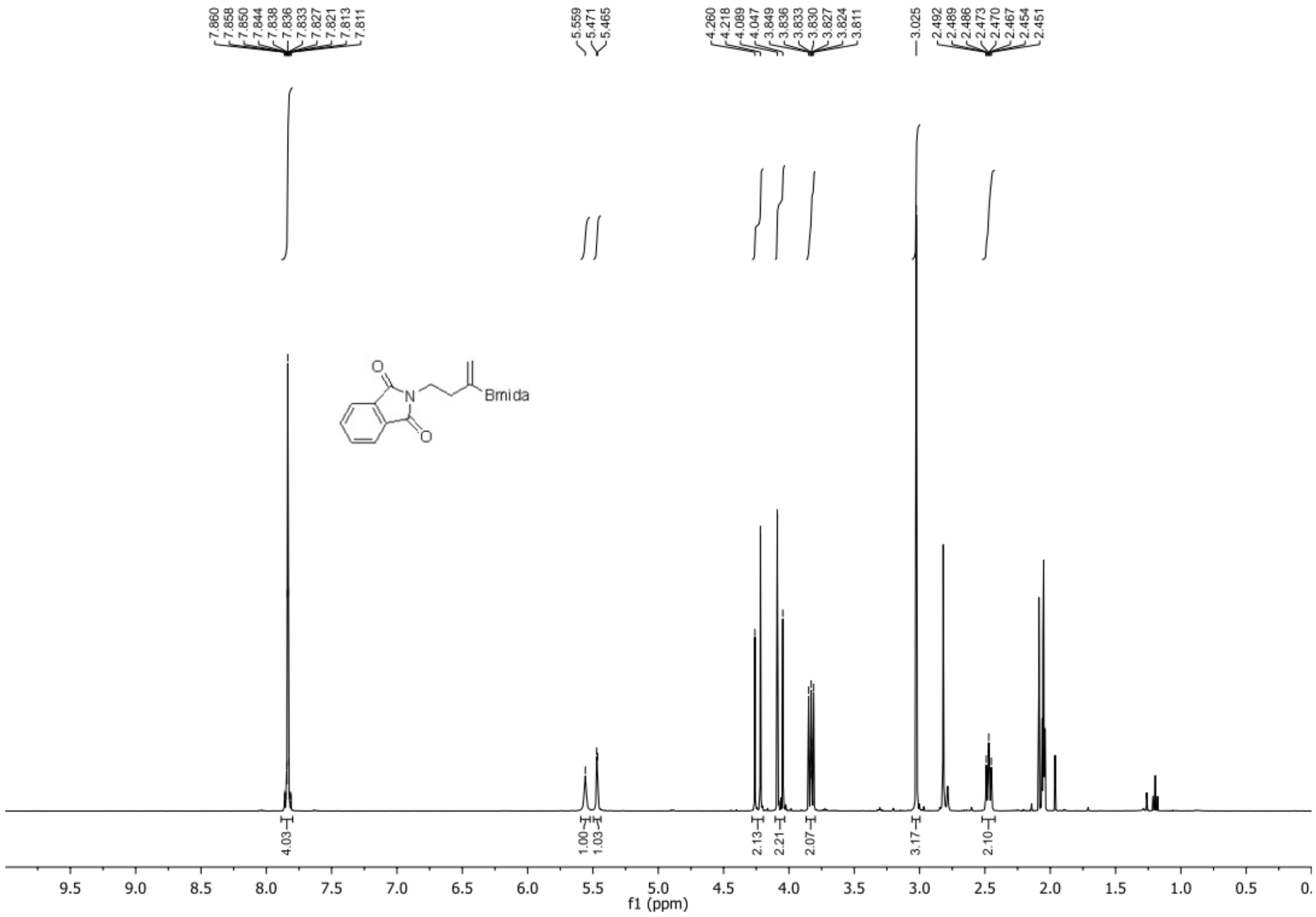

^{13}C NMR ($(CD_3)_2CO$, 100 MHz). 2-(4-(1,3-Dioxoisoindolin-2-yl)but-1-en-2-yl)-6-methyl-1,3,6,2-dioxazaborocane-4,8-dione (**1k**)

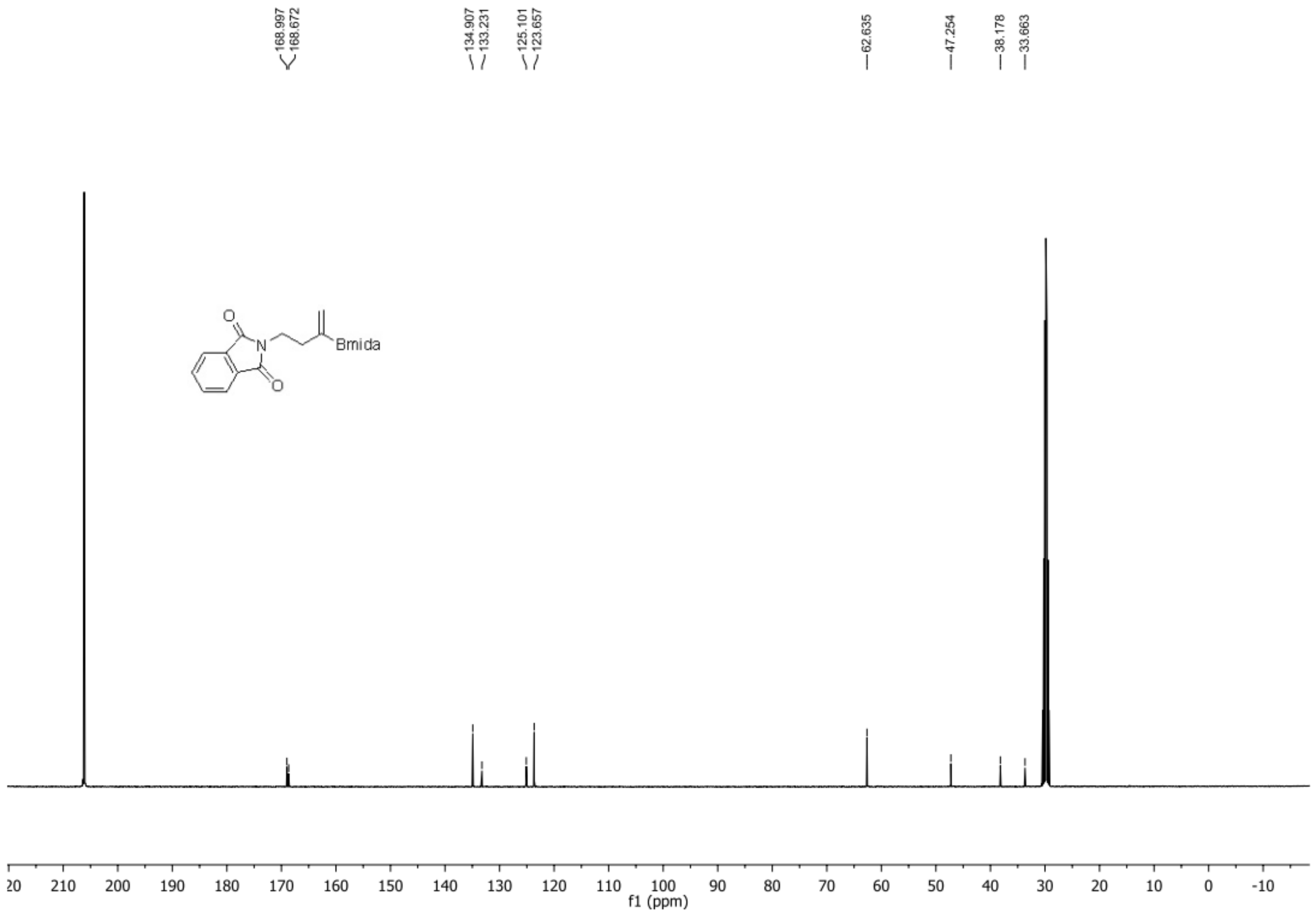

^{1}H NMR ($(CD_3)_2CO$, 400 MHz). 2-(1-Cyclohexylvinyl)-6-methyl-1,3,6,2-dioxazaborocane-4,8-dione (**1l**)

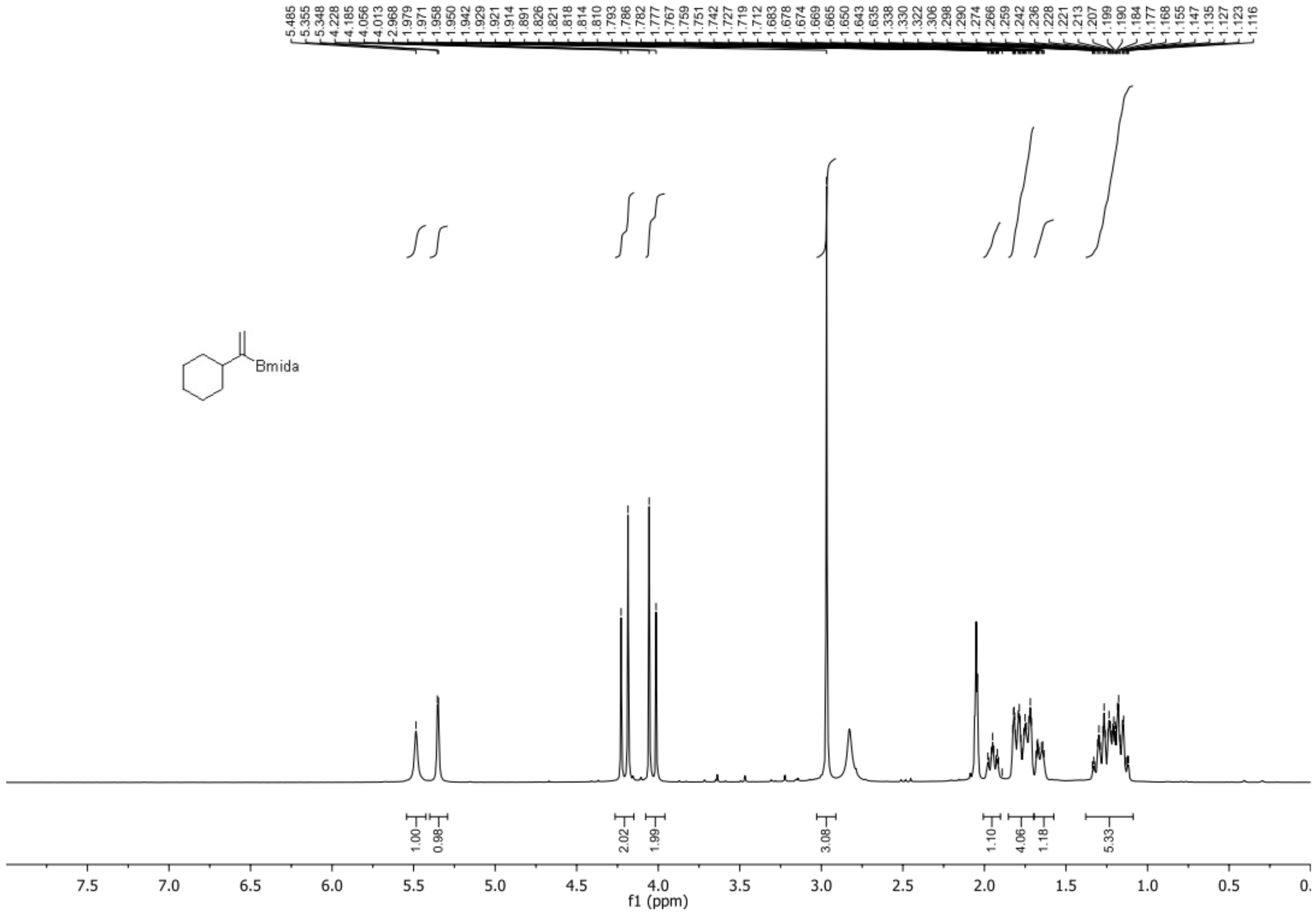

^{13}C NMR ($(CD_3)_2CO$, 100 MHz). 2-(1-Cyclohexylvinyl)-6-methyl-1,3,6,2-dioxazaborocane-4,8-dione (**1l**)

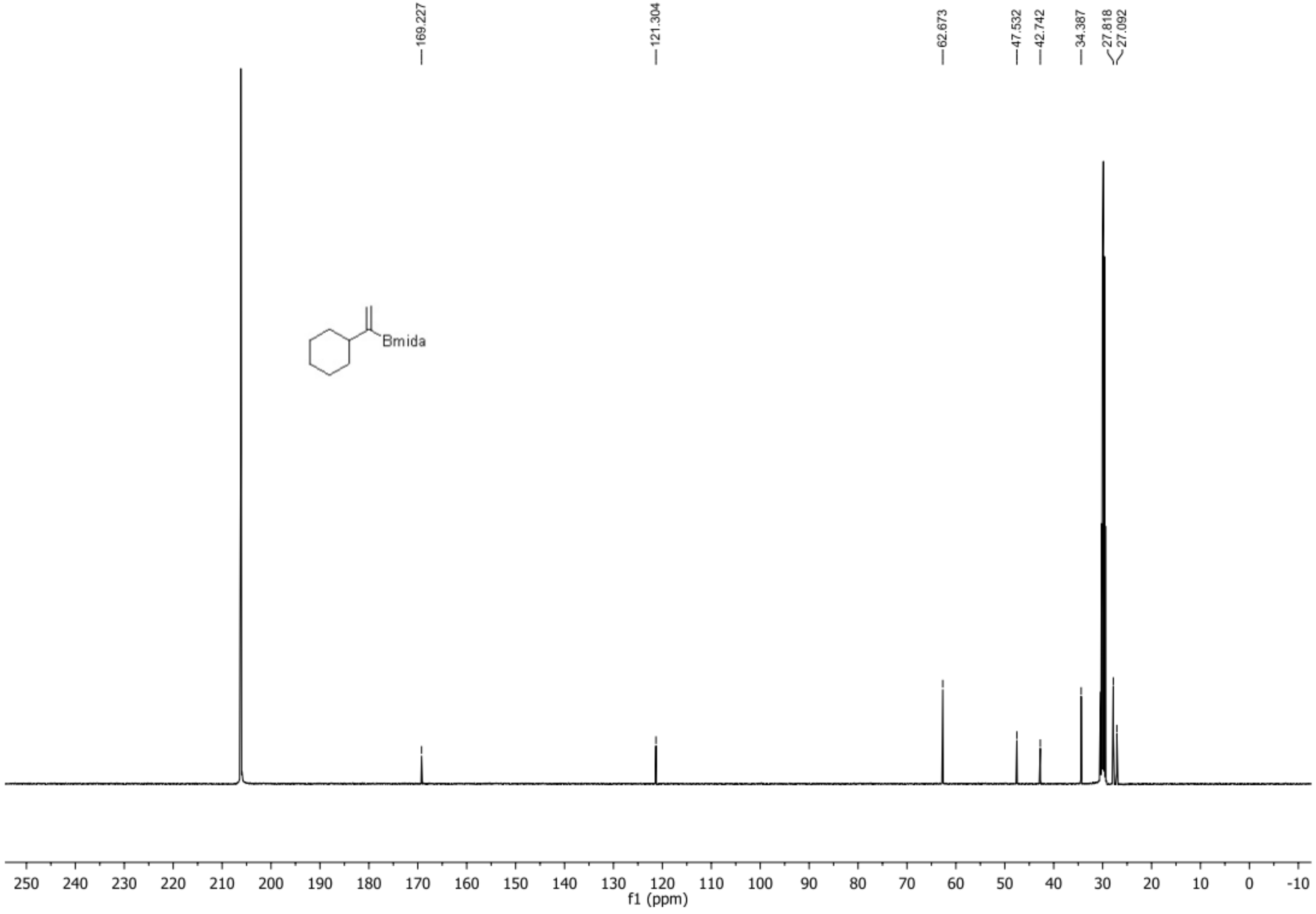

^{1}H NMR ($CDCl_3$, 400 MHz). 2-(2,2-Difluoroheptyl)-6-methyl-1,3,6,2-dioxazaborocane-4,8-dione (**2a**)

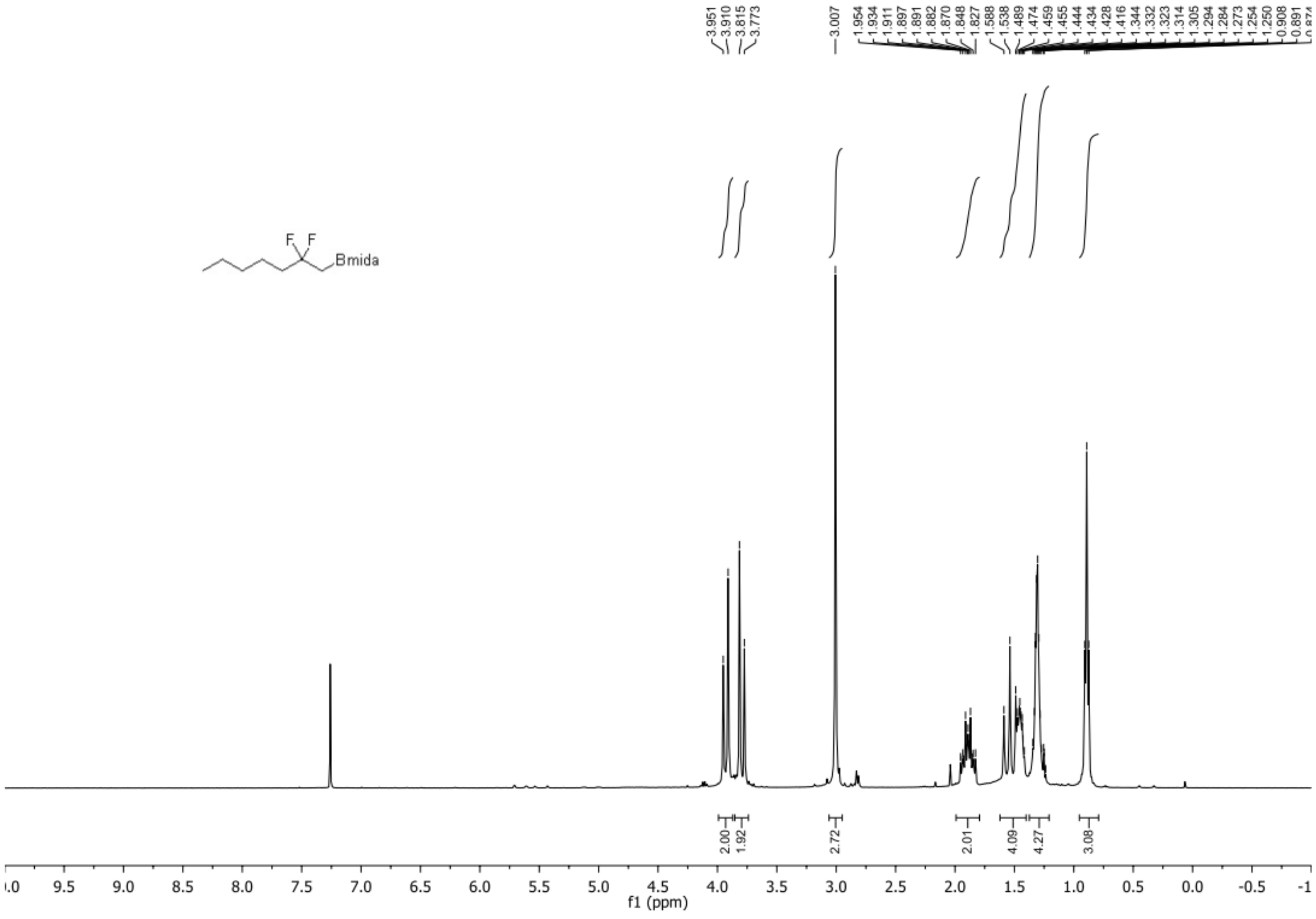

^{13}C NMR ($CDCl_3$, 100 MHz). 2-(2,2-Difluoroheptyl)-6-methyl-1,3,6,2-dioxazaborocane-4,8-dione (**2a**)

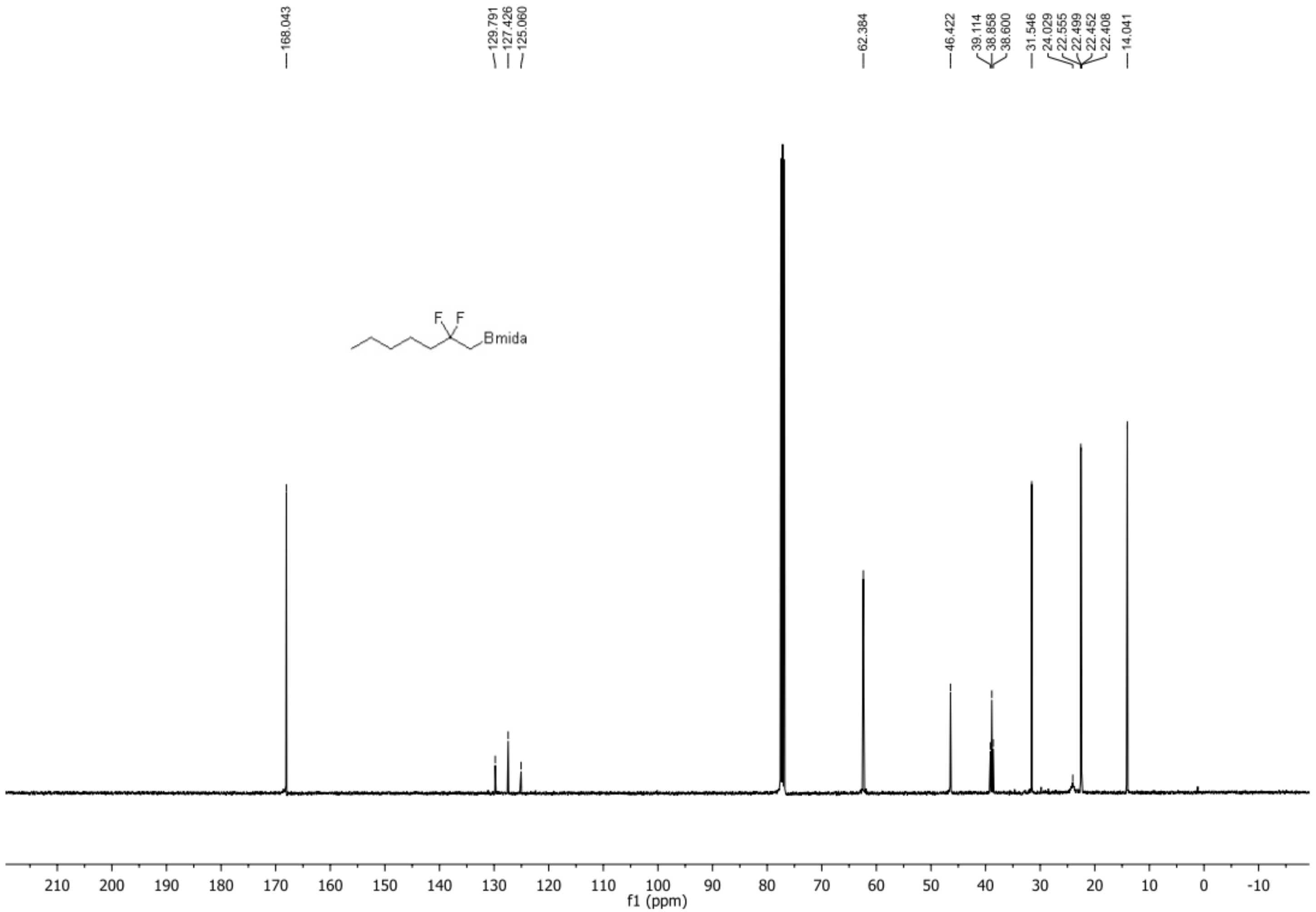

^{19}F NMR ($CDCl_3$, 377 MHz). 2-(2,2-Difluoroheptyl)-6-methyl-1,3,6,2-dioxazaborocane-4,8-dione (**2a**)

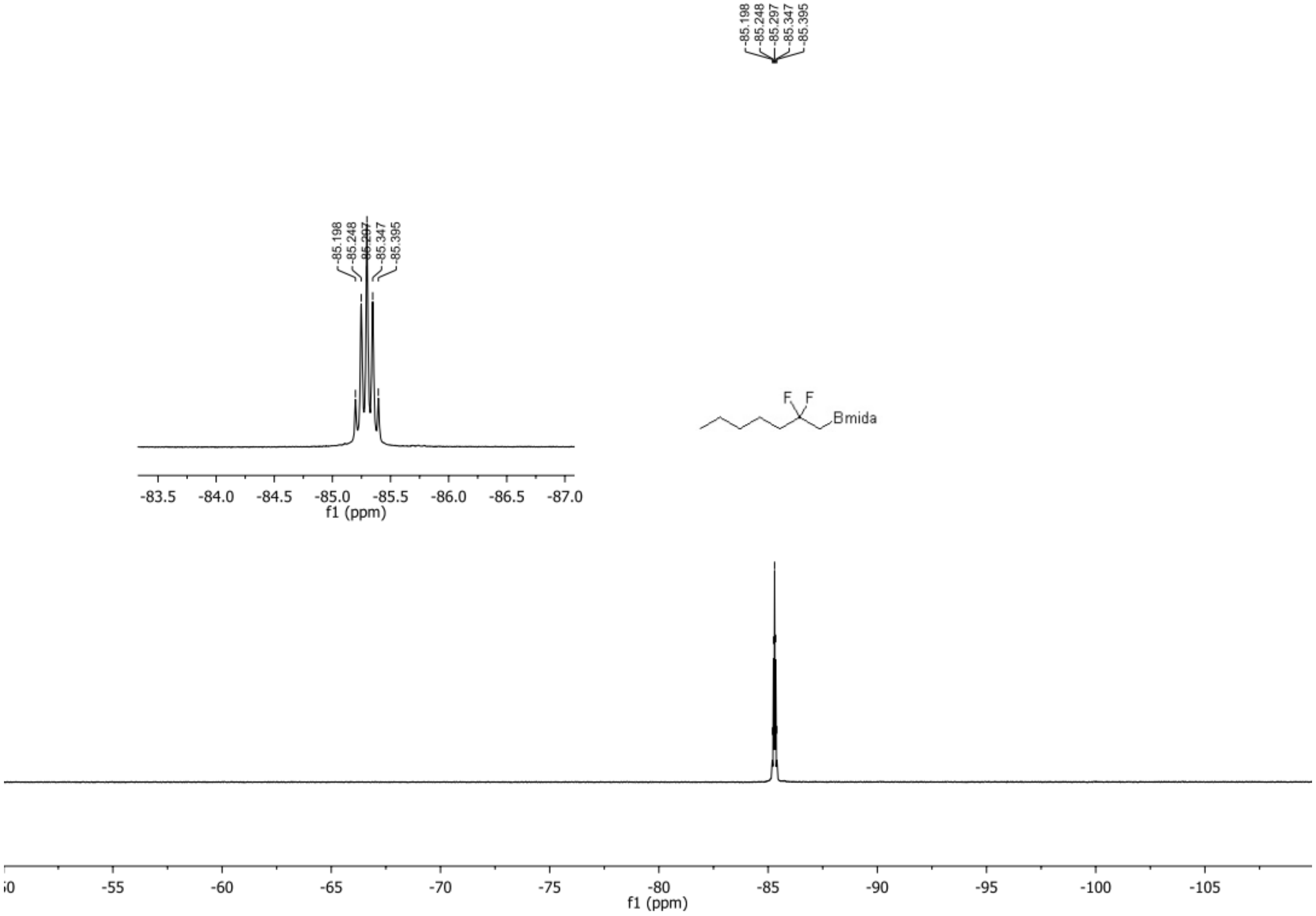

^{11}B NMR ($CDCl_3$, 128 MHz). 2-(2,2-Difluoroheptyl)-6-methyl-1,3,6,2-dioxazaborocane-4,8-dione (**2a**)

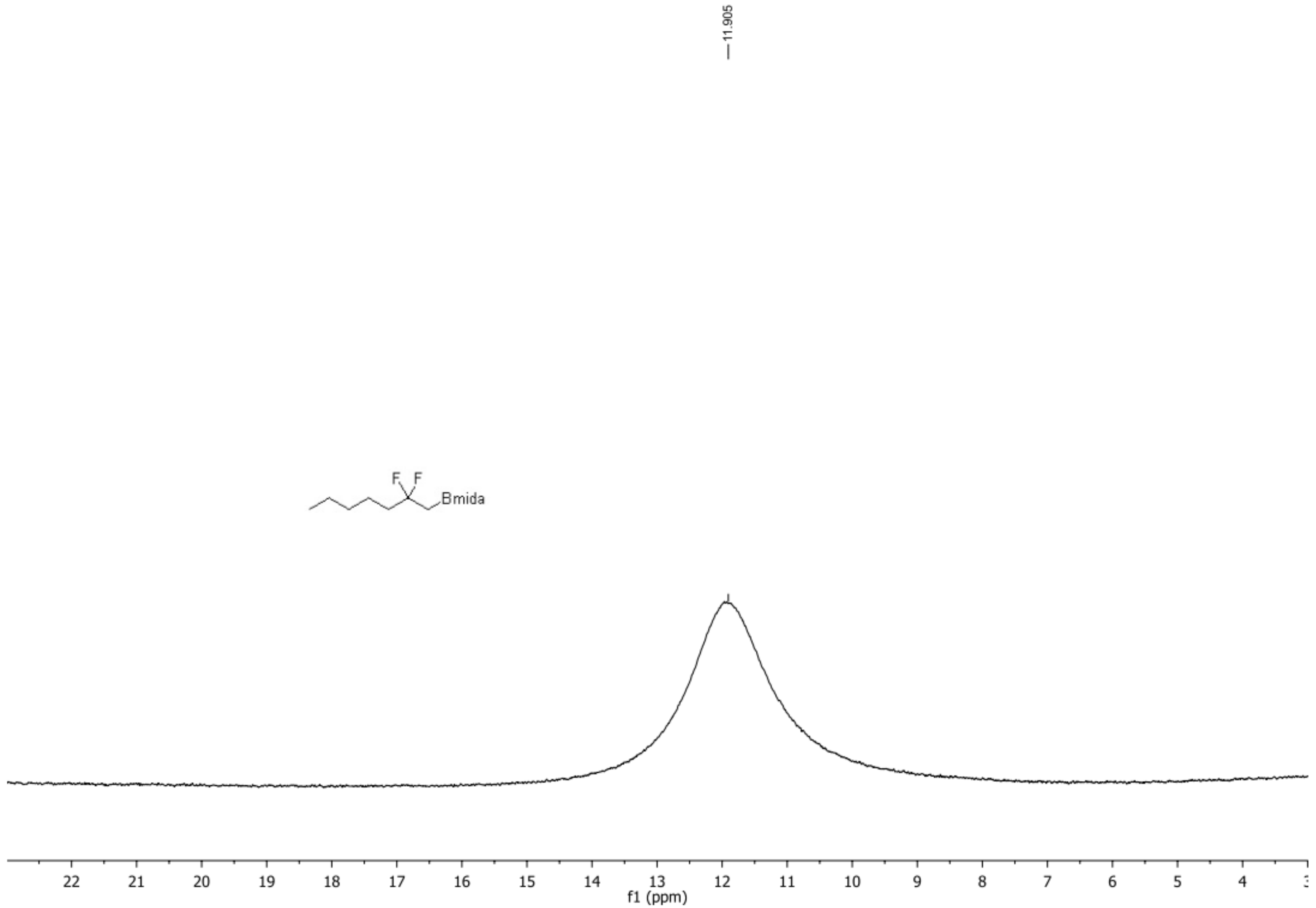

^{1}H NMR ($CDCl_3$, 400 MHz). 2-(2,2-Difluorononyl)-6-methyl-1,3,6,2-dioxazaborocane-4,8-dione (**2b**)

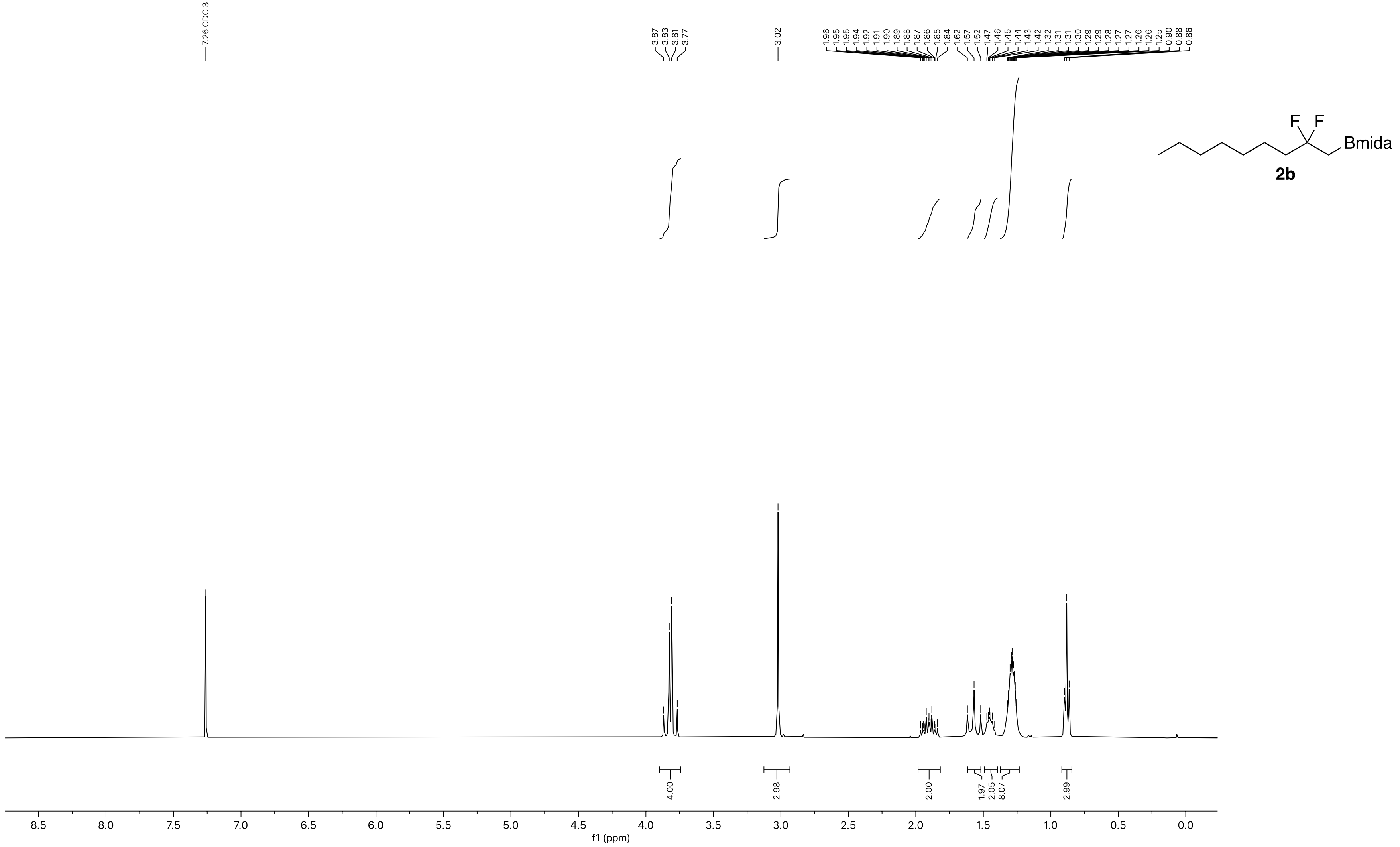

^{13}C NMR ($CDCl_3$, 100 MHz). 2-(2,2-Difluorononyl)-6-methyl-1,3,6,2-dioxazaborocane-4,8-dione (**2b**)

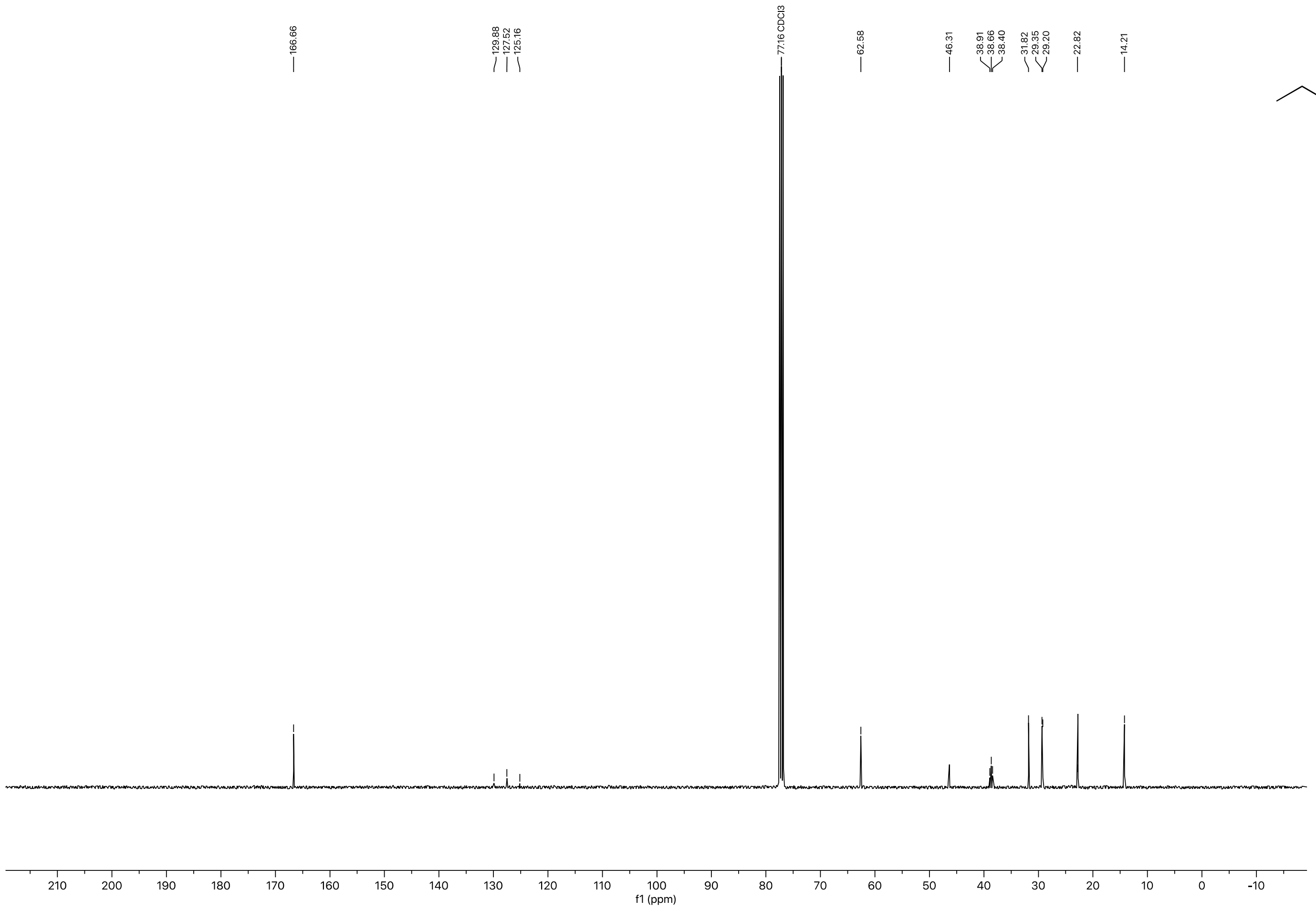

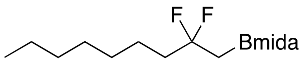

2b

^{19}F NMR ($CDCl_3$, 377 MHz). 2-(2,2-Difluorononyl)-6-methyl-1,3,6,2-dioxazaborocane-4,8-dione (**2b**)

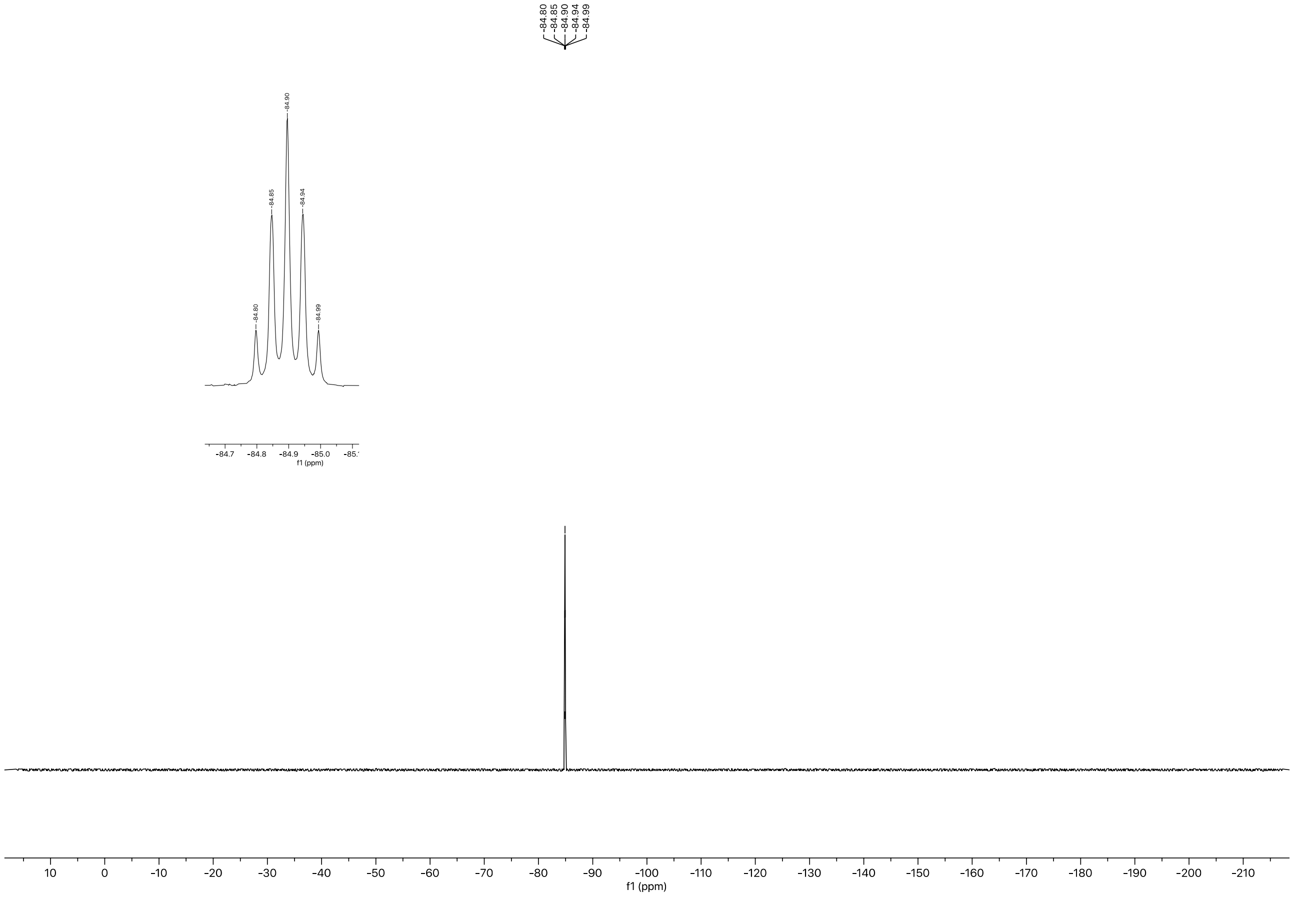

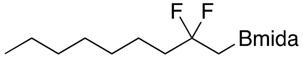

2b

^{11}B NMR ($CDCl_3$, 128 MHz). 2-(2,2-Difluorononyl)-6-methyl-1,3,6,2-dioxazaborocane-4,8-dione (**2b**)

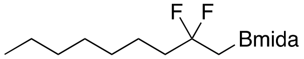

2b

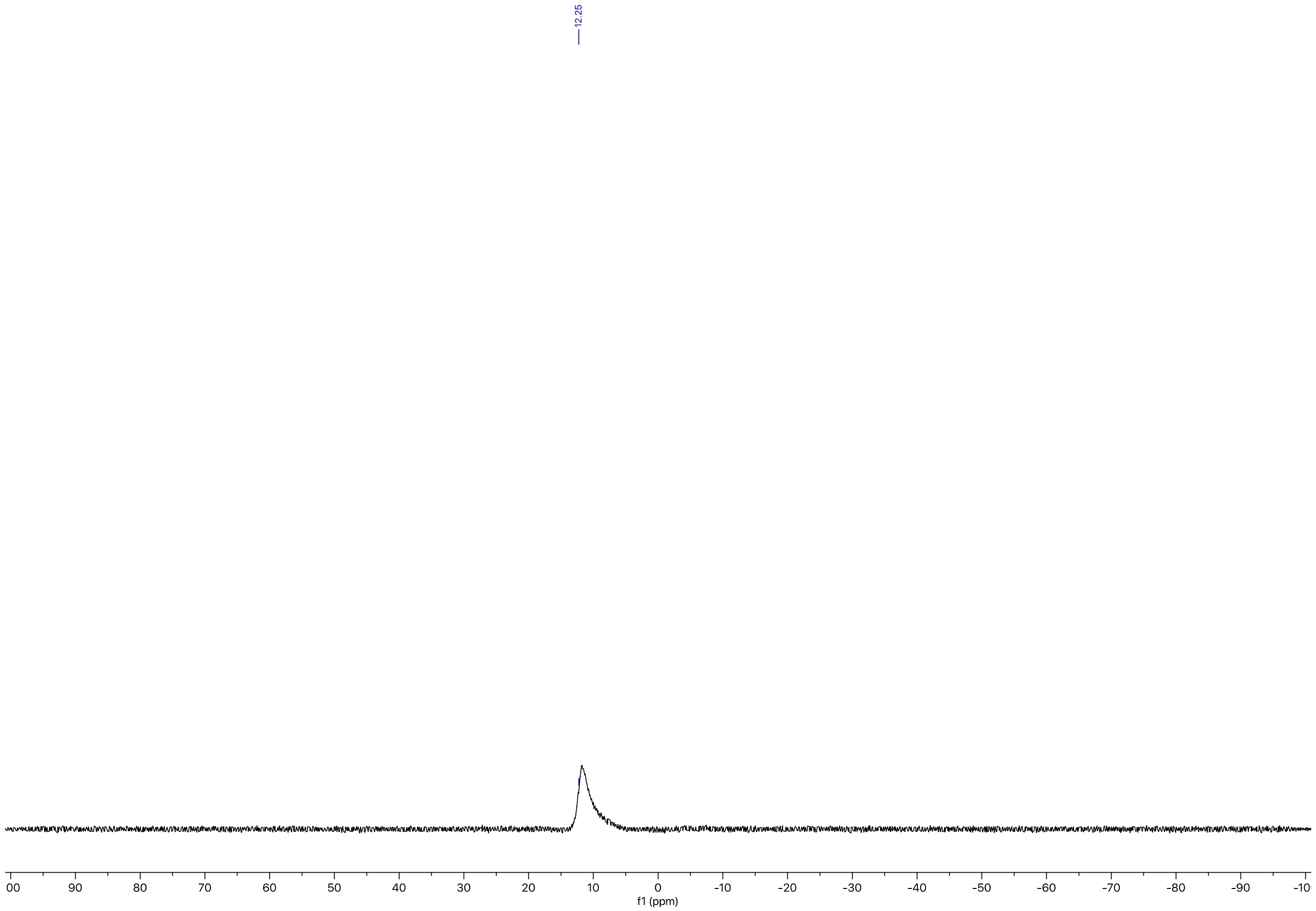

^{1}H NMR ($CDCl_3$, 400 MHz). 2-(2,2-Difluoropentyl)-6-methyl-1,3,6,2-dioxazaborocane-4,8-dione (**2c**)

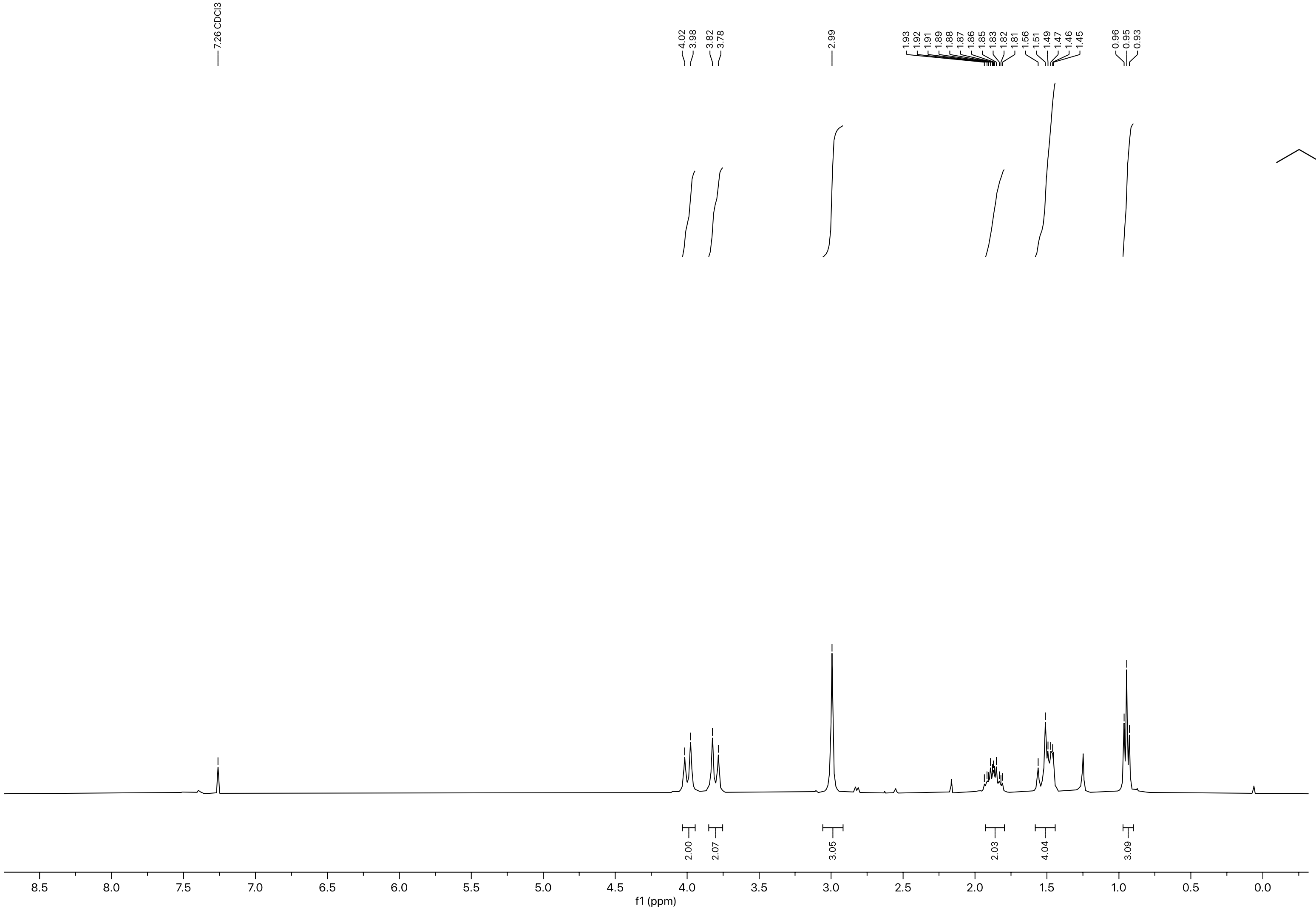

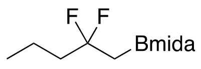

2c

^{13}C NMR (CDCl$_3$, 100 MHz). 2-(2,2-Difluoropentyl)-6-methyl-1,3,6,2-dioxazaborocane-4,8-dione (**2c**)

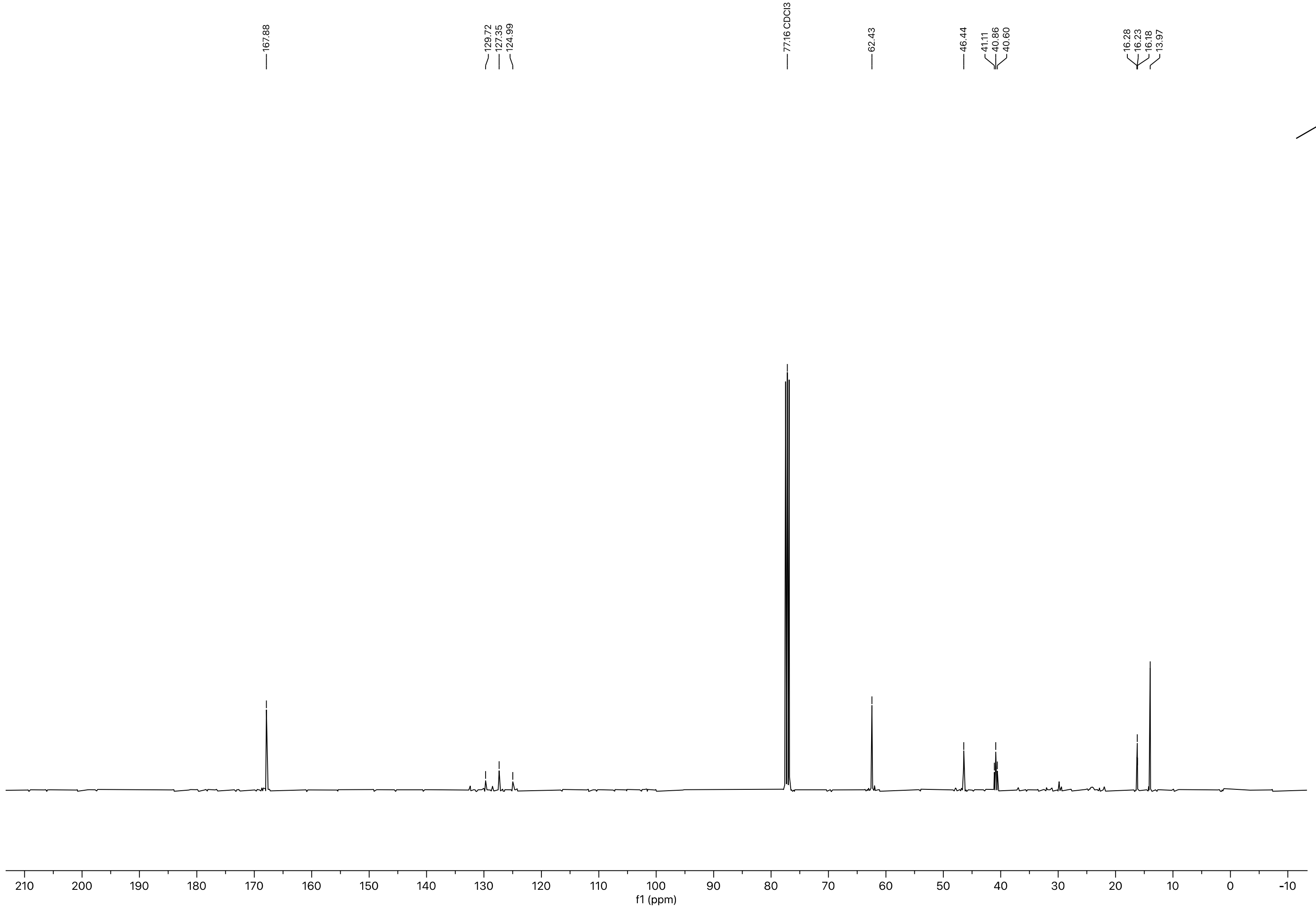

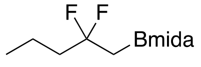

2c

^{19}F NMR ($CDCl_3$, 377 MHz). 2-(2,2-Difluoropentyl)-6-methyl-1,3,6,2-dioxazaborocane-4,8-dione (**2c**)

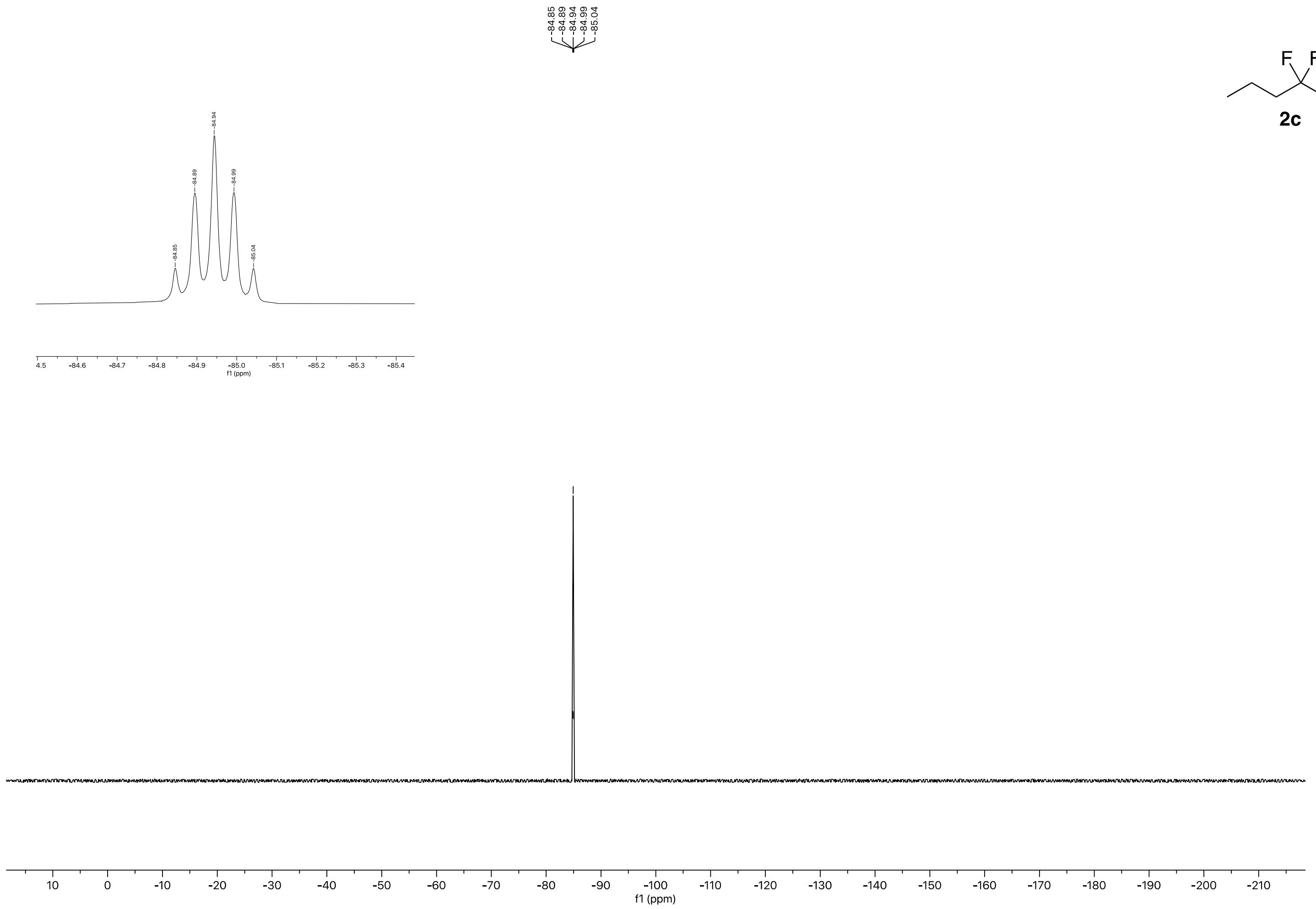

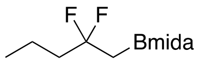

2c

^{11}B NMR ($CDCl_3$, 128 MHz). 2-(2,2-Difluoropentyl)-6-methyl-1,3,6,2-dioxazaborocane-4,8-dione (**2c**)

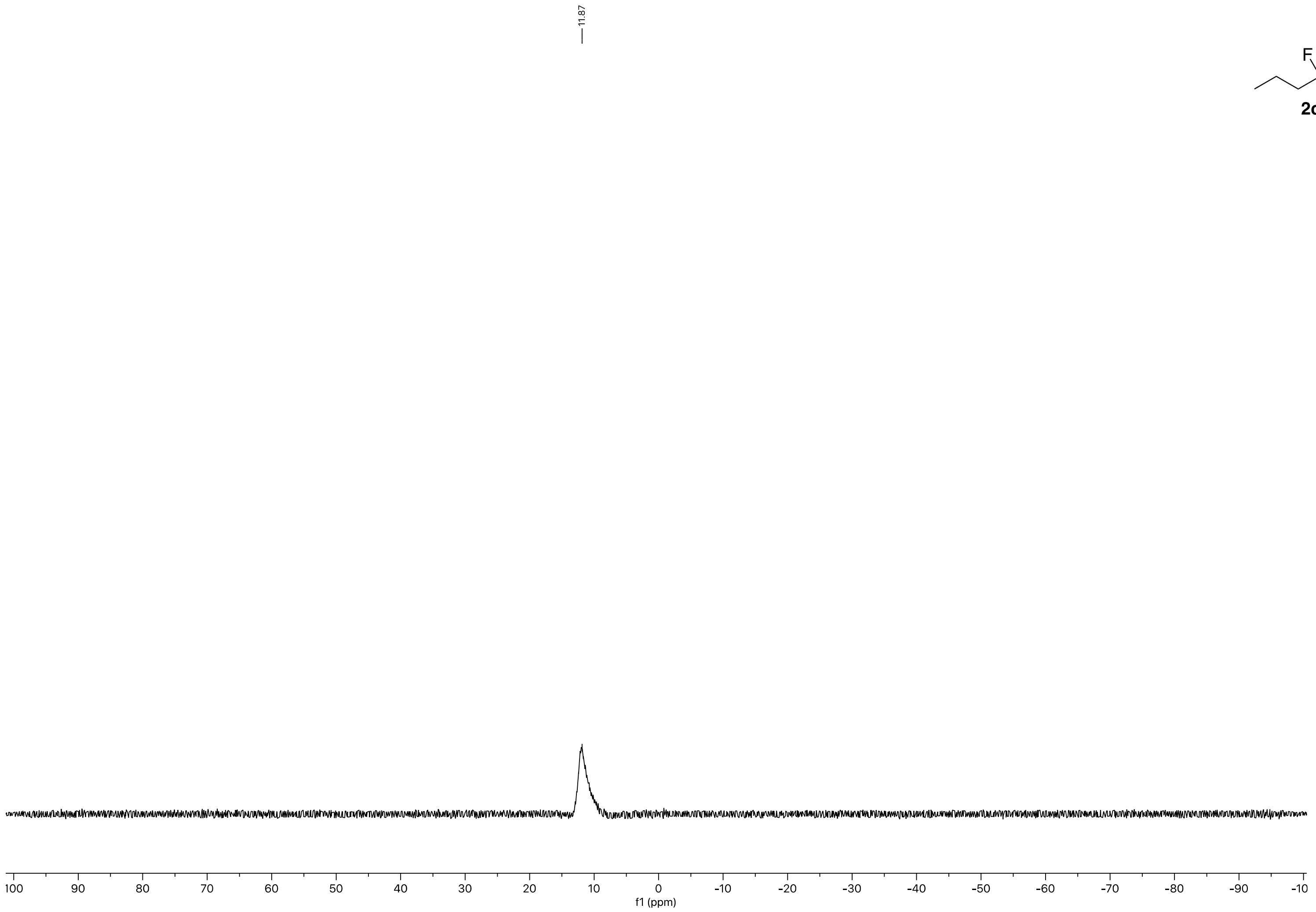

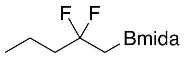

2c

^{1}H NMR ((CD$_3$)$_2$CO, 400 MHz). 2-(2,2-Difluoro-5-methylhexyl)-6-methyl-1,3,6,2-dioxazaborocane-4,8-dione (**2d**)

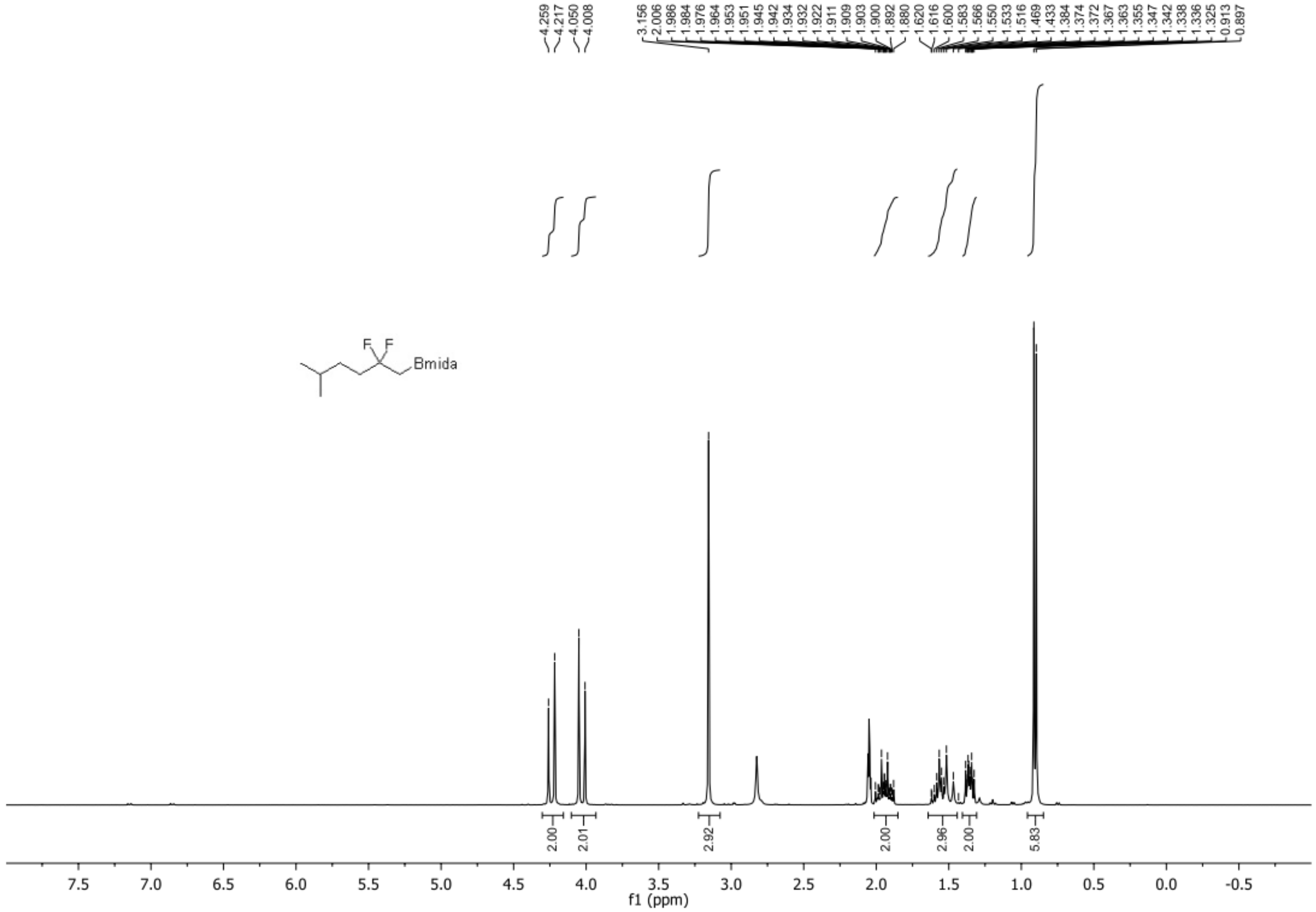

^{13}C NMR ((CD$_3$)$_2$CO, 100 MHz). 2-(2,2-Difluoro-5-methylhexyl)-6-methyl-1,3,6,2-dioxazaborocane-4,8-dione (**2d**)

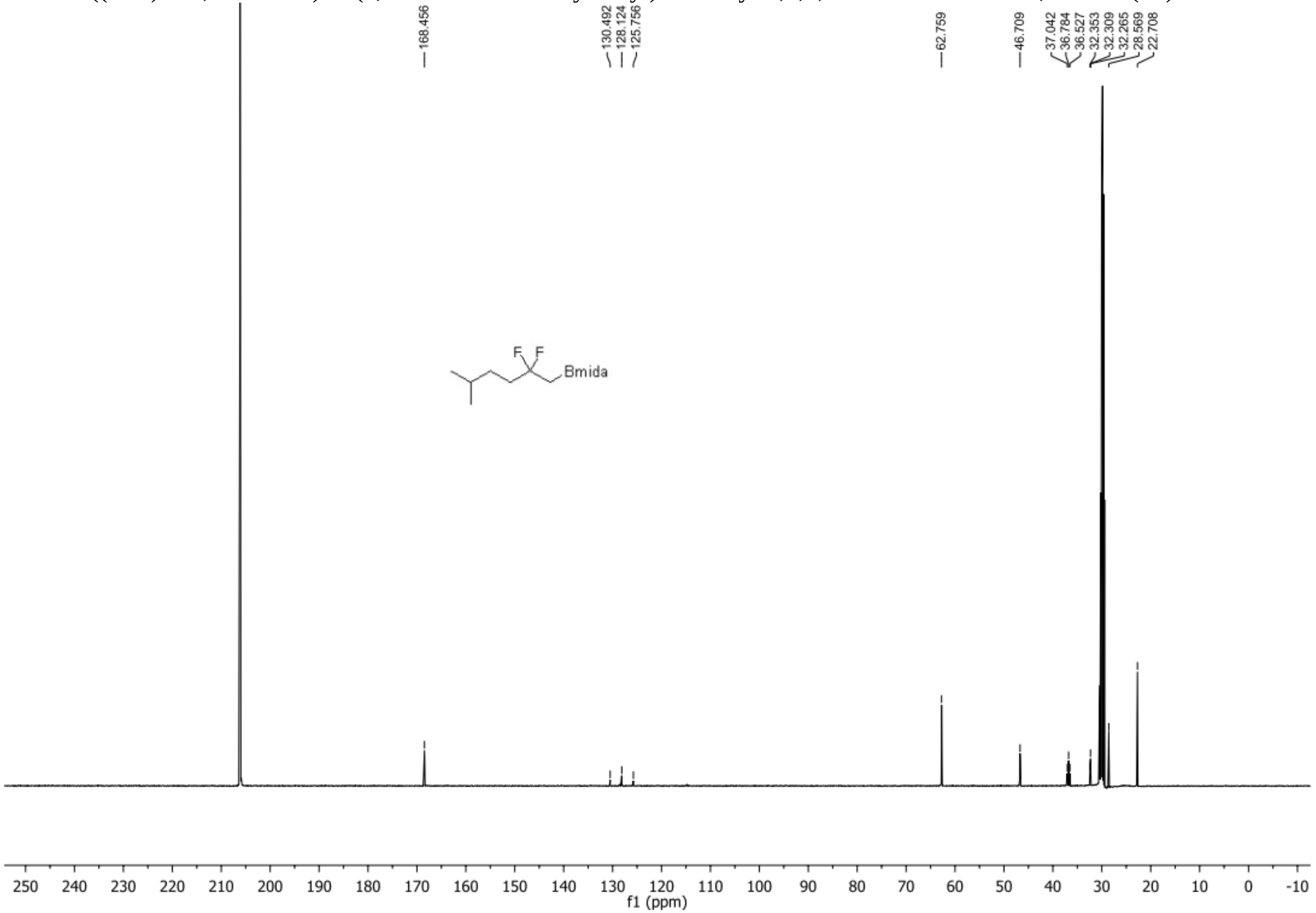

^{19}F NMR (($(CD_3)_2CO$, 377 MHz). 2-(2,2-Difluoro-5-methylhexyl)-6-methyl-1,3,6,2-dioxazaborocane-4,8-dione (**2d**)

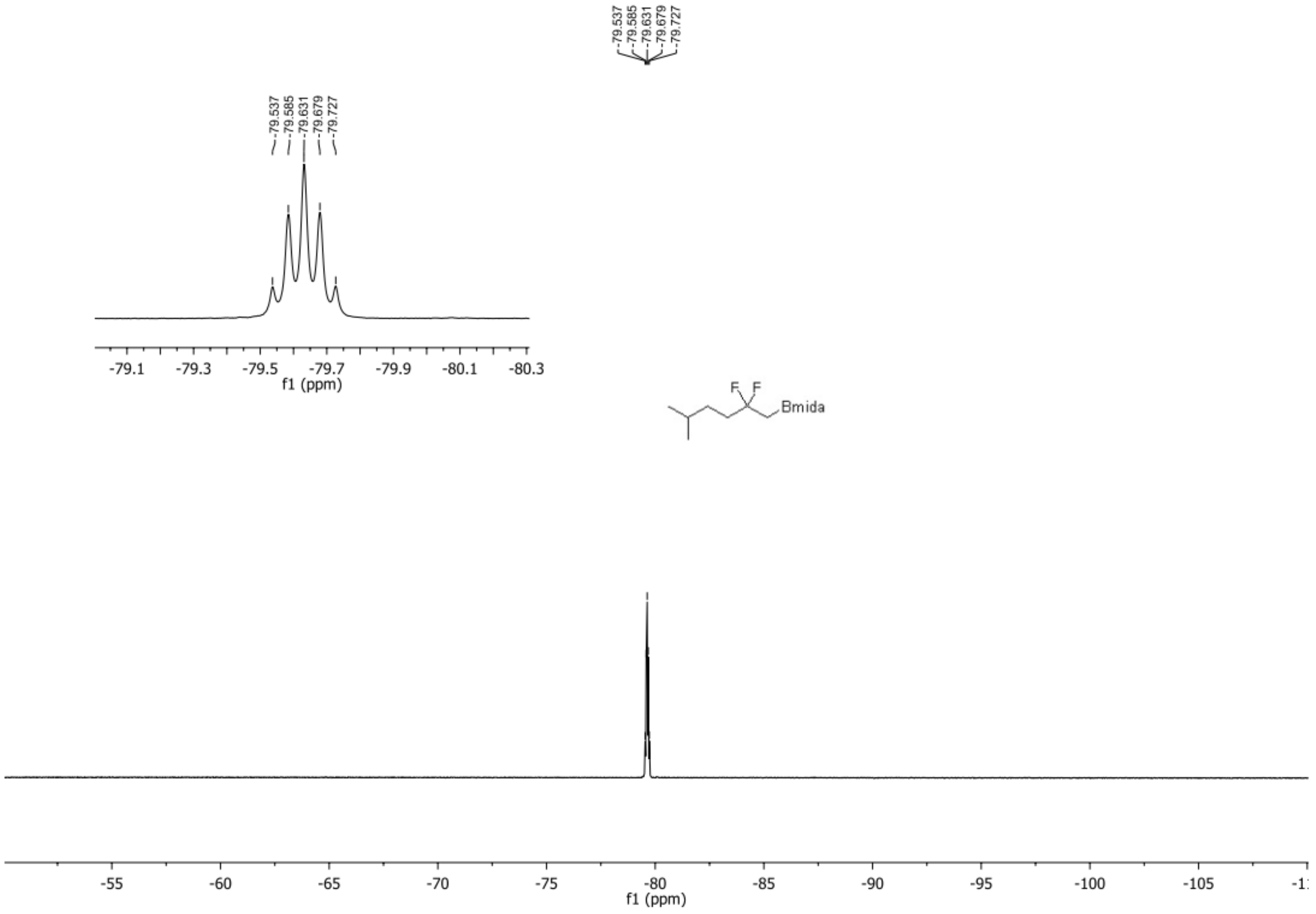

^{11}B NMR (($(CD_3)_2CO$, 128 MHz). 2-(2,2-Difluoro-5-methylhexyl)-6-methyl-1,3,6,2-dioxazaborocane-4,8-dione (**2d**)

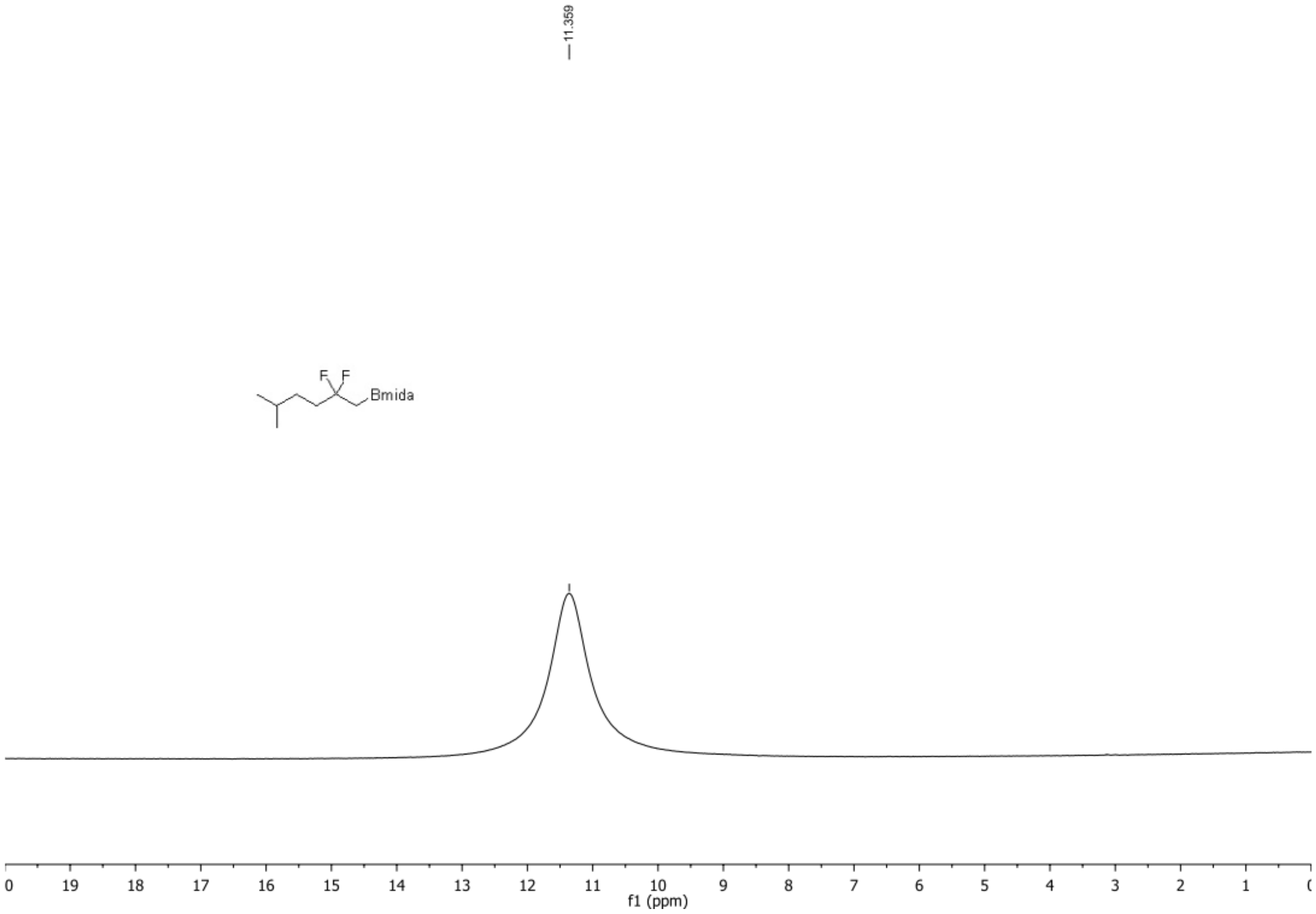

^{1}H NMR ($(CD_3)_2CO$, 400 MHz). 2-(3-Cyclohexyl-2,2-difluoropropyl)-6-methyl-1,3,6,2-dioxazaborocane-4,8-dione (**2e**)

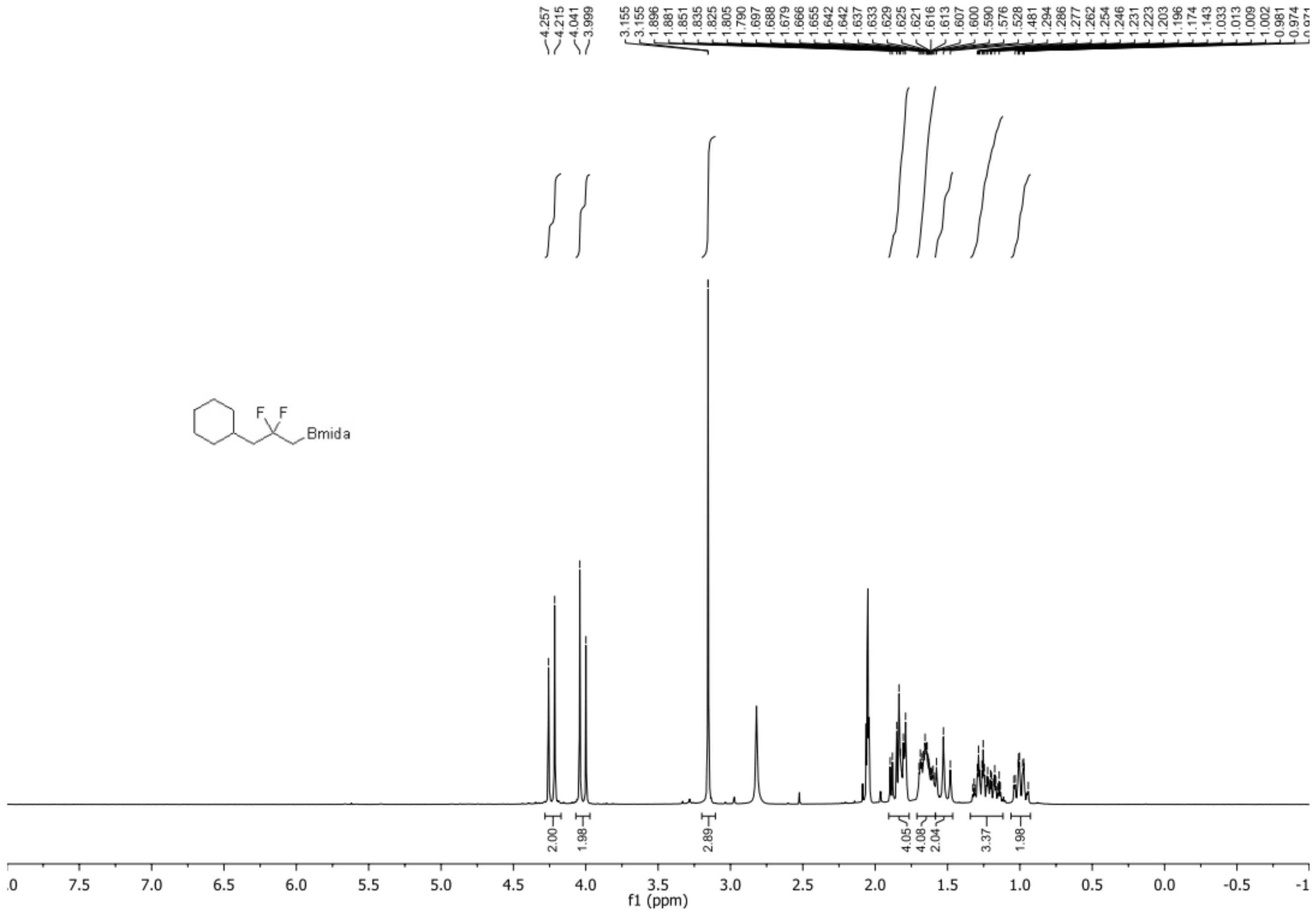

^{13}C NMR ($(CD_3)_2CO$, 100 MHz). 2-(3-Cyclohexyl-2,2-difluoropropyl)-6-methyl-1,3,6,2-dioxazaborocane-4,8-dione (**2e**)

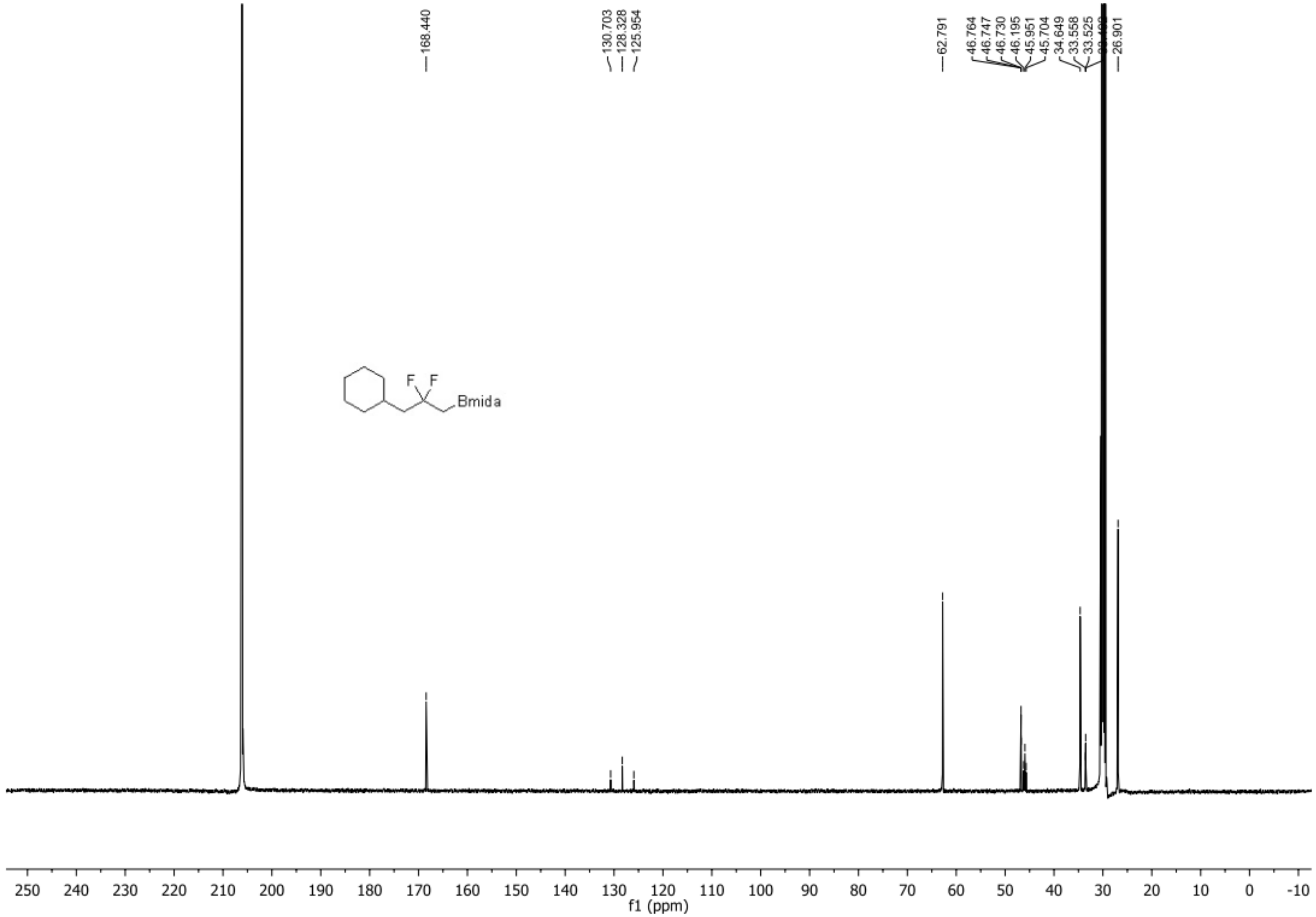

^{19}F NMR ((CD$_3$)$_2$CO, 377 MHz). 2-(3-Cyclohexyl-2,2-difluoropropyl)-6-methyl-1,3,6,2-dioxazaborocane-4,8-dione (**2e**)

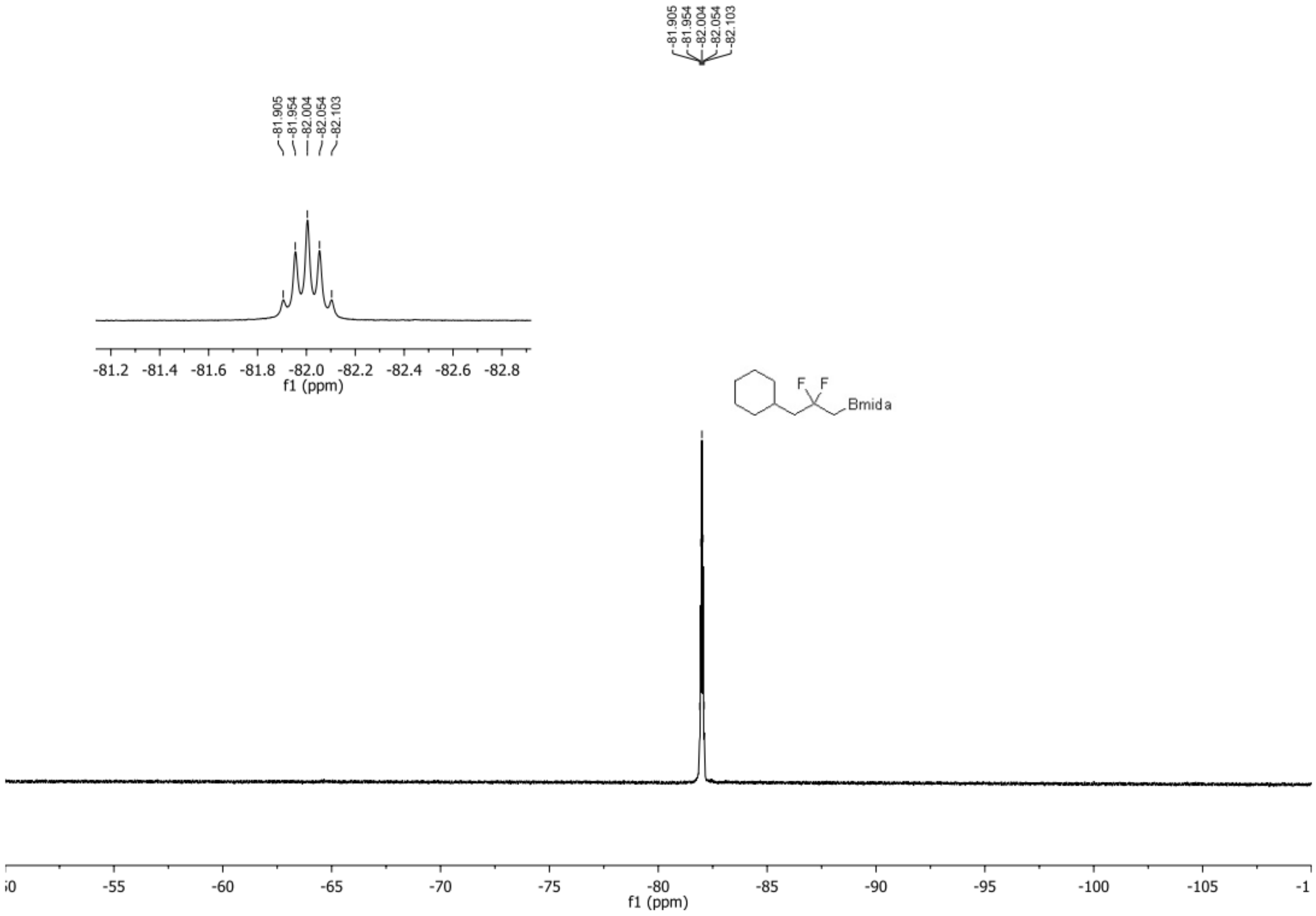

^{11}B NMR ($(CD_3)_2CO$, 128 MHz). 2-(3-Cyclohexyl-2,2-difluoropropyl)-6-methyl-1,3,6,2-dioxazaborocane-4,8-dione (**2e**)

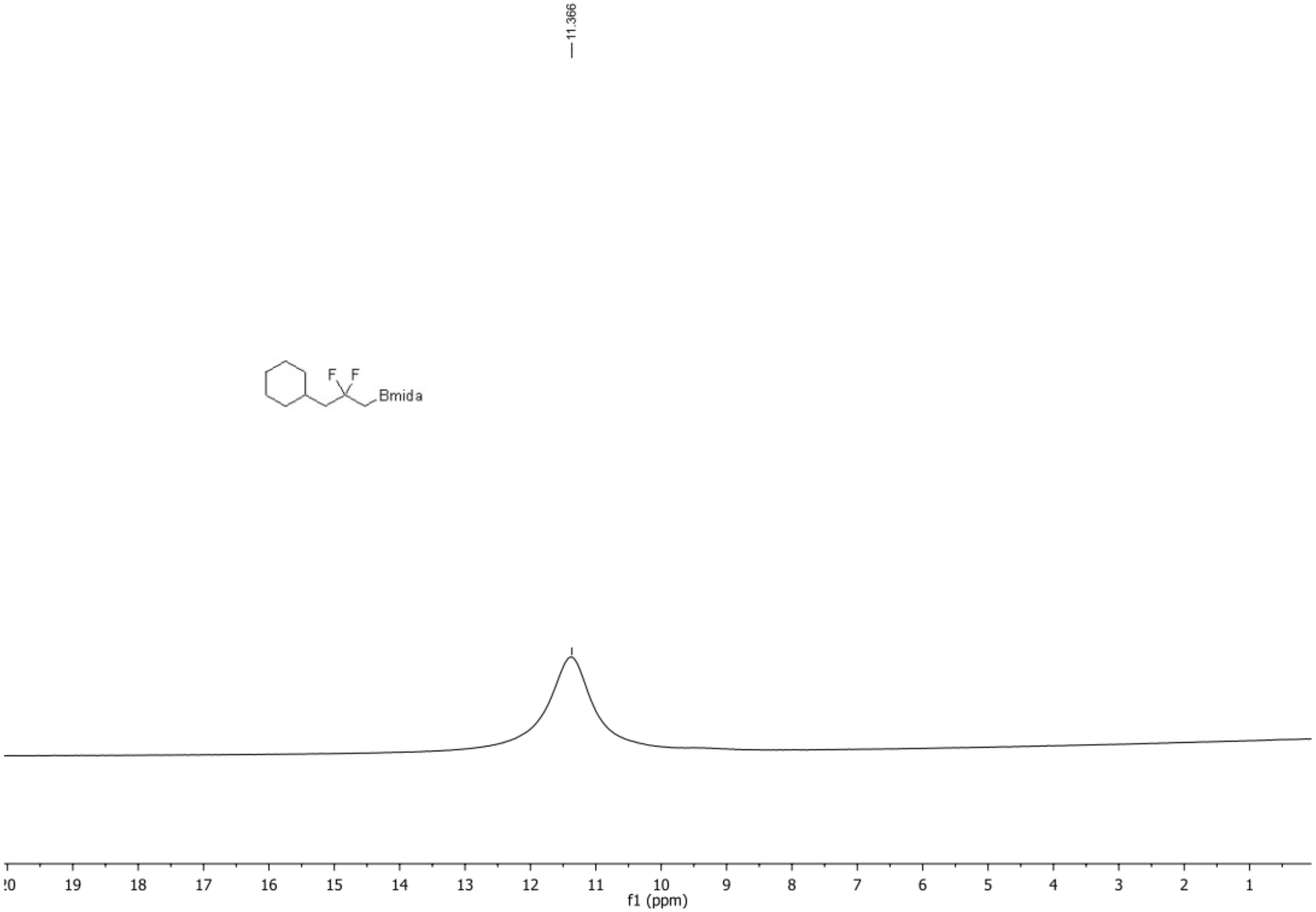

^{1}H NMR ($(CD_3)_2CO$, 400 MHz). 2-(2,2-Difluoro-4-phenylbutyl)-6-methyl-1,3,6,2-dioxazaborocane-4,8-dione (**2f**)

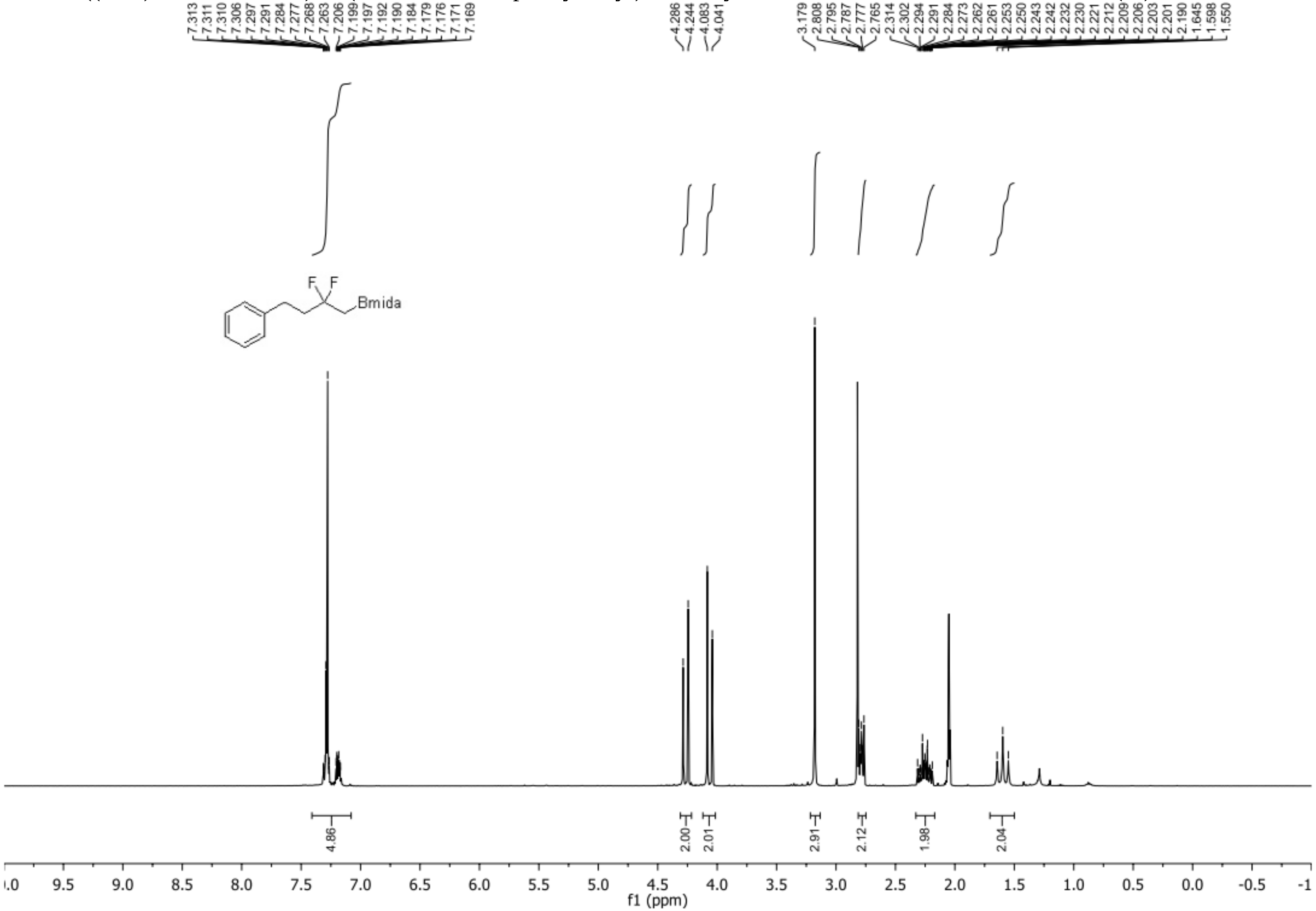

^{13}C NMR ($(CD_3)_2CO$, 100 MHz). 2-(2,2-Difluoro-4-phenylbutyl)-6-methyl-1,3,6,2-dioxazaborocane-4,8-dione (**2f**)

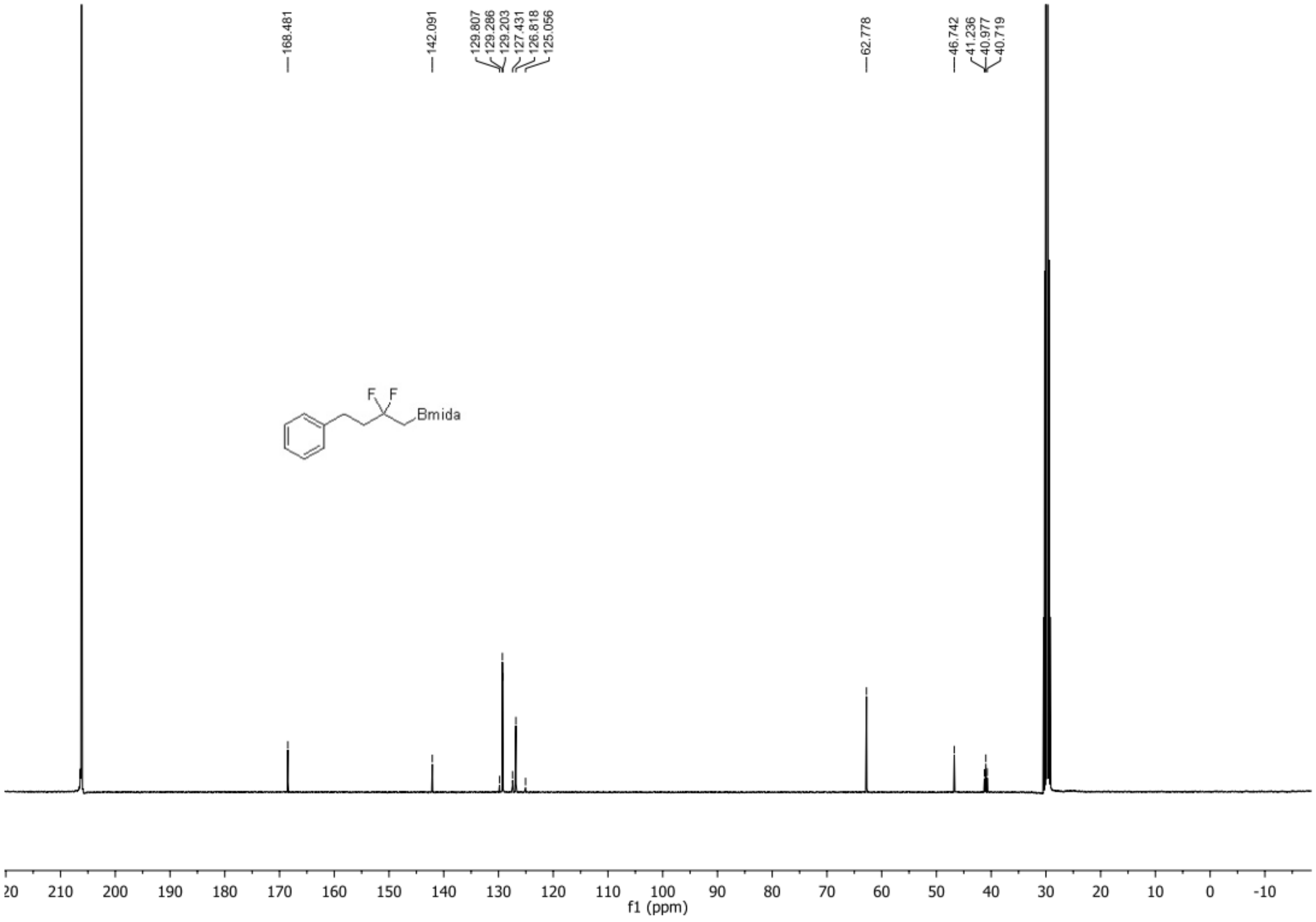

^{19}F NMR ($(CD_3)_2CO$, 377 MHz). 2-(2,2-Difluoro-4-phenylbutyl)-6-methyl-1,3,6,2-dioxazaborocane-4,8-dione (**2f**)

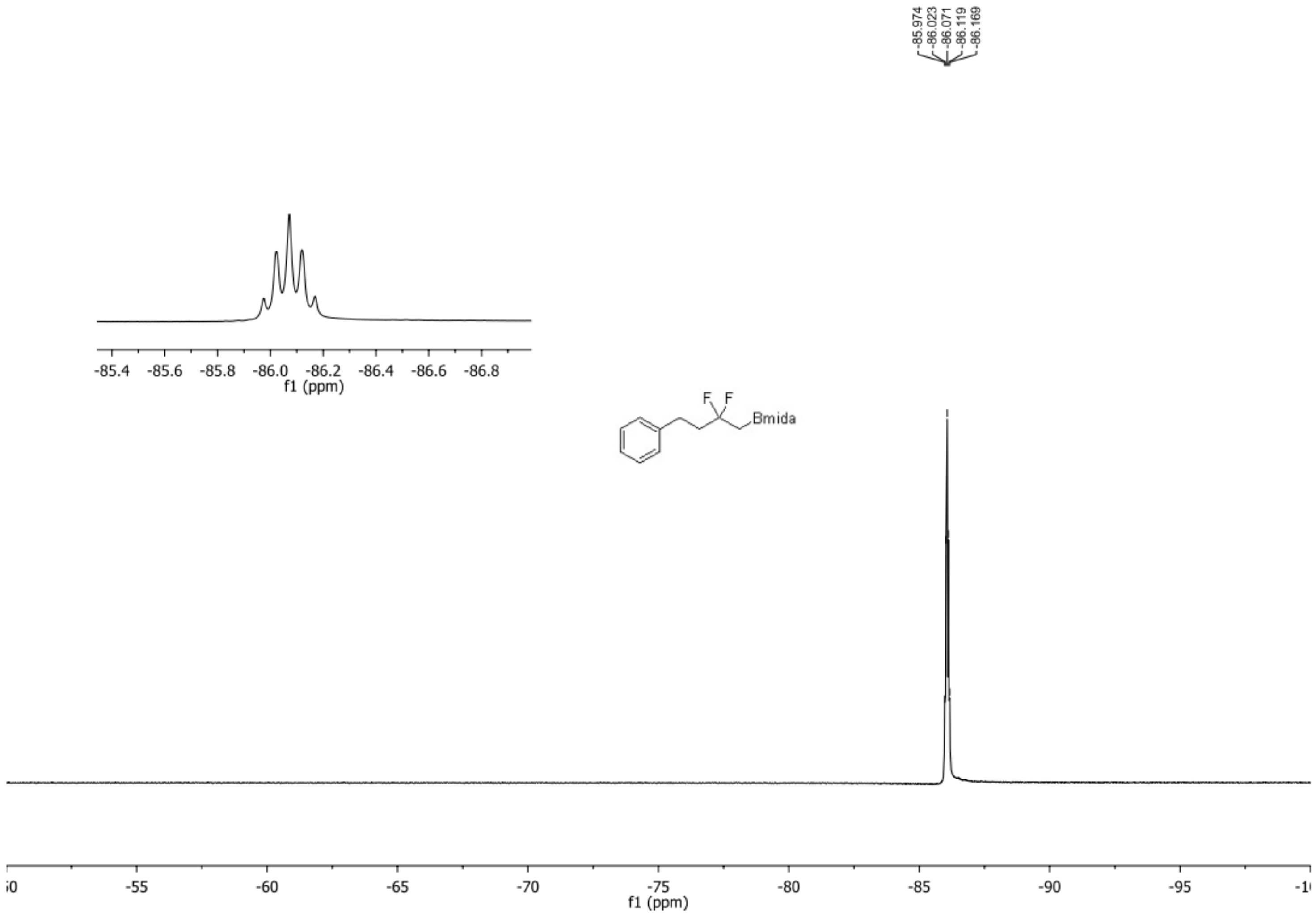

^{11}B NMR ($(CD_3)_2CO$, 128 MHz). 2-(2,2-Difluoro-4-phenylbutyl)-6-methyl-1,3,6,2-dioxazaborocane-4,8-dione (**2f**)

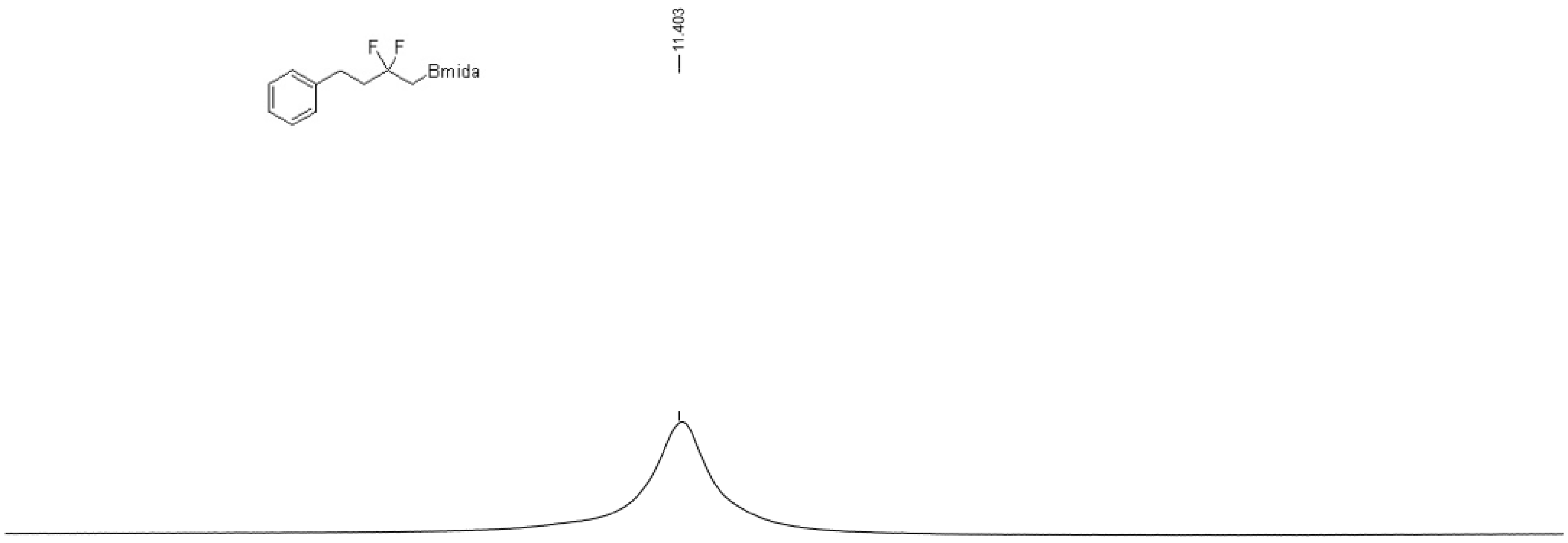

^{1}H NMR ($CDCl_3$, 400 MHz). 2-(5-Chloro-2,2-difluoropentyl)-6-methyl-1,3,6,2-dioxazaborocane-4,8-dione (**2g**)

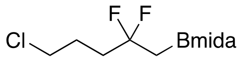

2g

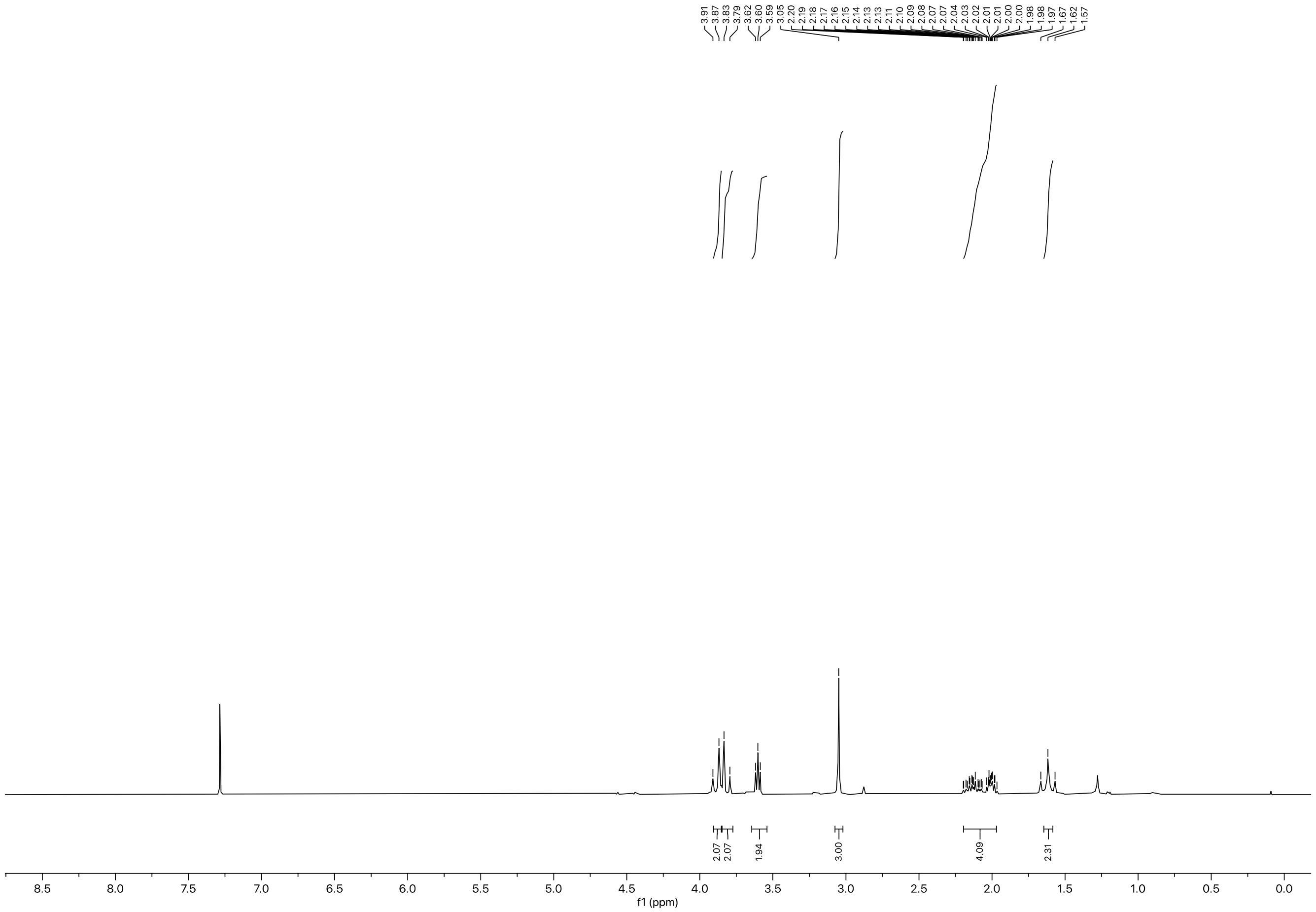

^{13}C NMR ($CDCl_3$, 100 MHz). 2-(5-Chloro-2,2-difluoropentyl)-6-methyl-1,3,6,2-dioxazaborocane-4,8-dione (**2g**)

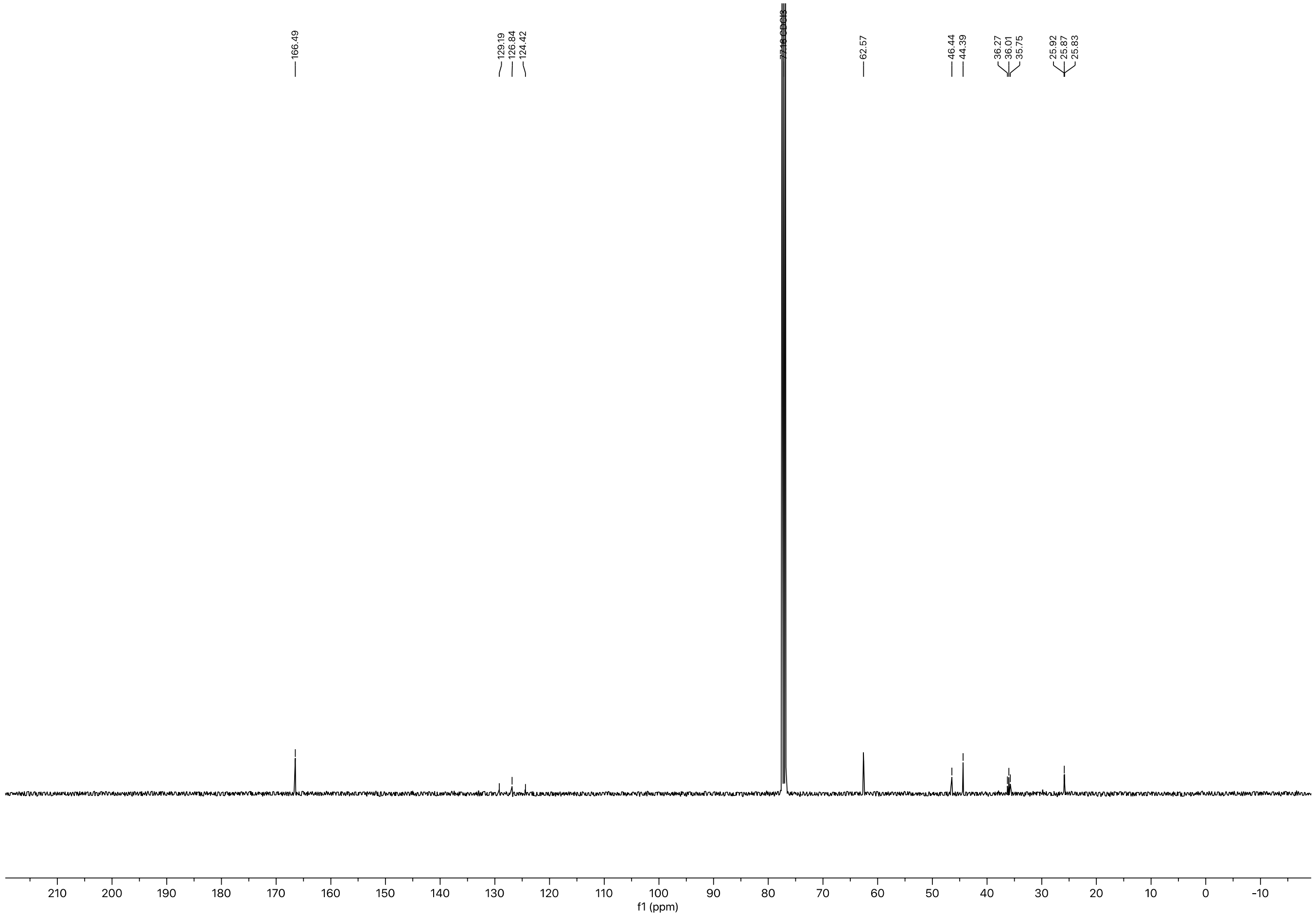

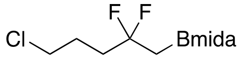

2g

^{19}F NMR ($CDCl_3$, 377 MHz). 2-(5-Chloro-2,2-difluoropentyl)-6-methyl-1,3,6,2-dioxazaborocane-4,8-dione (**2g**)

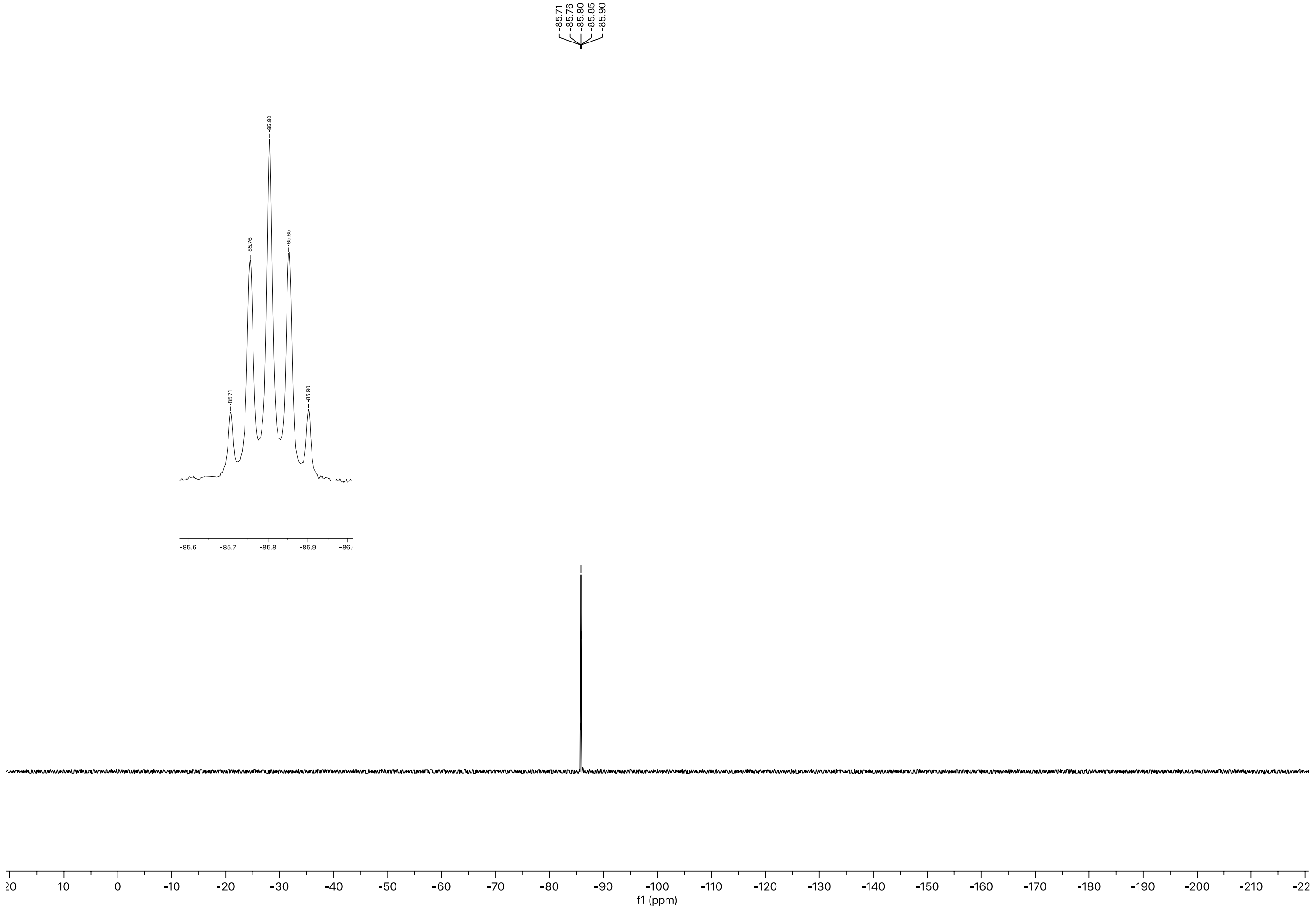

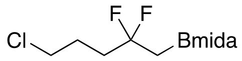

2g

^{11}B NMR ($CDCl_3$, 128 MHz). 2-(5-Chloro-2,2-difluoropentyl)-6-methyl-1,3,6,2-dioxazaborocane-4,8-dione (**2g**)

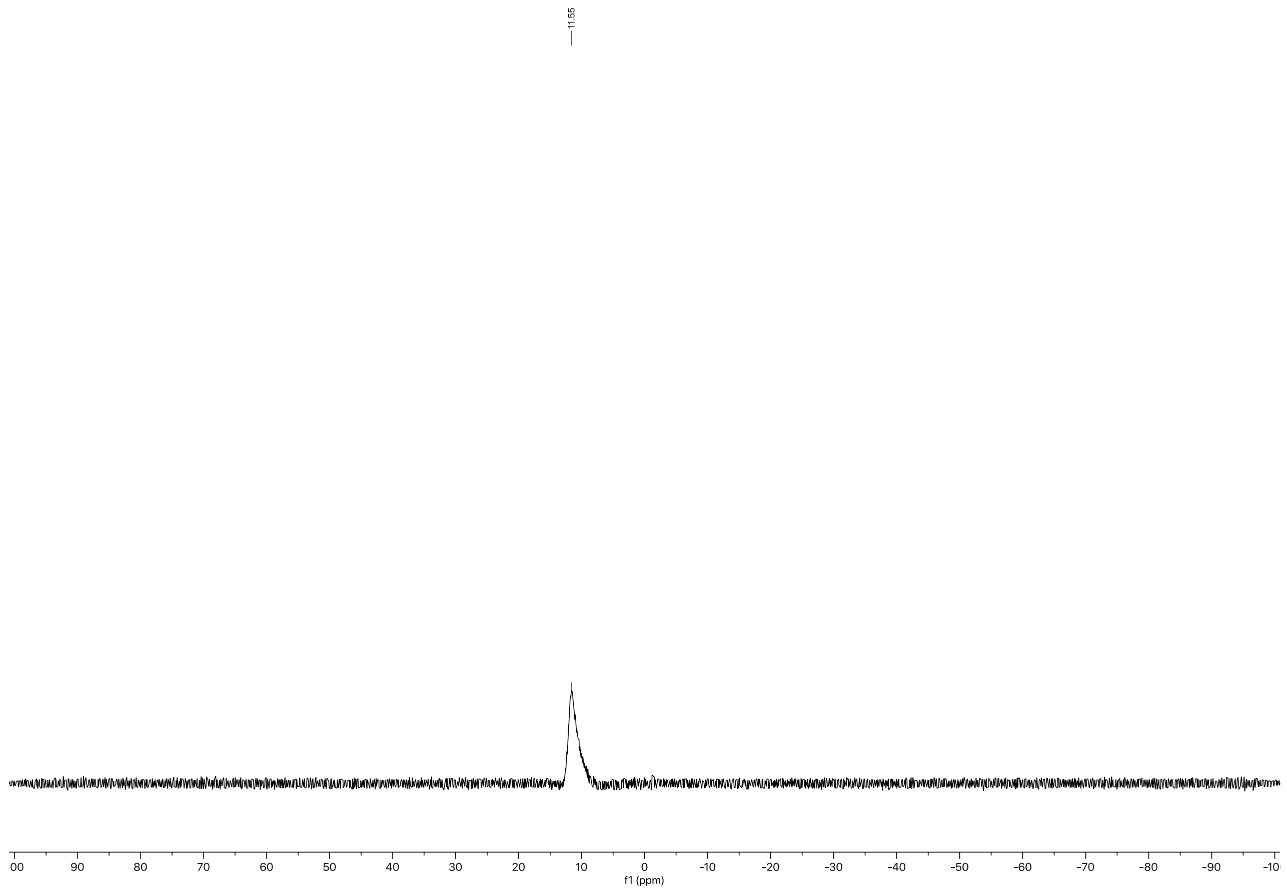

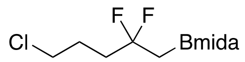

2g

^{1}H NMR ($CDCl_3$, 400 MHz). 2-(2,2-Difluoro-3-phenylpropyl)-6-methyl-1,3,6,2-dioxazaborocane-4,8-dione (**2h**)

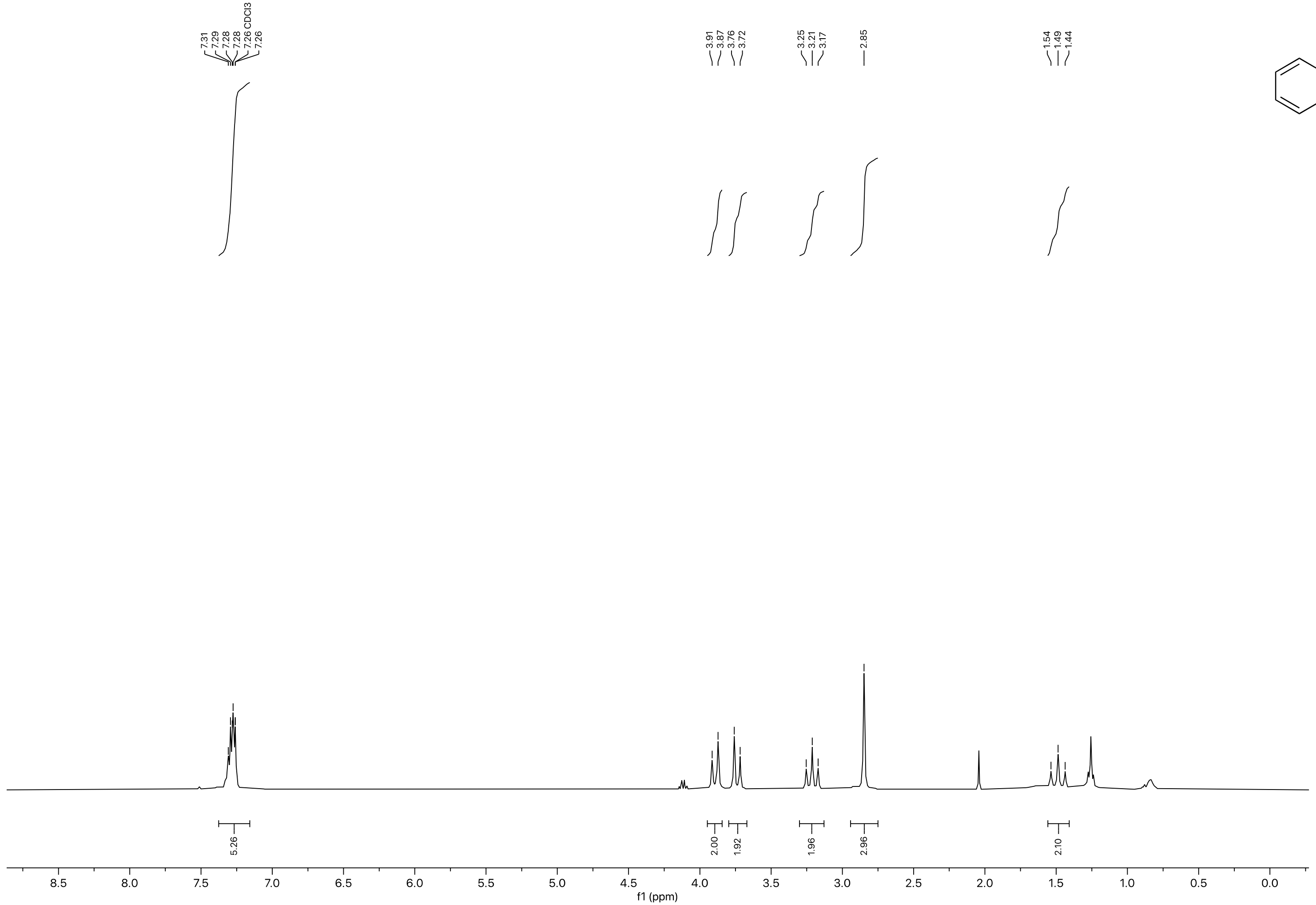

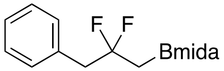

2h

^{13}C NMR ($CDCl_3$, 100 MHz). 2-(2,2-Difluoro-3-phenylpropyl)-6-methyl-1,3,6,2-dioxazaborocane-4,8-dione (**2h**)

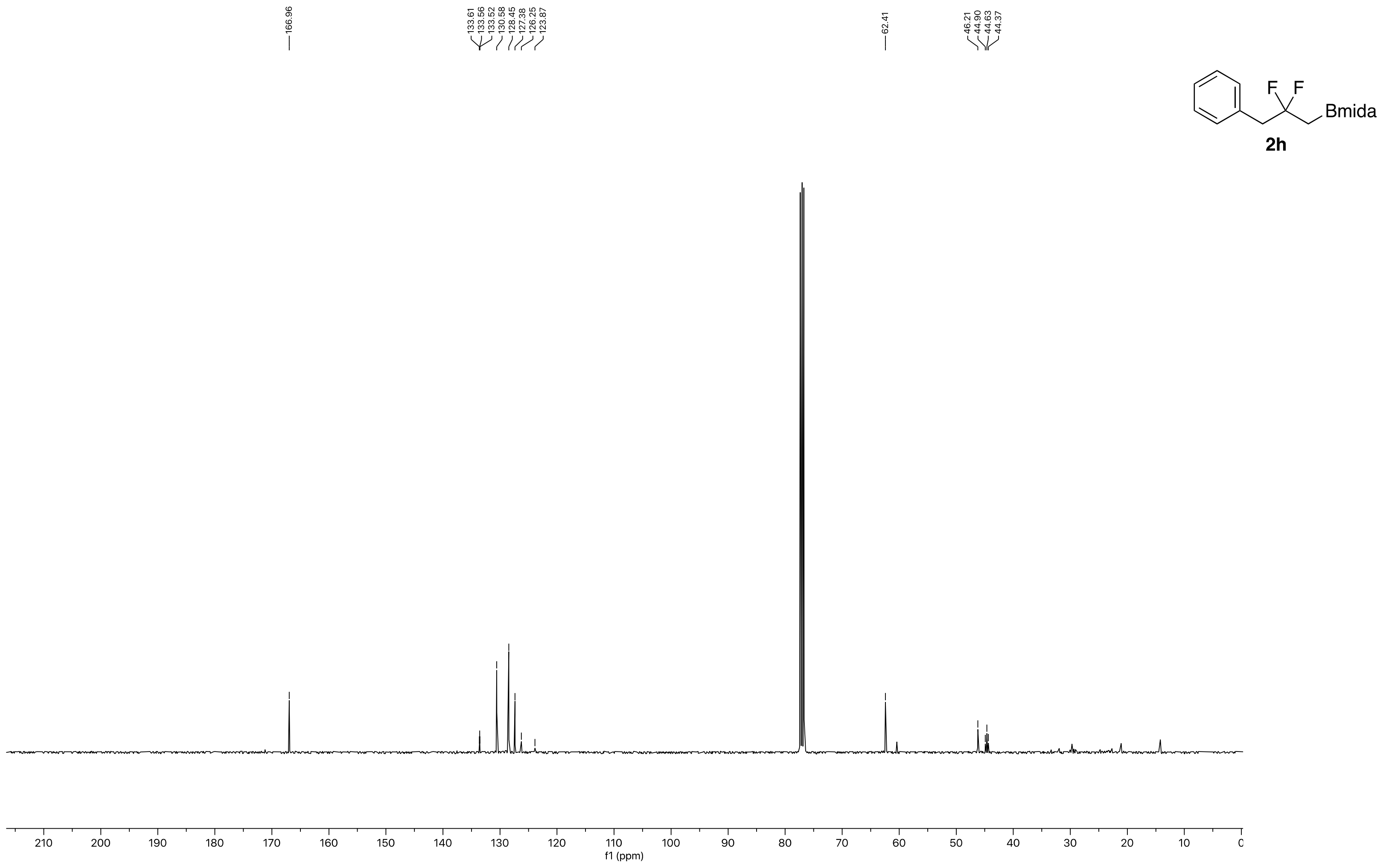

^{19}F NMR ($CDCl_3$, 377 MHz). 2-(2,2-Difluoro-3-phenylpropyl)-6-methyl-1,3,6,2-dioxazaborocane-4,8-dione (**2h**)

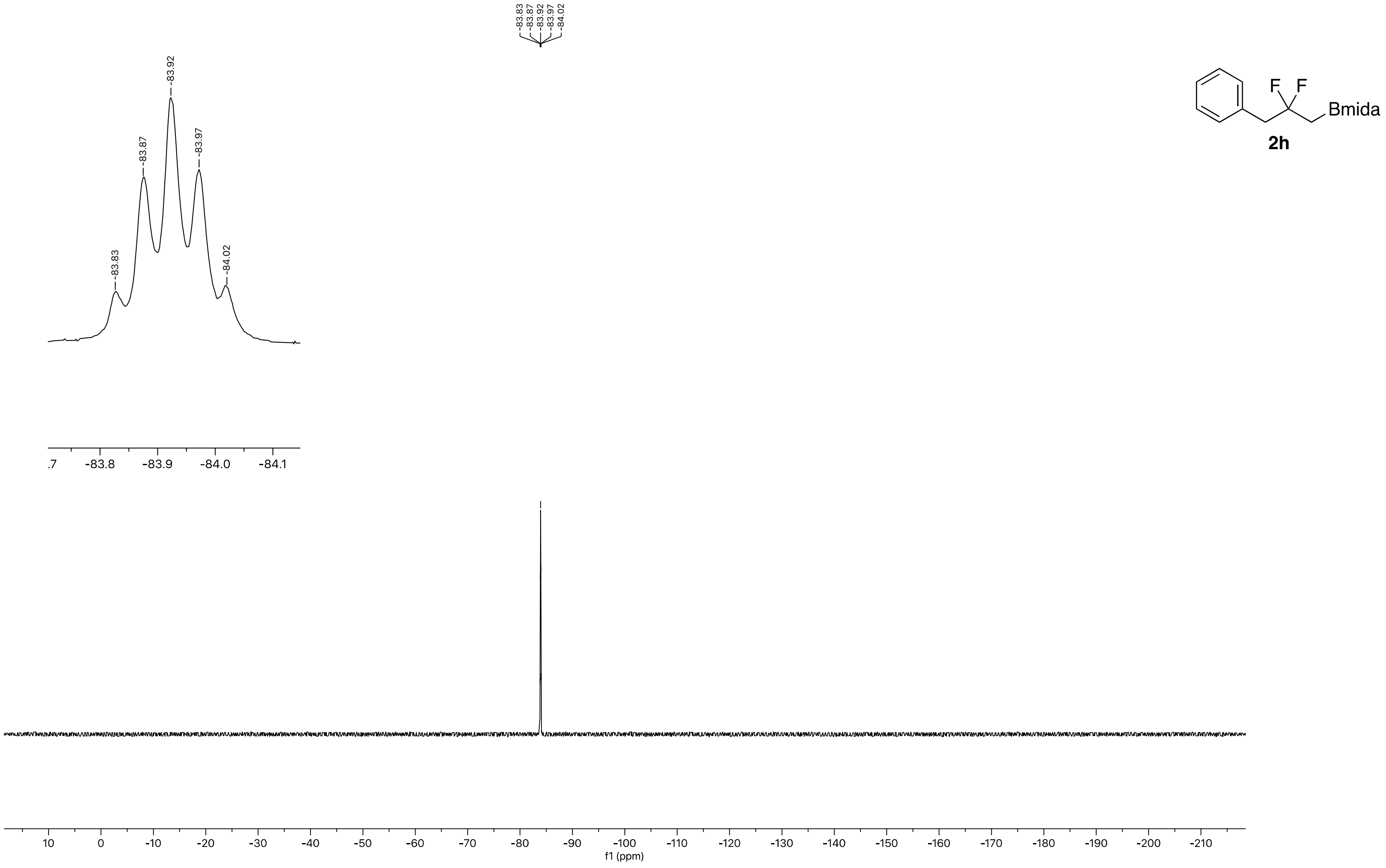

^{11}B NMR ($CDCl_3$, 128 MHz). 2-(2,2-Difluoro-3-phenylpropyl)-6-methyl-1,3,6,2-dioxazaborocane-4,8-dione (**2h**)

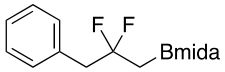

2h

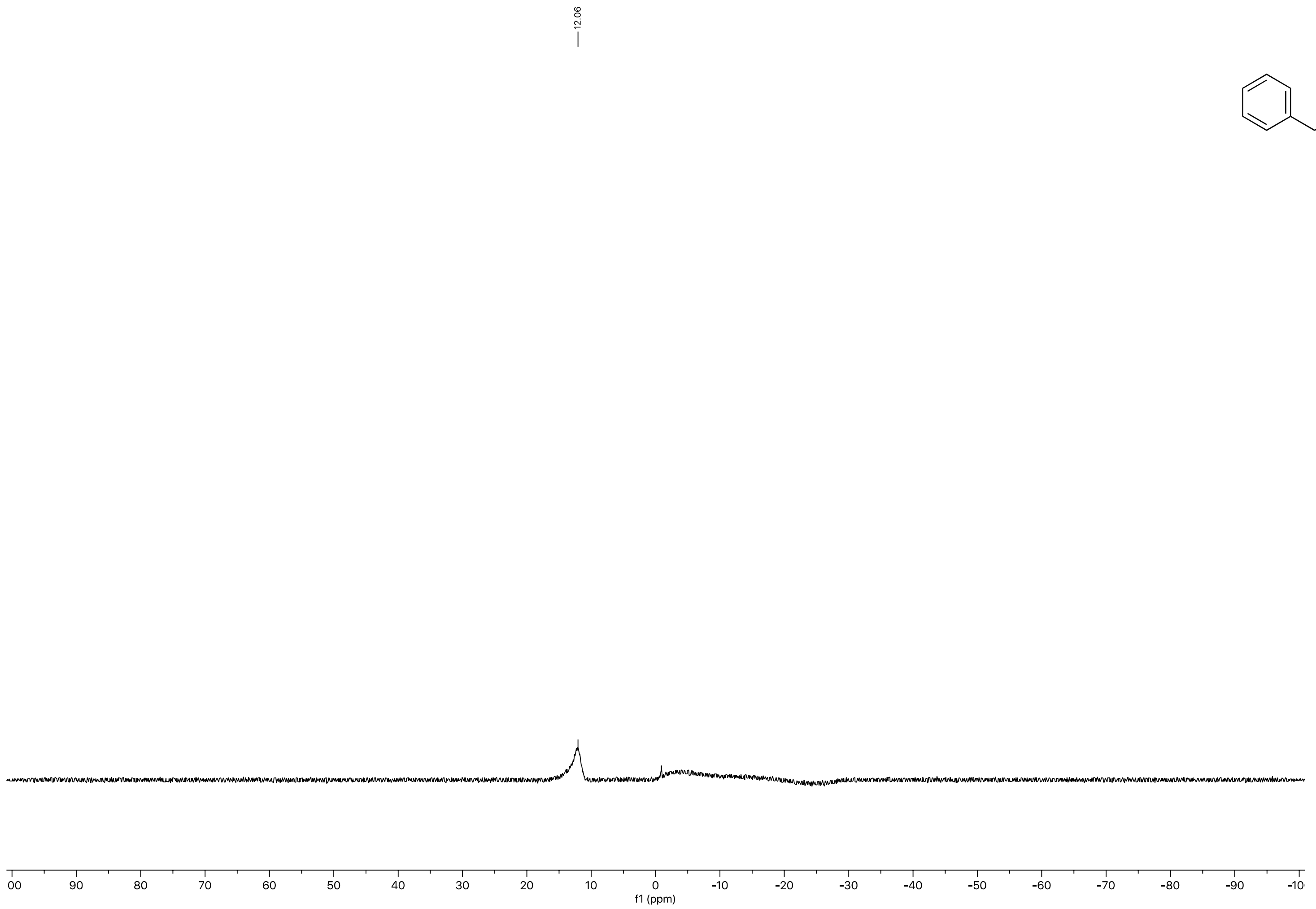

^{1}H NMR (($(CD_3)_2CO$, 400 MHz). 2-(2,2-Difluoro-3-(p-tolyl)propyl)-6-methyl-1,3,6,2-dioxazaborocane-4,8-dione (**2i**)

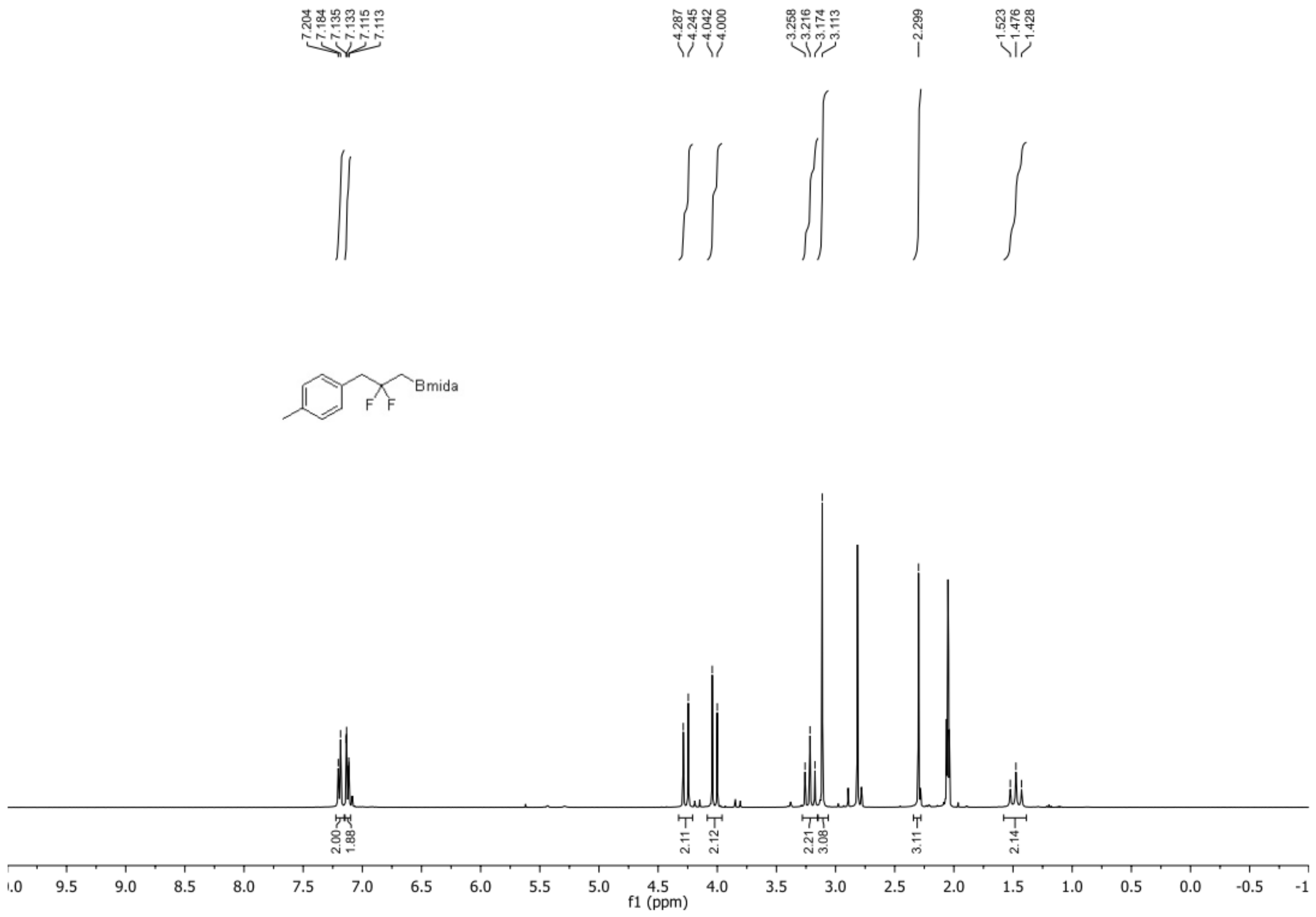

^{13}C NMR ($(CD_3)_2CO$, 100 MHz). 2-(2,2-Difluoro-3-(p-tolyl)propyl)-6-methyl-1,3,6,2-dioxazaborocane-4,8-dione (**2i**)

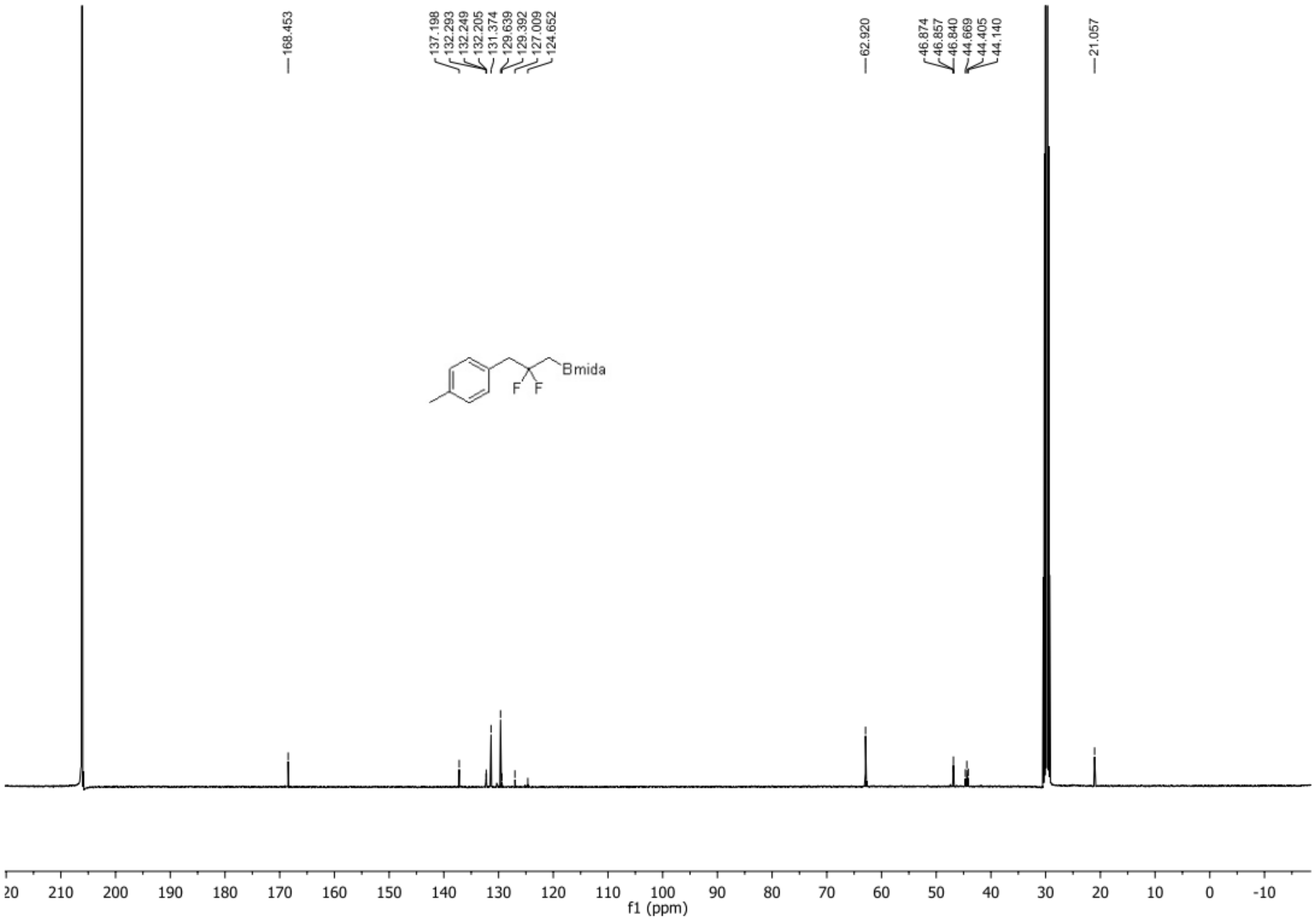

^{19}F NMR ($(CD_3)_2CO$, 377 MHz). 2-(2,2-Difluoro-3-(p-tolyl)propyl)-6-methyl-1,3,6,2-dioxazaborocane-4,8-dione (**2i**)

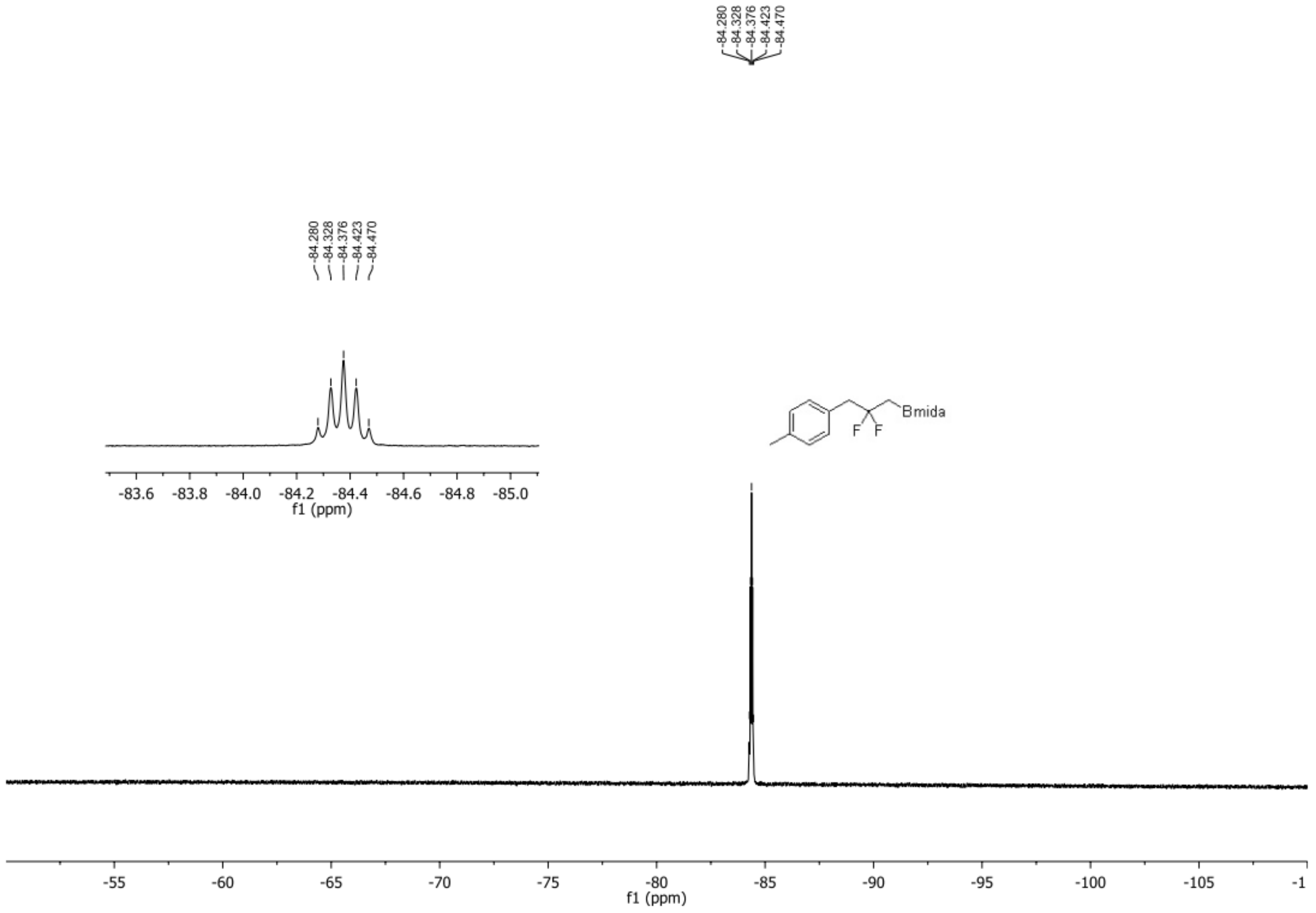

^{11}B NMR ($(CD_3)_2CO$, 128 MHz). 2-(2,2-Difluoro-3-(p-tolyl)propyl)-6-methyl-1,3,6,2-dioxazaborocane-4,8-dione (**2i**)

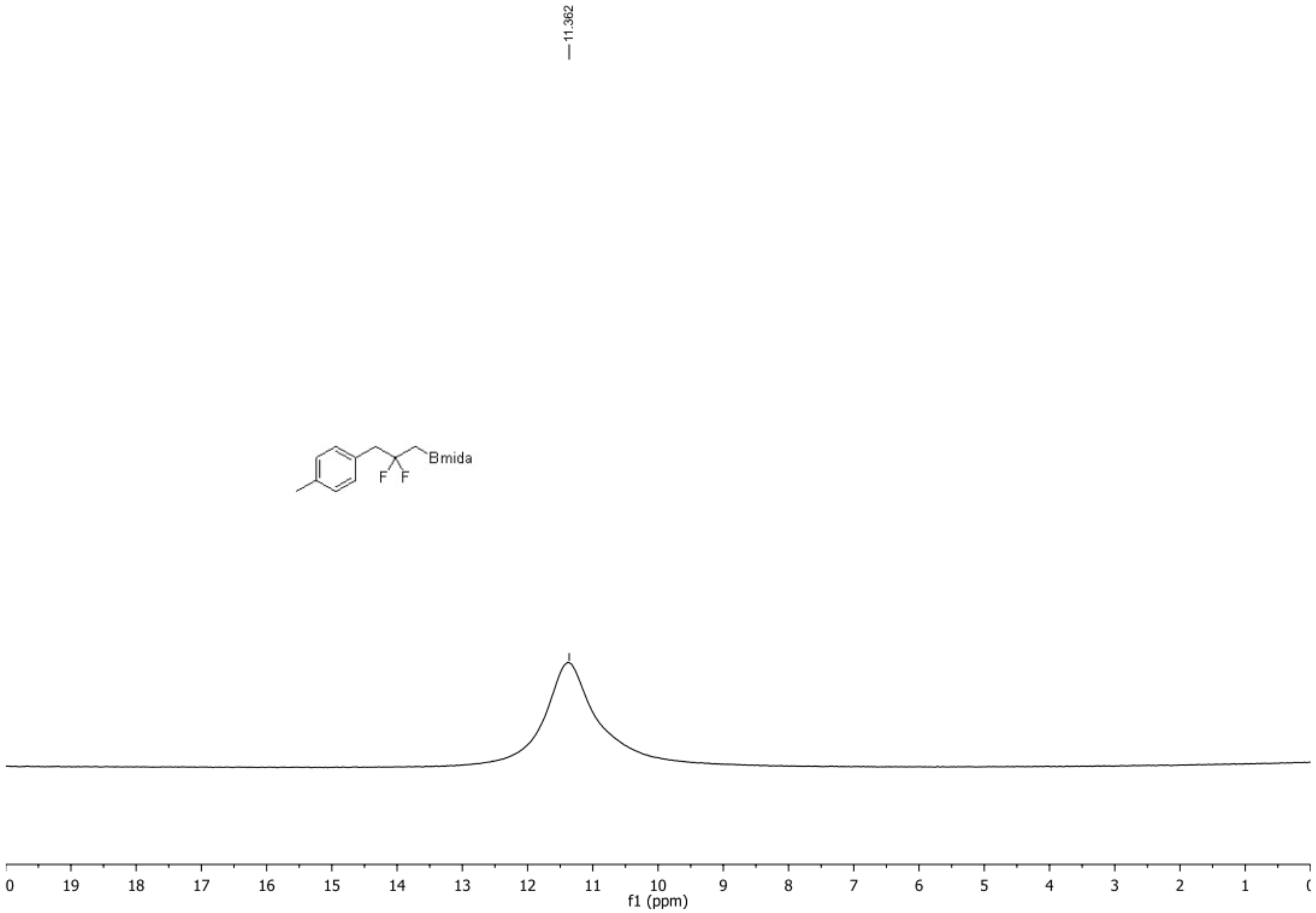

^1H NMR ($(CD_3)_2CO$, 400 MHz). 2-(3-(4-Bromophenyl)-2,2-difluoropropyl)-6-methyl-1,3,6,2-dioxazaborocane-4,8-dione (**2j**)

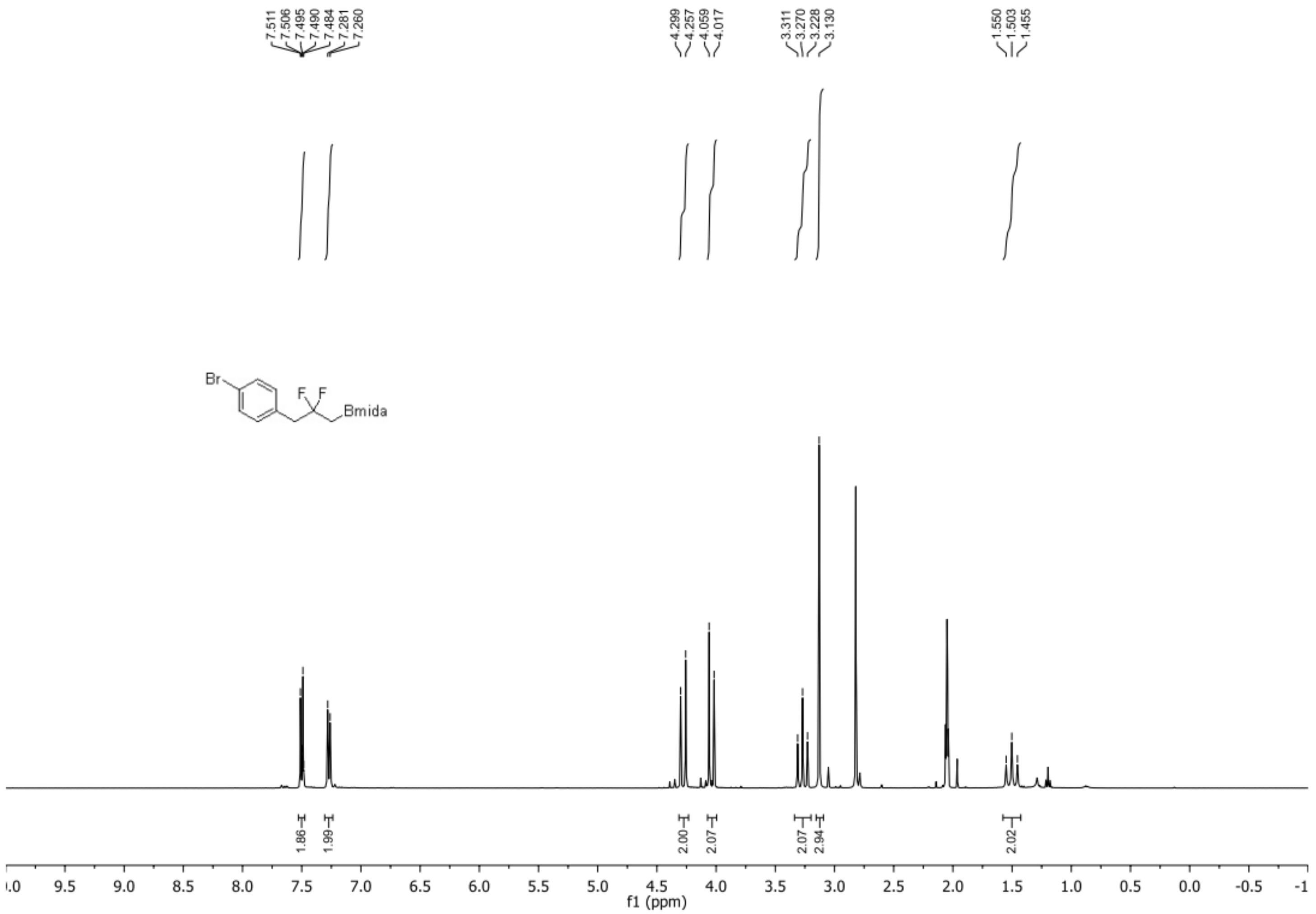

^{13}C NMR ($(CD_3)_2CO$, 100 MHz). 2-(3-(4-Bromophenyl)-2,2-difluoropropyl)-6-methyl-1,3,6,2-dioxazaborocane-4,8-dione (**2j**)

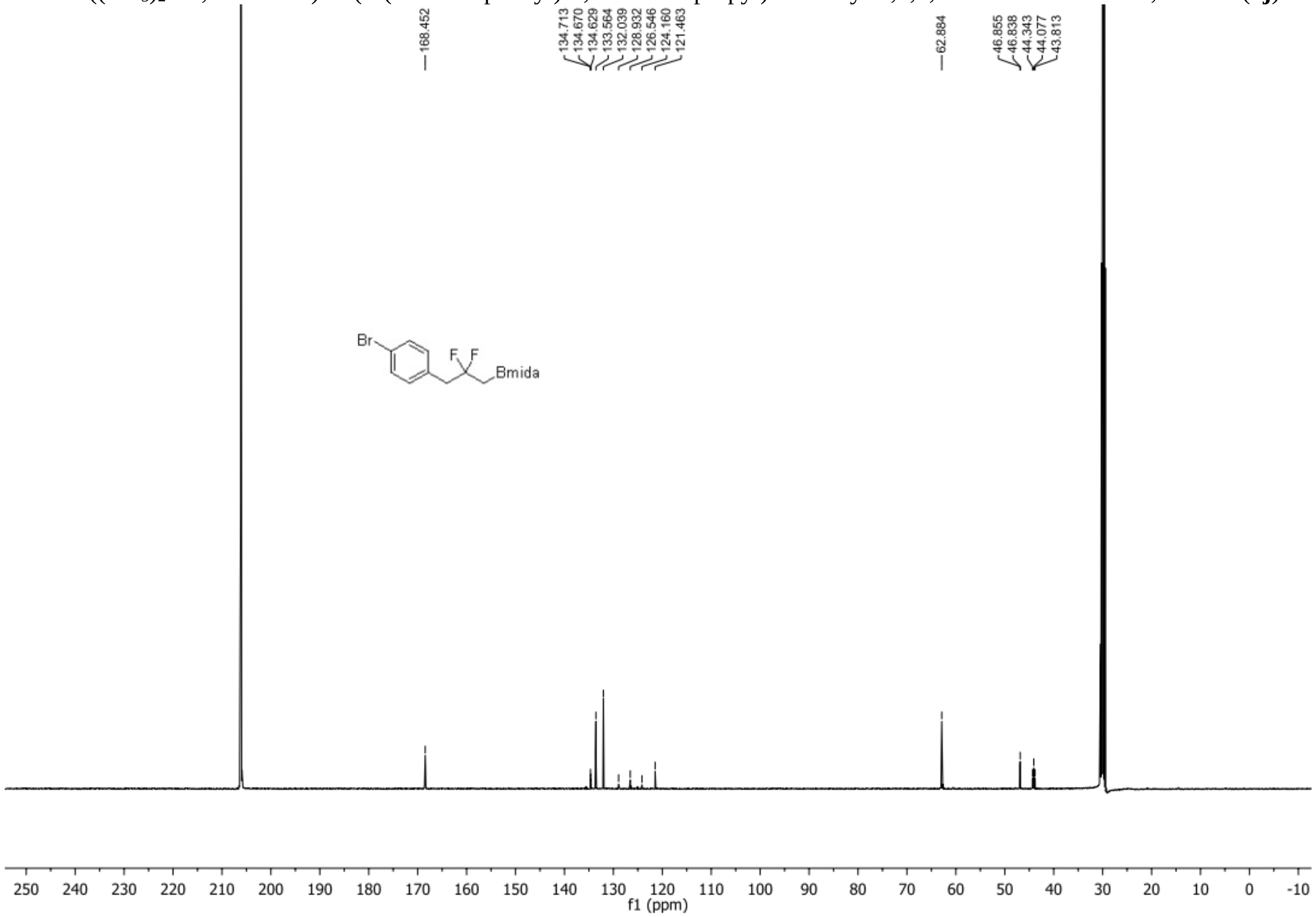

^{19}F NMR (($(CD_3)_2CO$, 377 MHz). 2-(3-(4-Bromophenyl)-2,2-difluoropropyl)-6-methyl-1,3,6,2-dioxazaborocane-4,8-dione (**2j**)

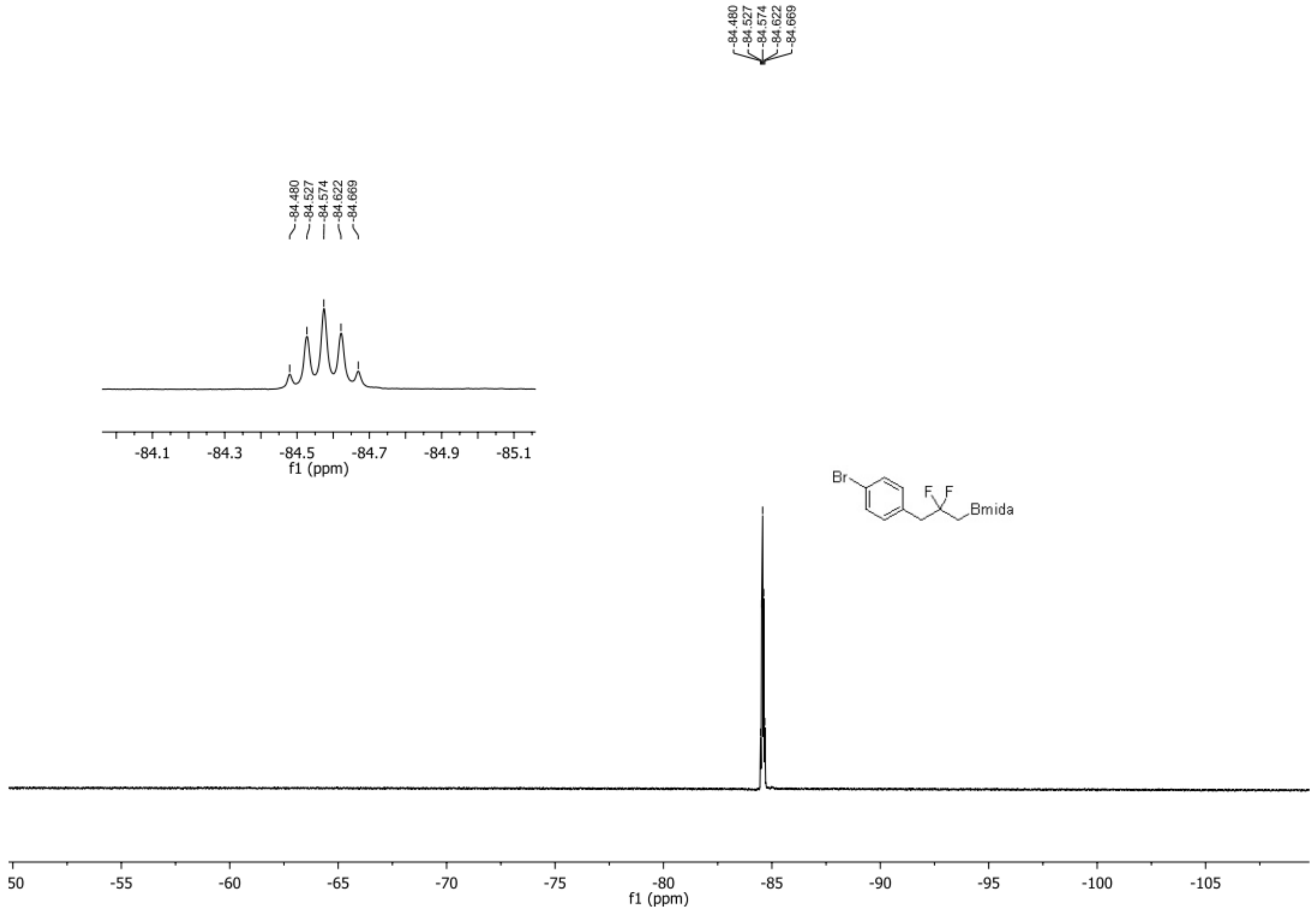

^{11}B NMR ($(CD_3)_2CO$, 128 MHz). 2-(3-(4-Bromophenyl)-2,2-difluoropropyl)-6-methyl-1,3,6,2-dioxazaborocane-4,8-dione (**2j**)

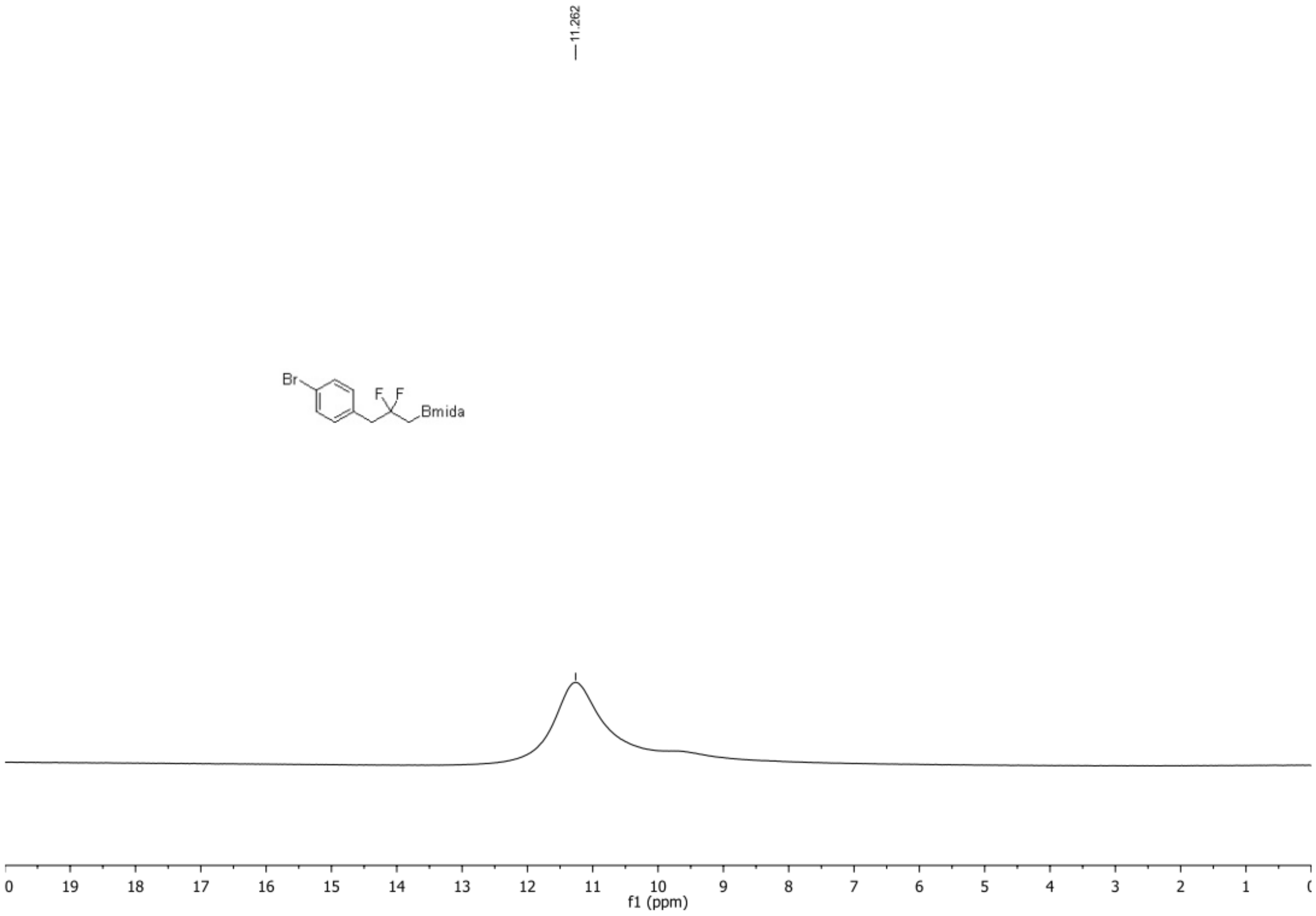

^{1}H NMR ($(CD_3)_2CO$, 400 MHz). 2-(4-(1,3-Dioxoisoindolin-2-yl)-2,2-difluorobutyl)-6-methyl-1,3,6,2-dioxazaborocane-4,8-dione (**2k**)

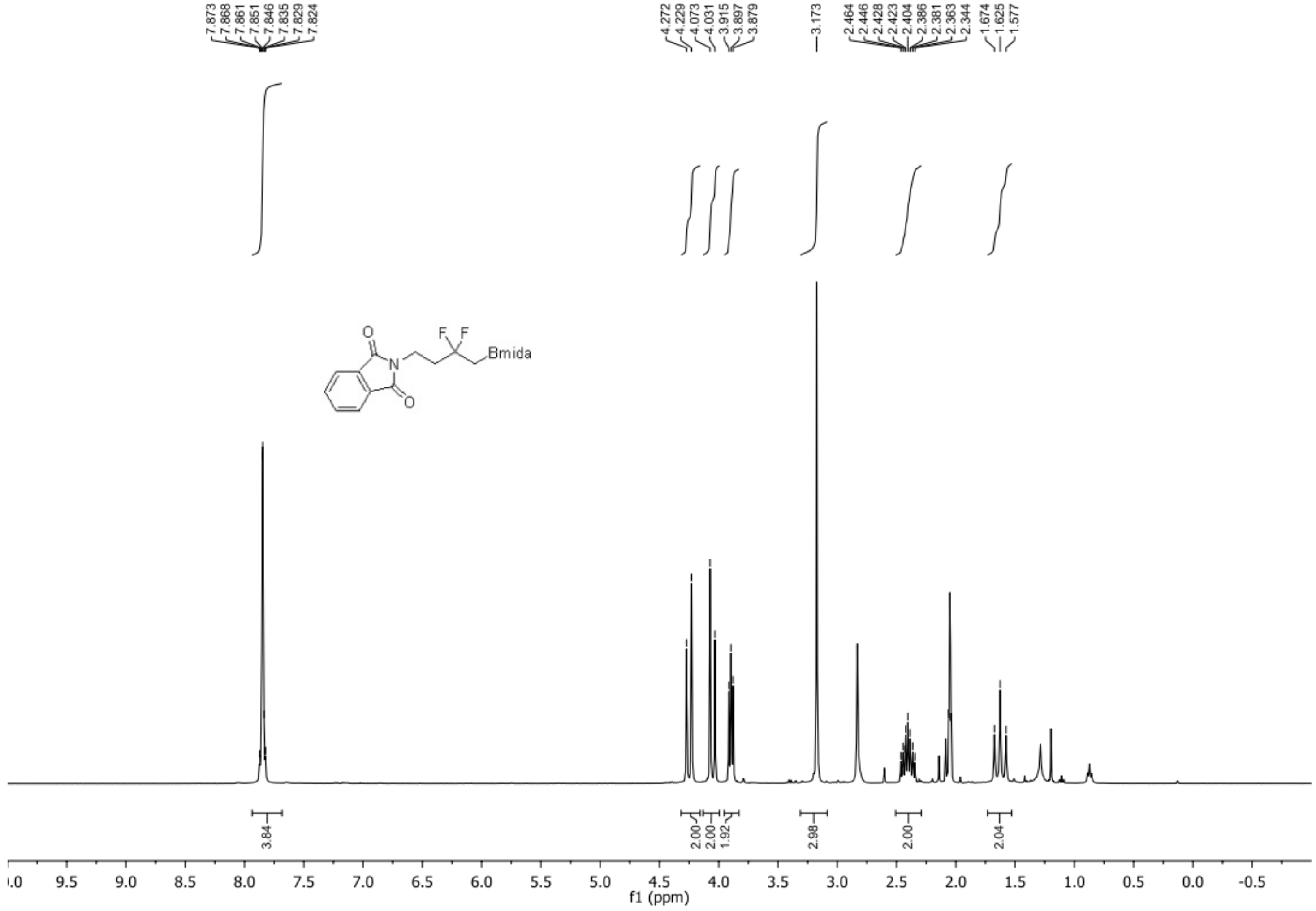

^{13}C NMR ($(CD_3)_2CO$, 100 MHz). 2-(4-(1,3-Dioxoisoindolin-2-yl)-2,2-difluorobutyl)-6-methyl-1,3,6,2-dioxazaborocane-4,8-dione (**2k**)

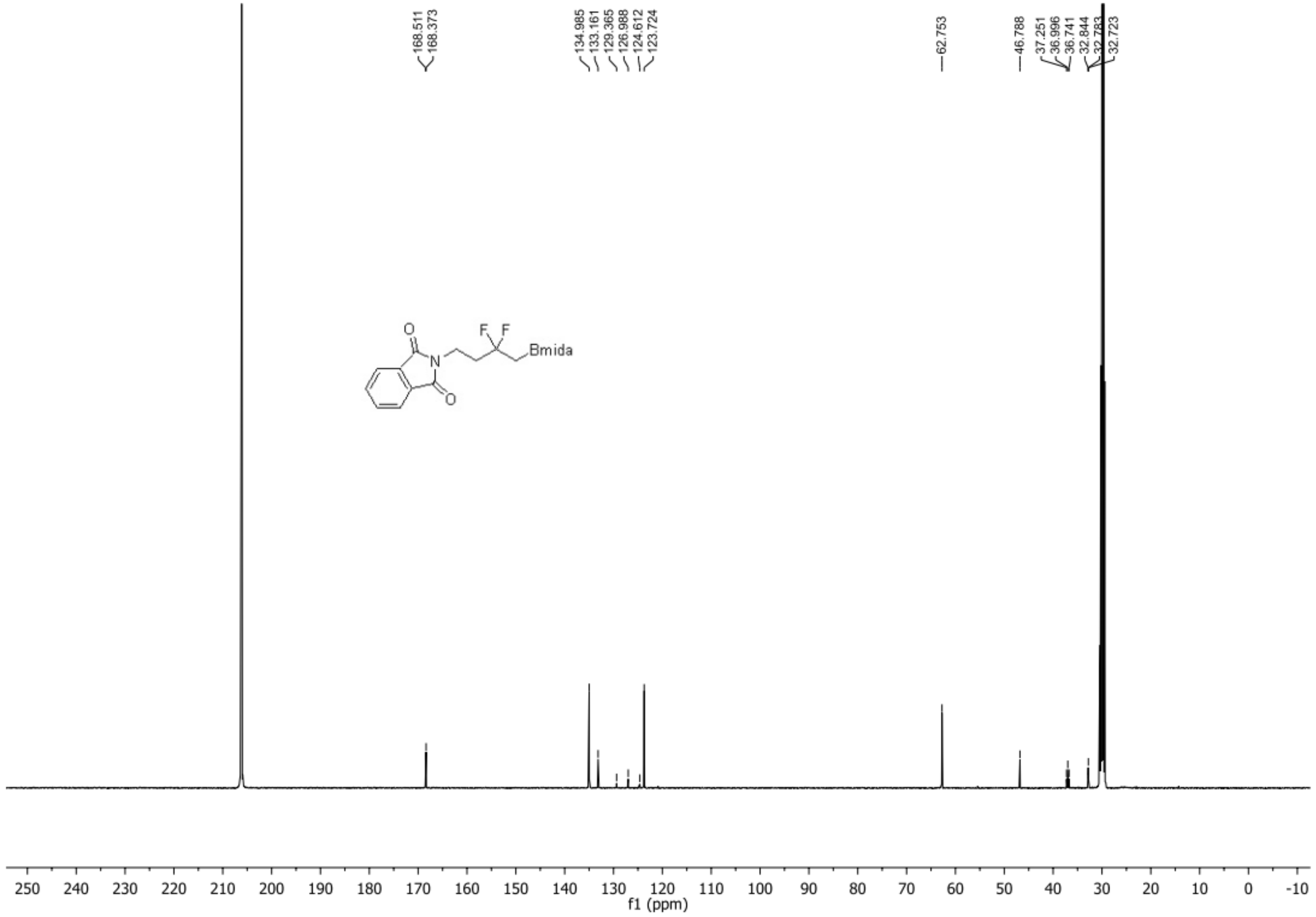

^{19}F NMR ((CD_3)$_2$CO, 377 MHz). 2-(4-(1,3-Dioxoisoindolin-2-yl)-2,2-difluorobutyl)-6-methyl-1,3,6,2-dioxazaborocane-4,8-dione (**2k**)

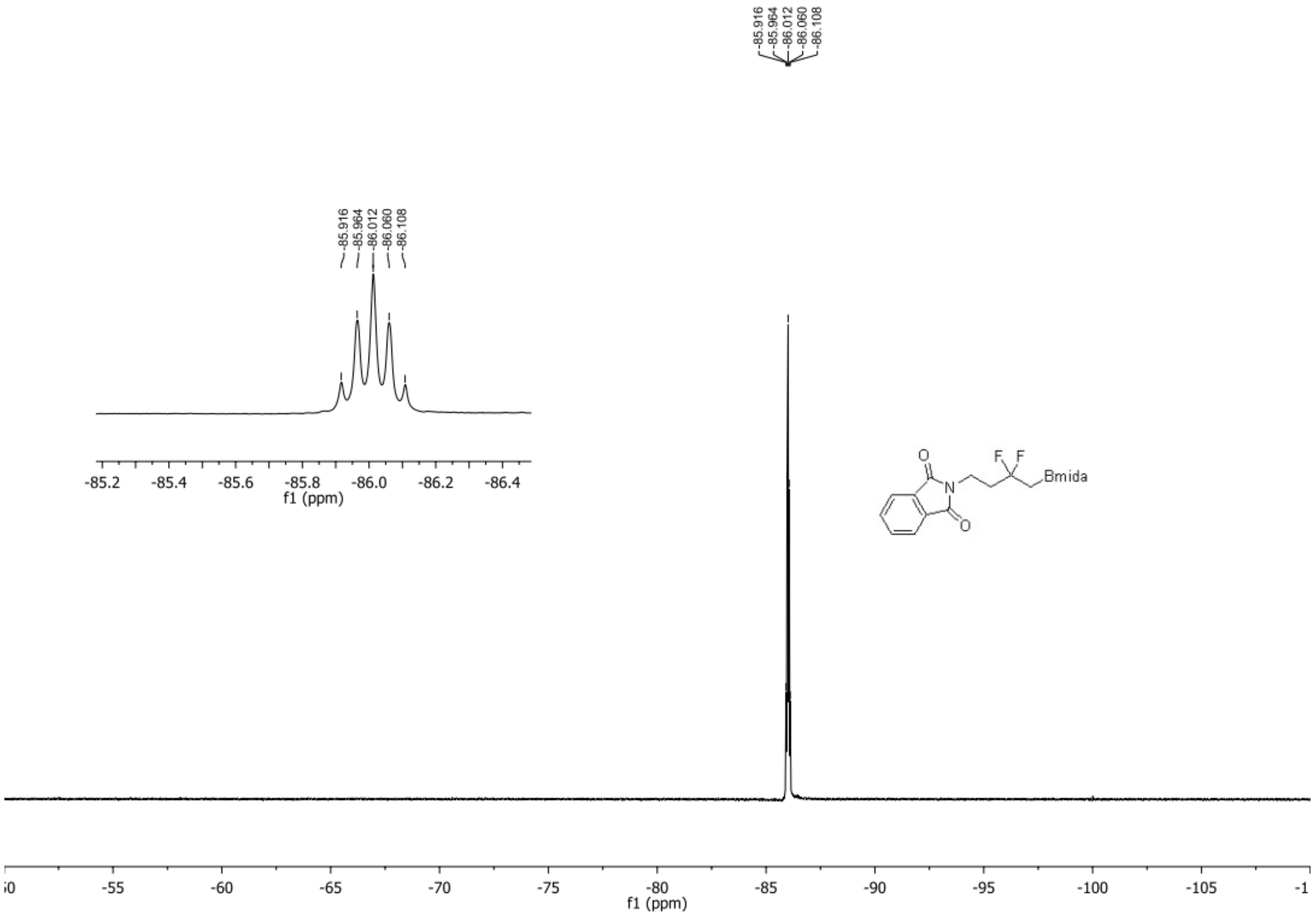

^{11}B NMR ($(CD_3)_2CO$, 128 MHz). 2-(4-(1,3-Dioxoisoindolin-2-yl)-2,2-difluorobutyl)-6-methyl-1,3,6,2-dioxazaborocane-4,8-dione (**2k**)

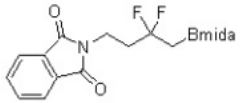

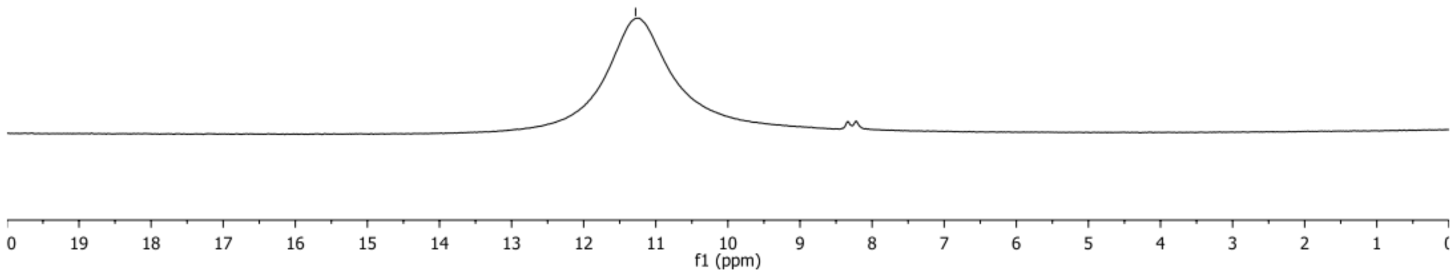

^{1}H NMR (($(CD_3)_2CO$, 400 MHz). 2-(2-Cyclohexyl-2,2-difluoroethyl)-6-methyl-1,3,6,2-dioxazaborocane-4,8-dione (**2l**)

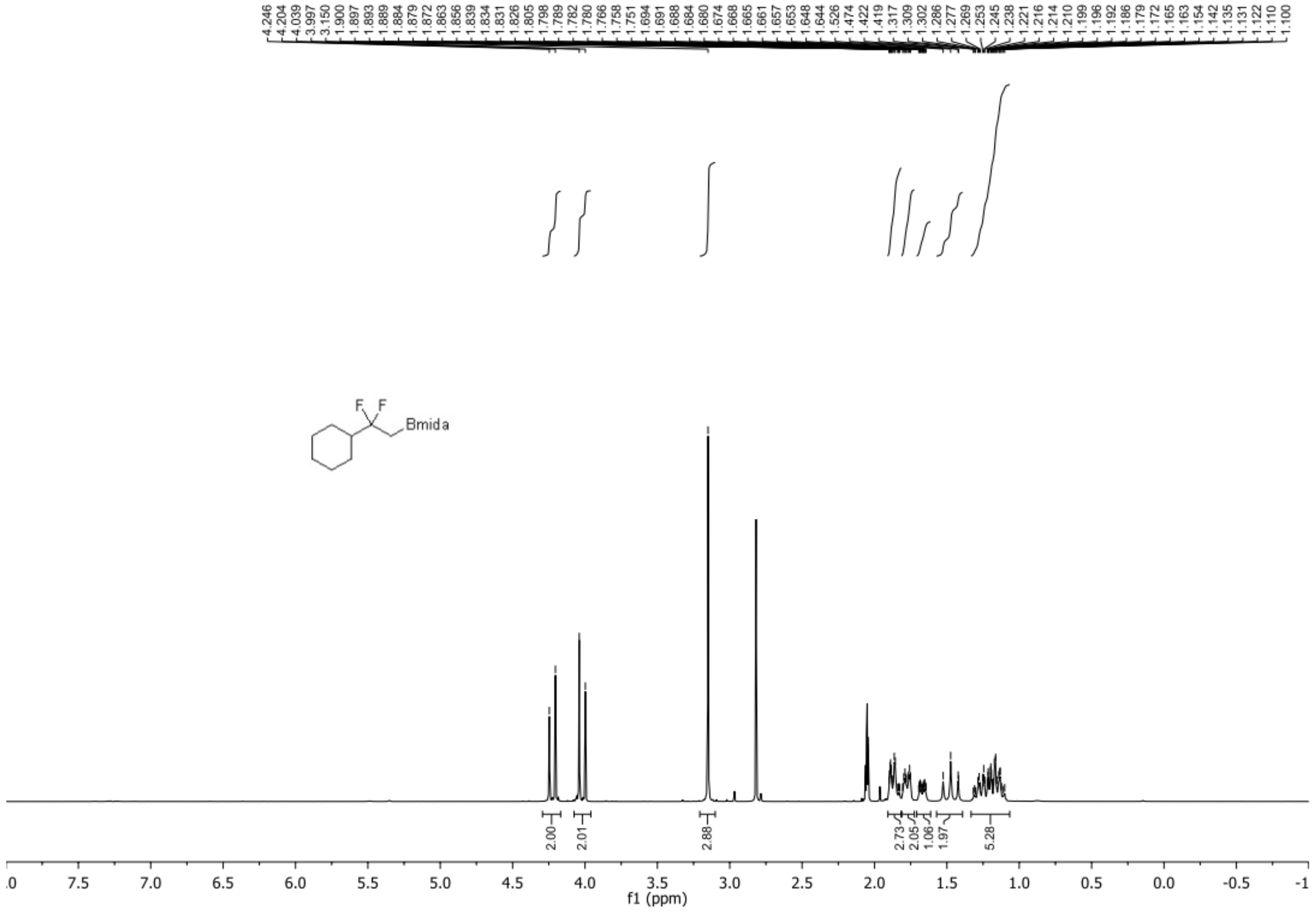

^{13}C NMR (($(CD_3)_2CO$, 100 MHz). 2-(2-Cyclohexyl-2,2-difluoroethyl)-6-methyl-1,3,6,2-dioxazaborocane-4,8-dione (**2l**)

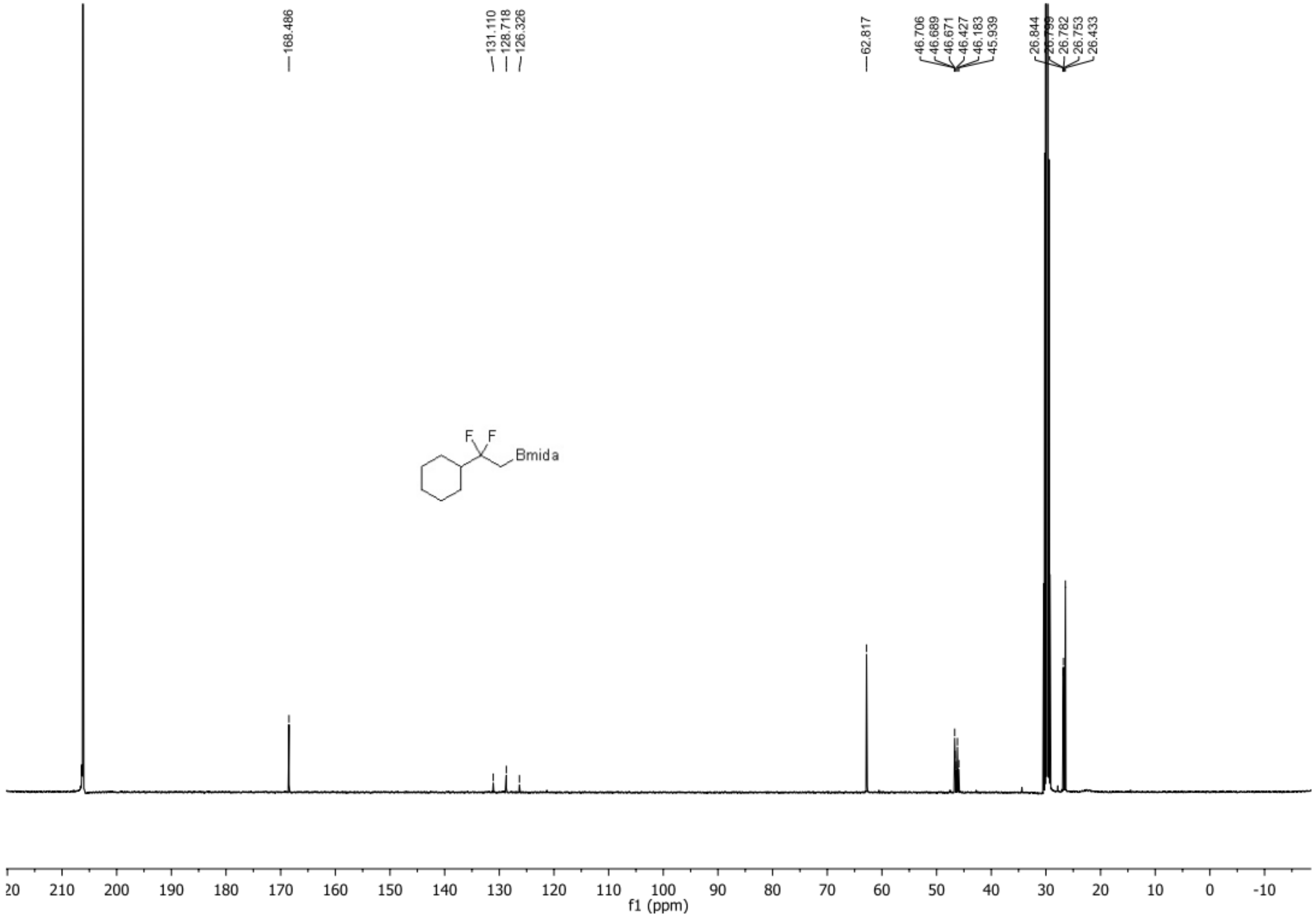

^{19}F NMR (($(CD_3)_2CO$, 377 MHz). 2-(2-Cyclohexyl-2,2-difluoroethyl)-6-methyl-1,3,6,2-dioxazaborocane-4,8-dione (**2l**)

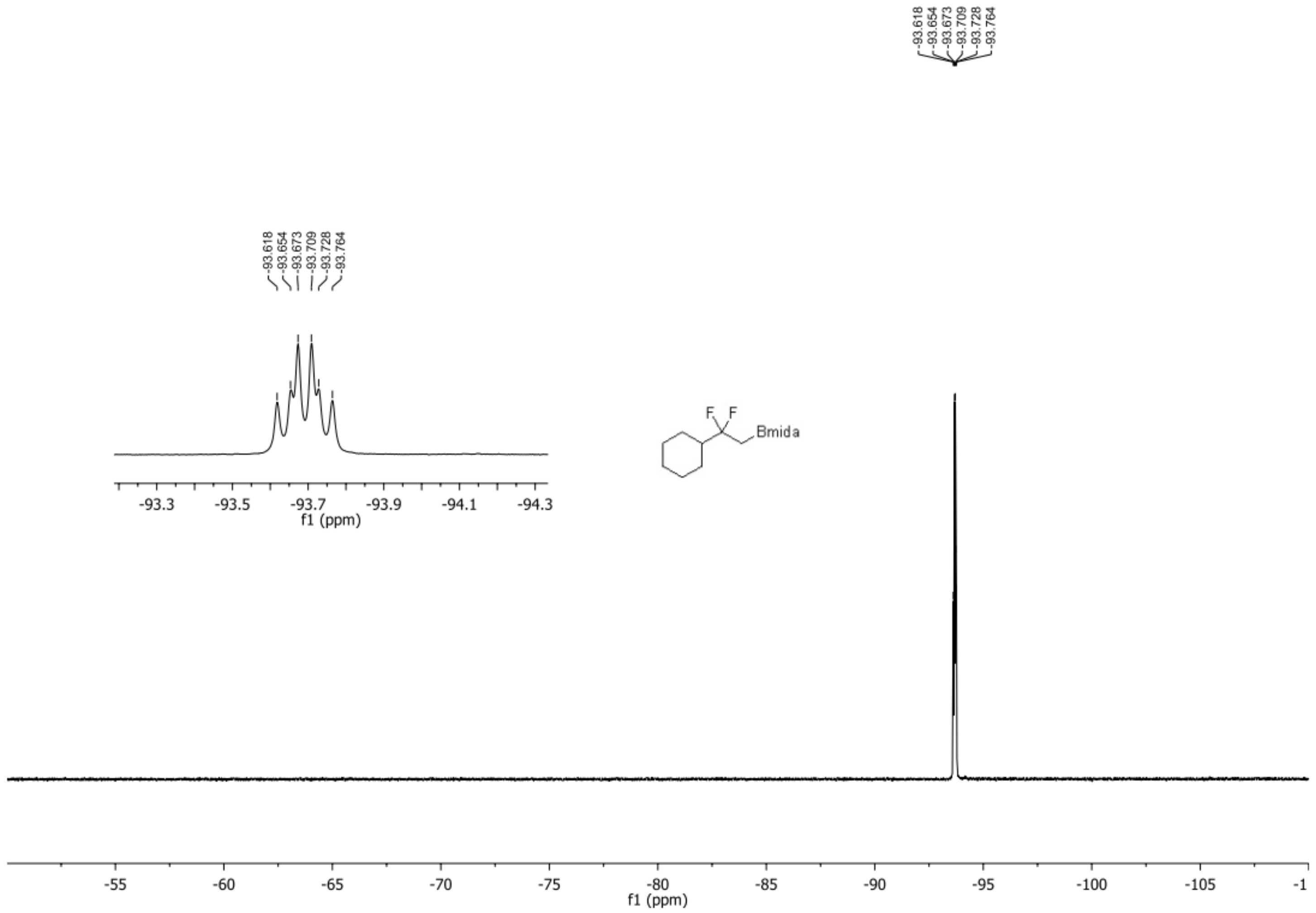

^{11}B NMR ($(CD_3)_2CO$, 128 MHz). 2-(2-Cyclohexyl-2,2-difluoroethyl)-6-methyl-1,3,6,2-dioxazaborocane-4,8-dione (**2l**)

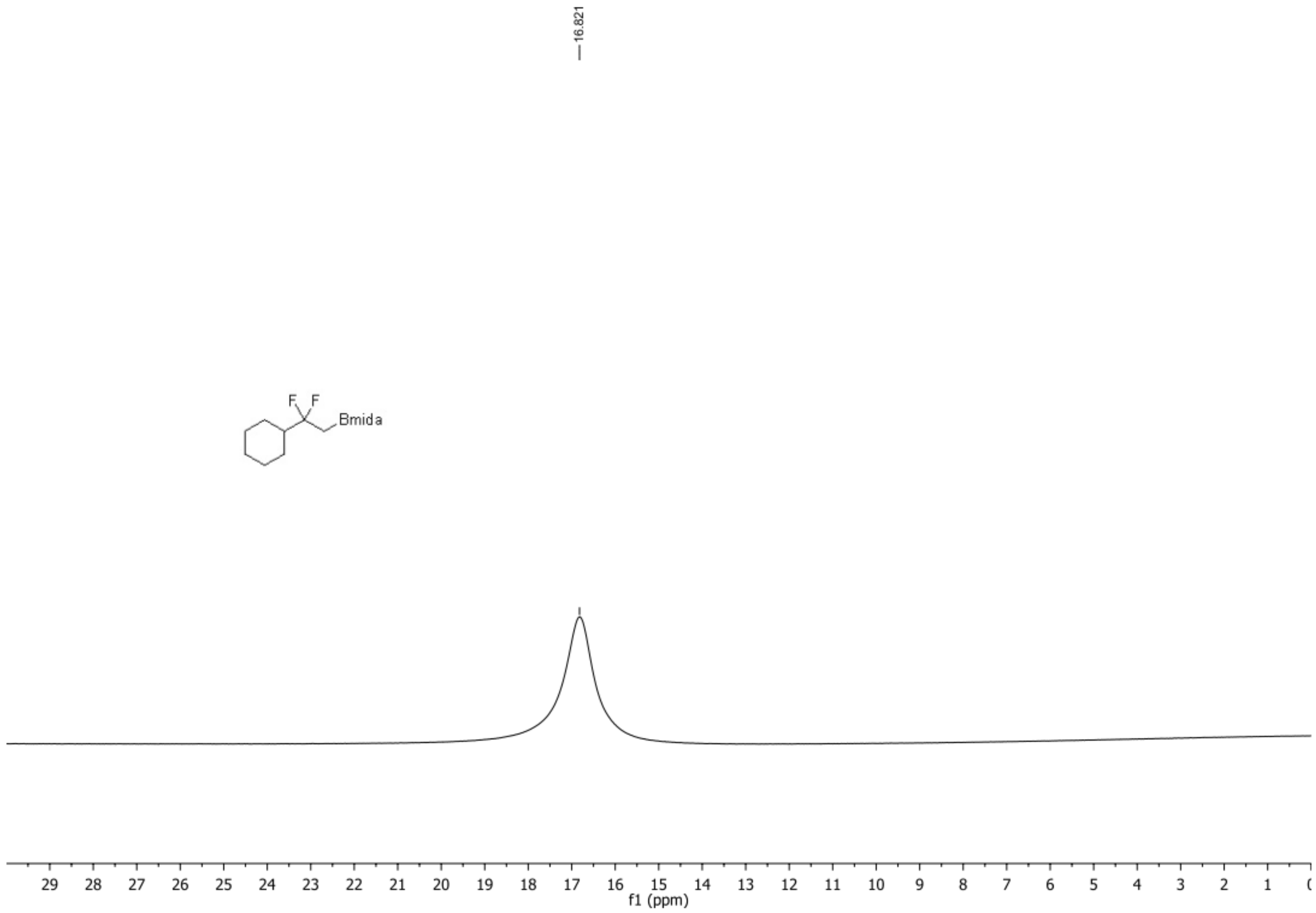

Spectra for control experiments for stability of 1a, 2a and 1h in pyr·9HF (see S23 and Eqs. 1-3 in the paper)

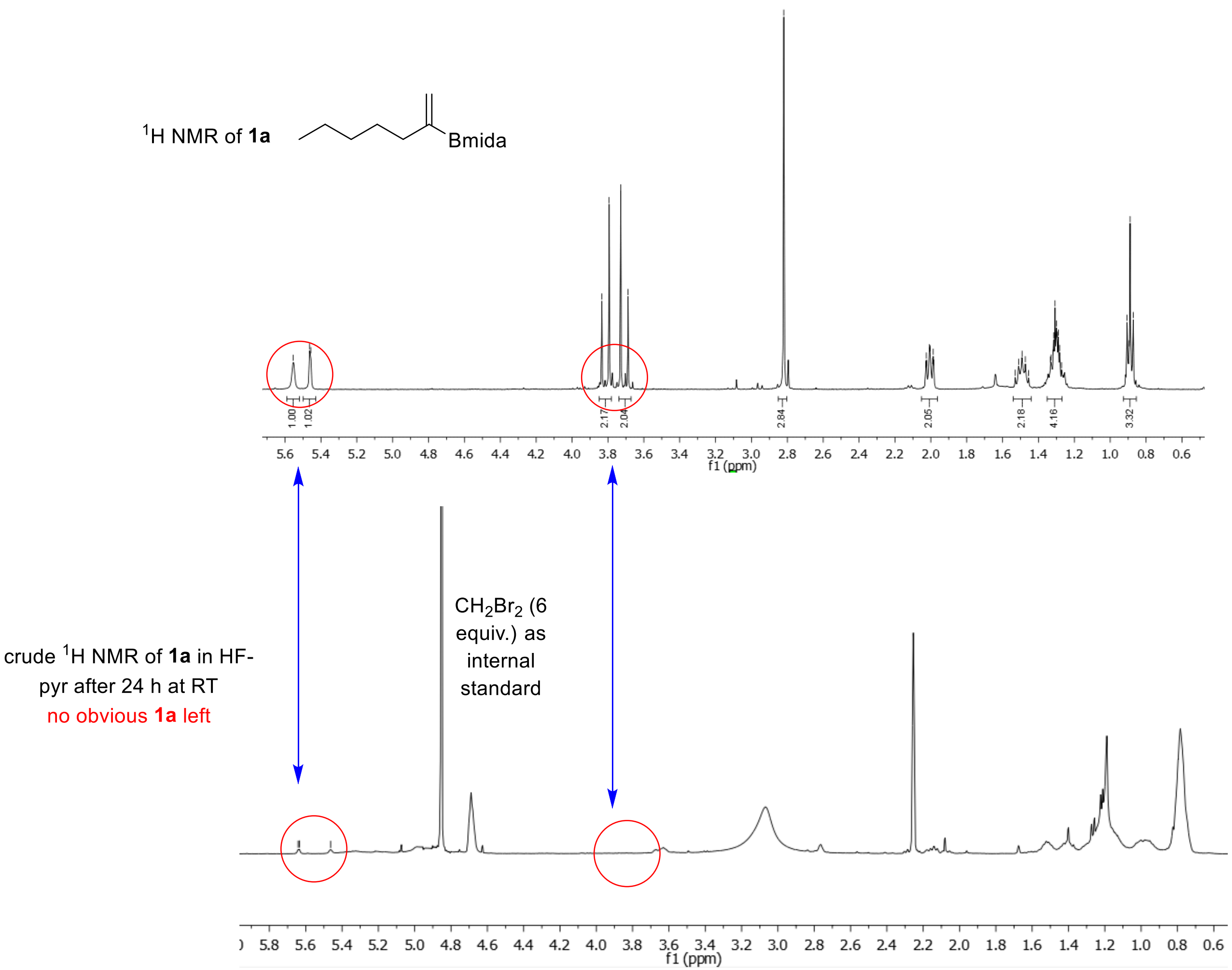

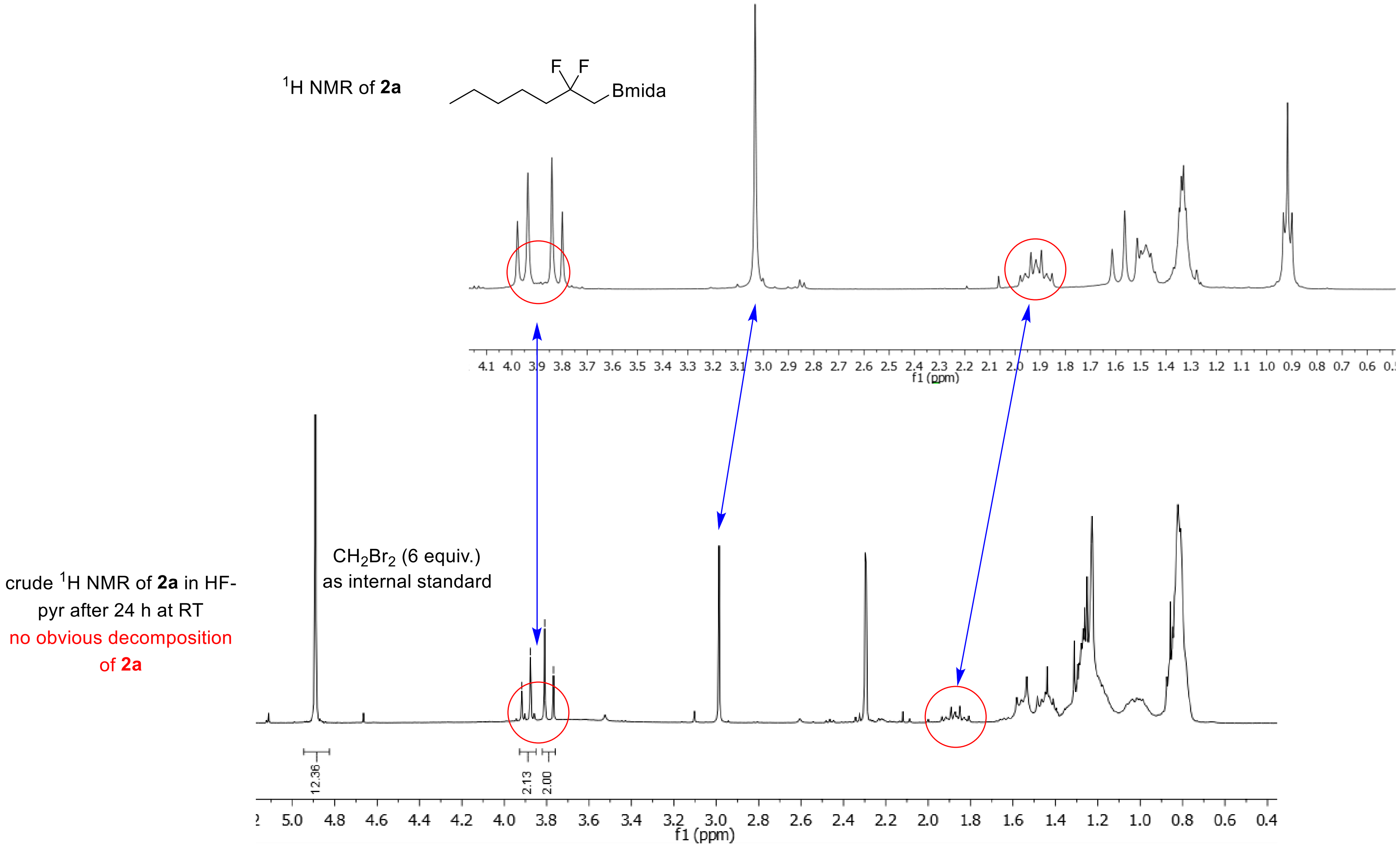

^{1}H NMR of **2a**
F F
Bmida
crude ^{1}H NMR of **2a** in HF-pyr after 24 h at RT
no obvious decomposition of **2a**
CH_2Br_2 (6 equiv.) as internal standard
12.36
2.13
2.00
f1 (ppm)
f1 (ppm)

crude ^1H NMR of **1h** in
HF-pyr after 24 h at RT
56% **1h** remained

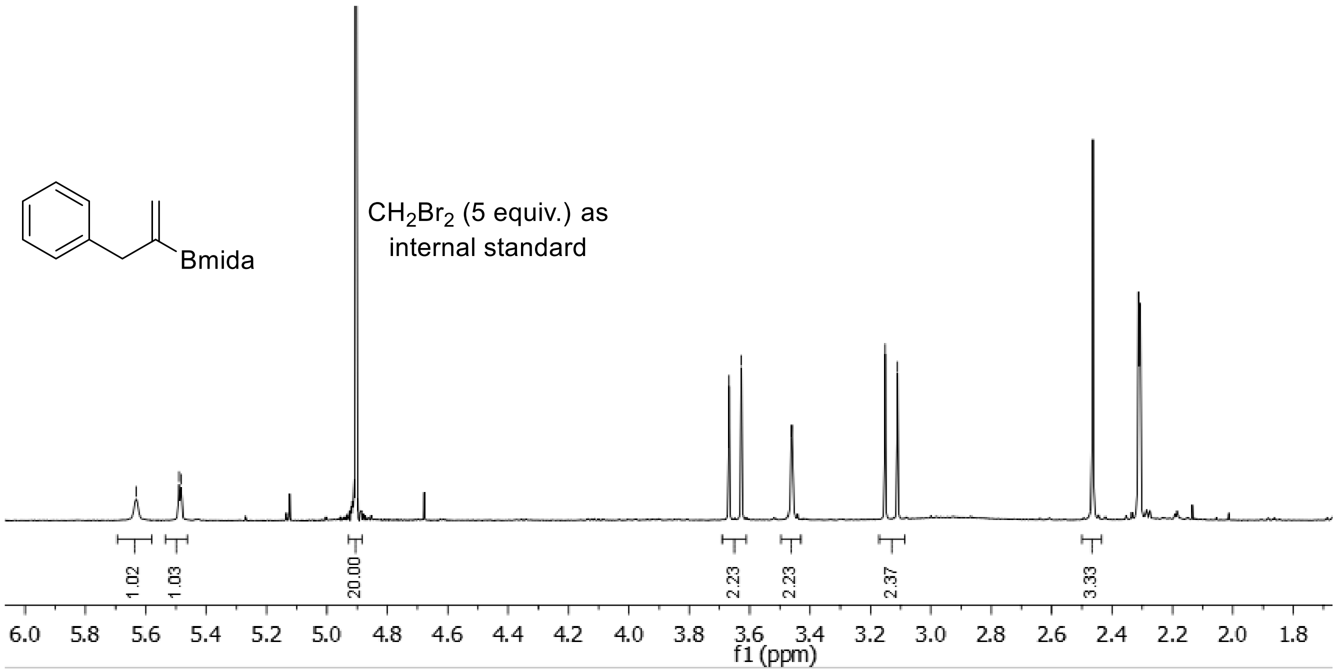

^{1}H NMR ($CDCl_3$, 400 MHz). 2-(2-cyclohexyl-2,2-difluoroethyl)-4,4,5,5-tetramethyl-1,3,2-dioxaborolane (**4**)

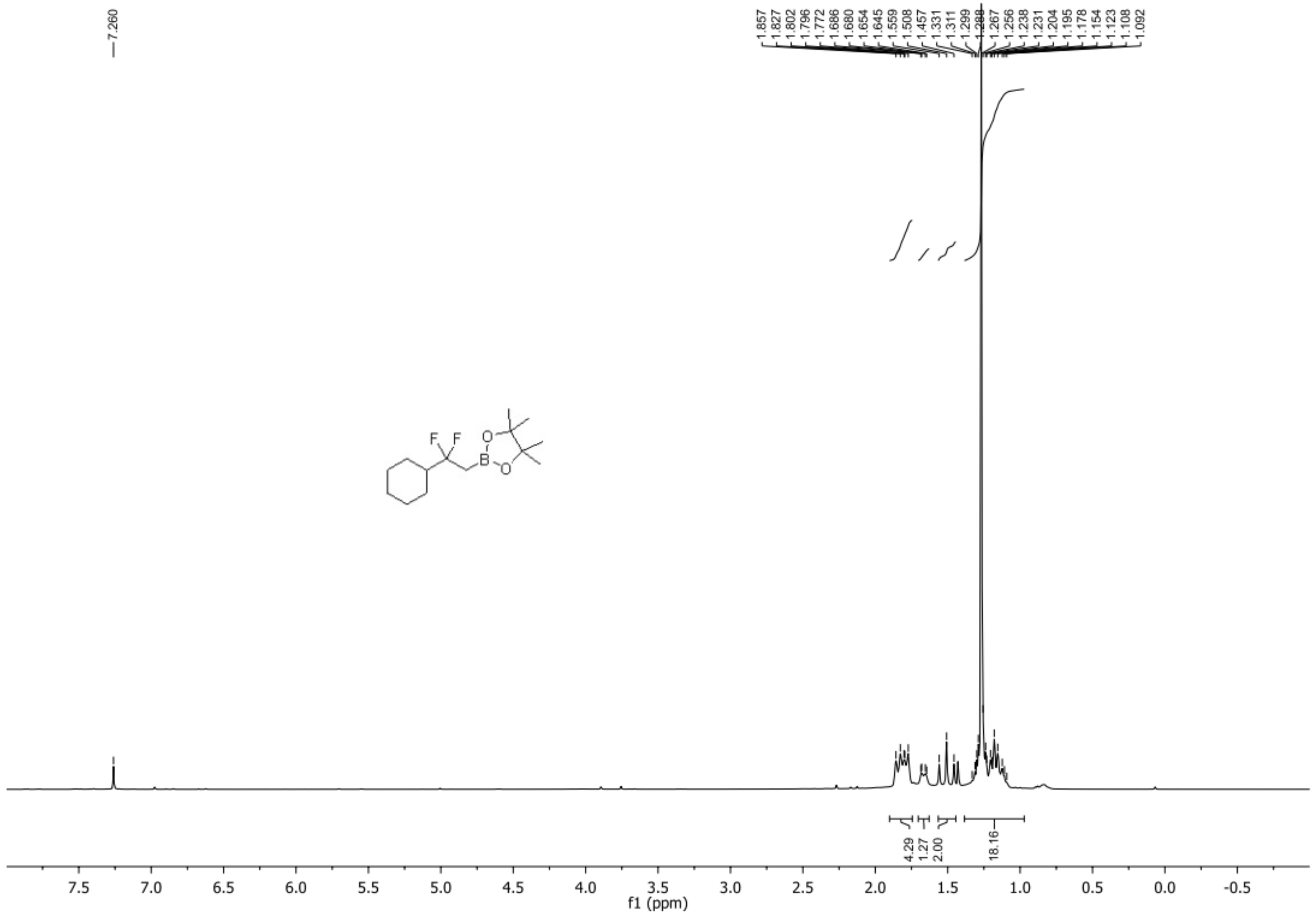

^{13}C NMR ($CDCl_3$, 100 MHz). 2-(2-cyclohexyl-2,2-difluoroethyl)-4,4,5,5-tetramethyl-1,3,2-dioxaborolane (**4**)

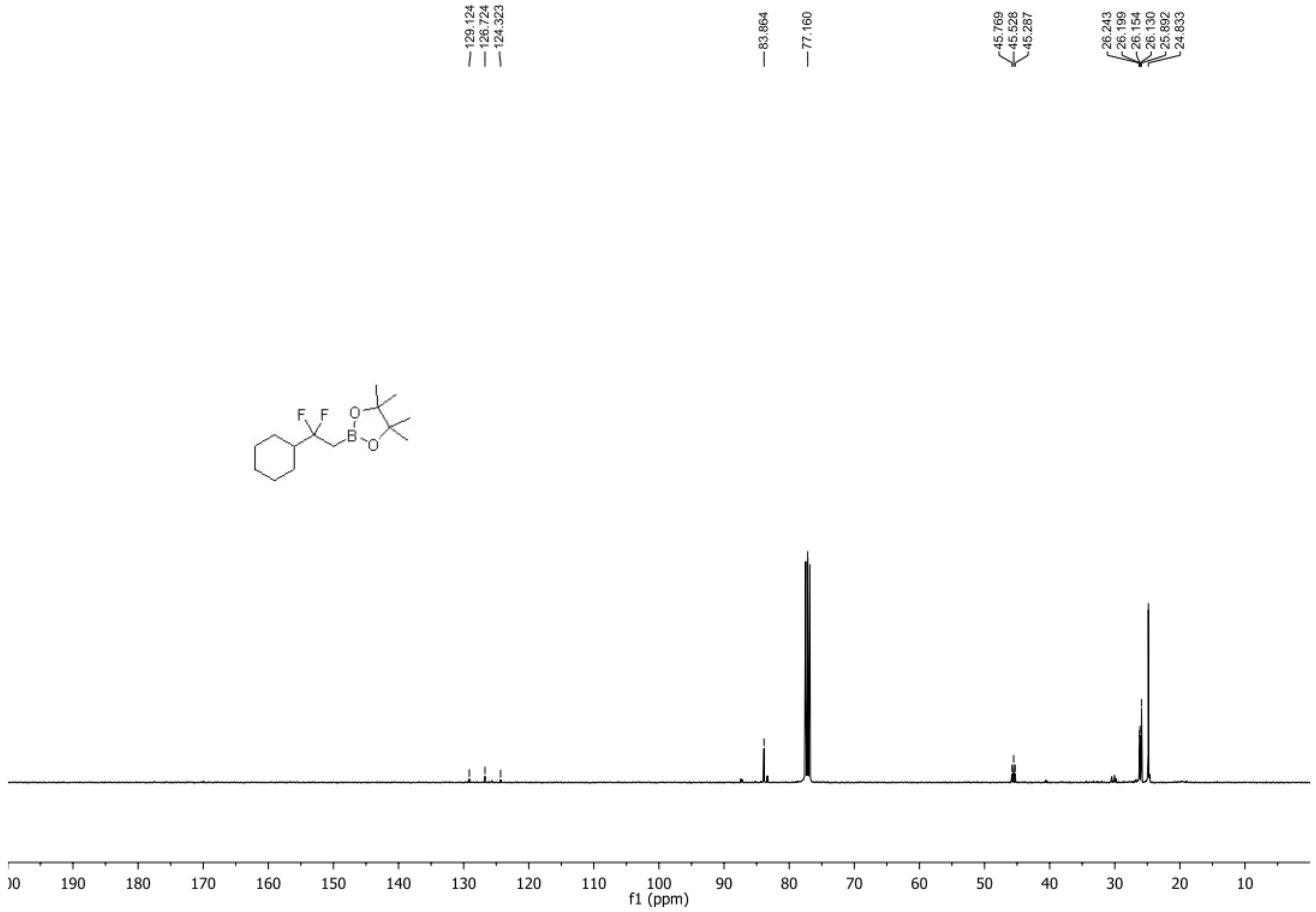

^{19}F NMR ($CDCl_3$, 377 MHz). 2-(2-cyclohexyl-2,2-difluoroethyl)-4,4,5,5-tetramethyl-1,3,2-dioxaborolane (**4**)

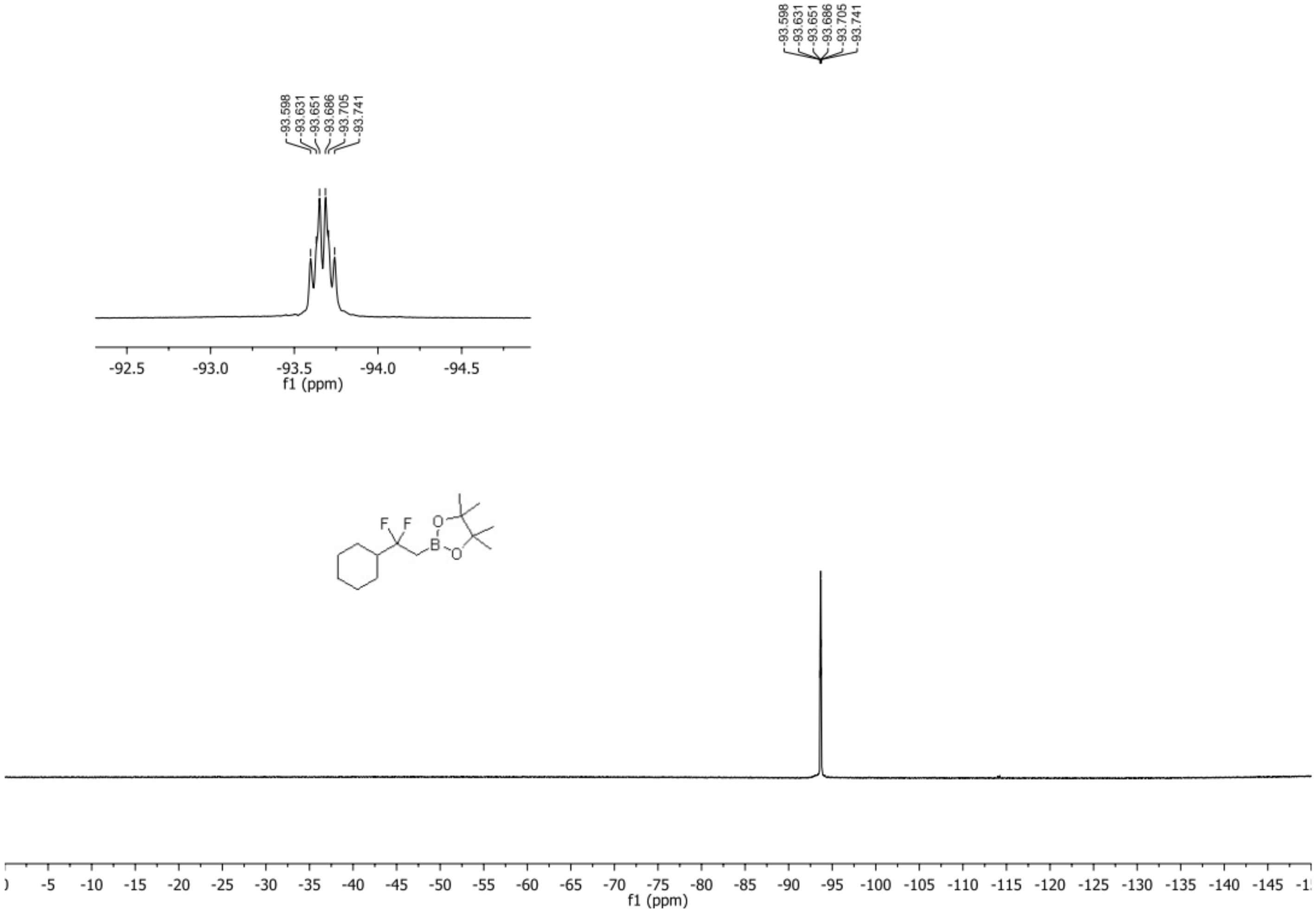

^{11}B NMR ($CDCl_3$, 128 MHz). 2-(2-cyclohexyl-2,2-difluoroethyl)-4,4,5,5-tetramethyl-1,3,2-dioxaborolane (**4**)

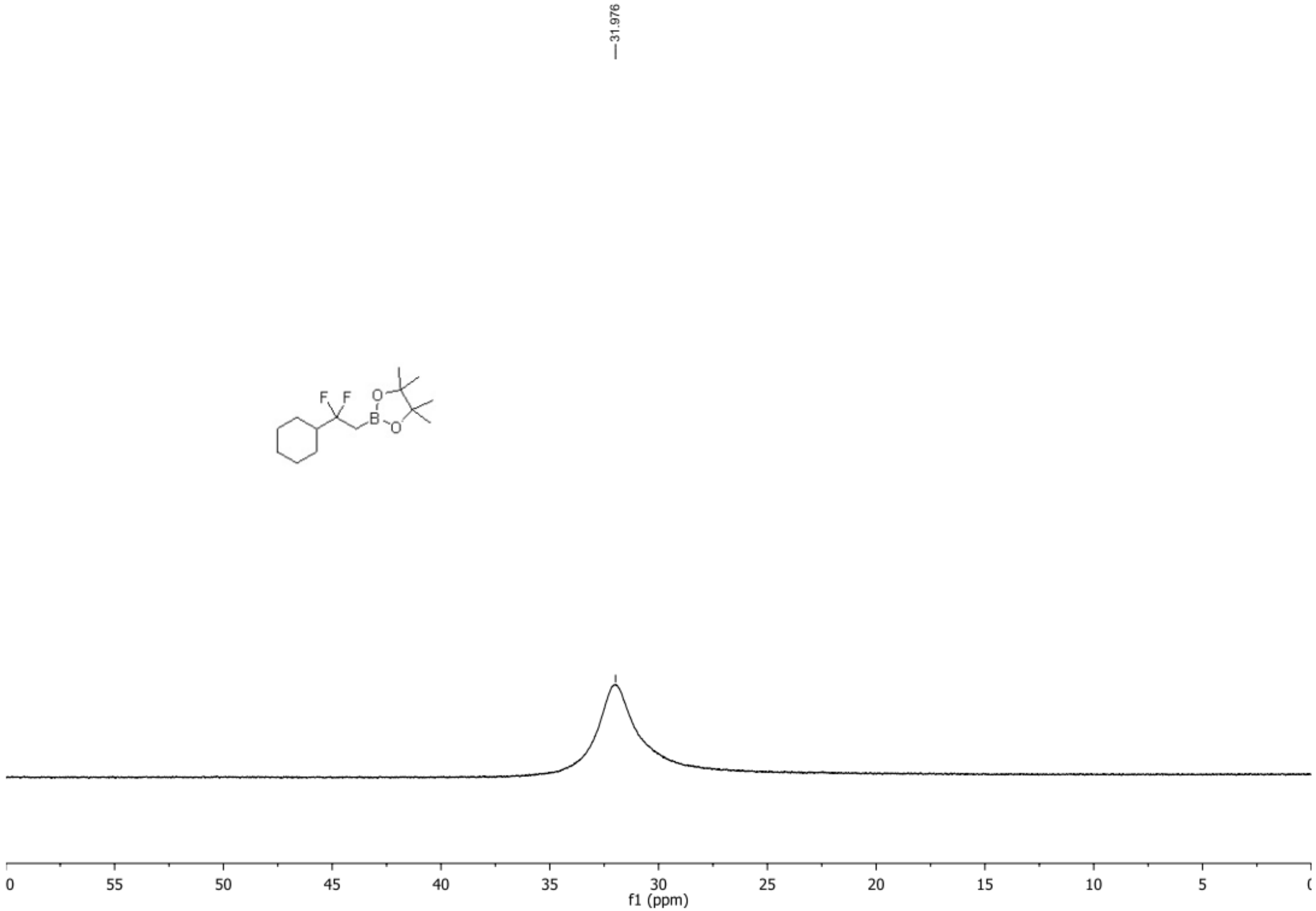

Supporting Information for the Computational Studies

Computational details

The calculations were carried out using the B3LYP-D3(BJ) functional[14-17] as implemented in the Gaussian 16 program package.[18] For the geometry optimizations, the LANL2DZ pseudopotential[19] and the 6-31G(d,p) basis set was used for all other atoms. Implicit solvation using the SMD[20] model with the parameters for chloroform was included in the geometry optimization. To obtain better accuracy, single-point calculations were carried out on the basis of the optimized structures with LANL2DZ pseudopotential with the corresponding basis set augmented with polarization and diffuse functions was used for the iodine[21] and the 6-311+G(2d,2p) basis set for all other elements. Frequencies were calculated at the level of theory of the geometry optimization. The reported energies are Gibbs free energies in solution. A correction term of [RT ln(24.46) = +1.89 kcal/mol] was added to free energy for each species to account for the 1 atm to 1M standard state change.

Catalytic cycle

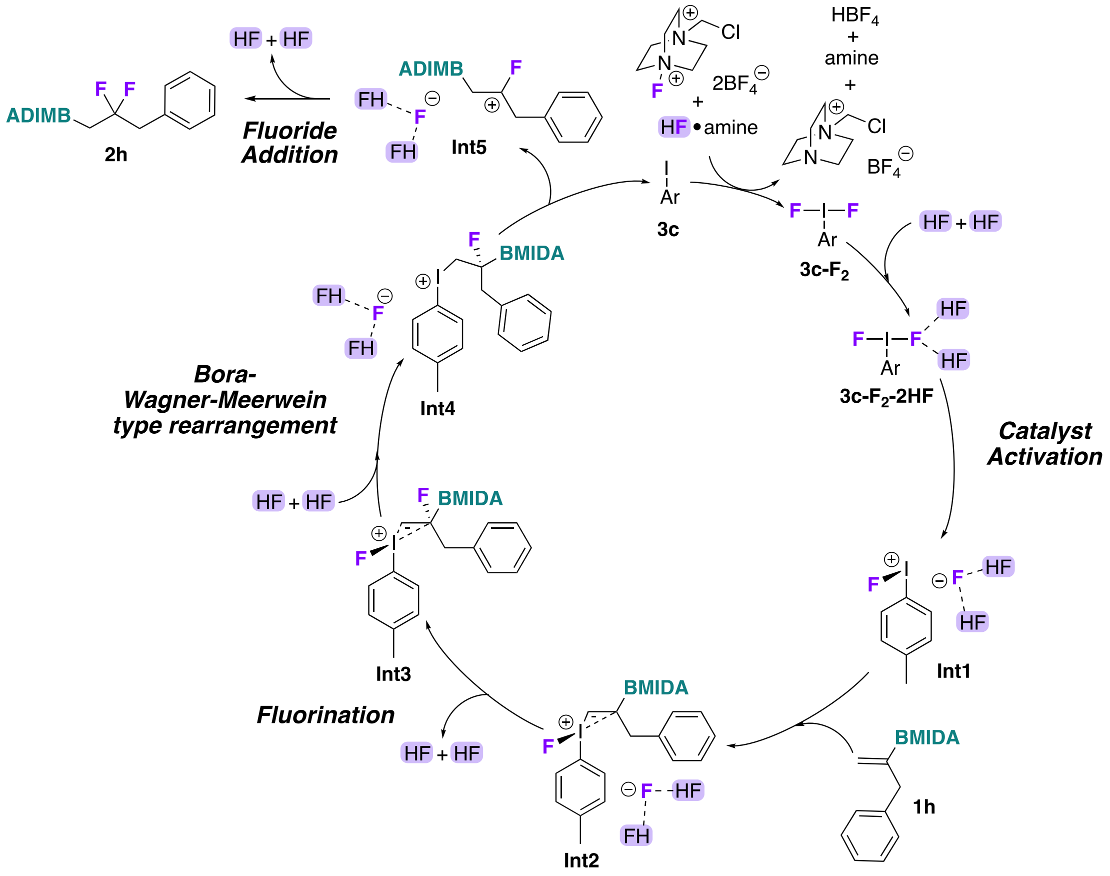

Scheme S11. Catalytic cycle based on DFT calculations for aryl iodide-catalyzed bora-Wagner-Meerwein type rearrangement of **1h** with catalyst **3c**.

Optimized structures of intermediates and transition states

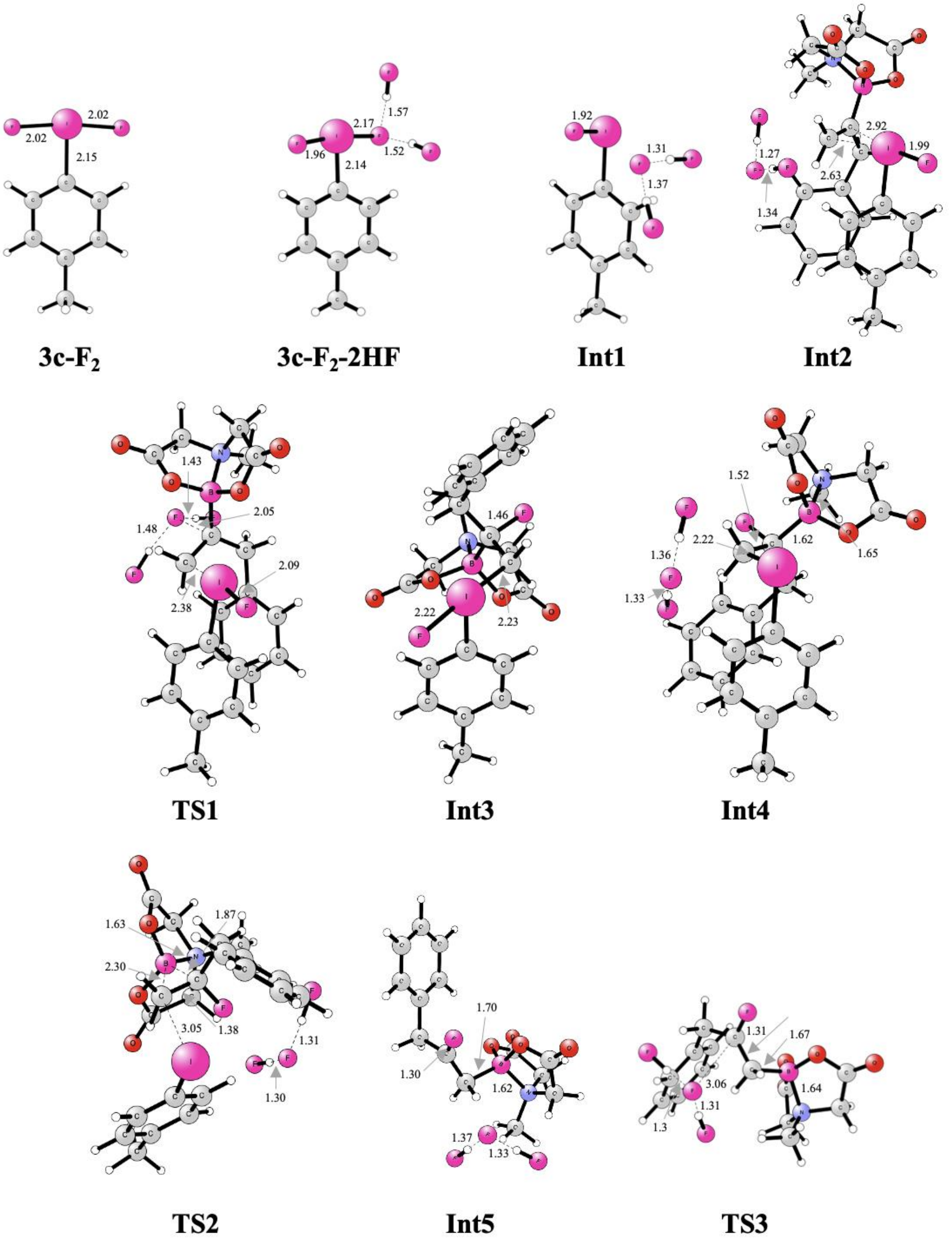

Figure S1. Optimized structures of intermediates and transition states along the reaction pathway. Selected bond distances are indicated in Å.

TS for the nucleophilic attack at the less substituted carbon of 1h

From **Int2**, we considered the possibility of the counterion $(HF)_2F^-$ attacking the less substituted carbon of the olefin (**1h**). Such a transformation leads to the reverse regioselectivity and was calculated to be 3 kcal/mol higher in energy compared to the attack of $(HF)_2F^-$ at the most substituted carbon of **1h** (**TS1**).

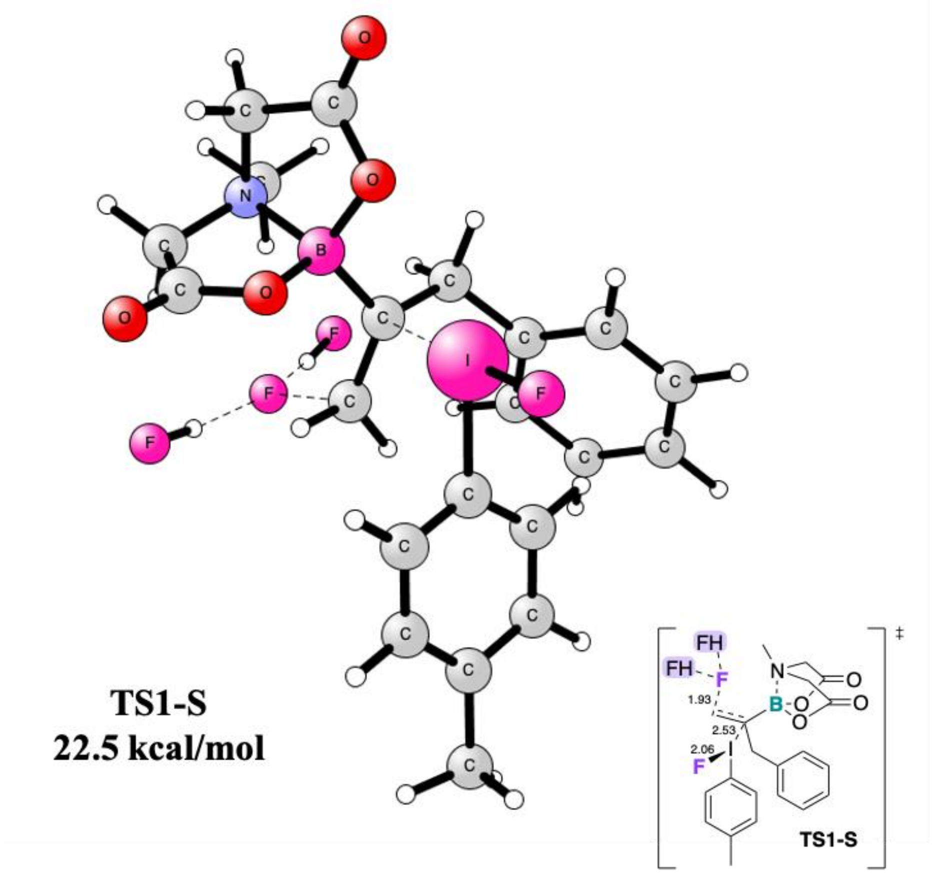

Figure S2. Optimized structure of the transition state for the nucleophilic attack at the less substituted carbon of **1h**. Energy relative to **3c-F₂** is indicated.

Alternative mechanism Wagner-Meerwein rearrangement of the alkyl group and nucleophilic attack of the $(HF)_2F^-$ in Int4

From **Int4**, we considered an alternative mechanism where the alkyl group undergoes a 1,2-rearrangement. This step occurs via **TS2-S** (Figure S2), with a calculated barrier of 19.2 kcal/mol relative to **Int2**. This scenario can be ruled out because **TS2-S** is 6.9 kcal/mol higher than **TS2**.

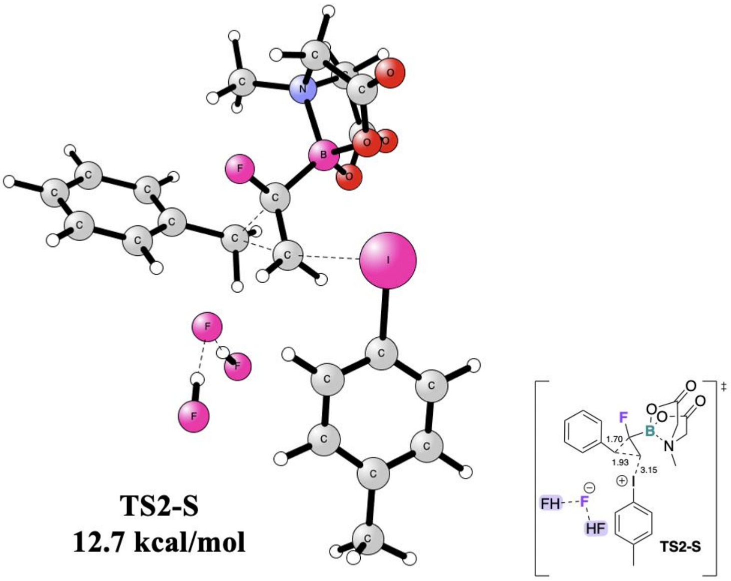

Figure S3. Optimized structure of the transition state of Wagner-Meerwein rearrangement of the alkyl group. Energy relative to **3c-F$_2$** is indicated.

In addition, from **Int4**, we also studied the nucleophilic attack of the counterion $(HF)_2F^-$ at the carbon bonded to the iodine. This alternative TS was found to be 4.0 kcal/mol higher in energy than **TS2**, and this mechanism can therefore also be discarded.

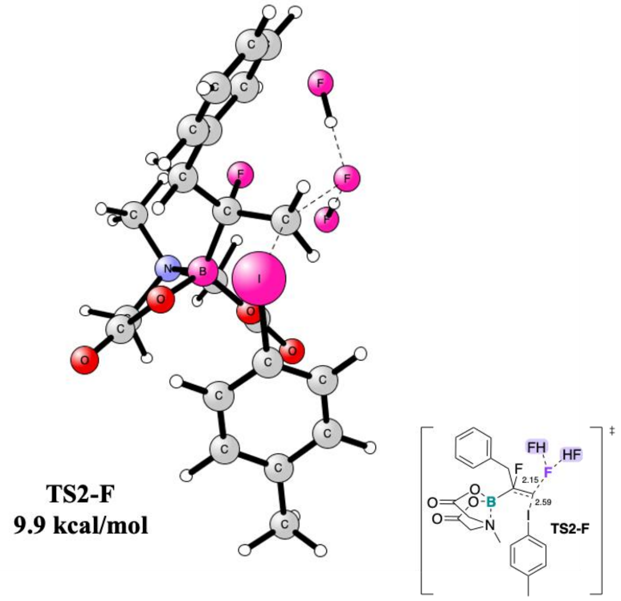

Figure S4. Optimized structure of the transition state for the competing nucleophilic attack of $(HF)_2F^-$. Energy relative to **3c-F$_2$** is indicated.

Energetics of the TSs for aryl/alkyl migration vs Bmida migration

In Table S1, the calculated free energy barriers for the Bmida and aryl/alkyl migrations are compared. For catalyst **3c** and substrate **1h** (entry 1), the difference is 6.9 kcal/mol clearly in favor of the Bmida migration. Using the same catalyst, but with substate **4a**, the difference decreases to 1 kcal/mol in favor of the Bmida migration (entry 2). Considering **3a** as a catalyst, as in the study of Wang and co-workers,[22] the calculated difference using **4a** becomes 0.6 kcal/mol in favor the Bmida migration (entry 3). For this combination, Wang and co-workers observed the formation of the product of the aryl migration, albeit with poor yield (39%). We also performed calculations with ^{t}Bu derivative **4b**, which underwent the rearrangement reaction with a high yield (80%). In this case, the aryl migration is favored by 1.4 kcal/mol (entry 4). In conclusion, these calculations suggests that when styryl boronate derivatives are used the activation energy difference between the Bmida and aryl migrations is relatively small and depends on the substituents of the aryl group.

Table S1. Calculated barriers for the aryl/alkyl migration vs Bmida migration. Energies relative to the corresponding difluoroiodoarene catalyst are indicated.

Entry	Catalyst Substrate	Bmida migration	Aryl/Alkyl migration
1	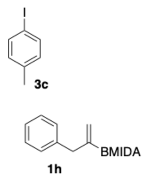 3c, 1h	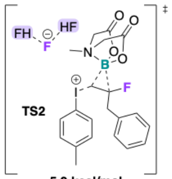 TS2, 5.9 kcal/mol	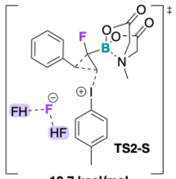 TS2-S, 12.7 kcal/mol
2	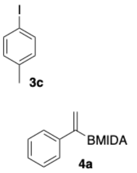 3c, 4a	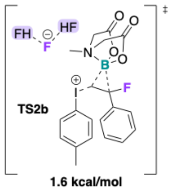 TS2b, 1.6 kcal/mol	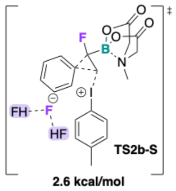 TS2b-S, 2.6 kcal/mol
3	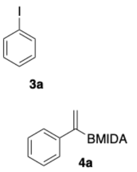 3a, 4a	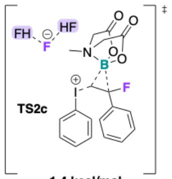 TS2c, 1.4 kcal/mol	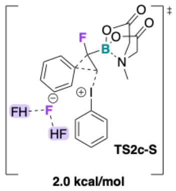 TS2c-S, 2.0 kcal/mol
4	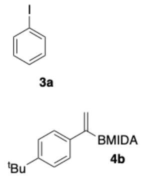 3a, 4b	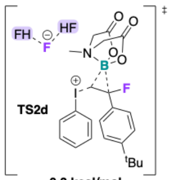 TS2d, 0.3 kcal/mol	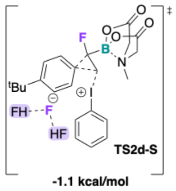 TS2d-S, -1.1 kcal/mol

Absolute energies and energy corrections

Stationary point	Electronic energy 6-31G(d,p)/LANL2DZ (a.u.)	Thermal correction to Gibbs free energy (a.u)	Single-point energy 6-311+G(2d,2p)/LANL2DZdp (a.u.)
**3c-F_2**	-481.978830721	0.082487	-482.189070365
**3-F_2-2HF**	-682.8843442	0.100432	-683.1939699
**Int1**	-682.8509335	0.095756	-683.1658412
**Int2**	-1606.754386	0.368061	-1607.341022
**TS1**	-1606.748706	0.371166	-1607.331425
**TS1-S**	-1606.741825	0.371046	-1607.326545
**Int3**	-1405.85395	0.352609	-1406.340778
**Int4**	-1606.805289	0.372428	-1607.374062
**TS2**	-1606.80327	0.370862	-1607.352947
**TS2-S**	-1606.787339	0.36766	-1607.338743
**TS2-F**	-1606.790675	0.372862	-1607.348314
**TS2b**	-1567.483787	0.342341	-1568.023756
**TS2b-S**	-1567.47957	0.342467	-1568.022229
**TS2c**	-1528.157520	0.318754	-1528.687939
**TS2c-S**	-1528.153318	0.318300	-1528.686641
**TS2d**	-1685.449646	0.424490	-1686.019301
**TS2d-S**	-1685.446349	0.423093	-1686.020121
**Int5**	-1324.45256	0.262948	-1324.907038
**TS3**	-1324.455333	0.265534	-1324.905846

Cartesian Coordinates

3c-F_2

C	-3.24214800	0.00003000	-0.01041300
C	-2.52427900	-1.20342700	-0.01013600
C	-1.12809800	-1.21956700	-0.00443000
C	-0.47031600	0.00002100	-0.00087500
C	-1.12808500	1.21960100	-0.00432900
C	-2.52427900	1.20347000	-0.01003300
H	-3.06096700	-2.14817600	-0.01630300
H	-0.56792800	-2.14511100	-0.00633900
H	-0.56788000	2.14512400	-0.00616500
H	-3.06096700	2.14821600	-0.01614600
I	1.68600300	-0.00001700	0.00151000
F	1.55178200	-2.01118800	0.00154300
F	1.55177800	2.01124700	0.00142300
C	-4.74923900	-0.00003000	0.01484000
H	-5.15630000	-0.88645400	-0.48012100
H	-5.12117500	-0.00220600	1.04708200
H	-5.15633100	0.88838200	-0.47648800

3c-F_2-2HF

C	3.57972900	0.38987200	-0.15356800
C	2.64001500	1.37969300	0.16513800
C	1.27515800	1.09708600	0.24125400
C	0.88225300	-0.20856700	-0.01389000
C	1.76729700	-1.23210900	-0.32062600
C	3.12435200	-0.91539100	-0.38502300
H	2.97497600	2.39270100	0.36873300
H	0.56556500	1.86614900	0.51585300
H	1.42028000	-2.24403200	-0.48832000
H	3.83640700	-1.70029600	-0.62301000
I	-1.19597300	-0.73393100	0.07564400
F	-1.50382700	1.25471600	-0.73410800
F	-0.62335900	-2.47705300	0.75843800
C	5.04372000	0.72630500	-0.26466000
H	5.31498400	1.54757800	0.40465700
H	5.67068100	-0.13781100	-0.02796300
H	5.29061400	1.04148200	-1.28616400
H	-3.05404600	1.31584900	-0.98354300
H	-1.47590100	2.26271400	0.40747800
F	-3.99609800	1.20208000	-0.96648600
F	-1.32472800	2.81278000	1.17453400

Int1

C	3.25007300	-0.37184600	-0.06555900
C	2.42325300	0.71557800	-0.38813000
C	1.04195700	0.58269500	-0.45400700
C	0.50254100	-0.67957100	-0.18384600
C	1.28706100	-1.79446000	0.14664700
C	2.66273400	-1.62501500	0.19461800
H	2.86252000	1.68890600	-0.58004200
H	0.40999600	1.41535300	-0.72491400
H	0.83339600	-2.75663100	0.35320400
H	3.29502400	-2.47228900	0.44171900
I	-1.56303600	-0.89471100	-0.21215900
F	-1.89487900	-0.52451200	1.64347900
C	4.73878100	-0.19791100	0.02959400
H	5.08423700	0.63083600	-0.59354100
H	5.02404900	0.02697000	1.06529700
H	5.26680600	-1.10963100	-0.26345500
F	-1.32737500	2.14975000	0.78676800
H	-1.38947700	2.31076300	-0.51644100
H	-0.11849800	2.78996900	0.87420100
F	-1.31157400	2.37629900	-1.52764000
F	0.77099100	3.23386600	0.82100500

Int2

C	-4.75648100	0.45547900	0.44697400
C	-4.11802400	1.22205100	1.42951600
C	-2.81379500	1.68425600	1.25134000
C	-2.17364600	1.36902300	0.05842600
C	-2.77339600	0.63827100	-0.96368800
C	-4.06926900	0.17862700	-0.74506400
H	-4.63668600	1.45034200	2.35582900
H	-2.32193800	2.26833300	2.02023700
H	-2.25696200	0.43388400	-1.89691600
H	-4.55416100	-0.40753600	-1.52016600
I	-0.20086500	2.12622200	-0.21154500
F	-0.98653600	3.93259200	0.06472000
C	0.45904200	-0.31962300	-0.91629100
H	-0.48560800	-0.75113900	-1.20734900
H	1.06500600	0.05043500	-1.73403300
C	0.97666800	-0.48756300	0.34256200
C	0.21691700	-1.17009100	1.45111800
H	0.92061400	-1.86049500	1.93679300
H	0.02034500	-0.41902600	2.22776300
C	-1.06052900	-1.90988200	1.11725500
C	-2.18920200	-1.73331400	1.92574100
C	-1.13476200	-2.81485100	0.04759300
C	-3.36791500	-2.43820000	1.67779100
H	-2.14558100	-1.03288700	2.75460500
C	-2.31819600	-3.50563200	-0.21296800
H	-0.28838400	-2.95209900	-0.61468400
C	-3.43740700	-3.32618500	0.60336500
H	-4.23242500	-2.28377500	2.31661700
H	-2.36297200	-4.18239900	-1.06117500
H	-4.35649700	-3.86793000	0.39939100
B	2.47965800	0.00320800	0.62348700
O	2.76421000	0.27316600	2.02573500
O	2.86812100	1.12798300	-0.25497800
C	4.00910000	-0.08744600	2.37091700
C	3.65014500	0.76106700	-1.28424400
O	4.49716800	0.09825100	3.45928900
O	4.01382700	1.50874500	-2.15973700
C	4.71541500	-0.74059400	1.18830900
H	5.39539400	-0.01013900	0.74256800
H	5.28558100	-1.61957100	1.48812700
C	3.96671300	-0.72505600	-1.19323500
H	3.29209000	-1.27537400	-1.85796700
N	3.63536100	-1.09794400	0.21227400
C	3.26190500	-2.53788700	0.33878300
H	2.97287100	-2.73692700	1.37133400
H	2.44356300	-2.74603800	-0.34862500
H	4.12992300	-3.14782400	0.08056600
H	5.00379300	-0.94989500	-1.44273600
F	-0.62772700	-2.12356200	-3.00760900
H	0.55042000	-2.38258200	-2.59751700
H	-0.76251300	-0.83004300	-3.31454500
F	-0.86616000	0.15909300	-3.47824300
F	1.49165900	-2.60233400	-2.21835100
C	-6.14458700	-0.08418300	0.66144600
H	-6.82383300	0.25178500	-0.12977100
H	-6.13770800	-1.17965700	0.63575900
H	-6.55418800	0.23350100	1.62342800

TS1

C	-5.28085100	0.11130700	0.76237600
C	-4.99024200	-0.83705200	-0.22709000
C	-3.71378800	-1.38215900	-0.35889000
C	-2.73081900	-0.96140400	0.52693100

C	-2.97246100	-0.04307100	1.53789300
C	-4.25920700	0.48947500	1.64232800
H	-5.76968800	-1.15437300	-0.91398300
H	-3.49507500	-2.13098300	-1.10807600
H	-2.20923500	0.25130200	2.24691900
H	-4.46486000	1.20709400	2.43122700
I	-0.78281500	-1.84948000	0.26037400
F	-1.89247800	-3.51459100	-0.26048000
C	0.22892300	0.24382100	0.97971000
H	-0.64243200	0.88526800	1.01001200
H	0.62662900	-0.04725900	1.94601200
C	1.17164700	0.48620600	-0.04542600
C	0.73825700	1.11233700	-1.34252500
H	1.38001400	1.98082800	-1.51161900
H	0.98564200	0.39943800	-2.13981300
C	-0.70403400	1.54564200	-1.45119900
C	-1.17989200	2.62090900	-0.68763300
C	-1.58232100	0.88166100	-2.31281900
C	-2.51692800	3.00790400	-0.77535900
H	-0.51440500	3.11877900	0.00914900
C	-2.91791100	1.27671200	-2.40960400
H	-1.21970000	0.04552000	-2.90475500
C	-3.38951400	2.33914500	-1.63761200
H	-2.87839000	3.83206100	-0.16711800
H	-3.59021500	0.74847400	-3.07897100
H	-4.43016700	2.64216800	-1.70439100
B	2.55638300	-0.34040700	0.07940600
O	2.38357000	-1.58616500	-0.67730900
O	2.90480900	-0.59267600	1.48346200
C	3.52981800	-2.02708200	-1.21665900
C	3.97676100	0.08986900	1.90444900
O	3.64109700	-3.05456900	-1.83920800
O	4.35527000	0.12475400	3.05123000
C	4.65484700	-1.05541400	-0.89853500
H	5.23635300	-1.46562100	-0.06912600
H	5.31616800	-0.89981300	-1.74992600
C	4.63533200	0.81218600	0.73947800
H	4.37326900	1.86751200	0.80365600
N	3.99524400	0.23195900	-0.47974600
C	3.99433700	1.18949300	-1.62509000
H	3.45716900	0.74054100	-2.46119600
H	3.51976300	2.11914900	-1.32489200
H	5.02860300	1.38107400	-1.91704700
H	5.71764500	0.68524000	0.72614100
F	2.16225100	2.22343200	0.86655500
H	2.23353900	3.23507600	-0.10655900
H	1.06260700	2.70011700	1.68376100
F	0.27752500	3.03884100	2.14372400
F	2.28687100	3.84618900	-0.87527700
C	-6.65047800	0.73001000	0.86169500
H	-7.41940600	0.06449200	0.45972800
H	-6.90528200	0.97070600	1.89787300
H	-6.69234200	1.66539200	0.28973700

TS1-S

C	-4.34460300	-1.99693100	0.87685200
C	-4.28088800	-0.66059300	0.45692800
C	-3.20764000	-0.18702300	-0.29633600
C	-2.19084100	-1.07862600	-0.62169300
C	-2.21000200	-2.41291200	-0.22515200
C	-3.29687000	-2.86006100	0.52604900
H	-5.07750200	0.02748400	0.72501700
H	-3.16571400	0.84940500	-0.60442200
H	-1.39987100	-3.08592900	-0.48079600
H	-3.32465500	-3.89718600	0.84729500
I	-0.54017000	-0.38800300	-1.77290000
F	-1.62915800	-1.03011900	-3.39725400
C	0.04227700	-0.26382400	1.18019600
H	-0.90058700	0.09230800	1.57628700
C	0.74747000	0.48712200	0.21785000
C	0.55855500	2.01464600	0.22237700
H	1.07347400	2.38890700	1.11247100
H	1.07358700	2.42707500	-0.64918100
C	-0.86300700	2.52953100	0.26090200
C	-1.50437400	2.95654800	-0.91049600
C	-1.56741700	2.59592800	1.47230800
C	-2.82280500	3.41446800	-0.87955300
H	-0.96398500	2.93225400	-1.85210700
C	-2.89057100	3.04089400	1.50430300
H	-1.05679800	2.33404100	2.39319800
C	-3.52515000	3.44645800	0.32832200
H	-3.30073400	3.74492000	-1.79726900
H	-3.42115400	3.08228000	2.45122200
H	-4.55247200	3.79769000	0.35363600
B	2.16569100	-0.08507800	-0.28154900
O	2.53487900	0.53106300	-1.56358600
O	2.19328800	-1.55956000	-0.35591900
C	3.84455500	0.76267100	-1.69240100
C	3.03725500	-2.15338800	0.49765900
O	4.36691400	1.22002600	-2.68131100
O	3.21939800	-3.34596700	0.55587400
C	4.57805000	0.34355900	-0.42787200
H	5.07569100	-0.61035100	-0.61975300
H	5.32798600	1.07630500	-0.13143100
C	3.69854300	-1.10669200	1.37807800
H	3.13263600	-1.06049300	2.30832300
N	3.53234300	0.17577400	0.63642900
C	3.53416800	1.35287900	1.55027400
H	3.41808500	2.25955600	0.95669200
H	2.71413300	1.25959500	2.25467600
H	4.48706200	1.38430500	2.08258200
H	4.74541900	-1.32449200	1.58751600
C	-5.52429200	-2.49748500	1.66798800
H	-5.26056300	-3.37403100	2.26595200
H	-5.91240000	-1.72306000	2.33604100
H	-6.34199000	-2.78942000	0.99728100
H	0.20250800	-1.33328000	1.24162300
F	0.89057100	-0.01288500	2.89067100
H	1.18921800	-1.43091800	3.14843300
H	0.90155000	1.37287000	3.33717700
F	0.97776100	2.31972200	3.52120400
F	1.42981600	-2.36207500	3.08715400

Int3

C	4.29260500	1.52613700	-0.48885700
C	3.47196700	1.31245800	-1.60015900
C	2.08478200	1.47495500	-1.52120800
C	1.53880300	1.86345900	-0.31010500
C	2.31893200	2.11806800	0.81269400
C	3.69524200	1.93817000	0.71383800
H	3.91292600	1.00629700	-2.54449100
H	1.46919300	1.29385100	-2.39368500
H	1.84640000	2.48990900	1.71140200
H	4.31675900	2.12333000	1.58611000
I	-0.58551900	2.06053300	-0.06683300
F	0.13022900	3.80459800	1.11244300
C	-0.98925200	0.17197100	-1.18689700
H	-1.95846300	0.37621000	-1.64473200
H	-0.23768200	0.06924800	-1.96595900
C	-1.05550000	-1.06901800	-0.30848700
C	-2.11660200	-0.96188800	0.79942300
H	-2.26313500	-1.95289600	1.23465000
H	-1.70101300	-0.33717100	1.59438800
C	-3.45262000	-0.40436900	0.36033900
C	-4.25079400	-1.09012400	-0.56841100
C	-3.91417900	0.81951600	0.86260000
C	-5.47267600	-0.56166500	-0.98485300
H	-3.90010800	-2.03284700	-0.97392600
C	-5.13825700	1.35066900	0.44819500
H	-3.30982700	1.35840600	1.58710100
C	-5.92124000	0.66169900	-0.47887500
H	-6.07651100	-1.10556500	-1.70598600
H	-5.47746800	2.30091700	0.85065700
H	-6.87295100	1.07172300	-0.80404600

B	0.41403700	-1.54434500	0.17180500
O	0.87393500	-0.87886900	1.38524400
O	1.39135200	-1.42897300	-0.93353000
C	1.65718400	-1.65762100	2.13794100
C	1.72282500	-2.58452500	-1.50556100
O	2.22513500	-1.29638200	3.14127700
O	2.41937800	-2.69238900	-2.48943400
C	1.74028100	-3.05799200	1.54169900
H	2.70634000	-3.16924700	1.04398100
H	1.64844400	-3.82969500	2.30571500
C	1.08163300	-3.74253200	-0.75384500
H	0.20707800	-4.08283100	-1.31141300
N	0.62716100	-3.15211400	0.53722800
C	-0.52627300	-3.89958400	1.11297300
H	-0.81043900	-3.43862800	2.05929300
H	-1.35325100	-3.85728900	0.40821800
H	-0.22733800	-4.93516000	1.28712100
H	1.76844300	-4.57552000	-0.60263300
F	-1.48988400	-2.12574000	-1.21723300
C	5.78497300	1.33030500	-0.57069300
H	6.12069400	0.56631400	0.14009500
H	6.09232400	1.01895400	-1.57251400
H	6.31927300	2.25462400	-0.32271700

Int4

C	4.63903200	-0.86006300	1.53283500
C	3.59125200	-1.15984700	2.41517000
C	2.31635200	-1.47667800	1.94289800
C	2.12428500	-1.49210200	0.56602000
C	3.12871800	-1.21363200	-0.35293800
C	4.38637900	-0.88744500	0.15353200
H	3.76715300	-1.13751800	3.48673500
H	1.50584500	-1.69193000	2.62937500
H	2.92468200	-1.22315200	-1.41721200
H	5.18487100	-0.64660700	-0.54238200
I	0.17664800	-1.95174700	-0.18544900
C	-0.28729800	0.03205100	-1.06467400
H	0.69796100	0.42243100	-1.32516200
H	-0.81910500	-0.29059700	-1.95787300
C	-1.12193400	0.90472200	-0.13708800
C	-0.29114100	1.65642800	0.90633900
H	-0.95854400	2.34467600	1.43496700
H	0.02738900	0.91702800	1.64685700
C	0.91733300	2.40284600	0.38543300
C	0.78580400	3.46203300	-0.52556400
C	2.20103400	2.04667600	0.81594600
C	1.91150000	4.13513400	-0.99956100
H	-0.19995600	3.75026100	-0.87231600
C	3.32885500	2.72123900	0.34551900
H	2.31969600	1.23066500	1.52093300
C	3.18791800	3.76653100	-0.56725100
H	1.79203700	4.94877800	-1.70942900
H	4.31523100	2.42288200	0.68810600
H	4.06351300	4.29004300	-0.94002900
B	-2.42367300	0.14516200	0.47103400
O	-2.22398100	-0.35049500	1.82855900
O	-2.89176400	-0.92729900	-0.43793300
C	-3.35019600	-0.31609400	2.55401300
C	-3.98675500	-0.60392500	-1.13873100
O	-3.45166800	-0.73837900	3.68049200
O	-4.48139800	-1.29557600	-1.99559400
C	-4.47657900	0.30937000	1.74170300
H	-5.11541200	-0.49081200	1.36002500
H	-5.08211600	0.99300100	2.33544700
C	-4.49531000	0.76437000	-0.70120000
H	-4.16513500	1.51124300	-1.42605300
N	-3.81108100	1.02345800	0.59860800
C	-3.70011600	2.48046500	0.90669500
H	-3.19905800	2.60048500	1.86819900
H	-3.12385300	2.96257100	0.12258800
H	-4.70415000	2.90454500	0.96283000
H	-5.58064900	0.79654200	-0.60562500

F	-1.63348500	1.86745300	-1.09076800
C	6.01468800	-0.53336600	2.05218500
H	6.65130600	-1.42687800	2.04779200
H	6.50635200	0.21794100	1.42730800
H	5.97560200	-0.16151600	3.07956400
F	1.80251600	-1.22333300	-3.18136400
H	0.48364200	-1.37332700	-3.45810700
H	2.07582400	0.04602900	-2.87247000
F	-0.50058700	-1.46932800	-3.62154200
F	2.28022500	0.96937300	-2.52772100

TS2

C	-0.68548000	4.25367300	0.06734400
C	-0.43393400	3.21663300	0.97670600
C	0.60361400	2.30672200	0.77472600
C	1.38349000	2.43235500	-0.37405100
C	1.17519900	3.45637300	-1.29371600
C	0.14256800	4.36877100	-1.05548400
H	-1.07245300	3.07199000	1.84270700
H	0.75752600	1.50984800	1.49230600
H	1.78857300	3.54306100	-2.18350900
H	-0.02897600	5.16864000	-1.77083000
I	2.80039200	0.87776700	-0.82495400
C	0.10734600	-0.36907200	-1.54992900
H	-0.01040200	0.70465600	-1.52030100
H	0.26283000	-0.84432100	-2.51406000
C	-0.24460800	-1.15495400	-0.46585200
C	0.22429200	-2.59881900	-0.32851800
H	-0.36554400	-3.09948900	0.43793700
H	0.04102100	-3.10013400	-1.28128900
C	1.69542100	-2.63308000	0.03100000
C	2.10582200	-2.37871500	1.34539300
C	2.65755500	-2.84417700	-0.96280000
C	3.46560000	-2.34253000	1.65644000
H	1.36074200	-2.20420100	2.11520500
C	4.01776000	-2.80583000	-0.64959900
H	2.34132800	-3.03548700	-1.98500900
C	4.42444900	-2.55144800	0.66249200
H	3.77643200	-2.14294600	2.67786100
H	4.75649400	-2.97439100	-1.42782000
H	5.48189500	-2.51871500	0.90800600
B	-2.03154200	-1.02113800	-1.00245100
O	-2.30686500	-2.05754100	-1.94186600
O	-2.39040900	0.31969900	-1.38019100
C	-3.51190500	-2.63091400	-1.69268800
C	-3.11684000	0.98526500	-0.44588700
O	-3.98916300	-3.50495900	-2.36499200
O	-3.46856100	2.12848800	-0.57312400
C	-4.15190000	-1.96463700	-0.48447300
H	-4.86169200	-1.21336400	-0.84140600
H	-4.67429700	-2.67678300	0.15231400
C	-3.37630500	0.08163800	0.73924900
H	-2.71467300	0.37776100	1.56468900
N	-3.02272300	-1.29458700	0.26671700
C	-2.61696900	-2.17392700	1.41209900
H	-2.41043700	-3.17267500	1.02806000
H	-1.74706400	-1.77160200	1.92407400
H	-3.46207300	-2.22336000	2.10075500
H	-4.41382000	0.13031000	1.07060400
F	-0.28352800	-0.49372700	0.74186300
F	0.26792700	0.37107400	3.23634200
H	-0.90552200	0.91335300	3.13331200
H	-0.02796800	-0.90547100	3.34460600
F	-0.28890900	-1.88588300	3.41465400
F	-1.84399000	1.31401800	3.03040000
C	-1.85109800	5.18381300	0.28037900
H	-1.82094700	6.03237400	-0.40872500
H	-2.79347700	4.64771800	0.11904100
H	-1.86949900	5.57169400	1.30433000

TS2-S

C	5.61696800	0.33480100	1.16206800
C	5.41663600	-1.04117400	1.32895500
C	4.34170300	-1.70026900	0.72510200
C	3.46231200	-0.96101600	-0.06087400
C	3.63270800	0.40741000	-0.25248100
C	4.70954700	1.04427800	0.36411100
H	6.10620600	-1.61317700	1.94422300
H	4.20026500	-2.76496500	0.87325600
H	2.94497800	0.99163500	-0.84800900
H	4.81933600	2.11234500	0.20432500
I	1.77051000	-1.93838600	-0.96036900
C	-0.03447900	0.36320300	0.23101000
H	0.42231800	-0.06378600	1.11475400
H	0.56364800	0.98767700	-0.43443200
C	-1.39735300	0.15207900	-0.03192200
C	-1.39113700	1.48902000	1.01793400
H	-1.84089500	1.08463600	1.92249200
H	-0.38102600	1.85021000	1.31889500
C	-2.09438100	2.65787600	0.38173000
C	-3.37196700	3.00340600	0.83533500
C	-1.47399500	3.43082100	-0.60732200
C	-4.03693700	4.10327700	0.29084300
H	-3.83962500	2.42545500	1.62604500
C	-2.14769800	4.52284000	-1.15410500
H	-0.46363700	3.19257000	-0.92771600
C	-3.42988900	4.85941600	-0.71285700
H	-5.02592500	4.36689700	0.65359500
H	-1.66320500	5.11860700	-1.92195700
H	-3.94770600	5.71247000	-1.14109400
B	-2.17238500	-1.19122600	0.42790000
O	-2.15692700	-1.38248700	1.86315700
O	-1.61894500	-2.32828900	-0.32342500
C	-3.28875400	-1.96369300	2.30473500
C	-2.41257400	-2.76194300	-1.31287700
O	-3.49043200	-2.27545500	3.45093200
O	-2.10447900	-3.61421900	-2.10982500
C	-4.24993400	-2.18409900	1.14298900
H	-4.20080800	-3.23420100	0.84461700
H	-5.27743000	-1.93875000	1.40924100
C	-3.73452400	-2.00490900	-1.29204100
H	-3.72925500	-1.25813900	-2.08934800
N	-3.75417000	-1.30558500	0.02717700
C	-4.53326000	-0.03361500	-0.00751500
H	-4.51760500	0.40816500	0.98754400
H	-4.07687700	0.64575300	-0.72284200
H	-5.56236600	-0.25524700	-0.29459500
H	-4.59370000	-2.66375300	-1.41648500
F	-1.77573700	0.54064700	-1.30098400
C	6.77935900	1.04040700	1.81322400
H	7.32518300	0.37476600	2.48751600
H	7.48603700	1.41101500	1.06111300
H	6.44185000	1.90896900	2.38931900
F	1.51533700	2.54150900	-0.97967200
H	1.55909000	2.57303200	0.39289800
H	2.79185500	3.14474300	-1.20810100
F	1.51393900	2.47996600	1.38470000
F	3.69707300	3.50095200	-1.29275600

TS2-F

C	-4.53087100	-1.40098800	-0.86157300
C	-3.65923600	-1.56393500	-1.94837300
C	-2.31551200	-1.88204100	-1.75830100
C	-1.85209200	-2.03300400	-0.45465600
C	-2.68367100	-1.89804900	0.65050000
C	-4.02614400	-1.58403700	0.43156800
H	-4.03188000	-1.43235700	-2.96042600
H	-1.65099100	-1.99402900	-2.60728800
H	-2.30331500	-1.99993300	1.65832200
H	-4.68420500	-1.46702400	1.28801800
I	0.23675200	-2.38001200	-0.13727200
C	1.04388300	-0.08363600	-1.01728500
H	1.95586800	-0.55137300	-1.34949700
H	0.19726500	-0.06322900	-1.68343500
C	1.03981700	0.80461700	0.23405800
C	1.97109700	0.30458100	1.35332900
H	2.19208800	1.18377400	1.96838900
H	1.41223100	-0.38238700	1.99032900
C	3.25798500	-0.35551800	0.91078800
C	3.58750400	-1.62755100	1.39837400
C	4.12672500	0.26488900	-0.00062300
C	4.76181200	-2.26824000	0.99549600
H	2.91873300	-2.11609500	2.10239200
C	5.29532700	-0.37970700	-0.40821400
H	3.87965500	1.23283600	-0.42120500
C	5.62027300	-1.64479100	0.08874700
H	5.00138100	-3.25286100	1.38695800
H	5.95060800	0.11035900	-1.12292400
H	6.53181200	-2.14117800	-0.23130300
B	-0.48526600	1.13149200	0.71406900
O	-0.92052700	0.24495500	1.79232700
O	-1.43076700	1.13161000	-0.41448600
C	-1.78134200	0.83061700	2.62803400
C	-1.91913200	2.33912600	-0.73311900
O	-2.37391500	0.25746800	3.51256900
O	-2.74701600	2.53247200	-1.58979600
C	-1.91742300	2.30750600	2.28974800
H	-2.88111100	2.46493900	1.79923400
H	-1.87234500	2.93401800	3.17993700
C	-1.27902000	3.39468400	0.15675500
H	-0.41965000	3.78904100	-0.38736900
N	-0.79483100	2.62801500	1.34191700
C	0.30581300	3.32197200	2.07194200
H	0.61500600	2.69739600	2.91129500
H	1.13838400	3.47332300	1.39170500
H	-0.06529500	4.27875900	2.44382000
H	-1.96484100	4.19265300	0.43974600
F	1.59123400	2.04929300	-0.21678700
C	-5.96974500	-1.01595100	-1.08809400
H	-6.43492700	-1.64752200	-1.85218400
H	-6.55535400	-1.09988200	-0.16887900
H	-6.04117500	0.02044400	-1.43932200
F	1.68908400	1.24265700	-2.58287800
H	2.84860200	2.02421700	-2.27009900
H	0.81819800	2.34784600	-2.45226600
F	0.23332800	3.13372800	-2.38287000
F	3.62069600	2.59321300	-2.07766900

TS2b

C	4.52952100	1.21228300	-1.32897000
C	4.42758700	1.49839800	0.04001300
C	3.71015900	0.67026300	0.90231100
C	3.09210600	-0.46144600	0.37563800
C	3.17094200	-0.78581700	-0.97384600
C	3.89436500	0.06394800	-1.81524700
H	4.90749000	2.38765700	0.44029100
H	3.62965900	0.91219400	1.95576400
H	2.66973200	-1.66278600	-1.36632800
H	3.95594900	-0.17468600	-2.87362400
I	1.96013900	-1.73200000	1.68906300
C	-0.73179100	-1.06551700	0.47245100
H	-0.35022100	-1.25149900	-0.53059600
H	-1.09936100	-1.89771900	1.05624300
C	-0.95883000	0.21970500	0.91276400
B	-2.57759500	0.46757000	-0.09684900
O	-2.29348600	0.53006600	-1.47790500
O	-3.00791800	1.68596600	0.51679600
C	-3.29789300	0.01868000	-2.22899200
C	-4.17903000	1.59008700	1.18052700
O	-3.33659500	0.08122400	-3.42756600
O	-4.68397400	2.50208100	1.78143800
C	-4.39811500	-0.52360900	-1.33228200

H	-5.25911800	0.14526300	-1.38984300
H	-4.69203000	-1.52581800	-1.63617600
C	-4.73061200	0.18366300	1.06541200
H	-4.64807200	-0.30275000	2.03912400
N	-3.85248300	-0.53459100	0.08188000
C	-3.63918100	-1.95163900	0.53141500
H	-2.98949900	-2.44453900	-0.18855800
H	-3.20753600	-1.93666800	1.53085800
H	-4.61289900	-2.44316500	0.56063600
H	-5.77370600	0.17647000	0.75166800
F	-1.40851100	0.30974200	2.21404100
C	5.27982000	2.14068200	-2.24939700
H	6.28841500	2.34507200	-1.87378400
H	5.36830500	1.72038700	-3.25465100
H	4.76673700	3.10639700	-2.33323700
C	-0.14950500	1.40358600	0.49107200
C	-0.01416300	2.48180000	1.37217900
C	0.48087600	1.42967800	-0.76219200
C	0.77315700	3.57536000	1.01205700
H	-0.51450000	2.46002700	2.33236500
C	1.25162600	2.53334400	-1.11539400
H	0.37267000	0.60025800	-1.45236700
C	1.40505100	3.60512100	-0.23156900
H	0.88574500	4.40480200	1.70352300
H	1.74420900	2.54830600	-2.08176100
H	2.01432700	4.45889800	-0.51268000
F	-0.00023400	-1.36990200	-2.36184100
H	-1.22079500	-1.89064000	-2.25124800
H	0.77627600	-2.47631300	-2.03361400
F	-2.12234900	-2.30540900	-2.04939900
F	1.31281400	-3.24387100	-1.71958600

TS2b-S

C	3.71264700	-0.73320600	-1.86940100
C	3.43101500	0.46592200	-1.19911000
C	2.80411200	0.46760600	0.04663700
C	2.45556300	-0.75396900	0.61367500
C	2.70754300	-1.96498600	-0.01919400
C	3.33984900	-1.94011000	-1.26568400
H	3.68510600	1.42293800	-1.64419900
H	2.55829700	1.40205900	0.53229200
H	2.39585900	-2.90517100	0.41698100
H	3.53225000	-2.88040600	-1.77524700
I	1.41739800	-0.75577400	2.49840400
C	-0.64934700	0.91868200	1.39025100
H	-1.26489800	0.74095800	2.26368700
H	0.13194500	1.68205800	1.42941000
C	-1.06985800	0.38988000	0.11216200
F	-0.05620100	0.50228700	-0.84517100
C	4.40899200	-0.71613700	-3.20664000
H	5.48411500	-0.53375500	-3.08605700
H	4.29241800	-1.66863600	-3.73157000
H	4.01935400	0.08172000	-3.84705300
B	-1.75480700	-1.06542100	-0.08626300
O	-2.95422500	-1.25945600	0.71039000
O	-0.78467100	-2.14496300	0.10555300
N	-2.25487400	-1.34822600	-1.63447600
C	-3.94559800	-1.85219600	0.02514500
C	-0.45008600	-2.79420400	-1.01546600
C	-3.49449000	-2.16286900	-1.39461000
C	-1.14092000	-2.15918100	-2.21264100
C	-2.57074500	-0.15696800	-2.48140300
O	-5.02822800	-2.11350100	0.48833900
O	0.30412900	-3.73655400	-1.06326300
H	-3.26231400	-3.22891600	-1.45840400
H	-4.26743600	-1.92719000	-2.12502000
H	-0.43372500	-1.48782200	-2.70492200
H	-1.50411200	-2.89600900	-2.92854300
H	-3.38405800	0.40043300	-2.01856100
H	-1.68269800	0.46653100	-2.55591300
H	-2.87447800	-0.50259800	-3.47097400
C	-1.94589900	1.68191200	0.08981400
C	-1.39988800	2.82771900	-0.52977800
C	-3.25391200	1.71409200	0.62370200
C	-2.19132100	3.95981900	-0.68012100
H	-0.37280500	2.81976000	-0.88098100
C	-4.02676100	2.85646200	0.46516400
H	-3.64808600	0.85360200	1.14786000
C	-3.50176000	3.97504500	-0.19313900
H	-1.77918800	4.83753300	-1.16714800
H	-5.03645500	2.88079700	0.86144500
H	-4.11147300	4.86569900	-0.31028100
F	1.56549400	3.27635700	-0.92182800
H	2.86854600	3.47612600	-1.34302800
H	1.51270500	3.19080000	0.35006700
F	3.80906300	3.57945000	-1.63234500
F	1.45568000	3.04480900	1.37479800

TS2c

C	-4.52184900	0.98135300	2.11442400
C	-4.50178900	1.68432200	0.90842000
C	-3.88830000	1.13436300	-0.22008600
C	-3.30333800	-0.12403000	-0.10734900
C	-3.30347400	-0.84711600	1.08222300
C	-3.92232700	-0.27673500	2.19809300
H	-4.95831300	2.66705800	0.83852600
H	-3.86222600	1.68105700	-1.15541100
H	-2.82283200	-1.81638700	1.14259200
H	-3.92813900	-0.82457900	3.13572400
I	-2.35053300	-0.97960200	-1.83511500
C	0.47641500	-0.87199400	-0.71878800
H	0.15211700	-1.31911700	0.22033400
H	0.73333000	-1.51734900	-1.54708800
C	0.77314600	0.47051500	-0.79677100
B	2.46051000	0.33372900	0.10268000
O	2.27435400	0.01579500	1.46549200
O	2.94803700	1.64969400	-0.17748500
C	3.30276100	-0.71585200	1.95791300
C	4.04764000	1.67973500	-0.96001400
O	3.43450100	-0.99117700	3.11915800
O	4.58083300	2.69439000	-1.32576500
C	4.30294600	-1.01672300	0.85332300
H	5.18946800	-0.39799400	1.00850200
H	4.58778300	-2.06682700	0.85179200
C	4.47591200	0.26863100	-1.30853000
H	4.24429700	0.08538000	-2.35973000
N	3.64029200	-0.64126500	-0.45718800
C	3.29242700	-1.88537000	-1.22177900
H	2.67353500	-2.51475300	-0.58535500
H	2.77730200	-1.59777500	-2.13684200
H	4.22392900	-2.39657100	-1.47107700
H	5.54144200	0.10840600	-1.14504800
F	1.14708900	0.90104900	-2.05324800
C	0.08080600	1.52722200	0.00225200
C	-0.01670400	2.82056800	-0.52200200
C	-0.48388600	1.22331000	1.25020000
C	-0.70348100	3.80470100	0.18874200
H	0.43362500	3.05055500	-1.47963800
C	-1.15501300	2.21799300	1.95527300
H	-0.40126400	0.22466600	1.66508100
C	-1.27223500	3.50676900	1.42753900
H	-0.78757200	4.80458900	-0.22585400
H	-1.60073400	1.97897900	2.91484100
H	-1.80419800	4.27467100	1.98110300
F	-0.08720800	-1.92271300	1.97275900
H	1.09230900	-2.46087200	1.65072200
H	-0.94123100	-2.85407000	1.39603200
F	1.94985800	-2.86084900	1.29499500
F	-1.53530700	-3.47584400	0.90897500
H	-4.99862700	1.41565600	2.98779700

TS2c-S

C	-3.62228400	-0.79886500	2.38873000
C	-3.44004400	0.38405200	1.66932300
C	-2.93125500	0.34555300	0.36851900
C	-2.61096200	-0.89272000	-0.17733300
C	-2.77444300	-2.08691400	0.51700800
C	-3.28889500	-2.02767800	1.81595700
H	-3.67642400	1.35217600	2.09842000
H	-2.75816600	1.26239700	-0.17860800
H	-2.48622600	-3.03478800	0.08150000
H	-3.41688000	-2.94926300	2.37589900
I	-1.76747300	-0.95211500	-2.15838100
C	0.33061100	0.85317300	-1.37519100
H	0.84925500	0.65494700	-2.30550100
H	-0.47868800	1.58740200	-1.35551200
C	0.92292100	0.40973400	-0.13216200
F	0.02339800	0.53596200	0.93260700
B	1.68353400	-1.01106700	0.04818000
O	2.79566200	-1.20028200	-0.86742900
O	0.73723200	-2.12883700	0.01471400
N	2.36206300	-1.21227800	1.53676600
C	3.86484000	-1.76123000	-0.27765400
C	0.54036200	-2.72058600	1.19916000
C	3.57237300	-2.03366200	1.19110200
C	1.33451500	-2.00084400	2.27950400
C	2.76340300	0.02353800	2.27363000
O	4.89452600	-2.02347900	-0.84792900
O	-0.17632800	-3.67640200	1.37695800
H	3.34946800	-3.09738600	1.30839500
H	4.41738800	-1.77750900	1.82930500
H	0.66692000	-1.31092500	2.80049100
H	1.78665200	-2.68526200	2.99661500
H	3.49872800	0.56707400	1.68095600
H	1.88152700	0.63984000	2.43225900
H	3.19928500	-0.26441400	3.23166600
C	1.74088400	1.73079400	-0.27319400
C	1.22897800	2.88699000	0.35598800
C	2.96878300	1.78239500	-0.97003000
C	1.98515600	4.05261700	0.35194000
H	0.25525100	2.86065800	0.83444300
C	3.70732800	2.95805500	-0.96450600
H	3.33043600	0.90971600	-1.49755700
C	3.22274200	4.08949000	-0.29760900
H	1.60099000	4.93893100	0.84620100
H	4.65737300	2.99755100	-1.48696800
H	3.80473800	5.00599000	-0.30011300
F	-1.68899300	3.24386300	1.09838200
H	-2.94284100	3.41323600	1.65710600
H	-1.77791800	3.10207500	-0.16547800
F	-3.84911900	3.49332600	2.04652900
F	-1.83182200	2.91157600	-1.18386400
H	-4.01690500	-0.76293600	3.39994000

TS2d

C	-4.42332200	-0.92868300	1.53566600
C	-4.51272700	-0.72146600	0.15768100
C	-3.55430000	-1.26164400	-0.70281200
C	-2.52282100	-2.01664600	-0.15081100
C	-2.41039300	-2.24627700	1.21717200
C	-3.37463600	-1.68622600	2.05991600
H	-5.32534400	-0.13283800	-0.25542600
H	-3.61285100	-1.09118300	-1.77145700
H	-1.59207000	-2.83303100	1.61501900
H	-3.29781000	-1.84863400	3.13088600
I	-1.03518400	-2.84931000	-1.46009100
C	1.27882300	-1.24662700	-0.39098500
H	0.98141400	-1.43150600	0.64145100
H	1.88944200	-1.98140700	-0.89620300
C	1.09822300	-0.00653800	-0.96828700
B	2.52548000	0.83646900	-0.03450000
O	2.21379400	0.96425100	1.33877000
O	2.58994800	2.05955600	-0.78029700
C	3.32691800	0.94115600	2.10962900
C	3.73172500	2.22622400	-1.47965500
O	3.33488900	1.18173200	3.28616400
O	3.94724900	3.17010400	-2.19442700
C	4.55316600	0.68871900	1.24729800
H	5.11607300	1.62052600	1.16022900
H	5.18854300	-0.08264100	1.67756100
C	4.66356600	1.05530300	-1.23916400
H	4.68506300	0.43644100	-2.13850000
N	4.05256700	0.26764100	-0.11966700
C	4.30426000	-1.19623800	-0.32352900
H	3.82479700	-1.74371000	0.48544400
H	3.91841800	-1.48418900	-1.29957600
H	5.38354400	-1.35724100	-0.30011500
H	5.67753600	1.37473400	-1.00007800
F	1.50929600	0.07515900	-2.28467700
C	-0.04020100	0.90712100	-0.65482400
C	-0.42328200	1.87295900	-1.58693500
C	-0.72318600	0.81874100	0.56829700
C	-1.48033900	2.73855900	-1.30514600
H	0.10466800	1.95459600	-2.52917800
C	-1.77235900	1.68929100	0.83208600
H	-0.42878300	0.08909300	1.31438000
C	-2.16972800	2.67725800	-0.08790700
H	-1.74877100	3.47733900	-2.05029900
H	-2.28795800	1.59746500	1.78170200
F	0.63542200	-1.51136800	2.46599600
H	1.97526200	-1.54857200	2.45655900
H	0.41708200	-2.86005400	2.21899100
F	2.97358300	-1.64218900	2.34125700
F	0.26399400	-3.79983100	1.95427800
H	-5.16954400	-0.50077300	2.19792700
C	-3.29608600	3.65195100	0.27924100
C	-3.57538000	4.66950400	-0.83792900
H	-3.89056800	4.17944900	-1.76509000
H	-4.38220400	5.34080600	-0.52690300
H	-2.69699600	5.28538400	-1.05663700
C	-2.89024700	4.42611300	1.55271700
H	-3.67937400	5.13320400	1.83179600
H	-2.72686900	3.75401100	2.40012300
H	-1.96719000	4.99208900	1.38859200
C	-4.59379500	2.86479400	0.55522200
H	-4.46486100	2.14137600	1.36436400
H	-5.39701000	3.55278100	0.84182600
H	-4.91518800	2.31893200	-0.33741900

TS2d-S

C	3.94070300	-1.81026100	2.35692900
C	2.97843400	-2.53956500	1.65521100
C	2.61913100	-2.16488500	0.35807400
C	3.23886000	-1.05281600	-0.20122200
C	4.19456800	-0.30180100	0.47502400
C	4.54387800	-0.69608700	1.77040600
H	2.48337500	-3.39886400	2.09548700
H	1.84966900	-2.70655000	-0.17530800
H	4.63575000	0.58037200	0.02973000
H	5.28299200	-0.11812300	2.31712000
I	2.63679500	-0.43048800	-2.17313300
C	-0.05772900	-0.25985800	-1.36325500
H	-0.32094800	0.23471800	-2.29078000
H	-0.01186800	-1.35092100	-1.33323400
C	-0.17317300	0.48367700	-0.12086300
F	0.41357400	-0.21583200	0.94425300
B	0.27242200	2.03318000	0.03691100
O	-0.40303400	2.93659300	-0.87925900
O	1.73000400	2.18296900	-0.01280400
N	-0.05958500	2.66268400	1.52584200
C	-0.77325100	4.08776000	-0.29506500
C	2.29578100	2.47337400	1.16410200
C	-0.36131500	4.09144600	1.17038700
C	1.23573600	2.51797200	2.25492300
C	-1.19968000	2.06523700	2.28529200
O	-1.33419600	4.98965200	-0.86680900

O	3.47809900	2.65845500	1.33003300
H	0.53755400	4.70402100	1.27740500
H	-1.14281600	4.49968900	1.81035200
H	1.24010500	1.56135200	2.78201000
H	1.39542500	3.32802100	2.96608700
H	-2.11532400	2.19098400	1.70801700
H	-1.00043200	1.00823800	2.44569900
H	-1.29161300	2.58069400	3.24271700
C	-1.67705400	0.10935700	-0.20947500
C	-2.10375800	-1.07463900	0.43126000
C	-2.62091300	0.91213800	-0.88038000
C	-3.45310100	-1.38394300	0.46388800
H	-1.37784500	-1.73647200	0.89231500
C	-3.96807800	0.57784900	-0.83665700
H	-2.29998300	1.79161800	-1.42333400
C	-4.41843700	-0.56716100	-0.15698500
H	-3.75997300	-2.29000500	0.97480100
H	-4.67298400	1.22093800	-1.34815900
F	-0.28209300	-3.40823500	1.11239800
H	0.51905600	-4.38310200	1.67358600
H	-0.12978500	-3.35704700	-0.15258200
F	1.12538600	-5.05958000	2.06808500
F	0.03602800	-3.25182100	-1.17091600
H	4.21526100	-2.10516100	3.36553600
C	-5.89741600	-0.95288800	-0.08266500
C	-6.80103000	0.03908800	-0.83123200
H	-6.55956500	0.08484300	-1.89805200
H	-7.84335100	-0.28158800	-0.74107800
H	-6.72824500	1.05017900	-0.41754400
C	-6.08714500	-2.35427000	-0.70440900
H	-5.51122900	-3.11813400	-0.17466400
H	-7.14363100	-2.63922500	-0.65912300
H	-5.77635600	-2.36180500	-1.75423800
C	-6.32795500	-0.98615600	1.40091400
H	-7.38476400	-1.26392500	1.47467600
H	-5.74840800	-1.71338600	1.97649500
H	-6.20056600	-0.00370100	1.86775000

Int5

C	0.50668100	-1.04814000	-0.63883300
H	1.23645600	-1.63917300	-0.06664700
H	0.71204800	-0.97945000	-1.70596100
C	-0.82950900	-1.36355900	-0.37638100
C	-1.98537300	-1.17402800	-1.27520700
H	-1.67765900	-0.51060200	-2.08609800
H	-2.14608800	-2.16698500	-1.72787000
C	-3.24273300	-0.70080600	-0.57257700
C	-3.48319500	0.67057900	-0.43119600
C	-4.15354300	-1.62269500	-0.04763700
C	-4.63296700	1.11302700	0.22365700
H	-2.77006100	1.38541400	-0.82846800
C	-5.30188200	-1.17747100	0.60724500
H	-3.96688300	-2.68695200	-0.15856300
C	-5.54326700	0.19110200	0.74361900
H	-4.81592600	2.17819200	0.32731500
H	-6.00830700	-1.89853700	1.00683200
H	-6.43825200	0.53738300	1.25146800
B	0.64241800	0.53014200	-0.01771600
O	0.44963000	0.53818500	1.41687100
O	-0.21905400	1.43794200	-0.74639500
C	1.59480000	0.73075800	2.10700800
C	0.37339900	2.62692400	-0.97080800
O	1.66073200	0.72274900	3.31007600
O	-0.16979700	3.56232600	-1.50064600
C	2.75762600	0.92718100	1.15237100
H	3.39172100	1.76364800	1.44636300
H	3.36721600	0.01845000	1.10558700
C	1.79296400	2.60660700	-0.42659100
H	2.50199900	3.06597900	-1.11404300
N	2.13114200	1.15895200	-0.18771400
C	2.96182700	0.63293500	-1.31627300
H	3.11789300	-0.43472900	-1.20211600
H	2.43674900	0.83568600	-2.25062200
H	3.91936200	1.15476800	-1.30985500
H	1.80774500	3.15084800	0.52043500
F	-1.11645100	-1.82742300	0.80927400
F	2.77267500	-2.67146300	0.29339200
H	3.88450100	-1.95015700	0.47401600
H	2.78346400	-2.62848500	-1.07802400
F	2.76697500	-2.45263100	-2.05687000
F	4.67378800	-1.32879200	0.54189200

TS3

C	0.20478900	-1.19582000	0.07973300
H	0.63192100	-2.13573600	-0.28076900
H	-0.40744800	-0.70168600	-0.67288300
C	-0.57101000	-1.45439400	1.23202500
C	-1.86495100	-0.89111500	1.60193900
H	-2.55898500	-1.69485900	1.25590900
H	-1.94453600	-0.86993100	2.69429200
C	-2.23581300	0.41257700	0.93138400
C	-3.06012000	0.41243100	-0.19919000
C	-1.75231100	1.62412200	1.43685500
C	-3.38789700	1.61578900	-0.82321100
H	-3.44417600	-0.52888900	-0.56902200
C	-2.08073100	2.82578400	0.80957900
H	-1.10772100	1.62643000	2.30934300
C	-2.89717800	2.82308500	-0.32342100
H	-4.02601700	1.60730500	-1.70162300
H	-1.68966900	3.75896300	1.20031600
H	-3.15097500	3.75887000	-0.81253800
B	1.53320100	-0.24044800	0.34898300
O	2.61459900	-0.97253900	0.97625100
O	1.25265300	1.02763300	1.01281100
C	3.80514700	-0.67903400	0.42447400
C	1.29842000	2.08917700	0.18984800
O	4.86178300	-1.10442600	0.81915200
O	1.12803900	3.23018900	0.54003300
C	3.62483400	0.28075600	-0.74563300
H	3.92420700	1.28247000	-0.42749800
H	4.22766100	-0.01042700	-1.60498600
C	1.57812700	1.62580300	-1.23350200
H	0.62773700	1.54408600	-1.76818800
N	2.15926700	0.25927900	-1.08145200
C	1.91259700	-0.60427700	-2.27867200
H	2.33001200	-1.59373400	-2.08577700
H	0.84098000	-0.67540300	-2.46750300
H	2.41728500	-0.15634700	-3.13617600
H	2.24631600	2.29680900	-1.77223000
F	-0.12162000	-2.36272800	2.05584000
F	-1.85494800	-2.61520300	-1.29648400
H	-2.93230600	-2.65971300	-0.58466400
H	-1.60514800	-1.53975600	-2.01052600
F	-1.33285200	-0.68032100	-2.48742200
F	-3.74169300	-2.63922800	0.04590500

References

[1] J. H. Docherty, K. Nicholson, A. P. Dominey, S. P. Thomas, *ACS Catal.* **2020**, *10*, 4686.

[2] J. R. Coombs, L. Zhang, J. P. Morken, *Org. Lett.* **2015**, *17*, 1708.

[3] Y. M. Ivon, Y. O. Kuchkovska, Z. V. Voitenko, O. O. Grygorenko, *Eur. J. Org. Chem.* **2020**, 3367.

[4] N. J. O'Brien, N. Kano, N. Havare, R. Uematsu, R. Ramozzi, K. Morokuma, *Eur. J. Inorg. Chem.* **2020**, 1995.

[5] S.-I. Kawaguchi, Y. Gonda, H. Masuno, H. T. Vu, K. Yamaguchi, H. Shinohara, M. Sonoda, A. Ogawa, *Tetrahedron Letters*, **2014**, *55*, 6779.

[6] C. Shu, A. Noble, V. K. Aggarwal, *Angew. Chem. Int. Ed.* **2019**, *58*, 3870.

[7] D. P. Ojha, K. R. Prabhu, *Org. Lett.* **2016**, *18*, 432.

[8] K. Hong, X. Liu, J. P. Morken, *J. Am. Chem. Soc.* **2014**, *136*, 10581.

[9] H.-Y. Wang, L. L. Anderson, *Org. Lett.* **2013**, *15*, 3362.

[10] M. L. Lepage, S. Lai, N. Peressin, R. Hadjerci, B. O. Patrick, D. M. Perrin, *Angew. Chem. Int. Ed.* **2017**, *56*, 15257.

[11] S. González-Granda, D. Méndez-Sánchez, I. Lavandera, V. Gotor-Fernández, *ChemCatChem* **2020**, *12*, 520.

[12] L. Mao, R. Bertermann, K. Emmert, K. J. Szabó, T. B. Marder, *Org. Lett.* **2017**, *19*, 6586.

[13] F. Gao, A. H. Hoveya, *J. Am. Chem. Soc.* **2010**, *132*, 10961.

[14] A. D. Becke, *Phys. Rev. A* **1988**, *38*, 3098.

[15] A. D. Becke, *J. Chem. Phys.* **1993**, *98*, 5648.

[16] S. Grimme, J. Antony, S. Ehrlich, H. Krieg, *J. Chem. Phys.* **2010**, *132*, 154104.

[17] S. Grimme, S. Ehrlich, L. Goerigk, *J. Comput. Chem.* **2011**, *32*, 1456.

[18] M. J. Frisch, G. W. Trucks, H. B. Schlegel, G. E. Scuseria, M. A. Robb, J. R. Cheeseman, G. Scalmani, V. Barone, G. A. Petersson, H. Nakatsuji, X. Li, M. Caricato, A. V. Marenich, J. Bloino, B. G. Janesko, R. Gomperts, B. Mennucci, H. P. Hratchian, J. V. Ortiz, A. F. Izmaylov, J. L. Sonnenberg, D. Williams-Young, F. Ding, F. Lipparini, F. Egidi, J. Goings, B. Peng, A. Petrone, T. Henderson, D. Ranasinghe, V. G. Zakrzewski, J. Gao, N. Rega, G. Zheng, W. Liang, M. Hada, M. Ehara, K. Toyota, R. Fukuda, J. Hasegawa, M. Ishida, T. Nakajima, Y. Honda, O. Kitao, H. Nakai, T. Vreven, K. Throssell, J. A. Montgomery, Jr., J. E. Peralta, F. Ogliaro, M. J. Bearpark, J. J. Heyd, E. N. Brothers, K. N. Kudin, V. N. Staroverov, T. A. Keith, R. Kobayashi, J. Normand, K. Raghavachari, A. P. Rendell, J. C. Burant, S. S. Iyengar, J. Tomasi, M. Cossi, J. M. Millam, M. Klene, C. Adamo, R. Cammi, J. W. Ochterski, R. L. Martin, K. Morokuma, O. Farkas, J. B. Foresman, D. J. Fox, Gaussian, Inc., Wallingford CT, 2016.

[19] P. J. Hay, W. R. Wadt, *J. Chem. Phys.* **1985**, *82*, 299.

[20] A. V. Marenich, C. J. Cramer, D. G. Truhlar, *J. Chem. Phys B* **2009**, *113*, 6378.

[21] B. P. Pritchard, D. Altarawy, B. Didier, T. D. Gibson, T. L. Windus, *J. Chem. Inf. Model.* **2019**, *59*, 4814.

[22] W. X. Lv, Q. Li, J. L. Li, Z. Li, E. Lin, D. H. Tan, Y. H. Cai, W. X. Fan, H. Wang, *Angew. Chem. Int. Ed.* **2018**, *57*, 16544.
